# Steric pressure in heteropentacenes modulates the photophysical properties – a molecular design strategy for functional materials †

**DOI:** 10.1039/d5sc03028e

**Published:** 2025-07-15

**Authors:** Alexander Huber, Tobias Thiele, Tobias Rex, Constantin Daniliuc, Christoph Wölper, Rick Y. Lorberg, Lea Höfmann, Cristian A. Strassert, Michael Giese, Jens Voskuhl

**Affiliations:** a Faculty of Chemistry (Organic Chemistry), CENIDE and Center of Medical Biotechnology (ZMB), University of Duisburg-Essen Universitätsstraße 7 45141 Essen Germany jens.voskuhl@uni-due.de; b Faculty of Chemistry (Organic Chemistry), CENIDE and Co-Creationlab for Product Innovations (CCLP), University of Duisburg-Essen Universitätsstraße 7 45141 Essen Germany michael.giese@uni-due.de; c Institut für Anorganische und Analytische Chemie, CeNTech, CiMIC, SoN, Universität Münster Heisenbergstraße 11 48149 Münster Germany; d Organisch-Chemisches Institut, Universität Münster Corrensstraße 40 48149 Münster Germany; e Faculty of Chemistry (Inorganic Chemistry), University of Duisburg-Essen Universitätsstraße 7 45141 Essen Germany

## Abstract

Discovering the versatile ability of environment-independent solution and solid-state emission (SSSE) enabled new possibilities of fine-tuning photophysical properties, targeting specific organelles, or developing remarkable materials. Herein, we report an unprecedented design concept for SSSE by employing the “magic methyl” effect in a series of alkylated heteropentacyclic luminophores R8, Y8, and G8. Implementing an increasing amount of *ortho*-methyl groups influences the vertical electronic transitions, tuning the emission colors from red over yellow to green and inverting the preferred state of luminescence from solution to solely the solid-state or even both. An in-depth analysis was performed using X-ray diffractometric structure elucidation, packing analysis and density functional theory calculations to correlate the photophysical properties with the steric pressure induced by the methyl groups. Additionally, the application scope of these new materials was investigated. Mesoporous silica nanoparticles loaded with the three new luminophores were prepared and employed as additives for 3D printing using digital light processing. Ultimately, these stimuli-responsive molecules performed as optical sensors of microenvironmental temperature and phase transition changes in liquid crystals.

## Abstract 2

Steric pressure induced by methyl groups modulates the emissive states (solution to solid) and colors (red to green) of heteropentacyclic luminophores for sensing applications.

## Graphical abstract

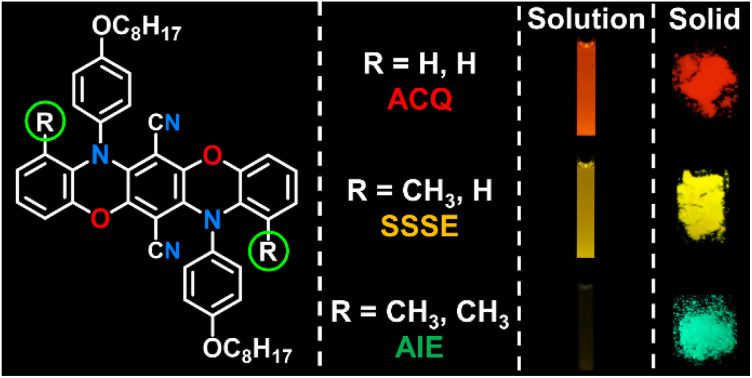

## Introduction

Fluorophore research is an interdisciplinary research area due to its versatile applicability, high sensitivity, high spatial resolution and temporal fingerprint. Specifically, in materials science, there is an ongoing need for novel emitters used in optoelectronic applications such as organic light-emitting diodes (OLEDs),^1^ information encryption,^2^ solar concentrators,^3^ or displays of liquid crystalline (LC) materials.^4^ For instance, synthetic approaches can structurally adjust tetraoxapentacene derivatives^5^ or the commonly used tetraphenylethylene (TPE) motif,^6^ forming luminescent LC phases.^7^ Synthetically less challenging approaches can be realized by the doping of luminophores in the LC host materials.^8,9^

For arrays and devices that require emissive capability in the solid-state, researchers often utilize fluorophores exhibiting aggregation-induced emission (AIE).^10^ Although long known,^11^ this phenomenon experienced a renaissance of interest since its rediscovery in 2001.^12^ Since then, significant efforts have been made to fully understand the systematic requirements to invert the emission profile of isolated molecules exhibiting luminescence in dilute solutions that suffer from aggregation-caused quenching (ACQ).^13,14^ In densely packed structures of systems exhibiting ACQ, π–π stacking most often leads to non-radiative energy dissipation after electronic excitation.^15^ Hence, one possibility to achieve AIE is preventing the detrimental stacking effects by implementing twistable, movable moieties.^16^

Ultimately, it was recognized that these two fluorescence phenomena are not entirely contradictory and can be united to combine their advantages, overcoming their respective limitations and expanding application possibilities.^17,18^ This anomaly of microenvironmentally independent luminescence, *e.g.*, in dilute solution, amorphous powders, or applied materials, is preferably referred to as “solution and solid-state emission” (SSSE). Although often ambiguously declared as “dual-state emission”, it should not be confused with dual-emission processes, *e.g.*, from singlet and triplet states.^19,20^ Several concepts have been postulated for designing highly versatile systems displaying SSSE.^21^ Popular approaches combine planar and rigid scaffolds with stacking-preventing rotors^22^ or incorporate vibrationally twisting moieties.^16^ Terephthalonitrile cores are particularly prominent because various commercially available nucleophiles can be conveniently used to facilely synthesize donor–acceptor structured luminophores.^23,24^ Maly and co-workers reported the spectroscopic properties of a series of novel pentacyclic *N*-arylated heteroacenes, which display pronounced red emission in dilute solutions owing to their restricted conformational mobility but comparably low photoluminescence quantum yields in the amorphous powders.^25^ Recently, we published our findings on cationic bridged oxo- and thioethers showing modulated SSSE properties that allow bioimaging of cells, bacteria, and even protists.^26^ However, we were interested in further exploring the requirements for SSSE, aiming for additional specific functionalization possibilities while maintaining the rigid structure required for pronounced emission in solution. Nitrogen atoms were a logical choice since they offer a third covalent bonding site and can be easily derivatized.^27^

Elegantly altering the photophysical properties involving designated luminophores has been an ongoing research field for the last few years.^28^ Generally, the approaches include changing the substitution pattern,^29^ varying electron-donating and -accepting groups,^30,31^ or sophisticatedly modifying the core aromatic structures by photochemical cyclization.^32^

Recently, the conformational influence of *ortho*-positioned substituents in various systems has gained more attention.^33,34^ Tian's group published intriguing strategies for manipulating the photophysical properties of *N*,*N*′-disubstituted phenazines.^35^ In these systems, aromatic frameworks^36^ or even methyl groups^37^ in the *ortho*-position to the bridging atoms exert substantial steric strain. This pressure induces rehybridization of the central nitrogen atoms from sp^2^ to sp^3^, causing out-of-plane bending of the *N*-substituents. Upon irradiation, the azine rings of the dyes undergo photoinduced planarization, resulting in orange-red emission with large Stokes shifts above 10 000 cm^−1^.^38^ Previously, Bryce and colleagues observed similar effects on *ortho*-methylated phenoxazines, forcing the *N*-substituents to adopt axial instead of the usually preferred equatorial conformations. This conformational change influenced the absorption maxima but did not affect the emission wavelengths.^39^ Additionally, *ortho*-positioned methyl groups significantly impacted the rotation velocities of Feringa's molecular motors.^40^ This universal phenomenon has already been recognized in medicinal chemistry, often referred to as the unpredictable yet versatile “magic methyl” effect.^41^

Hence, in this study, we examined the impact of steric pressure induced by *ortho*-positioned methyl groups on the photophysical properties of *N*,*N*-diaryl diazadioxatetrahydro-pentacene luminophores. Ultimately, these stimuli-responsive molecules were applied as optical sensors of temperature changes and phase transitions in liquid crystals due to their rapid response to microenvironmental changes.

## Results and discussion

### Design and synthesis

**Fig. 1:**
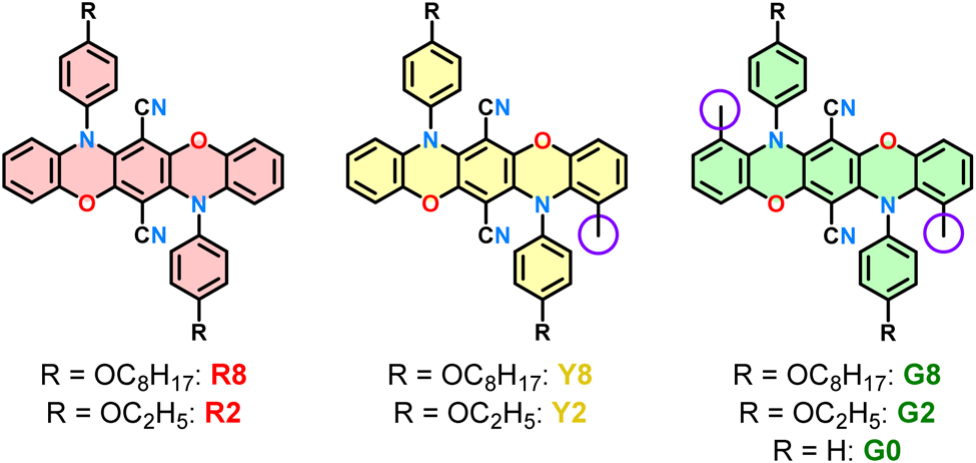
Structural formulae of the target compounds R8/R2, Y8/Y2, and G8/G2/G0.

The design strategy comprises the three heteropentacyclic derivatives R8, Y8, and G8, possessing none, one, or two methyl groups in the 3-position (Fig. 1). Based on the report of Hiscock *et al.*, octyloxy groups in the peripheral positions were chosen to ensure sufficient solubility.^25^ However, the octyl chains induced high disorder in attempted crystallization experiments, resulting in flawed structure models. Therefore, ethyloxy derivatives R2, Y2, and G2 were also synthesized, crystallized, and investigated for their photophysical properties. Unfortunately, crystals of G2 were unmeasurable, mostly due to poor scattering. Thus, G0 was prepared and successfully crystallized. Although hypothetically possible, compounds R0 and Y0 were not synthesized since the literature-known compound R0 failed to crystallize,^25^ and the influence of the ethoxy chains compared to hydrogenated versions on the out-of-plane twisting was expected to be negligible.

The synthesis of all target luminophores was achieved by first Ullmann–Goldberg type copper-catalyzed *N*-arylation of 2-aminophenol or 2-amino-3-methylphenol with the respective iodobenzene derivatives (Scheme S1†).^42^ Subsequently, nucleophilic aromatic substitution reactions (S_N_Ar) with tetrafluoroterephthalonitrile (TFTN) yielded the desired pentacyclic congeners (R8/R2 and G8/G2/G0) as well as the corresponding tricyclic phenoxazine products 4a–4e (Fig. S1†). Asymmetric S_N_Ar reactions of the non-methylated phenoxazines (4a–b) with the methylated aminophenol precursors (3c–e) finally provided the mono-methylated target compounds Y8/Y2. The proposed structures were verified using ^1^H-, ^13^C-, and 2D-NMR spectroscopy, high-resolution mass spectrometry, and IR spectroscopy. The absence of signals in ^19^F-NMR spectra proved full conversions to hexa-substituted compounds. Furthermore, high-performance liquid chromatography (HPLC) was applied to assert high sample purity (>99%), ensuring that the photophysical properties are unaffected by unknown impurities (Fig. S19 and S20†). X-ray diffractometric analyses of single crystals (*vide infra*) ultimately validated the proposed molecular structures, confirming that the *anti*-substituted products were formed instead of the possible *syn*-substituted products.

### X-ray diffractometric analysis

**Fig. 2:**
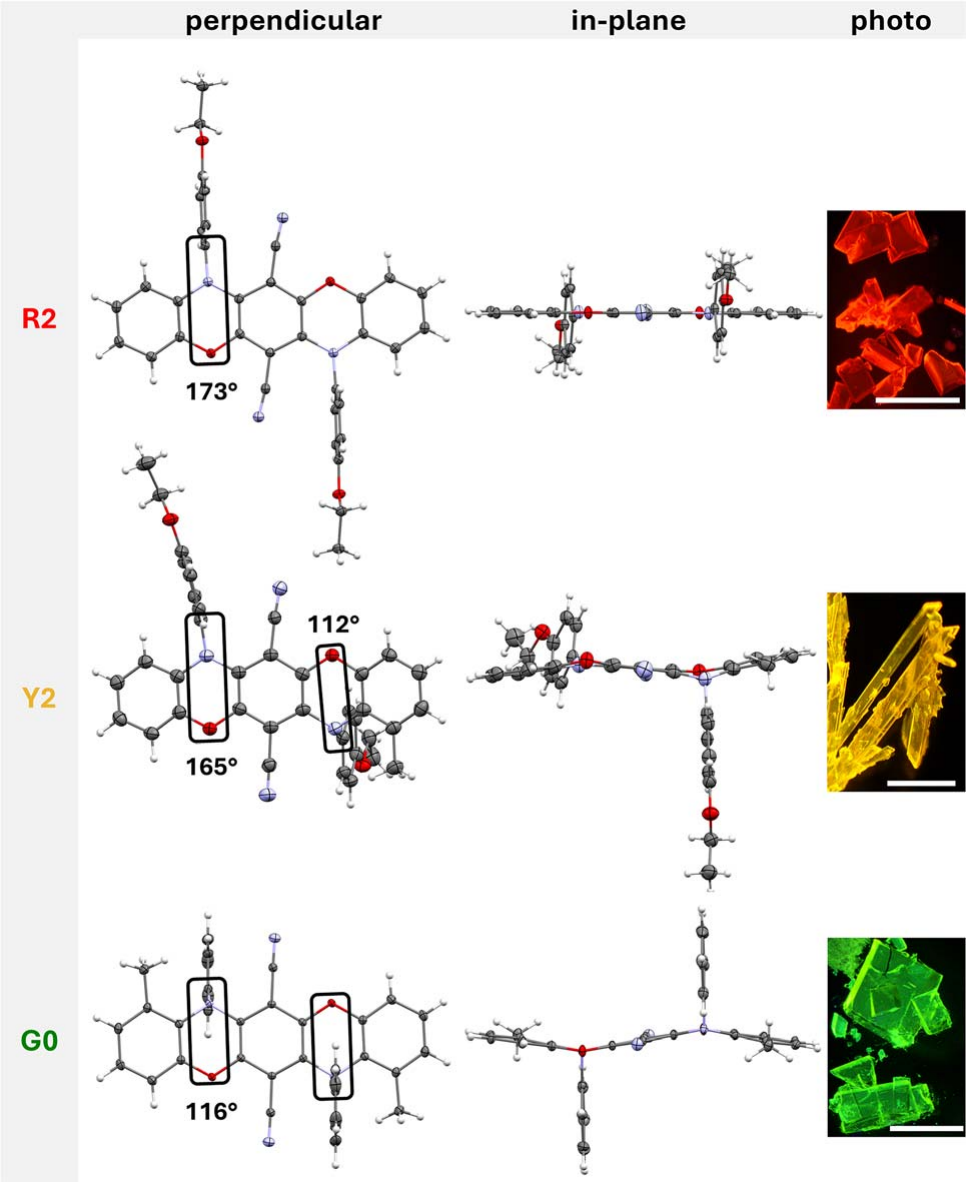
Molecular structures found in crystals of R2, Y2, and G0 with perpendicular (left) and in-plane (central) views of the luminophore unit, microscope images under 365 nm UV light (right) and *α*_ONC_ angle. Displacement ellipsoids are displayed at 50% probability levels. Scale bar: 0.5 mm.

Crystals of R2, Y2, and G0 and phenoxazine precursors 4c and 4d were characterized using X-ray diffractometry (deposition numbers CCDC 2427615–2427617, 2429052 and 2429053 contain the crystallographic data in this study). The crystal packing was analyzed to highlight the structural differences between these compounds resulting from the introduction of the *ortho*-positioned methyl groups on the luminophoric units. Fig. 2 displays the crystal structures of all crystallized compounds with perpendicular and in-plane views of the luminophoric unit and the measured angle *α*_ONC_ of the linearly arranged bridging oxygen and nitrogen atoms with the *ipso*-carbon atom of the aromatic substituent.

Slow evaporation from dichloromethane/cyclohexane yielded red plate-like single crystals of compound R2, which crystallized with one half-molecule in the asymmetric unit, situated on an inversion center in the triclinic crystal system of the *P*1̄ space group. The luminophore unit reveals a planar structure, with equatorial and nearly linear orientation of both *N*-aryl substituents (Σ*N*_CCC_ = 359.3°, *α*_ONC_ = 173°).

**Fig. 3:**
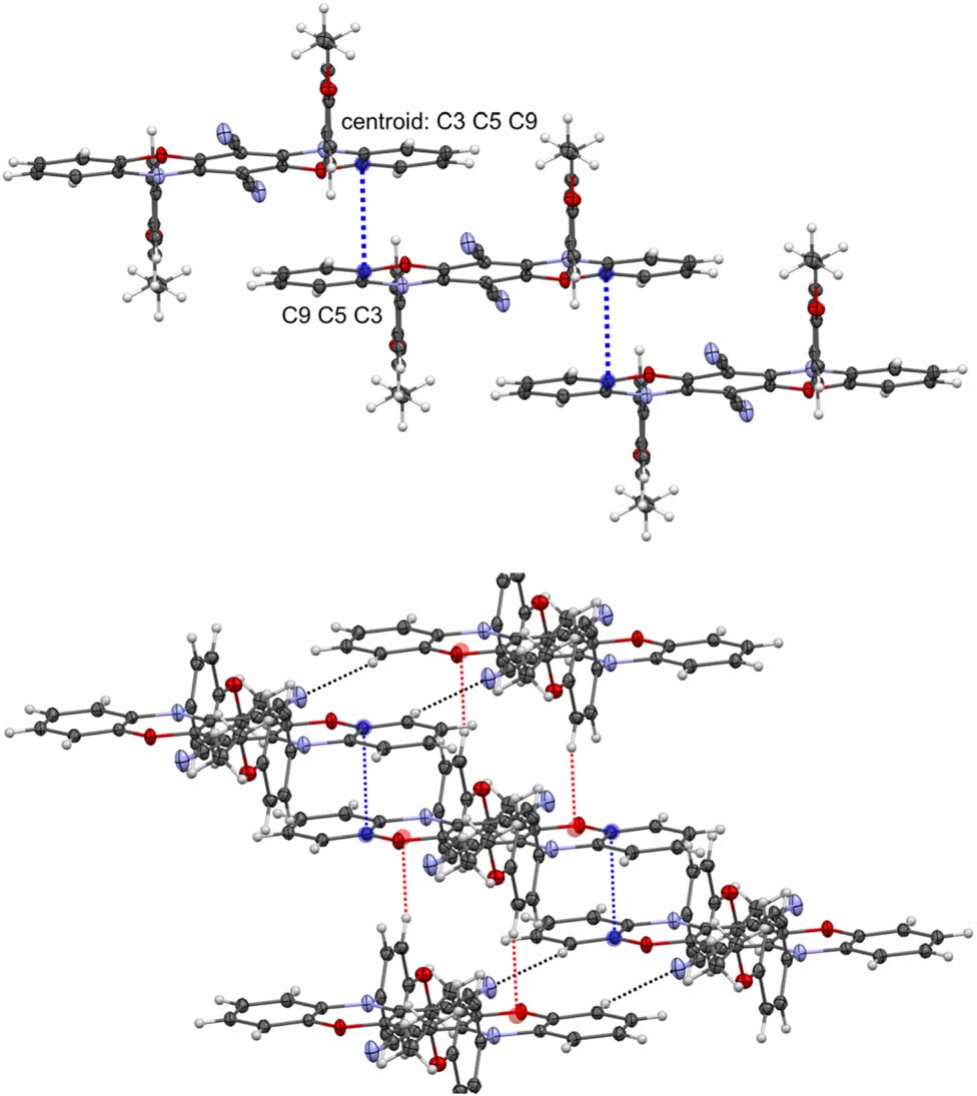
Stair-like chain formation *via* π⋯π interactions between the luminophore units (top) and excerpt of the packing diagram representing all three types of interactions (CH⋯π, π⋯π and CH⋯N) between the R2 molecules (bottom). Displacement ellipsoids are displayed at 50% probability levels. Non-covalent intermolecular interactions: 3.287 Å (blue), 2.488 Å (red), and 2.540 Å (black).

These *N*-aryl substituents adopt a parallel orientation to the neighboring nitrile groups, with a twist angle of 89.9°. The nitrile groups are slightly bent away from the *N*-aryl substituents (C1–C4–N1 173°), and a short distance of 3.310 Å is present between the centroid of the phenyl ring and the nitrogen atom of the CN group, indicating an intramolecular C–N⋯π interaction and implicating the hindered rotation of these *N*-aryl substituents. In the packing diagram of compound R2, the formation of a 3D-network is observed (Fig. 3). The most relevant interactions are the π⋯π contacts (3.287 Å, blue color) between the pentacene units supported by C–H⋯π bridges (2.488 Å, red color) between the *N*-aryl substituent and the adjacent oxazine ring as well as C–H⋯N hydrogen bonds (2.540 Å, black color) involving the luminophore unit and the nitrile groups.

Compound Y2 crystallized as yellow plates in the triclinic crystal system of the *P*1̄ space group. Introducing one methyl-substituent in the *ortho*-position of the luminophore unit significantly impacts the structure. Consequently, the *N*-aryl substituent on the methyl side is twisted out-of-plane due to steric repulsion (*α*_ONC_ = 112°), changing the hybridization of the nitrogen atom from sp^2^ to sp^3^. The sum of the angles around this nitrogen atom is 334.3°. The geometry of the nitrogen atom at the non-methylated unit remains planar, with a sum of angles around the nitrogen atom of 359.3°, although showcasing slightly distorted linearity (*α*_ONC_ = 165°).

**Fig. 4:**
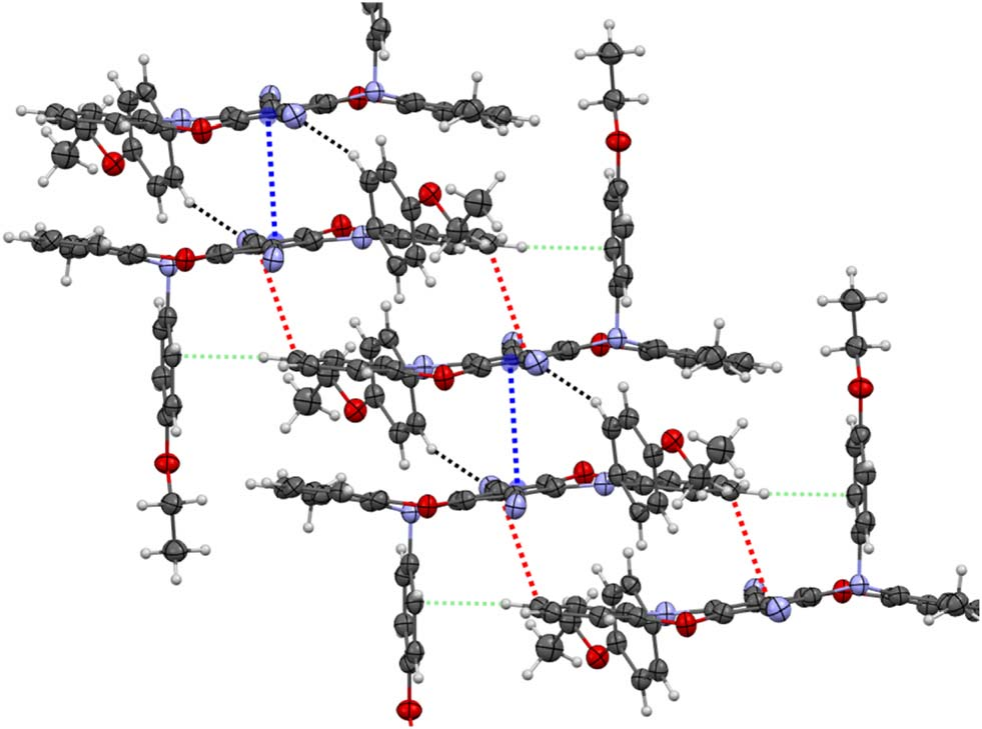
Excerpt of the packing diagram depicting CH⋯π, π⋯π and CH⋯N interactions between the Y2 molecules. Displacement ellipsoids are displayed at 50% probability levels. Non-covalent intermolecular interactions: 3.383 Å (blue); 3.335 Å (red); 2.558 Å (black); 2.594 Å (light green).

As expected, the π⋯π interactions and, implicitly, the overlapping mode of the luminophore units change appreciably, forming a stair-like chain containing alternating dimer-type units. The overlapping of the analogous parts of the luminophore containing the sp^2^ hybridized nitrogen atom (blue color) is very similar to that of compound R2 (the distance between the centroids is 3.383 Å), but with an additional C–H⋯N interaction (2.558 Å, black color) involving the sp^2^*N*-aryl substituent and the nitrile group. For the part of the luminophore unit containing the sp^3^-hybridized nitrogen atom, the overlapping mode is decreased, and the CN groups play a double role in the formation of these dimeric-type units (Fig. 4). These groups are involved in the formation of π⋯π interactions (3.335 Å, red color) with part of the neighboring oxazine ring and support this dimer-type structure by additional C–H⋯π interactions (2.594 Å, light green color) involving the out-of-plane *N*-aryl substituent.

**Fig. 5:**
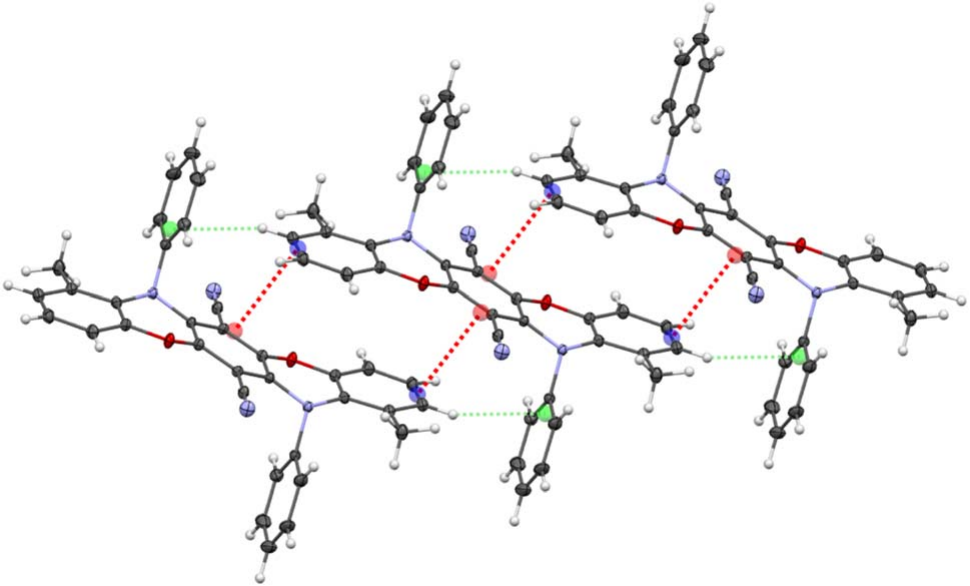
Stair-like chain formation *via* π⋯π interactions (red color) supported by C–H⋯π interactions (light green color). Displacement ellipsoids are displayed at 50% probability levels.

Compound G0 crystallized as green emissive plates with one half-molecule in the asymmetric unit, situated on an inversion center in the triclinic crystal system of the *P*1̄ space group. Due to the second *ortho*-positioned methyl group, both nitrogen atoms in the oxazine moieties exhibit a distorted tetrahedral geometry (sp^3^ hybridization) with both *N*-substituents oriented out-of-plane. A slight deviation from the planarity of the entire luminophore unit is observed (S-like shape motif, Fig. 5). The stair-like chain observed in the packing diagram reveals the formation of additional C–H⋯π interactions (2.594 Å, light green color) involving the out-of-plane *N*-aryl substituents. The nitrile groups interact with two phenyl groups of the neighboring luminophore units *via* C–H⋯N interactions and with an adjacent CN group *via* π⋯π interaction (see Fig. S90–S93†).

The selected interactions of the crystal analysis agree well with the Hirshfeld surface analyses using CrystalExplorer17 (see Fig. S100–S102†).^43^R2 exhibits primarily π⋯π interactions involving the pentacene moiety, accompanied by hydrogen bonding, whereas the incorporated methyl groups on the oxazine rings shift the binding sites to the nitrile groups and the *N*-aryl rings for Y2 and G0. Hence, the *ortho*-methyl groups induce stronger steric repulsion with the *N*-substituents and are consequently responsible for the twisting of the *N*-substituents out of the plane. As a result, the number of C–H⋯π interactions gradually increases, while the π⋯π interaction between the identical heteropentacene units reduces.

### Quantum chemical calculations

Gaussian 16 was used for the quantum chemical calculations.^44^ Ground-state (S_0_) geometry optimizations were conducted using density functional theory (DFT) with Grimme's dispersion correction (D3BJ);^45^ for the excited singlet states S_1_, time-dependent density functional theory (TD-DFT) was applied.^46^ The calculations were performed in the gas phase employing the functional PBE0 (ref. 47) with the def2-TZVP^48^ basis set. For all initial input geometries, the obtained crystal structure geometries were used. The absence of imaginary frequencies verified the acquired structures as stationary points.

Generally, the geometries from X-ray diffractometry and the computed structures obtained from optimized parameters are in good agreement. To evaluate the energetic stabilization associated with the out-of-plane bending in Y2 and G2, the energies were compared with those when assuming planar initial geometries similar to R2. The twisting in Y2 (*α*_ONC_ = 116°) stabilizes the structure by an energetic difference of 5.4 kcal mol^−1^ compared to the planar isomer (*α*_ONC_ = 176°). For G2 (*α*_ONC_ = 118°), the energetic stabilization increases to 9.9 kcal mol^−1^ compared to the planar isomer (*α*_ONC_ = 169°).

Further indication of the aromaticity change of the oxazine ring upon methyl-induced rehybridization from sp^2^ to sp^3^ was accomplished by computing values for nucleus-independent chemical shifts (NICS) at *z*-directed distances of 1 Å from the ring centroids.^49^ The calculated NICS(1) value of benzene at the same level of theory (GIAO-PBE0/def2-TZVP) corresponds to the literature value of −10.0 ppm, denoting aromaticity.^50^ The computed NICS(1) value for R2 indicates an anti-aromatic (6.4 ppm) and for G2 an expected non-aromatic character (0.3 ppm). Comparable NICS(1) indices were computed for Y2, yielding −0.1 ppm for the methylated side and 6.7 ppm for the non-methylated side.

**Fig. 6:**
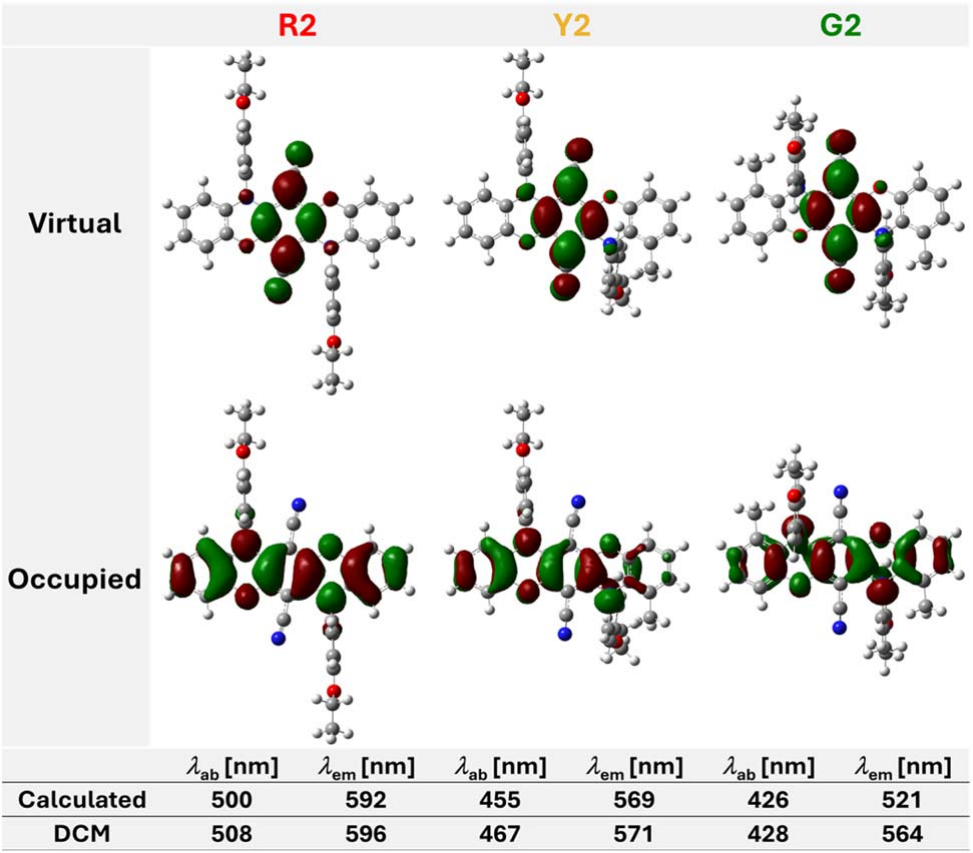
Top: calculated optimized geometries in the S_0_ states and occupied and virtual NTO pairs. Bottom: calculated absorption and emission wavelengths and experimental data obtained from DCM solutions. Calculations were performed in the gas phase using PBE0-D3BJ/def2-TZVP for S_0_ states and TD-PBE0/def2-TZVP for S_1_ states.

For all three compounds, the nature of the lowest excited singlet states is primarily characterized by monoelectronic (n + π)–π* excitations from the highest occupied molecular orbitals (HOMOs) to the lowest unoccupied molecular orbitals (LUMOs). In this context, the HOMOs are distributed at the electron-rich oxazine moieties. In contrast, the LUMOs are localized at the vertical axis of the electron-withdrawing nitrile groups. This suggests that intramolecular charge-transfer processes occur upon photoexcitation, which can be visualized using electron density difference calculations (Fig. S103–S105†). The electronic excitations are qualitatively visualized by natural transition orbital (NTO) pairs (Fig. 6). In contrast to R2, the occupied NTOs of Y2 and G2 showcase reduced contribution from the π-systems at the methylated side due to the weakened conjugation caused by the non-aromaticity of the oxazine moiety. Consequently, the HOMO–LUMO energy gap gradually increases from R2 to G2 (see Table S12†) in accordance with the hypsochromic shift of the maximum absorption wavelength (*vide infra*). Additionally, theoretical absorption and emission wavelengths were calculated and compared to the measured values (*vide infra*). All simulated values agree well with the experimental data obtained from DCM solutions, except for the emission wavelength *λ*_em_ of G2 (521 nm calculated *vs.* 564 nm measured). This can be explained by the nature of gas-phase calculations, where, *e.g.*, solvent-specific interactions or facilitated relaxation processes leading to higher Stokes shifts are not considered. Hence, this highlights the predictability of the presented systems, as the accurate calculation of matching molecular properties is often a major challenge when designing new luminophores.

The anticipated negligible impact of ethoxy chains *versus* the hydrogenated compounds G0 and hypothetical R0 and Y0 was confirmed by performing comparable geometry optimizations and UV calculations, proving similar bent geometries of G2 and G0.

### Investigation of the photophysical properties

The photophysical properties of the compounds were investigated using solid-state samples, solutions in dichloromethane (DCM), and mixtures of tetrahydrofuran (THF) and water, since apolar solvents such as toluene or polar solvents such as acetonitrile displayed limited solubilization capability. First, UV/vis spectra were recorded in DCM and THF (Fig. S21 and S22†). Although the absorbances decrease slightly when changing the terminal chains from octyl to ethyl, the maximum absorption wavelengths *λ*_ab_ remain identical, as the terminal chains do not affect the aromatic core structure. However, *λ*_ab_ values substantially decrease by approximately 40 nm per methyl group (Table 1). Consequently, R8 appears reddish-orange in solution, Y8 yellowish, and G8 faintly yellowish. These findings are in accordance with the previous section. The degree of sp^2^-hybridization of the bridging nitrogen atom and the aromaticity changes of the oxazine cores strongly affect the HOMO energy, resulting in an increased energy required for a monoelectronic excitation when progressing from R8 to G8.

**Table 1:** Overview of selected photophysical properties in DCM (10 μM) and the solid-state: wavelengths *λ* [nm] for absorption *λ*_ab_, excitation *λ*_ex_, and emission *λ*_em_, absolute photoluminescence quantum yield *Φ*_PL_, and amplitude-weighted average fluorescence lifetime *τ*_av_amp_ [ns]

		R8	R2	Y8	Y2	G8	G2
DCM	*λ*_ab_ [nm]	508	508	467	468	428	428
	*λ*_em_ [nm]	597	596	571	571	564	563
	*Φ*_PL_	0.52 ± 0.03	0.53 ± 0.03	0.14 ± 0.02	0.17 ± 0.02	<0.01	<0.01
	*τ*_av_amp_ [ns]	10.66 ± 0.02	10.65 ± 0.02	3.11 ± 0.01	3.42 ± 0.01	n.d.	n.d.
Powder	*λ*_ex_ [nm]	574	587	544	523	473	483
	*λ*_em_ [nm]	610	622	571	578	499	507
	*Φ*_PL_	0.22 ± 0.02	0.04 ± 0.02	0.26 ± 0.02	0.23 ± 0.02	0.40 ± 0.02	0.47 ± 0.02
	*τ*_av_amp_ [ns]	6.55 ± 0.08	1.31 ± 0.04	8.72 ± 0.05	7.18 ± 0.07	6.96 ± 0.04	6.84 ± 0.08

**Fig. 7:**
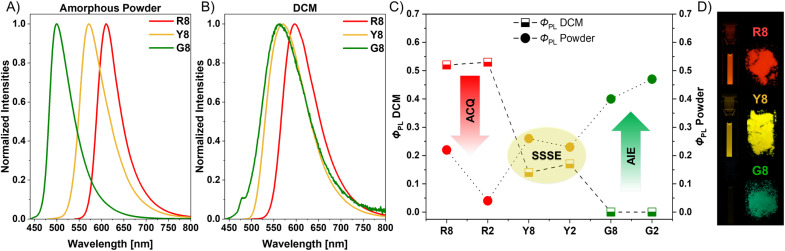
Steady-state photoluminescence spectra of the octyl-substituted compounds in (A) amorphous powders (R8: *λ*_ex_ = 520 nm; Y8: *λ*_ex_ = 440 nm; G8: *λ*_ex_ = 420 nm) and (B) DCM solutions (10 μM; R8: *λ*_ex_ = 480 nm; Y8: *λ*_ex_ = 460 nm; G8: *λ*_ex_ = 420 nm). (C) Plot of the absolute photoluminescence quantum yields for R8/R2, Y8/Y2, and G8/G2 in DCM and as amorphous powders. (D) Respective photographs of the DCM solutions (10 μM, 365 nm UV light) and amorphous powders (395 nm).

Steady-state and time-resolved photoluminescence spectroscopy were utilized to assess the emission behavior of the compounds (Fig. 7 and Table S1†). Similarly, no significant differences between octyl- and ethyl-substituted compounds are observable in solution. A strong reddish-orange emission can be detected for R8 at nearly *λ*_em_ = 600 nm. Accordingly, Y8 shows a yellowish emission at *λ*_em_ = 571 nm, whereas only a faint, weak yellow luminescence can be observed for G8. This can be ascribed to motion-induced fluorescence quenching because non-radiative deactivation pathways are favored, resulting from the free rotation of the *N*-aryl rings in G8. These rotations are restricted in R8 due to the confined space induced by the nitrile groups, which stabilize the system by intramolecular C–N⋯π interactions with the substituent ring. For all compounds, fluorescence from an excited singlet state is the main emission mechanism since all measured lifetimes are in the nanosecond range (up to 11 ns). Moreover, the Stokes shifts steadily increase from R8 to G8, indicating motion enhancement in the excited states through out-of-plane twisting and unrestricted rotation of the *N*-aryl substituents.

In contrast to the solutions, all compounds show strong emission signals in the solid-state, with matching color impressions (red for R8, yellow for Y8, and green for G8). Furthermore, all emission wavelengths of the ethyl compounds are somewhat bathochromically shifted compared to those of the octyl-substituted compounds. This is because the terminal chains influence the packing behavior, leading to more hydrophobic environments and distinctive packing effects (Fig. S27†).^51^ Absolute photoluminescence quantum yields (*Φ*_PL_) were determined to classify the investigated compounds (Fig. 7 and Table 1). When comparing the values in DCM with those from the amorphous powders, R8 displays a substantial decrease in the solid-state, whereas G8 shows a significant emission turn-on as an amorphous powder. For Y8, however, the values are similar in both the solution and the solid-state. These effects are even more pronounced for the respective ethyl compounds, which are less effective in averting the stacking of the core luminophores. The packing analyses (*vide supra*) revealed strong π⋯π interactions for R2 between identical luminophore units, which are significantly reduced for G2. This π⋯π stacking is also responsible for the low solid-state *Φ*_PL_ value of R2 (0.04) compared with R8 (0.22), as it is known that longer alkyl chains can sufficiently suppress detrimental π⋯π interactions through self-isolation of the chromophores.^52^ Also, R8 and R2 show comparable optical properties with the previously mentioned congeners reported by Maly *et al.*^27^ A more detailed tabular comparison of the optical properties with literature-known dyes is depicted in the ESI (Table S13†).

These results demonstrate that achieving the desired emission characteristics in both solution and the solid-state requires a delicate balance. In this case, introducing two methyl groups reverses the fluorescence behavior from ACQ to AIE; however, a single methyl group maintains both characteristics by sufficiently preventing packing-caused quenching on one half of the molecule and retaining substantial emission in solution on the other half.

The response of the octyl-substituted compounds to aggregation was investigated using binary mixtures of THF with an increasing amount of water (Fig. S28†). Compared to the DCM solutions, the emission wavelengths in pure THF shift hypsochromically, yielding yellow emission (*λ*_em_ = 578 nm) for R8 and lime-colored emission (*λ*_em_ = 553 nm) for Y8. Upon adding water, the relative emission intensity decreases, accompanied by a bathochromic emission shift resulting from the enhanced polarity in the presence of water.^14^ Aggregates were first formed at 40/60 THF/water content for R8 and Y8, whereas for G8, aggregation already occurs at 60/40 THF/water content. This is due to the lower dipole moment of G8, which affects the polarizability and solubility.

As expected for chromophores displaying AIE, the aggregation process of G8 induces an emission turn-on due to the restriction of intramolecular motion (RIM). Similarly, Y8 exhibits an increase in relative emission intensity, exceeding the initial value in pure THF. At higher water contents, the emission intensities of Y8 and G8 remain substantial, although slightly decreasing compared to that of their respective 40/60 THF/water mixtures. In contrast, the ACQ effect of R8 leads to a concomitant decrease in relative emission intensity with higher water content. The observed phenomena correlate with the *Φ*_PL_ values, measured at 0%, 60%, and 99% water contents (Table S3†).

### Mesoporous silica nanoparticles for additive manufacturing

After the detailed analysis of the photophysical properties, material applications of these compounds were investigated. According to our previous studies, we aimed to utilize the octyl-substituted compounds as fluorescent additives for 3D-printed materials using digital light processing (DLP).^29^ Initially, 0.1 wt% of the compounds were attempted to be dissolved in a resin containing 69 wt% of monomer 2-[[(butyl amino)carbonyl]-oxy]ethyl acrylate (see the ESI† for further details). However, even 0.005 wt% of compounds failed to dissolve homogeneously within the resin. Although adding solutions of the compounds dissolved in DCM to the resins and evaporating afterwards seemingly improved the solubilizing process, the 3D-printed elastomeric discs (*Ø* 1 cm) revealed inhomogeneous distributions of the dyes after photopolymerization (405 nm, Fig. S31†).

Therefore, as previously reported, mesoporous silica nanoparticles (MSNs) were prepared *via* a modified Stöber synthesis (see the ESI† for details).^53^ THF solutions of the octyl-substituted compounds were used in the synthesis, leading to the incorporation of the luminophores into the MSNs. Photometric analysis yielded mass fractions of 3–6 μg compound per mg MSN. The average particle diameters range between 70 and 110 nm, as determined with scanning electron microscopy (SEM) images (Fig. S56–S60†).

**Fig. 8:**
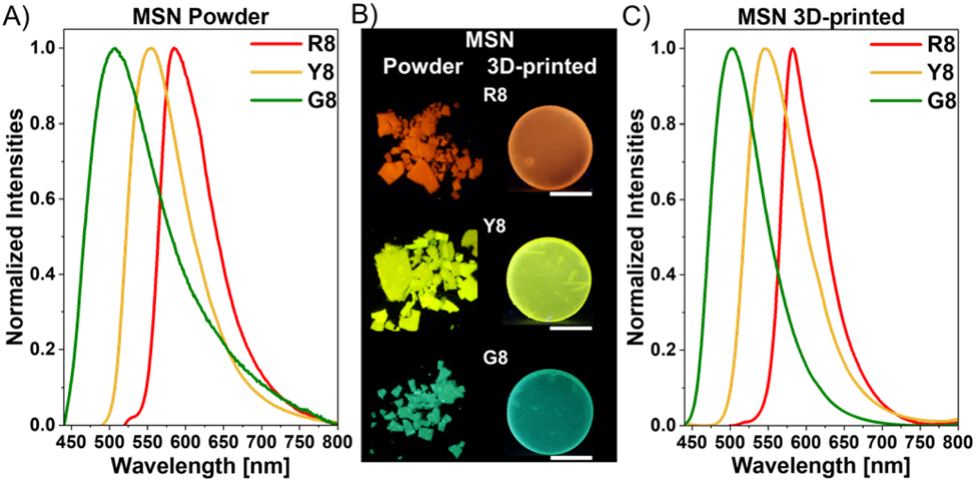
Normalized emission spectra of Mesoporous Silica Nanoparticles (MSNs) loaded with the octyl-substituted compounds (A) as a bulk powder (R8: *λ*_ex_ = 510 nm, Y8: *λ*_ex_ = 460 nm, and G8: *λ*_ex_ = 420 nm) and (C) in 3D-printed discs (R8: *λ*_ex_ = 480 nm, Y8: *λ*_ex_ = 420 nm, and G8: *λ*_ex_ = 420 nm); (B) corresponding images taken under 365 nm UV light. Scale bar: 5 mm.

3 wt% of these luminescent MSNs were conveniently dispersed in the resins and photopolymerized, yielding homogeneous distributions of the particles within the 3D-printed objects (Fig. S30†). Fig. 8 displays the steady-state emission spectra of the loaded MSNs as both bulk powders and the 3D-printed objects. As anticipated, the compounds display similar photophysical properties in both states (MSN powders and 3D-printed materials) because the outer silica spheres effectively shield the core luminophores from the influence of the external environments (Table S4†).

### Fluorescence in liquid crystalline materials

The compounds were embedded as single compounds in liquid crystalline (LC) host materials to further explore the order-dependent fluorescence of the systems in response to changing the mesogenic phases. E7, a mixture of four different cyanobiphenyls (see the ESI†), was chosen as a commercially available host to rationalize the response to temperature within the same phase, as E7 exhibits a broad nematic phase up to ∼60 °C. Additionally, 4′-octyl-4-cyanobiphenyl (8CB) was examined due to showcasing three phase transitions (crystalline (Cr) → smectic (Sm) → nematic (N) → isotropic (Iso)) over a relatively small temperature range of less than 20 °C, which helps minimize temperature effects. Both systems are based on cyanobiphenyls and are anticipated to show a minimum degree of solvatochromism between the hosts.

**Fig. 9:**
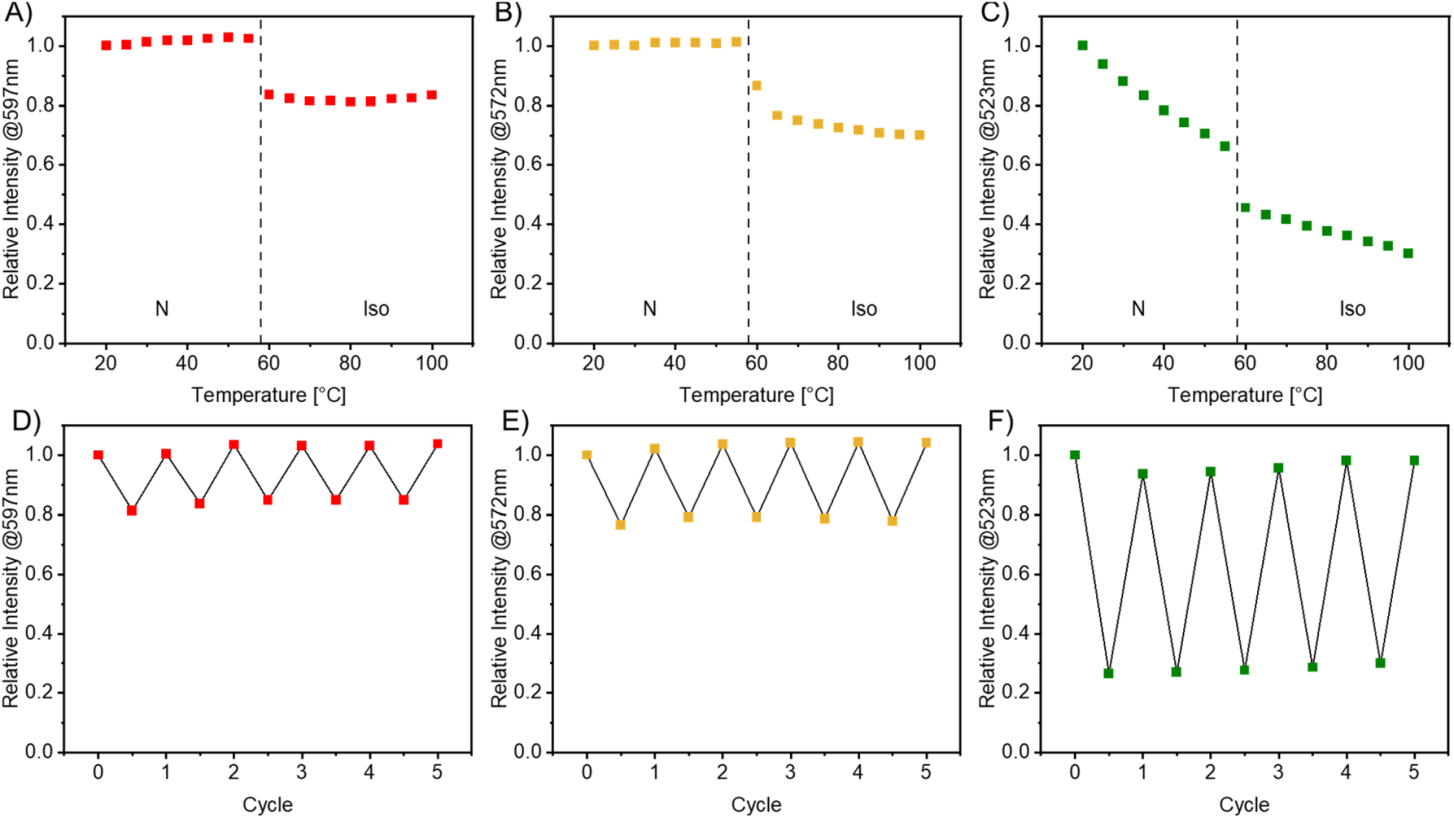
Plots of the relative photoluminescence intensities at the emission maximum *versus* the temperature in the second cooling cycle (top) or *versus* the number of cycles (bottom) for R8 (A, D), Y8 (B, E), and G8 (C, F) in E7 (0.1 mol%). The intensity of the spectrum at 20 °C was normalized to 1. The dotted line marks the transition temperature between the nematic (N) and isotropic phase (Iso) according to DSC measurements (peak temperature).

To commence, 0.1 mol% of each octyl emitter (R8, Y8, or G8) was mixed into E7 by preparing stock solutions in DCM and evaporating the solvent afterwards. Differential scanning calorimetry (DSC) measurements, conducted at a cooling rate of 10 °C min^−1^, confirmed that this low amount of emitter affects the liquid crystalline behavior of the host insignificantly, shifting the N → Iso phase transition from 59 °C to approximately 58 °C (peak temperature, Fig. S69–S71†). Polyimide-coated cells were filled with the samples and positioned on a heating stage within a benchtop fluorescence spectrometer setup (see the ESI† for details). After an initial heating and cooling cycle, fluorescence spectra were recorded at various temperatures in 5 °C steps from 20–100 °C. An equilibration time of 30 s was selected to ensure a stable thermodynamic phase prior to each measurement. Fig. 9 shows the plots of the relative intensity at the emission maximum *versus* the temperature in the second cooling cycle for each emitter. The intensity of the spectrum at 20 °C was normalized to 1.

As expected, the three luminophores exhibit vastly different fluorescence behaviors in the LC host. The intensity of R8 in the same phase of E7 remains consistent within the margin of experimental uncertainty. However, the intensity decreases to 82% in the isotropic phase compared to the nematic phase. We attribute these observations to the aggregation tendency of R8. With increased temperature, non-emissive pathways through movement are more accessible, leading to a decrease in relative emission intensity. Conversely, reducing order in the LC enhances the ACQ effect by amplifying the relative emission, compensating for the previous effect. A phase transition entirely changes the molecular environment and, thus, the temperature-independent emission intensity. Y8 behaves quite similarly, with a slight decrease in intensity in the isotropic phase between 65 and 100 °C from 77 to 70%. In contrast, G8 shows a substantial temperature dependency. Even within the same phase, the intensity decreases linearly with increasing temperature. In the nematic phase, the emission intensity decreases to 66% at 55 °C compared to 20 °C. In the isotropic phase, it further decreases from 46% to 30% from 60 °C to 100 °C, respectively. These findings support our previous assumption that G8 acts as an aggregation-induced emitter. The increase in temperature and its subsequent effect on the order of the liquid crystal promote non-emissive pathways and explain the observed effects.

In conclusion, all emitters display their highest relative emission intensity at 20 °C, with a noticeable decrease in intensity upon phase transition to the isotropic phase. R8 is temperature-independent, while Y8 exhibits a weak and G8 a distinct temperature-dependency, agreeing well with ACQ, SSSE, and AIE characteristics.

To investigate the thermal stability of the compounds and the photobleaching effects due to relatively high excitation intensity, four additional heating and cooling cycles were conducted, and a spectrum was obtained at 20 and 100 °C each time. The results, compared to the initially observed spectrum, are shown in Fig. 9 and demonstrate the absence of a photobleaching effect in the liquid crystal films, indicating that the heating and cooling process can be reiterated over multiple cycles. Additionally, the luminophores remain within the LC host since a change in the start and end intensities would be expected if aggregates were formed. This was also confirmed using polarized optical microscopy (POM) images (Fig. S75–S80†).

**Fig. 10:**
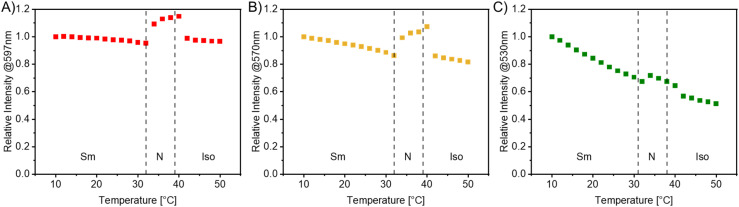
Plots of the relative photoluminescence intensities at the emission maximum *versus* the temperature in the second cooling cycle for emitter R8 (A), Y8 (B), and G8 (C) in 8CB (0.05 mol%). The intensity of the spectrum at 20 °C was normalized to 1. The dotted lines mark the transition temperatures between the smectic (Sm), nematic (N) and isotropic phases (Iso) according to DSC measurements.

Compared to the large intensity gap in E7, the analogous effect was investigated by employing the narrow temperature range phases of the 8CB host. 0.05 mol% of R8, Y8, and G8 were incorporated into 8CB *via* a stock solution approach in DCM. Surprisingly, this low amount of emitter suppressed crystallization, which was first observed by the naked eye and confirmed through DSC measurements (Fig. S72–S74†). Temperature-dependent fluorescence experiments were conducted, as explained above. A temperature interval from 10 to 50 °C in 2 °C steps was selected for these compositions to account for the distinguished transition temperatures. The relative intensity at the emission maximum was plotted *versus* the temperature in Fig. 10. The dotted lines represent the transition temperatures according to the DSC measurements (10 °C min^−1^, peak temperature). In this instance, minor discrepancies between the transition temperatures and points of emission intensity increase and decrease are present. We attribute these differences to variations in the cooling rates in the experimental setup (waiting for the thermodynamic equilibrium *versus* 10 °C min^−1^ in the DSC) and the divergence of thin films and bulk material. Nonetheless, the liquid crystalline phases can be differentiated effectively with the corresponding fluorescence spectra.

R8 in 8CB shows a constantly high relative emission intensity in the smectic and the isotropic phases with roughly the same intensity. The intensity is 10–15% higher in the nematic phase and slightly increased at elevated temperatures, which we attribute to the balanced attractive and repulsive forces in the nematic phase providing an environment of fluidity and orientational order. Specifically, there appears to be a unique interaction based on the molecular structure, leading to an increase in relative emission intensity in the nematic phase.

In 8CB, emitter Y8 exhibits a notably greater temperature dependency than in E7. However, the minimum intensity remains above 80%, comparable to that in E7, resulting in a smaller gap between the LC phases. G8 again demonstrates the most substantial temperature dependency and a minor yet noticeable intensity increase in the nematic phase, as anticipated for aggregation-induced emitters. Additional heating and cooling cycles (Fig. S65–S67†) confirmed the reproducibility and absence of photoinduced decay. For this series of mixtures, the increase in emission within the nematic phase remains the most surprising feature.

To conclude, incorporating R8, Y8, and G8 into the LC hosts E7 and 8CB yielded luminescent liquid crystalline materials, which were investigated towards their temperature and phase dependency. These experiments provided insights into the complex and unpredictable aggregation effects within the LCs. Noteworthily, the phases were easily distinguished in all examples through their fluorescence spectra. This allows new possibilities for designing multi-responsive materials.

## Conclusions

In conclusion, a new concept for achieving efficient solution and solid-state emission has been introduced. Implementing the “magic-methyl” effect in a series of diazadioxatetrahydro-pentacyclic luminophores results in significant exertion of steric strain, twisting the *N*-substituent out-of-plane due to repulsion. Extensive experimental studies substantiated by quantum chemical calculations disclosed an attenuated conjugation in the core, reducing the electron-donating ability and hypsochromically shifting the absorption and emission wavelengths. X-ray diffractometry was employed to validate the structural compositions; the elucidated packing analysis unveiled that C–N⋯π interactions impede rotational quenching, whereas reduced π–π interactions are required to promote efficient solid-state emission. Consequently, precise fine-tuning of the emission colors from red over yellow to green and specifying the preferred environment-dependent emissive state were enabled. Finally, the sensitivity effects have been utilized by materials applications, demonstrating the ability to sense nematic liquid crystalline phases by using fluorescence enhancements. This study is anticipated to extend the conceptual spectrum of novel pathways to design fluorophores exhibiting solution and solid-state emission and their application in sensing and novel optoelectronic technologies.

## Author contributions

A. Huber: conceptualization, data curation, formal analysis, investigation, methodology, visualization, writing – original draft, writing – review and editing; T. Thiele: conceptualization, data curation, formal analysis, investigation, methodology, visualization, writing – original draft, writing – review and editing; T. Rex: investigation, formal analysis; C. Daniliuc: data curation, writing – original draft; C. Wölper: data curation; R. Lorberg: formal analysis, methodology; L. Höfmann: formal analysis, methodology; C. A. Strassert: resources, writing – review & editing; M. Giese: supervision, resources, writing – review & editing; J. Voskuhl: conceptualization, resources, supervision, writing – review & editing.

## Conflicts of interest

There are no conflicts to declare.

## Supplementary Material

SC-OLF-D5SC03028E-s001

SC-OLF-D5SC03028E-s002

## Data Availability

The data that support the findings of this study are available from the corresponding authors upon reasonable request.
