# Supplementary material for: Steric pressure in heteropentacenes modulates the photophysical properties – a molecular design strategy for functional materials
Source: Chem Sci. 2025 Jul 15;16(34):15723–33. doi: 10.1039/d5sc03028e (PMC12326327; doi:10.1039/d5sc03028e)
Supplement: SC-016-D5SC03028E-s001 [file SC-016-D5SC03028E-s001.pdf]

Supplementary Information (SI) for Chemical Science.
This journal is © The Royal Society of Chemistry 2025

Supporting Information

Steric Pressure in Heteropentacenes Modulates the Photophysical Properties – A Molecular Design Strategy for Functional Materials

Alexander Huber[a]+, Tobias Thiele[b]+, Tobias Rex[c], Constantin Daniliuc[d], Christoph Wölper[e], Rick Y. Lorberg[a], Lea Höfmann[a], Cristian A. Strassert[c], Michael Giese[b]*, Jens Voskuhl[a]*

[a] M. Sc. A. Huber, M. Sc. R. Y. Lorberg, M. Sc. L. Höfmann, Prof. Dr. J. Voskuhl, Faculty of Chemistry (Organic Chemistry), CENIDE and Center of Medical Biotechnology (ZMB), University of Duisburg-Essen, Universitätsstraße 7, 45141 Essen, Germany. [+] Both authors contributed equally. * Corresponding authors, e-mail: jens.voskuhl@uni-due.de.

[b] M. Sc. T. Thiele, Prof. Dr. M. Giese, Faculty of Chemistry (Organic Chemistry), CENIDE and Co-Creationlab for Product Innovations (CCLP), University of Duisburg-Essen, Universitätsstraße 7, 45141 Essen, Germany. E-mail: michael.giese@uni-due.de.

[c] M. Sc. T. Rex, Prof. Dr. C. A. Strassert, Institut für Anorganische und Analytische Chemie, CeNTech, CiMIC, SoN, Universität Münster, Heisenbergstraße 11, 48149 Münster (Germany).

[d] Dr. C. G. Daniliuc, Organisch-Chemisches Institut, Universität Münster, Corrensstraße 40, 48149 Münster (Germany).

[e] Dr. C. Wölper, Faculty of Chemistry (Inorganic Chemistry), University of Duisburg-Essen, Universitätsstraße 7, 45141 Essen, Germany.

CONTENT

1 General information and concept ... 3

2 Synthetic procedures ... 5

General procedures ... 5

General procedure (GP1): Copper-Catalyzed Ullmann-Goldberg Type *N*-Arylation of Aminophenols ... 5

General procedure (GP2): Nucleophilic Aromatic Substitutions (S_NAr) ... 5

Overview of performed syntheses ... 5

Overview of synthesized compounds ... 6

Synthetic procedures ... 7

NMR spectra ... 17

HPLC chromatograms ... 34

3 Photophysical properties ... 36

UV/Vis absorption spectra ... 36

Photoluminescence in DCM ... 37

Photoluminescence in powders ... 38

Aggregation series ... 40

Photoluminescence in mesoporous silica nanoparticles (MSN) ... 41

Lifetime reports ... 43

4 Scanning electron microscopy (SEM) ... 55

5 Liquid Crystal (LC) Section ... 57

LC experiments ... 57

Differential Scanning Calorimetry (DSC) ... 60

Polarized Optical Microscopy (POM) ... 62

6 X-ray diffractometric analysis on single crystals ... 64

Crystal structure of R2 ... 66

Crystal structure of Y2 ... 68

Crystal structure of G0 ... 71

Crystal structure of 4c ... 73

Crystal structure of 4d ... 74

7 CrystalExplorer ... 77

8 Quantum chemical calculations ... 81

Computational details ... 81

Cartesian coordinates of the optimized geometries ... 81

Natural Transition Orbitals and Electron density differences ... 89

9 Comparison with literature-known luminophores ... 91

10 Literature ... 93

1 GENERAL INFORMATION AND CONCEPT

Chemicals and synthesis

Commercially available chemicals were purchased from Deutero, TCI, Sigma Aldrich, abcr, Acros Organics, and Fisher Scientific and used without further purification. Potassium carbonate and potassium triphosphate were dried at 90 °C. *n*-Pentane used for chromatography was of technical grade and distilled before use. MilliQ water was obtained through purification by *MicroPure ultrapure*-System from TKA. Sensitive reactions were performed under an argon atmosphere using dried solvents and flame-dried glassware. Sonication steps were conducted using *Sonorex SUPER RK 514 BH* from Bandelin Electronics. The final compounds were freeze-dried using the *ALPHA 1-2* from Christ. For this, dispersions of the compounds in distilled water were frozen in liquid nitrogen under rotation.

Chromatography

Reaction monitoring was performed by thin-layer chromatography (TLC) using *POLYGRAM® SIL G/UV254* plates (0.2 mm) from *Macherey-Nagel*. Spots were visualized by a UV-handlamp (254 nm, 365 nm and 395 nm) by *Herolab* or *AHOME*. Flash-column chromatographic separations were carried out under argon on silica gel *MN 60 M* (40–63 µm) from *Macherey-Nagel*. For this, the crude materials were dry-mounted on *Celite® 545* from *Sigma Aldrich*. The purity of the target compounds was determined with analytical high-performance liquid chromatography (HPLC) using a *NUCLEODUR 100-5* normal phase column (inner diameter 4.6 mm, length 250 mm, silica gel, particle size 5 µm) by *Macherey Nagel*. The setup was used as mentioned before[1] and consists of a *Gastorr* AG-32 degasser, a binary *Waters 1525 pump* with a flow of 1 mL/min, a *Waters 717plus autosampler* and a *Waters 2487 UV/Vis detector* (270 nm). Chromatograms were analyzed using the software *Breeze* (3.20) by *Waters*. HPLC-grade *n*-hexane and ethyl acetate were used with the following gradient run: *n*-hexane/ethyl acetate 90/10 → 0/100 over 30 min, holding 0/100 for 5 minutes. The samples were dissolved in DCM (1 mM) and filtered using a syringe filter (0.22 µM, PTFE).

General analytical methods

NMR spectra were recorded at room temperature using the *AVNEO400* or *AVANCE III* (^{1}H: 400 MHz or 600 MHz, ^{13}C: 101 MHz or 151 MHz, ^{19}F: 376 MHz or 565 MHz) spectrometer from *Bruker* and analyzed with *MestReNova* v15.01-35756. Analytical data are given with the respective frequency, solvent, temperature, chemical shift δ [ppm], multiplicity, integral, and assignment. The fine structure is abbreviated with s = singlet, d = doublet, t = triplet, q = quartet, m = multiplet, dd = doublet of doublet, tt = triplet of triplet, br = broad, ps = pseudo. Coupling constants $^{n}J_{XY}$ are given in Hertz [Hz]. Here, *n* describes the number of bonds between coupling nuclei X and Y. For internal referencing, the residual proton signals of $CDCl_3$ (^{1}H: 7.26 ppm, ^{13}C: 77.16 ppm), acetone-d_6 (^{1}H: 2.05 ppm, ^{13}C: 206.26 ppm, 29.84 ppm) or DMSO-d_6 (^{1}H: 2.50 ppm, ^{13}C: 39.52 ppm) were used. Signals were assigned using conventional 2D-NMR methods (HSQC, HMBC, COSY). High-resolution mass spectra were recorded on a *Bruker maXis 4G* (*Q-TOF*) *via* electrospray-ionization. Samples were dissolved in acetone or dichloromethane and injected *via* flow-injection. FT-IR spectra were measured on *a FT/IR-4600* (*Jasco*). For this, concentrated DCM solutions of the compounds were drop-casted. Melting points were determined using a *BÜCHI B-540* or *via* Dynamic Scanning Calorimetry (DSC, *vide infra*).

Optical spectroscopy

UV-Vis spectra were measured on a UV-2700 UV/Vis spectrophotometer from *Shimadzu Corporation* with baseline correction. Excitation and emission spectra were recorded on an RF-6000 spectrometer from *Shimadzu Corporation*. Diluted samples were prepared in the *Semi-Micro* quartz cuvettes (1.4 mL, 10x4 mm) from *Hellma Analytics*. Solid samples were placed between two quartz glass slides in 45° orientation to the excitation and emission slits. Appropriate long-pass filters were used to suppress the lamp signals. Measurements were conducted at room temperature. Absolute photoluminescence (PL) quantum yields were measured with the stand-alone absolute PL quantum yield measurement system

C9920-02 by *Hamamatsu Photonics* equipped with an integrating sphere, an L9799-01 CW Xe light source (150 W), a monochromator, and a C7473 photonic multi-channel analyzer. The data were analyzed using the U6039-05 software (*Hamamatsu Photonics*, Ltd., Shizuoka, Japan). Steady-state excitation and emission spectra were recorded on a *FluoTime 300* spectrometer from *PicoQuant* equipped with a 300 W ozone-free Xe lamp (250-900 nm), a 10 W Xe flash-lamp (250-900 nm, pulse width ca. 1 µs) with repetition rates of 0.1 – 300 Hz, a double-grating excitation monochromator (Czerny-Turner type, grating with 1200 lines/mm, blaze wavelength: 300 nm), diode lasers (pulse width < 80 ps) operated by a computer-controlled laser driver PDL-828 “Sepia II” (repetition rate up to 80 MHz, burst mode for slow and weak decays), two emission monochromators (Czerny-Turner, selectable between a double-grating blazed at 500 nm with 2.7 nm/mm dispersion and 1200 lines/mm, or a single-grating blazed at 1200 nm with 5.4 nm/mm dispersion and 600 lines/mm) with adjustable slit width between 25 µm and 7 mm, Glan-Thompson polarizers for excitation (after the Xe-lamps) and emission (after the sample). Different sample holders (Peltier-cooled mounting unit ranging from -15 to 110 °C or an adjustable front-face sample holder), along with two detectors (namely a PMA Hybrid-07 from *PicoQuant* with transit time spread FWHM < 50 ps, 200 – 850 nm, or a H10330C-45-C3 NIR detector with transit time spread FWHM 0.4 ns, 950-1400 nm from *Hamamatsu*) were used. Steady-state spectra and photoluminescence lifetimes were recorded in TCSPC mode by a *PicoHarp 300* (minimum base resolution 4 ps) or in MCS mode by a *TimeHarp 260* (where up to several ms can be traced). Emission and excitation spectra were corrected for source intensity (lamp and grating) by standard correction curves. For samples with lifetimes in the ns order, an instrument response function calibration (IRF) was performed using a diluted *Ludox*® dispersion. Lifetime analysis was performed using the commercial *EasyTau 2* software (*PicoQuant*). The quality of the fit was assessed by minimizing the reduced chi squared function (χ^2) and visual inspection of the weighted residuals and their autocorrelation. All solvents used were of spectrometric grade (*Uvasol*®, *Merck*). For data analysis and visualization, *OriginPro 2023* (*OriginLab™*) was used. CIE coordinates were constructed using an *OriginLab™* Plugin (Chromaticity Diagram, File Version 1.20, License: Free).

Photographs

Photographs of the samples were taken with a *Canon EOS R10* with a *Canon 18-150* lens. The crystals were imaged using an *Olympus SZX16* microscope with an *Olympus KL 1500 LCD* lamp, a *Bresser MikroCam II Full HD HSP* camera, and UV light from *StarLight Opto-Electronics* (type *TC 250*).

2 Synthetic Procedures

General Procedures

General procedure (GP1): Copper-Catalyzed Ullmann-Goldberg Type *N*-Arylation of Aminophenols

In a flame-dried pressure tube, corresponding 2-aminophenol (2 eq.), aryl iodide (1 eq.), copper(I)iodide (0.2 eq.), and dried potassium phosphate (2 eq.) were suspended in anhydrous *N,N*-dimethylformamide under argon atmosphere. The mixture was stirred at 80 °C until full consumption of the electrophile (TLC monitoring, $FeCl_3$-stain solution). Then, the reaction mixture was transferred into a round-bottom flask, concentrated, and dry-mounted over Celite® using ethyl acetate. The residue was purified by column chromatography and dried *in vacuo*.

General procedure (GP2): Nucleophilic Aromatic Substitutions (S_NAr)

In an argon-flushed pressure tube, di- or tetrafluoro-electrophile (1 eq.), 2-aminophenol-nucleophile (1–2 eq.), and dried potassium carbonate (3-6 eq.) were suspended in anhydrous *N,N*-dimethylformamide and stirred at 25–100 °C until full consumption of the electrophile (TLC monitoring). Then, the reaction mixture was transferred into a round-bottom flask, concentrated, and dry-mounted over Celite® using dichloromethane. The residue was purified by column chromatography and lyophilized.

Overview of performed syntheses

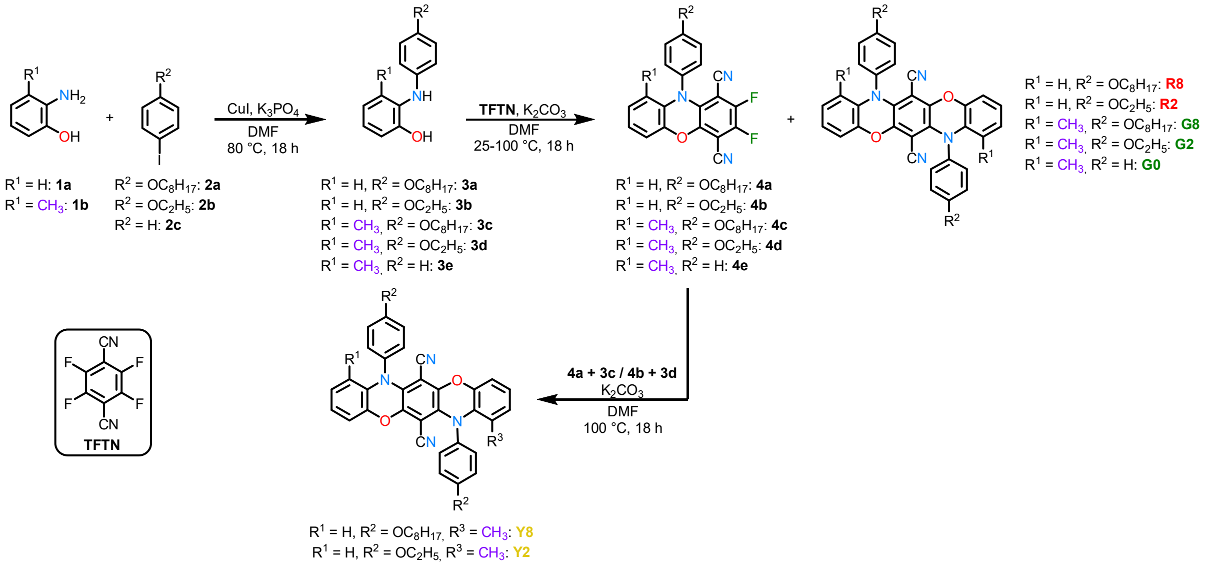

Scheme S1: Overview of performed syntheses.

Overview of synthesized compounds

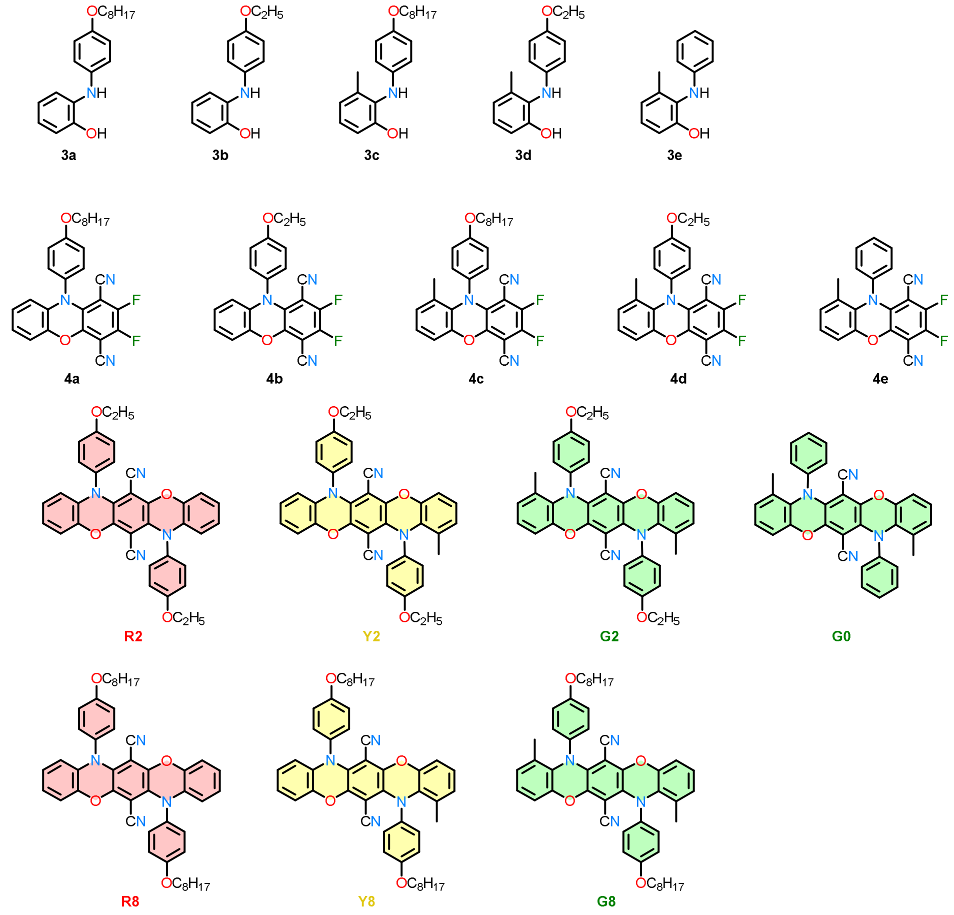

Figure S1: Overview of synthesized compounds.

SYNTHETIC PROCEDURES

1-Iodo-4-(octyloxy)benzene (2a)

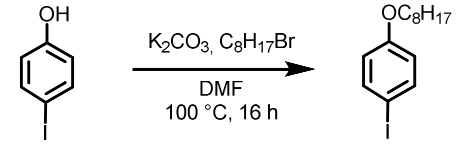

2a

Compound **2a** was synthesized using a literature known procedure.[2] 1.69 g (7.69 mmol, 1.0 eq.) of 4-Iodophenol, 1.60 mL (1.78 g, 9.20 mmol, 1.2 eq.) 1-octylbromide and 1.68 g (12.2 mmol, 1.6 eq.) of potassium carbonate were suspended in 40 mL DMF and stirred at 100 °C for 16 hours. Afterward, the mixture was washed with 400 mL brine and extracted twice with ethyl acetate. The combined organic layers were washed with brine again, before drying over magnesium sulfate. After filtration, the solvent was removed under reduced pressure. The crude product was purified by column chromatography on silica gel using cyclohexane as eluent. The product **2a** (2.08 g, 6.27 mmol, 82%) was obtained as a colorless liquid.

M($C_{14}H_{21}IO$): 332.23 g/mol.

^{1}H-NMR (400 MHz, $CDCl_3$, 298 K): δ [ppm] = 7.53 (m, 2H), 6.67 (m, 2H), 3.91 (t, ^3J = 6.6 Hz, 2H), 1.76 (m, 2H), 1.43 (m, 2H), 1.30 (m, 8H), 0.89 (m, 3H).

Spectroscopic data match literature values.[2]

2-((4-(Octyloxy)phenyl)amino)phenol (3a)

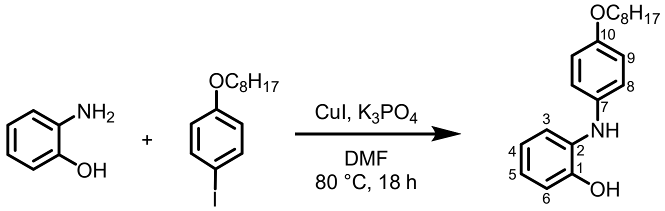

1a **2a** **3a**

3a was synthesized according to **GP1** using 2-aminophenol (**1a**, 0.157 g, 1.44 mmol, 2 eq.), 1-iodo-4-(acetyloxy)benzene (**2a**, 0.235 g, 0.707 mmol, 1 eq.), copper(I)iodide (31.3 mg, 0.164 mmol, 0.23 eq.), and tripotassium phosphate (0.294 g, 1.39 mmol, 2 eq.) in anhydrous *N,N*-dimethylformamide (3 mL). Column chromatography (4.5x30 cm, cyclohexane/ethyl acetate 5/1) yielded the product **3a** as a brownish waxy solid (0.164 g, 0.522 mmol, 74%).

M($C_{20}H_{27}NO_2$): 313.44 g/mol.

^{1}H-NMR (400 MHz, acetone-d_6, 298 K): δ [ppm] = 8.26 (s, 1H, O*H*), 7.05 (m, 3H, *H*8, *H*3), 6.83 (m, 3H, *H*9, *H*6), 6.72 (dt, $^3J_{H-H}$ = 7.6 Hz, $^4J_{H-H}$ = 1.8 Hz, 1H, *H*4), 6.67 (dt, $^3J_{H-H}$ = 7.5 Hz, $^4J_{H-H}$ = 1.8 Hz, 1H, *H*5), 6.32 (s, 1H, N*H*), 3.95 (q, $^3J_{H-H}$ = 6.5 Hz, 1H, OCH_2), 1.75 (m, 2H, OCCH_2), 1.47 (m, 2H, OCCCH_2), 1.33 (m, 8H, residual CH_2), 0.89 (m, 3H, CH_3).

^{13}C{^{1}H}-NMR (101 MHz, acetone-d_6, 298 K): δ [ppm] = 154.9 (*C*10), 147.2 (*C*1), 137.8 (*C*7), 134.3 (*C*2), 121.4 (*C*8), 120.8 (*C*4), 120.6 (*C*5), 116.1 (*C*3), 116.0 (*C*9), 115.6 (*C*6), 68.8 (O*C*H_2), 32.6 (*C*H_2), 30.2, 30.1 (3 *C*H_2, overlapping with acetone signal), 26.8 (*C*H_2), 23.3 (*C*H_2), 14.4 (*C*H_3).

HR-MS (ESI-pos, 70 eV): *m/z* = 314.2117 $[M + H]^+$, calculated for $[C_{20}H_{27}N_1O_2 + H]^+$ = 314.2115.

IR: $\tilde{\nu}$ [cm^{-1}] = 3328, 2922, 2853, 1773, 1701, 1604, 1505, 1468, 1392, 1284, 1230, 1169, 1104, 1028, 925, 823, 744, 508.

2-((4-Ethoxyphenyl)amino)phenol (3b)

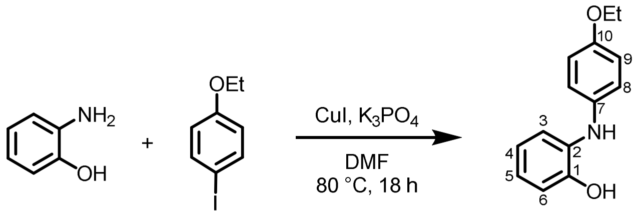

1a **2b** **3b**

3b was synthesized according to **GP1** using 2-aminophenol (**1a**, 0.544 g, 4.99 mmol, 2 eq.), 1-ethoxy-4-iodobenzene (**2b**, 0.621 g, 2.50 mmol, 1 eq.), copper(I)iodide (94.0 mg, 0.494 mmol, 0.2 eq.), and tripotassium phosphate (1.10 g, 5.18 mmol, 2.1 eq.) in anhydrous *N,N*-dimethylformamide (5 mL). Column chromatography (4.5x35 cm, cyclohexane/ethyl acetate 6/1) yielded the product **3b** as a brownish powder (0.245 g, 1.07 mmol, 43%).

M($C_{14}H_{15}NO_2$): 229.28 g/mol.

^{1}H-NMR (400 MHz, acetone-*d*$_6$, 298 K): δ [ppm] = 8.25 (s, 1H, O*H*), 7.05 (m, 3H, *H*8, *H*3), 6.84 (m, 3H, *H*9, *H*6), 6.72 (dt, $^3J_{H\text{-}H}$ = 7.6 Hz, $^4J_{H\text{-}H}$ = 1.8 Hz, 1H, *H*4), 6.67 (dt, $^3J_{H\text{-}H}$ = 7.5 Hz, $^4J_{H\text{-}H}$ = 1.8 Hz, 1H, *H*5), 6.32 (s, 1H, N*H*), 4.00 (q, $^3J_{H\text{-}H}$ = 7.0 Hz, 2H, C*H*2), 1.34 (t, $^3J_{H\text{-}H}$ = 7.0 Hz, 3H, C*H*3).

^{13}C{^{1}H}-NMR (101 MHz, acetone-*d*$_6$, 298 K): δ [ppm] = 154.7 (*C*10), 147.2 (*C*1), 137.8 (*C*7), 134.2 (*C*2), 121.3 (*C*8), 120.8 (*C*4), 120.6 (*C*5), 116.2 (*C*3), 116.0 (*C*9), 115.6 (*C*6), 64.2 (*C*H2), 15.2 (*C*H3).

HR-MS (ESI-pos, 70 eV): *m/z* = 230.1180 [M + H]$^+$, calculated for $[C_{14}H_{15}N_1O_2 + H]^+$ = 230.1176.

IR: $\tilde{\nu}$ [cm^{-1}] = 3331, 3065, 3044, 2976, 2924, 2872, 1773, 1701, 1603, 1584, 1503, 1390, 1281, 1227, 1168, 1110, 1039, 918, 815, 746.

Melting point: 189 °C.

3-Methyl-2-((4-(octyloxy)phenyl)amino)phenol (3c)

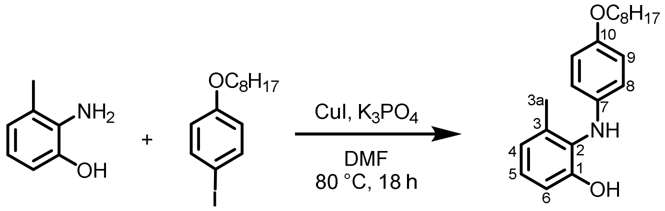

1b **2a** **3c**

3c was synthesized according to **GP1** using 2-amino-3-methylphenol (**1b**, 0.180 g, 1.46 mmol, 2 eq.), 1-iodo-4-(octyloxy)benzene (**2a**, 0.248 g, 0.745 mmol, 1 eq.), copper(I)iodide (29.9 mg, 0.157 mmol, 0.2 eq.), and tripotassium phosphate (0.319 g, 1.50 mmol, 2 eq.) in anhydrous *N,N*-dimethylformamide (3 mL). Column chromatography (4.5x32 cm, cyclohexane/ethyl acetate 5/1) yielded the product **3c** as a brownish powder (0.169 g, 0.515 mmol, 69%).

M($C_{21}H_{29}NO_2$): 327.47 g/mol.

^{1}H-NMR (400 MHz, acetone-*d*$_6$, 298 K): δ [ppm] = 7.72 (s, 1H, O*H*), 6.99 (ps t, $^3J_{H\text{-}H}$ = 7.8 Hz, 1H, *H*5), 6.79 (d, $^3J_{H\text{-}H}$ = 8.0 Hz, 1H, *H*6), 6.73 (m, 3H, *H*9, *H*4), 6.52 (m, 2H, *H*8), 6.08 (s, 1H, N*H*), 3.88 (t, $^3J_{H\text{-}H}$ = 6.5 Hz, 1H, OC*H*2), 2.11 (s, 3H, *H*3a), 1.71 (m, 2H, OCC*H*2), 1.45 (m, 2H, OCCC*H*2), 1.33 (m, 8H, residual C*H*2), 0.89 (m, 3H, C*H*3).

^{13}C{^{1}H}-NMR (101 MHz, acetone-*d*$_6$, 298 K): δ [ppm] = 154.8 (*C*1), 153.2 (*C*10), 141.8 (*C*7), 137.4 (*C*3), 129.2 (*C*2), 127.0 (*C*5), 122.4 (*C*4), 116.1 (*C*9), 116.0 (*C*8), 113.6 (*C*6), 69.0 (O*C*H2), 32.6 (*C*H2), 30.3 (3 *C*H2, overlapping with acetone signal), 26.8 (*C*H2), 23.3 (*C*H2), 18.2 (*C*3a), 14.4 (*C*H3).

HR-MS (ESI-pos, 70 eV): *m/z* = 328.2278 [M + H]$^+$, calculated for $[C_{21}H_{29}N_1O_2 + H]^+$ = 328.2271.

IR: $\tilde{\nu}$ [cm^{-1}] = 3363, 2923, 2853, 1641, 1593, 1505, 1469, 1391, 1281, 1222, 1158, 1117, 1075, 1029, 951, 821, 766, 718, 611, 512.

2-((4-Ethoxyphenyl)amino)-3-methylphenol (3d)

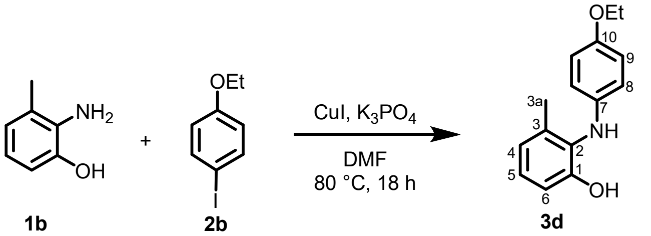

3d was synthesized according to **GP1** using 2-amino-3-methylphenol (**1b**, 0.400 g, 3.25 mmol, 2 eq.), 1-ethoxy-4-iodobenzene (**2b**, 0.390 g, 1.57 mmol, 1 eq.), copper(I)iodide (62.0 mg, 0.326 mmol, 0.2 eq.), and tripotassium phosphate (0.689 g, 3.25 mmol, 2 eq.) in anhydrous *N,N*-dimethylformamide (3.5 mL). Column chromatography (4.5x32 cm, cyclohexane/ethyl acetate 8/1) yielded the product **3d** as a brownish oil (0.240 g, 0.986 mmol, 63%).

M($C_{15}H_{17}NO_2$): 243.31 g/mol.

^{1}H-NMR (400 MHz, DMSO-*d*$_6$, 298 K): δ [ppm] = 8.99 (s, 1H, O*H*), 6.89 (ps t, $^3J_{H\text{-}H}$ = 7.7 Hz, 1H, *H*5), 6.72 (d, $^3J_{H\text{-}H}$ = 7.7 Hz, 1H, *H*6), 6.67 (m, 3H, *H*9, *H*4), 6.57 (s, 1H, N*H*), 6.41 (m, 2H, *H*8), 3.88 (q, $^3J_{H\text{-}H}$ = 7.0 Hz, 2H, OC*H*$_2$), 2.04 (s, 3H, *H*3a), 1.26 (t, $^3J_{H\text{-}H}$ = 7.0 Hz, 3H, C*H*$_3$).

^{13}C{^{1}H}-NMR (101 MHz, DMSO-*d*$_6$, 298 K): δ [ppm] = 152.9 (*C*1), 150.7 (*C*10), 140.9 (*C*7), 135.5 (*C*3), 128.4 (*C*2), 124.9 (*C*5), 121.0 (*C*4), 115.0 (*C*9), 114.6 (*C*8), 113.3 (*C*6), 63.3 (*C*H$_2$), 18.0 (*C*3a), 14.9 (*C*H$_3$).

HR-MS (ESI-pos, 70 eV): *m/z* = 244.1338 [M + H]$^+$, calculated for [$C_{15}H_{17}N_1O_2$ + H]$^+$ = 244.1332.

IR: $\tilde{\nu}$ [cm^{-1}] = 3365, 3040, 2976, 2923, 2871, 1719, 1649, 1597, 1504, 1469, 1392, 1343, 1280, 1260, 1220, 1158, 1116, 1042, 978, 950, 921, 820, 770, 722, 611, 514.

3-Methyl-2-(phenylamino)phenol (3e)

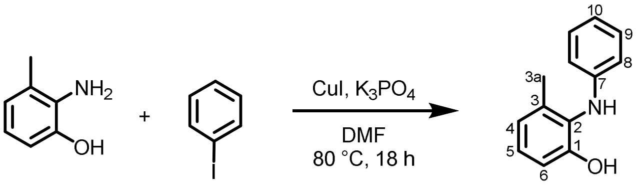

3e was synthesized according to **GP1** using 2-amino-3-methylphenol (**1b**, 0.505 g, 4.10 mmol, 2 eq.), iodobenzene (**2c**, 227 µL, 0.414 g, 2.03 mmol, 1 eq.), copper(I)iodide (77.8 mg, 0.409 mmol, 0.2 eq.), and tripotassium phosphate (0.901 g, 4.07 mmol, 2 eq.) in anhydrous *N,N*-dimethylformamide (5 mL). Column chromatography (5x33 cm, cyclohexane/ethyl acetate 5/1) yielded the product **3e** as an off-white powder (0.286 g, 1.43 mmol, 71%).

M($C_{13}H_{13}NO$): 199.25 g/mol.

^{1}H-NMR (400 MHz, $CDCl_3$, 298 K): δ [ppm] = 7.20 (m, 2H, *H*9), 7.13 (t, $^3J_{H\text{-}H}$ = 8.0 Hz, 1H, *H*5), 6.90 (d, $^3J_{H\text{-}H}$ = 8.0 Hz, 1H, *H*6), 6.84 (tt, $^3J_{H\text{-}H}$ = 7.2 Hz, $^4J_{H\text{-}H}$ = 1.1 Hz, 1H, *H*10), 6.79 (d, $^3J_{H\text{-}H}$ = 8.0 Hz, 1H, *H*4), 6.62 (m, 2H, *H*8), 6.32 (br s, 1H, N*H*), 2.14 (s, 3H, *H*3a). The O*H* signal is missing due to fast proton exchange processes.

^{13}C{^{1}H}-NMR (101 MHz, $CDCl_3$, 298 K): δ [ppm] = 154.3 (*C*1), 146.2 (*C*7), 137.3 (*C*3), 129.7 (*C*9), 128.2 (*C*5), 126.2 (*C*2), 122.3 (*C*4), 120.0 (*C*10), 114.4 (*C*8), 112.6 (*C*6), 17.9 (*C*3a).

HR-MS (ESI-pos, 70 eV): *m/z* = 200.1071 [M + H]$^+$, calculated for [$C_{13}H_{13}N_1O_1$ + H]$^+$ = 200.1070.

IR: $\tilde{\nu}$ [cm^{-1}] = 3380, 3080, 3047, 3013, 2941, 2917, 1933, 1597, 1495, 1471, 1441, 1410, 1350, 1284, 1256, 1231, 1176, 1076, 1025, 950, 874, 787, 747, 691, 615.

Melting point: 120 °C.

Compounds 4a & R8

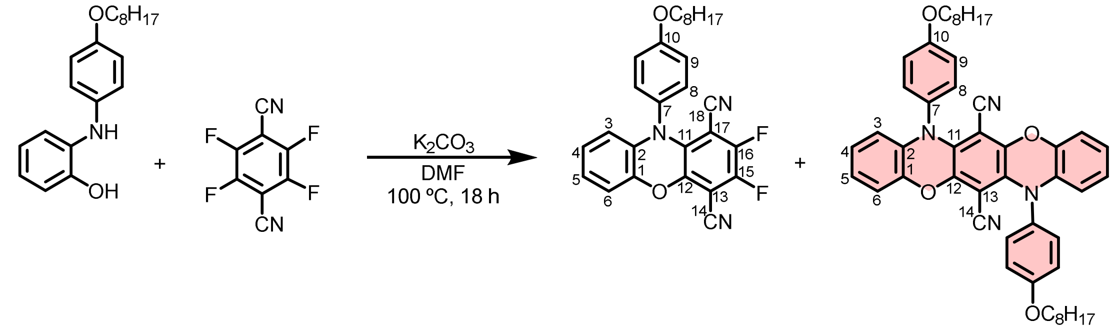

3a **TFTN** **4a** 45% **R8** 25%

Adopting **GP2**, compound **3a** (0.226 g, 0.721 mmol, 1.4 eq.), tetrafluoroterephthalonitrile (**TFTN**, 0.100 g, 0.500 mmol, 1 eq.), and potassium carbonate (0.380 g, 2.75 mmol, 5.5 eq.) were stirred in anhydrous *N,N*-dimethylformamide (15 mL) at 100 °C overnight. Column chromatography (6x40 cm, DCM/*n*-pentane 1/1 → 1/3) yielded product **4a** as a yellow powder (0.106 g, 0.224 mmol, 45%) and product **R8** as a red powder (0.095 g, 0.127 mmol, 25%) after washing with cyclohexane.

Characterization of compound **4a**:

M($C_{28}H_{25}F_2N_3O_2$): 473.52 g/mol.

^{1}H-NMR (600 MHz, $CDCl_3$, 298 K): δ [ppm] = 7.28 (d, $^3J_{H\text{-}H}$ = 8.8 Hz, 2H, *H*8), 7.07 (d, $^3J_{H\text{-}H}$ = 8.8 Hz, 2H, *H*9), 6.84 (m, 2H, *H*5, *H*6), 6.75 (m, 1H, *H*4), 6.13 (d, $^3J_{H\text{-}H}$ = 8.1 Hz, 1H, *H*3), 4.02 (q, $^3J_{H\text{-}H}$ = 6.6 Hz, 2H, OCH_2), 1.82 (m, 2H, OCCH_2), 1.47 (m, 2H, OCCCH_2), 1.31 (m, 8H, residual CH_2), 0.89 (m, 3H, CH_3).

^{13}C{^{1}H}-NMR (151 MHz, $CDCl_3$, 298 K): δ [ppm] = 161.3 (*C*10), 146.5 (dd, $^1J_{C\text{-}F}$ = 254.0 Hz, $^2J_{C\text{-}F}$ = 13.3 Hz, *C*16/*C*15), 144.3 (t, $^3J_{C\text{-}F}$ = 3.4 Hz, *C*12), 142.2 (dd, $^1J_{C\text{-}F}$ = 254.4 Hz, $^2J_{C\text{-}F}$ = 15.4 Hz, *C*15/*C*16), 141.8 (*C*1), 135.2 (d, $^3J_{C\text{-}F}$ = 2.2 Hz, *C*11), 132.4 (*C*8), 131.3 (*C*2), 129.2 (*C*7), 125.6 (*C*4), 124.7 (*C*5), 116.8 (*C*9), 116.2 (*C*6), 115.4 (*C*3), 108.9 (d, $^3J_{C\text{-}F}$ = 3.6 Hz, *C*18/*C*14), 108.3 (d, $^3J_{C\text{-}F}$ = 3.7 Hz, *C*14/*C*18), 95.3 (dd, $^2J_{C\text{-}F}$ = 16.8 Hz, $^3J_{C\text{-}F}$ = 2.5 Hz, *C*17/*C*13), 91.6 (d, $^2J_{C\text{-}F}$ = 18.5 Hz, *C*13/*C*17), 68.7 (O*C*H_2), 32.0 (*C*H_2), 29.5 (*C*H_2), 29.4 (*C*H_2), 29.2 (*C*H_2), 26.1 (*C*H_2), 22.8 (*C*H_2), 14.3 (*C*H_3).

^{19}F-NMR (565 MHz, $CDCl_3$, 298 K): δ [ppm] = -135.9 (d, $^3J_{F\text{-}F}$, 20.4 Hz), -143.0 (d, $^3J_{F\text{-}F}$, 20.4 Hz).

HR-MS (ESI-pos, 70 eV): *m/z* = 474.1986 [M + H]$^+$, calculated for [$C_{28}H_{25}F_2N_3O_2$ + H]$^+$ = 474.1988; 496.1810 [M + Na]$^+$, calculated for [$C_{28}H_{25}F_2N_3O_2$ + Na]$^+$ = 496.1807.

IR: $\tilde{\nu}$ [cm^{-1}] = 2923, 2853, 2223, 1649, 1610, 1560, 1507, 1461, 1440, 1382, 1312, 1287, 1247, 1167, 1106, 1041, 1007, 971, 930, 832, 753, 626, 598, 531, 483.

Melting point: 156 °C.

Characterization of compound **R8**:

M($C_{48}H_{50}N_4O_4$): 746.95 g/mol.

^{1}H-NMR (600 MHz, CD_2Cl_2, 298 K): δ [ppm] = 7.35 (d, $^3J_{H\text{-}H}$ = 8.8 Hz, 4H, *H*8), 7.04 (d, $^3J_{H\text{-}H}$ = 8.8 Hz, 4H, *H*9), 6.77 (m, 2H, *H*5), 6.73 (m, 4H, *H*4, *H*6), 6.32 (m, 2H, *H*3), 4.02 (q, $^3J_{H\text{-}H}$ = 6.6 Hz, 4H, OCH_2), 1.82 (m, 4H, OCCH_2), 1.48 (m, 4H, OCCCH_2), 1.34 (m, 16H, residual CH_2), 0.90 (m, 6H, terminal CH_3).

^{13}C{^{1}H}-NMR (151 MHz, CD_2Cl_2, 298 K): δ [ppm] = 160.5 (*C*10), 144.5 (*C*12), 143.8 (*C*1), 133.7 (*C*7), 133.4 (*C*2), 132.7 (*C*8), 130.4 (*C*11), 125.1 (*C*4), 124.1 (*C*5), 116.7 (*C*3), 116.4 (*C*9), 116.3 (*C*6), 111.0

(*C*14), 93.4 (*C*13), 69.1 (O*C*H2), 32.4 (*C*H2), 29.9 (*C*H2), 29.8 (*C*H2), 29.8 (*C*H2), 26.6 (*C*H2), 23.2 (*C*H2), 14.4 (*C*H3).

HR-MS (ESI-pos, 70 eV): *m/z* = 747.3886 [M + H]+, calculated for $[C_{48}H_{50}N_4O_4 + H]^+$ = 747.3905; 769.3718 [M + Na]+, calculated for $[C_{48}H_{50}N_4O_4 + Na]^+$ = 769.3724.

IR: $\tilde{\nu}$ [cm^{-1}] = 2943, 2923, 2857, 2222, 1606, 1506, 1449, 1421, 1310, 1281, 1249, 1222, 1164, 1107, 1056, 1017, 960, 939, 896, 833, 751, 729, 624, 600, 529, 452, 431.

Melting point: 244 °C.

Compounds 4b & R2

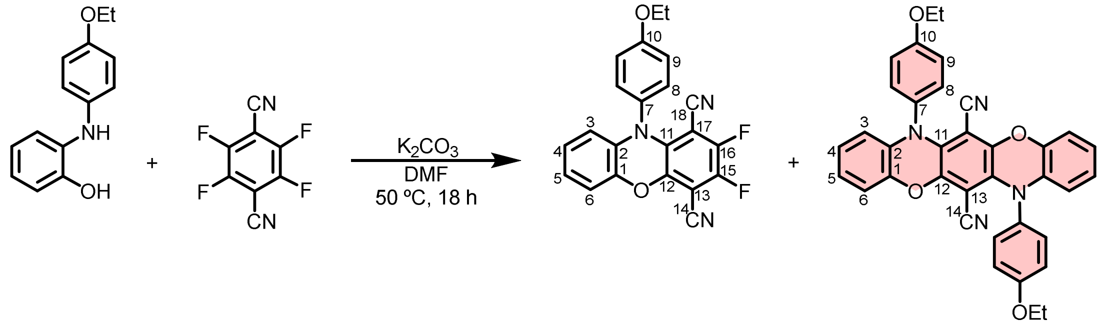

3b **TFTN** **4b** 52% **R2** 15%

Adopting **GP2**, compound **3b** (0.181 g, 0.789 mmol, 1.4 eq.), tetrafluoroterephthalonitrile (**TFTN**, 0.113 g, 0.565 mmol, 1 eq.), and potassium carbonate (0.380 g, 2.75 mmol, 4.9 eq.) were stirred in anhydrous *N,N*-dimethylformamide (9 mL) at 50 °C overnight. Column chromatography (6x35 cm, DCM/*n*-pentane 2/1 → 4/1 → 8/1 → 100/0) yielded product **4b** as a yellow powder (0.114 g, 0.293 mmol, 52%) and product **R2** as a red powder (0.050 g, 0.086 mmol, 15%) after washing with cyclohexane.

Characterization of compound **4b**:

M($C_{22}H_{13}F_2N_3O_2$): 389.36 g/mol.

^{1}H-NMR (600 MHz, CDCl$_3$, 298 K): δ [ppm] = 7.28 (d, $^3J_{H\text{-}H}$ = 8.9 Hz, 2H, *H*8), 7.07 (d, $^3J_{H\text{-}H}$ = 8.9 Hz, 2H, *H*9), 6.84 (m, 2H, *H*5, *H*6), 6.75 (m, 1H, *H*4), 6.12 (d, $^3J_{H\text{-}H}$ = 8.1 Hz, 1H, *H*3), 4.11 (q, $^3J_{H\text{-}H}$ = 7.0 Hz, 2H, C*H*2), 1.46 (t, $^3J_{H\text{-}H}$ = 7.0 Hz, 3H, C*H*3).

^{13}C{^{1}H}-NMR (151 MHz, CDCl$_3$, 298 K): δ [ppm] = 161.1 (*C*10), 146.5 (dd, $^1J_{C\text{-}F}$ = 254.0 Hz, $^2J_{C\text{-}F}$ = 13.3 Hz, *C*16/*C*15), 144.4 (t, $^3J_{C\text{-}F}$ = 3.2 Hz, *C*12), 142.2 (dd, $^1J_{C\text{-}F}$ = 254.0 Hz, $^2J_{C\text{-}F}$ = 14.9 Hz, *C*15/*C*16), 141.8 (*C*1), 135.2 (d, $^3J_{C\text{-}F}$ = 2.2 Hz, *C*11), 132.4 (*C*8), 131.3 (*C*2), 129.3 (*C*7), 125.6 (*C*4), 124.7 (*C*5), 116.8 (*C*9), 116.2 (*C*6), 115.4 (*C*3), 108.9 (d, $^3J_{C\text{-}F}$ = 3.4 Hz, *C*18/*C*14), 108.3 (d, $^3J_{C\text{-}F}$ = 3.9 Hz, *C*14/*C*18), 95.4 (dd, $^2J_{C\text{-}F}$ = 16.7 Hz, $^3J_{C\text{-}F}$ = 2.3 Hz, *C*17/*C*13), 91.5 (d, $^2J_{C\text{-}F}$ = 18.7 Hz, *C*13/*C*17), 64.1 (*C*H2), 14.8 (*C*H3).

^{19}F-NMR (565 MHz, CDCl$_3$, 298 K): δ [ppm] = -135.9 (d, $^3J_{F\text{-}F}$, 20.5 Hz), -143.0 (d, $^3J_{F\text{-}F}$, 20.5 Hz).

HR-MS (ESI-pos, 70 eV): *m/z* = 390.1047 [M + H]+, calculated for $[C_{22}H_{13}F_2N_3O_2 + H]^+$ = 390.1049; 412.0872 [M + Na]+, calculated for $[C_{22}H_{13}F_2N_3O_2 + Na]^+$ = 412.0868.

IR: $\tilde{\nu}$ [cm^{-1}] = 3065, 2990, 2935, 2235, 2224, 1647, 1607, 1560, 1507, 1468, 1438, 1390, 1314, 1262, 1248, 1168, 1111, 1040, 1008, 965, 919, 844, 750, 622, 592, 533.

Melting point: 219 °C.

Crystals were grown using a concentrated solution of **4b** in chloroform overlayered with methanol.

Characterization of compound **R2**:

M($C_{36}H_{26}N_4O_4$): 578.63 g/mol.

^{1}H-NMR (600 MHz, CD_2Cl_2, 298 K): δ [ppm] = 7.36 (m, 4H, *H*8), 7.03 (m, 4H, *H*9), 6.77 (m, 2H, *H*5), 6.73 (m, 4H, *H*4, *H*6), 6.32 (m, 2H, *H*3), 4.10 (q, $^3J_{H\text{-}H}$ = 7.0 Hz, 4H, CH_2), 1.44 (t, $^3J_{H\text{-}H}$ = 7.0 Hz, 6H, CH_3).

^{13}C{^{1}H}-NMR (151 MHz, CD_2Cl_2, 298 K): δ [ppm] = 160.3 (*C*10), 144.5 (*C*12), 143.8 (*C*1), 133.7 (*C*7), 133.3 (*C*2), 132.8 (*C*8), 130.4 (*C*11), 125.1 (*C*4), 124.1 (*C*5), 116.6 (*C*3), 116.4 (*C*9), 116.3 (*C*6), 111.0 (*C*14), 93.4 (*C*13), 64.5 (*C*H_2), 15.1 (*C*H_3).

HR-MS (ESI-pos, 70 eV): *m/z* = 579.2003 [M + H]$^+$, calculated for [$C_{36}H_{26}N_4O_4$ + H]$^+$ = 579.2027; 601.1835 [M + Na]$^+$, calculated for [$C_{36}H_{26}N_4O_4$ + Na]$^+$ = 601.1846.

IR: $\tilde{\nu}$ [cm^{-1}] = 2969, 2933, 2887, 2220, 1888, 1601, 1578, 1504, 1449, 1416, 1391, 1313, 1294, 1247, 1222, 1161, 1114, 1043, 1015, 923, 894, 852, 822, 751, 694, 628, 593, 525, 436.

Melting point: 336 °C.

Crystals were grown using a concentrated solution of **R2** in DCM overlayered with cyclohexane.

Compounds 4c & G8

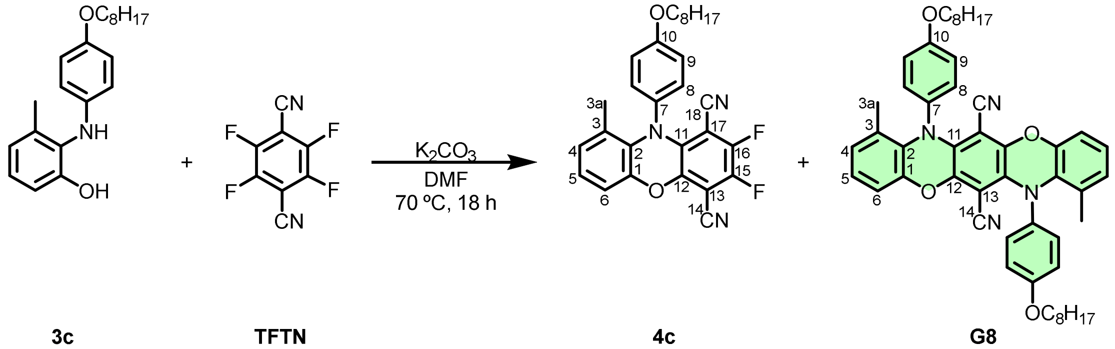

3c **TFTN** **4c** 32% **G8** 28%

Adopting **GP2**, compound **3c** (0.406 g, 1.24 mmol, 1.3 eq.), tetrafluoroterephthalonitrile (**TFTN**, 0.190 g, 0.950 mmol, 1 eq.), and potassium carbonate (0.486 g, 3.52 mmol, 3.7 eq.) were stirred in anhydrous *N,N*-dimethylformamide (15 mL) at 70 °C overnight. Column chromatography (6x38 cm, DCM/*n*-pentane 1/1 → 2/1) yielded product **4c** as a yellow oil (0.150 g, 0.308 mmol, 32%) and product **G8** as a yellow powder (0.265 g, 0.205 mmol, 28%) after washing with *n*-pentane.

Characterization of compound **4c**:

M($C_{29}H_{27}F_2N_3O_2$): 487.55 g/mol.

^{1}H-NMR (600 MHz, $CDCl_3$, 298 K): δ [ppm] = 7.54 (d, $^3J_{H\text{-}H}$ = 8.9 Hz, 2H, *H*8), 7.06 (m, 2H, *H*5, *H*6), 6.95 (m, 1H, *H*4), 6.84 (d, $^3J_{H\text{-}H}$ = 8.9 Hz, 2H, *H*9), 3.90 (t, $^3J_{H\text{-}H}$ = 6.5 Hz, 2H, OCH_2), 2.22 (s, 3H, *H*3a), 1.74 (m, 2H, OCCH_2), 1.42 (m, 2H, OCCCH_2), 1.28 (m, 8H, residual CH_2), 0.88 (m, 3H, CH_3).

^{13}C{^{1}H}-NMR (151 MHz, $CDCl_3$, 298 K): δ [ppm] = 158.6 (*C*10), 150.5 (t, $^3J_{C\text{-}F}$ = 2.4 Hz, *C*12), 149.3 (*C*1), 147.2 (overlapping dd, $^1J_{C\text{-}F}$ = 258.9 Hz, $^2J_{C\text{-}F}$ = 14.7 Hz, *C*16, *C*15), 141.0 (C7), 138.0 (d, $^3J_{C\text{-}F}$ = 4.1 Hz, *C*11), 134.5 (*C*3), 132.6 (*C*2), 131.0 (*C*8), 128.6 (*C*4), 126.7 (*C*5), 115.3 (*C*9), 114.9 (*C*6), 110.5 (d, $^3J_{C\text{-}F}$ = 3.8 Hz, *C*18/*C*14), 108.5 (d, $^3J_{C\text{-}F}$ = 3.7 Hz, *C*14/*C*18), 103.8 (dd, $^2J_{C\text{-}F}$ = 13.9 Hz, $^3J_{C\text{-}F}$ = 2.5 Hz, *C*17/*C*13), 96.8 (dd, $^2J_{C\text{-}F}$ = 16.0 Hz, $^3J_{C\text{-}F}$ = 2.3 Hz, *C*13/*C*17), 68.4 (O*C*H_2), 31.9 (*C*H_2), 29.4 (*C*H_2), 29.4 (*C*H_2), 29.3 (*C*H_2), 26.1 (*C*H_2), 22.8 (*C*H_2), 18.4 (*C*3a), 14.2 (*C*H_3).

^{19}F-NMR (565 MHz, $CDCl_3$, 298 K): δ [ppm] = -132.3 (d, $^3J_{F\text{-}F}$, 20.5 Hz), -133.9 (d, $^3J_{F\text{-}F}$, 20.5 Hz).

HR-MS (ESI-pos, 70 eV): *m/z* = 488.2143 [M + H]$^+$, calculated for [$C_{29}H_{27}F_2N_3O_2$ + H]$^+$ = 488.2144; 510.1962 [M + Na]$^+$, calculated for [$C_{29}H_{27}F_2N_3O_2$ + Na]$^+$ = 510.1964.

IR: $\tilde{\nu}$ [cm^{-1}] = 2959, 2917, 2851, 2240, 1637, 1610, 1581, 1504, 1456, 1391, 1299, 1248, 1212, 1186, 1171, 1139, 1108, 1072, 1028, 999, 961, 953, 903, 829, 777, 754, 722, 684, 618, 568, 544, 482, 438.

Melting point: 105 °C.

Characterization of compound **G8**:

M($C_{50}H_{54}N_4O_4$): 775.01 g/mol.

^{1}H-NMR (400 MHz, $CDCl_3$, 298 K): δ [ppm] = 7.53 (d, $^3J_{H-H}$ = 8.9 Hz, 4H, *H*8), 7.02 (m, 4H, *H*5, *H*6), 6.89 (m, 2H, *H*4), 6.81 (d, $^3J_{H-H}$ = 8.9 Hz, 4H, *H*9), 3.88 (t, $^3J_{H-H}$ = 6.5 Hz, 4H, OCH_2), 2.22 (s, 6H, *H*3a), 1.73 (m, 4H, OCCH_2), 1.40 (m, 4H, OCCCH_2), 1.30 (m, 16H, residual CH_2), 0.87 (m, 6H, CH_3).

^{13}C{^{1}H}-NMR (101 MHz, $CDCl_3$, 298 K): δ [ppm] = 158.1 (*C*10), 150.5 (*C*12), 150.2 (*C*1), 141.5 (*C*7), 137.4 (*C*11), 134.4 (*C*3), 133.3 (*C*2), 130.6 (*C*8), 127.8 (*C*4), 126.3 (*C*5), 115.1 (*C*9), 114.8 (*C*6), 112.7 (*C*14), 101.8 (*C*13), 68.4 (O*C*H_2), 31.9 (*C*H_2), 29.5 (*C*H_2), 29.4 (*C*H_2), 29.3 (*C*H_2), 26.2 (*C*H_2), 22.8 (*C*H_2), 18.3 (*C*3a), 14.2 (*C*H_3).

HR-MS (ESI-pos, 70 eV): *m/z* = 775.4200 [M + H]$^+$, calculated for [$C_{50}H_{54}N_4O_4$ + H]$^+$ = 775.4218; 797.4021 [M + Na]$^+$, calculated for [$C_{50}H_{54}N_4O_4$ + Na]$^+$ =797.4037.

IR: $\tilde{\nu}$ [cm^{-1}] = 3007, 2956, 2924, 2855, 2231, 1601, 1503, 1469, 1419, 1262, 1213, 1180, 1106, 1085, 1033, 1004, 957, 887, 836, 784, 750, 684, 622, 600, 560, 535, 468, 442, 405.

Melting point: 223 °C.

Compounds 4d & G2

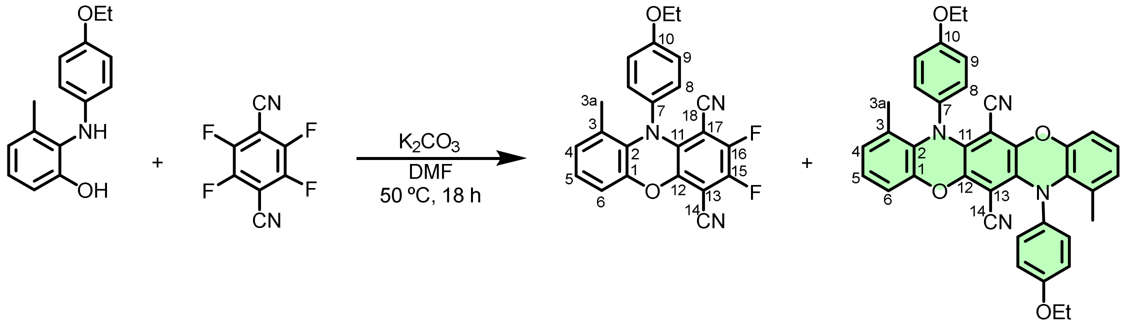

3d **TFTN** **4d** 46% **G2** 13%

Adopting **GP2**, compound **3d** (0.240 g, 0.986 mmol, 1 eq.), tetrafluoroterephthalonitrile (**TFTN**, 0.200 g, 1.00 mmol, 1 eq.), and potassium carbonate (0.530 g, 3.84 mmol, 3.8 eq.) were stirred in anhydrous *N,N*-dimethylformamide (11 mL) at 50 °C overnight. Column chromatography (4.5x40 cm, DCM/*n*-pentane 1/1 → 2/1 → 3/1 → 5/1) yielded product **4b** as a yellow powder (0.184 g, 0.456 mmol, 46%) and product **G2** as a yellow powder (80.9 mg, 0.133 mmol, 13%) after washing with *n*-pentane.

Characterization of compound **4d**:

M($C_{23}H_{15}F_2N_3O_2$): 403.39 g/mol.

^{1}H-NMR (600 MHz, $CDCl_3$, 298 K): δ [ppm] = 7.55 (d, $^3J_{H-H}$ = 8.9 Hz, 2H, *H*8), 7.07 (m, 2H, *H*5, *H*6), 6.95 (m, 1H, *H*4), 6.84 (d, $^3J_{H-H}$ = 8.9 Hz, 2H, *H*9), 3.99 (q, $^3J_{H-H}$ = 7.0 Hz, 2H, OCH_2), 2.22 (s, 3H, *H*3a), 1.39 (t, $^3J_{H-H}$ = 7.0 Hz, 3H, OCH$_2$CH_3).

^{13}C{^{1}H}-NMR (151 MHz, $CDCl_3$, 298 K): δ [ppm] = 158.4 (*C*10), 150.5 (t, $^3J_{C-F}$ = 2.9 Hz, *C*12), 149.4 (*C*1), 147.2 (overlapping dd, $^1J_{C-F}$ = 260.0 Hz, $^2J_{C-F}$ = 13.6 Hz, *C*16, *C*15), 141.0 (C7), 137.9 (d, $^3J_{C-F}$ = 4.1 Hz, *C*11), 134.5 (*C*3), 132.6 (*C*2), 131.0 (*C*8), 128.6 (*C*4), 126.7 (*C*5), 115.3 (*C*9), 114.9 (*C*6), 110.4 (d, $^3J_{C-F}$ = 3.9 Hz, *C*18/*C*14), 108.5 (d, $^3J_{C-F}$ = 3.8 Hz, *C*14/*C*18), 103.8 (dd, $^2J_{C-F}$ = 14.4 Hz, $^3J_{C-F}$ = 2.3 Hz, *C*17/*C*13), 96.8 (dd, $^2J_{C-F}$ = 15.8 Hz, $^3J_{C-F}$ = 1.8 Hz, *C*13/*C*17), 63.9 (O*C*H_2), 18.4 (*C*3a), 14.9 (OCH$_2$*C*H$_3$).

^{19}F-NMR (565 MHz, $CDCl_3$, 298 K): δ [ppm] = -132.3 (d, $^3J_{F-F}$, 20.5 Hz), -133.9 (d, $^3J_{F-F}$, 20.5 Hz).

HR-MS (ESI-pos, 70 eV): *m/z* = 404.1207 [M + H]$^+$, calculated for [$C_{23}H_{15}F_2N_3O_2$ + H]$^+$ = 404.1205; 426.1028 [M + Na]$^+$, calculated for [$C_{23}H_{15}F_2N_3O_2$ + Na]$^+$ = 426.1025.

IR: $\tilde{\nu}$ [cm^{-1}] = 2989, 2963, 2928, 2883, 2320, 2239, 1605, 1580, 1502, 1452, 1387, 1308, 1297, 1246, 1213, 1185, 1169, 1139, 1111, 1093, 1042, 1000, 959, 921, 836, 770, 755, 602, 544, 483.

Melting point: 134 °C.

Crystals were grown using a concentrated solution of **4d** in DCM.

Characterization of compound **G2**:

M($C_{38}H_{30}N_4O_4$): 606.68 g/mol.

^{1}H-NMR (600 MHz, CD_2Cl_2, 298 K): δ [ppm] = 7.52 (d, $^3J_{H\text{-}H}$ = 8.9 Hz, 4H, *H*8), 7.03 (m, 4H, *H*5, *H*6), 6.93 (ddd, $^3J_{H\text{-}H}$ = 7.2 Hz, $^4J_{H\text{-}H}$ = 1.8 Hz, $^4J_{H\text{-}H}$ = 0.9 Hz, 2H, *H*4), 6.81 (d, $^3J_{H\text{-}H}$ = 8.9 Hz, 4H, *H*9), 3.96 (q, $^3J_{H\text{-}H}$ = 7.0 Hz, 4H, OCH_2), 2.23 (s, 6H, *H*3a), 1.35 (t, $^3J_{H\text{-}H}$ = 7.0 Hz, 6H, OCH_2CH_3).

^{13}C{^{1}H}-NMR (151 MHz, CD_2Cl_2, 298 K): δ [ppm] = 158.4 (*C*10), 150.9 (*C*12), 150.7 (*C*1), 142.0 (*C*7), 137.7 (*C*11), 134.9 (*C*3), 133.7 (*C*2), 130.7 (*C*8), 128.1 (*C*4), 126.7 (*C*5), 115.4 (*C*9), 115.1 (*C*6), 112.9 (*C*14), 102.3 (*C*13), 64.3 (OCH_2), 18.5 (*C*3a), 15.1 (CH_3).

HR-MS (ESI-pos, 70 eV): *m/z* = 607.2331 [M + H]$^+$, calculated for [$C_{38}H_{30}N_4O_4$ + H]$^+$ = 607.2340; 629.2152 [M + Na]$^+$, calculated for [$C_{38}H_{30}N_4O_4$ + Na]$^+$ = 629.2159.

IR: $\tilde{\nu}$ [cm^{-1}] = 2985, 2920, 2852, 2231, 1604, 1504, 1478, 1456, 1422, 1389, 1264, 1251, 1215, 1181, 1111, 1088, 1043, 1006, 960, 923, 888, 839, 805, 785, 766, 747, 682, 660, 620, 601, 561, 539, 464, 410.

Melting point: 124 °C.

Compounds 4e & G0

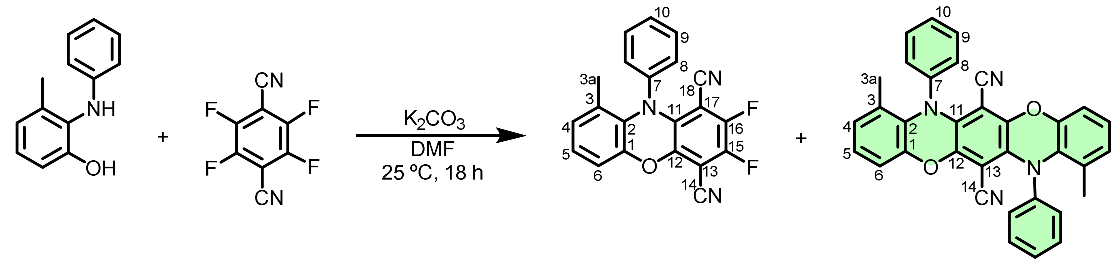

3e **TFTN** **4e** 55% **G0** 17%

Adopting **GP2**, compound **3e** (86.2 mg, 0.433 mmol, 1.1 eq.), tetrafluoroterephthalonitrile (**TFTN**, 83.6 mg, 0.418 mmol, 1 eq.), and potassium carbonate (0.156 g, 1.13 mmol, 2.7 eq.) were stirred in anhydrous *N,N*-dimethylformamide (8 mL) at 25 °C overnight. Column chromatography (5x33 cm, DCM) yielded both products as a mixture. This mixture was recrystallized from diethyl ether, yielding product **4e** as a yellow powder (82.2 mg, 0.418 mmol, 55%) in the mother liquor and product **G0** as a yellow powder (35.8 mg, 0.069 mmol, 17%) in the residue.

Characterization of compound **4e**:

M($C_{21}H_{11}F_2N_3O$): 359.34 g/mol.

^{1}H-NMR (400 MHz, $CDCl_3$, 298 K): δ [ppm] = 7.49 (m, 2H, *H*8), 7.35 (m, 2H, *H*9), 7.25 (m, 1H, *H*10, overlapping with chloroform signal), 7.12 (m, 2H, *H*5, *H*6), 7.02 (m, 1H, *H*4), 2.25 (s, 3H, *H*3a).

^{13}C{^{1}H}-NMR (101 MHz, $CDCl_3$, 298 K): δ [ppm] = 151.4 (t, $^3J_{C\text{-}F}$ = 2.7 Hz, *C*12), 150.2 (*C*1), 147.9 (*C*7), 147.9 (dd, $^1J_{C\text{-}F}$ = 260.0 Hz, $^2J_{C\text{-}F}$ = 13.6 Hz, *C*16/*C*15), 147.4 (dd, $^1J_{C\text{-}F}$ = 260.0 Hz, $^2J_{C\text{-}F}$ = 12.7 Hz, *C*15/*C*16), 137.0 (d, $^3J_{C\text{-}F}$ = 4.3 Hz, *C*11), 135.2 (*C*3), 132.0 (*C*2), 130.0 (*C*9), 128.5 (*C*4), 127.4 (*C*10), 127.2 (*C*5), 127.1 (*C*8), 115.1 (*C*6), 110.3 (d, $^3J_{C\text{-}F}$ = 3.9 Hz, *C*18/*C*14), 108.4 (d, $^3J_{C\text{-}F}$ = 3.8 Hz,

$C$14/$C$18), 103.8 (dd, $^2J_{C\text{-}F}$ = 13.8 Hz, $^3J_{C\text{-}F}$ = 2.5 Hz, $C$17/$C$13), 97.3 (dd, $^2J_{C\text{-}F}$ = 15.6 Hz, $^3J_{C\text{-}F}$ = 2.2 Hz, $C$13/$C$17), 18.1 (C3a).

^{19}F-NMR (376 MHz, CDCl$_3$, 298 K): δ [ppm] = -131.1 (d, $^3J_{F\text{-}F}$, 20.6 Hz), -133.5 (d, $^3J_{F\text{-}F}$, 20.6 Hz).

HR-MS (ESI-pos, 70 eV): *m/z* = 360.0948 [M + H]$^+$, calculated for [$C_{21}H_{11}F_2N_3O$ + H]$^+$ = 360.0943; 382.0767 [M + Na]$^+$, calculated for [$C_{21}H_{11}F_2N_3O$ + Na]$^+$ = 382.0762.

IR: $\tilde{\nu}$ [cm^{-1}] = 3069, 2956, 2921, 2853, 2320, 2240, 1636, 1590, 1469, 1447, 1382, 1310, 1257, 1214, 1190, 1137, 1092, 1076, 1037, 1000, 962, 932, 885, 783, 766, 752, 732, 700, 633, 611, 539, 479, 414.

Melting point: 192 °C.

Characterization of compound **G0**:

M($C_{34}H_{22}N_4O_2$): 518.58 g/mol.

^{1}H-NMR (400 MHz, CDCl$_3$, 298 K): δ [ppm] = 7.42 (m, 4H, $H$8), 7.31 (m, 4H, $H$9), 7.17 (tt, $^3J_{H\text{-}H}$ = 7.5 Hz, $^4J_{H\text{-}H}$ = 1.0 Hz 2H, $H$10), 7.10 (m, 4H, $H$5, $H$6), 6.99 (m, 2H, $H$4), 2.26 (s, 3H, H3a).

^{13}C{^{1}H}-NMR (101 MHz, CDCl$_3$, 298 K): δ [ppm] = 151.6 ($C$12), 151.3 ($C$1), 148.0 ($C$7), 137.0 ($C$11), 135.3 ($C$3/$C$2), 132.4 ($C$2/$C$3), 129.7 ($C$9), 127.7 ($C$4), 126.9 ($C$5), 126.4 ($C$10), 125.5 ($C$8), 115.1 ($C$6), 112.4 ($C$14), 103.4 ($C$13), 18.0 (C3a).

HR-MS (ESI-pos, 70 eV): *m/z* = 519.1822 [M + H]$^+$, calculated for [$C_{34}H_{22}N_4O_2$ + H]$^+$ = 519.1816; 541.1646 [M + Na]$^+$, calculated for [$C_{34}H_{22}N_4O_2$ + Na]$^+$ = 541.1635.

IR: $\tilde{\nu}$ [cm^{-1}] = 3056, 3025, 2961, 2922, 2236, 1588, 1475, 1455, 1425, 1378, 1296, 1265, 1214, 1176, 1086, 1042, 1000, 959, 919, 882, 785, 765, 751, 733, 699, 628, 555, 529, 489, 441.

Melting point: 364 °C.

Crystals were grown using a concentrated solution of **G0** in CDCl$_3$.

Compound Y8

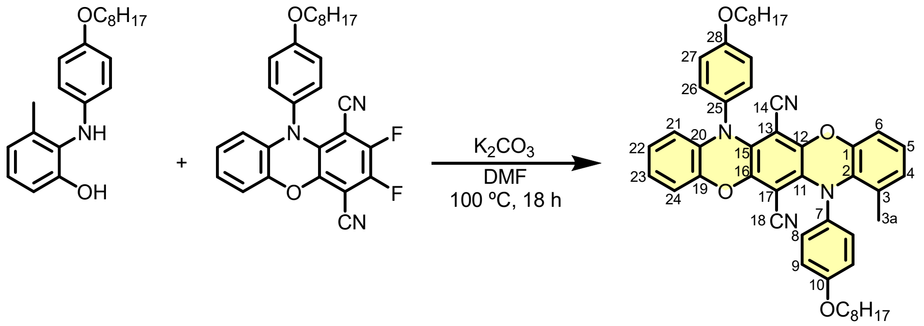

3c **4a** **Y8**

Adopting **GP2**, compound **3c** (76.0 mg, 0.242 mmol, 1 eq.), compound **4a** (113 mg, 0.232 mmol, 1 eq.), and potassium carbonate (50.0 mg, 0.362 mmol, 1.6 eq.) were stirred in anhydrous *N,N*-dimethylformamide (10 mL) at 100 °C overnight. Column chromatography (4.7x30 cm, DCM/*n*-pentane 1/1 → 3/1) yielded product **Y8** as an orange powder (52.8 mg, 0.0891 mmol, 54%) after washing with *n*-pentane.

M($C_{49}H_{52}N_4O_4$): 760.98 g/mol.

^{1}H-NMR (400 MHz, CDCl$_3$, 298 K): δ [ppm] = 7.48 (d, $^3J_{H\text{-}H}$ = 8.9 Hz, 2H, $H$8), 7.29 (d, $^3J_{H\text{-}H}$ = 8.9 Hz, 2H, $H$26), 7.05 (d, $^3J_{H\text{-}H}$ = 8.9 Hz, 2H, $H$27), 6.99 (m, 1H, $H$5), 6.92 (m, 1H, $H$6), 6.88 (d, $^3J_{H\text{-}H}$ = 7.4 Hz, 1H, $H$4), 6.81 (d, $^3J_{H\text{-}H}$ = 8.9 Hz, 2H, $H$9), 6.78 (m, 2H, $H$23, $H$24), 6.69 (m, 1H, $H$22), 6.14 (d, $^3J_{H\text{-}H}$ = 8.2 Hz, 1H, $H$21), 4.02 (t, $^3J_{H\text{-}H}$ = 6.6 Hz, 2H, OCH_2), 3.90 (t, $^3J_{H\text{-}H}$ = 6.5 Hz, 2H, OCH_2), 2.22 (s, 3H, H3a), 1.82 (m, 2H, OCCH_2), 1.73 (m, 2H, OCCH_2), 1.40 (m, 20H, residual CH_2), 0.89 (m, 6H, CH_3).

$^{13}C\{^1H\}$-NMR (101 MHz, $CDCl_3$, 298 K): δ [ppm] = 160.9 (*C*28), 157.8 (*C*10), 150.7 (*C*1), 150.2 (*C*12), 144.2 (*C*16), 142.6 (*C*19), 142.1 (*C*7), 135.1 (*C*11/*C*15), 134.5 (*C*3), 133.6 (*C*2), 132.5 (*C*26), 132.0 (*C*20), 130.7 (*C*15/*C*11), 130.5 (*C*25), 129.5 (*C*8), 127.4 (*C*6), 126.1 (*C*5), 124.8 (*C*22), 124.1 (*C*23), 116.5 (*C*27), 116.0 (*C*24), 115.3 (*C*21), 115.1 (*C*9), 114.9 (*C*4), 112.9 (*C*14/*C*18), 110.4 (*C*18/*C*14), 100.8 (*C*13/*C*17), 91.3 (*C*17/*C*13), 68.6 (OCH_2), 68.4 (OCH_2), 32.0 (CH_2), 32.0 (CH_2), 29.5 (CH_2), 29.5 (CH_2), 29.4 (CH_2), 29.4 (CH_2), 29.3 (CH_2), 29.3 (CH_2), 26.2 (2 CH_2), 22.8 (CH_2), 22.8 (CH_2), 18.1 (*C*3a), 14.3 (CH_3), 14.2 (CH_3).

HR-MS (ESI-pos, 70 eV): *m/z* = 761.4039 $[M + H]^+$, calculated for $[C_{49}H_{52}N_4O_4 + H]^+$ = 761.4061; 783.3866 $[M + Na]^+$, calculated for $[C_{49}H_{52}N_4O_4 + Na]^+$ = 783.3811.

IR: $\tilde{\nu}$ [cm^{-1}] = 2922, 2852, 2236, 2218, 1603, 1581, 1550, 1502, 1452, 1427, 1385, 1314, 1286, 1248, 1224, 1182, 1164, 1127, 1107, 1084, 1045, 1030, 1010, 964, 830, 778, 744, 672, 626, 598, 576, 541, 522, 450, 428.

Melting point: 186 °C.

Compound Y2

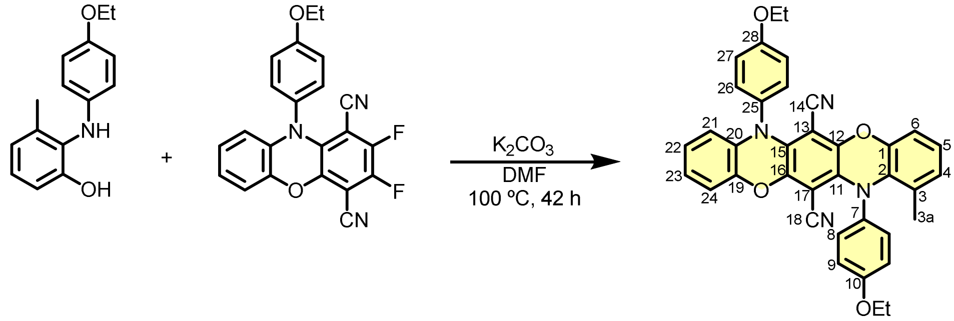

3d **4b** **Y2**

Adopting **GP2**, compound **3d** (40.0 mg, 0.164 mmol, 1 eq.), compound **4b** (64.0 mg, 0.164 mmol, 1 eq.), and potassium carbonate (0.180 g, 1.30 mmol, 7.9 eq.) were stirred in anhydrous *N,N*-dimethylformamide (10 mL) at 100 °C overnight. Column chromatography (5x35 cm, DCM) yielded product **Y2** as an orange powder (52.8 mg, 0.0891 mmol, 54%) after washing with cyclohexane.

M($C_{37}H_{28}N_4O_4$): 592.66 g/mol.

^1H-NMR (600 MHz, CD_2Cl_2, 298 K): δ [ppm] = 7.44 (d, $^3J_{H\text{-}H}$ = 8.9 Hz, 2H, *H*8), 7.31 (d, $^3J_{H\text{-}H}$ = 8.9 Hz, 2H, *H*26), 7.06 (d, $^3J_{H\text{-}H}$ = 8.9 Hz, 2H, *H*27), 7.02 (m, 1H, *H*5), 6.94 (m, 2H, *H*4, *H*6), 6.82 (d, $^3J_{H\text{-}H}$ = 8.9 Hz, 2H, *H*9), 6.80 (m, 2H, *H*23, *H*24), 6.72 (m, 1H, *H*22), 6.16 (d, $^3J_{H\text{-}H}$ = 7.9 Hz, 1H, *H*21), 4.12 (q, $^3J_{H\text{-}H}$ = 7.0 Hz, 2H, OCH_2), 3.98 (q, $^3J_{H\text{-}H}$ = 7.0 Hz, 2H, OCH_2), 2.23 (s, 3H, *H*3a), 1.45 (t, $^3J_{H\text{-}H}$ = 7.0 Hz, 3H, OCH_2CH_3), 1.36 (t, $^3J_{H\text{-}H}$ = 7.0 Hz, 3H, OCH_2CH_3).

$^{13}C\{^1H\}$-NMR (151 MHz, CD_2Cl_2, 298 K): δ [ppm] = 161.0 (*C*28), 158.0 (*C*10), 151.3 (*C*1), 150.6 (*C*12), 144.7 (*C*16), 143.1 (*C*19), 142.5 (*C*7), 135.6 (*C*11/*C*15), 135.0 (*C*3), 134.1 (*C*2), 133.1 (*C*26), 132.5 (*C*20), 131.1 (*C*25), 130.8 (*C*15/*C*11), 129.3 (*C*8), 127.7 (*C*4), 126.6 (*C*5), 125.2 (*C*22), 124.4 (*C*23), 116.7 (*C*27), 116.2 (*C*24), 115.8 (*C*21), 115.4 (*C*9), 115.1 (*C*6), 113.1 (*C*14/*C*18), 110.6 (*C*18/*C*14), 101.1 (*C*13/*C*17), 91.9 (*C*17/*C*13), 64.6 (OCH_2), 64.3 (OCH_2), 18.2 (*C*3a), 15.1 (OCCH_3), 15.1 (OCCH_3).

HR-MS (ESI-pos, 70 eV): *m/z* = 593.2171 $[M + H]^+$, calculated for $[C_{37}H_{28}N_4O_4 + H]^+$ = 593.2183; 615.1995 $[M + Na]^+$, calculated for $[C_{37}H_{28}N_4O_4 + Na]^+$ = 615.2003.

IR: $\tilde{\nu}$ [cm^{-1}] = 3060, 3006, 2957, 2919, 2852, 2225, 1729, 1630, 1603, 1584, 1551, 1501, 1453, 1432, 1385, 1311, 1260, 1172, 1110, 1081, 1041, 1009, 966, 919, 833, 813, 751, 673, 624, 594, 540, 518, 446, 424.

Melting point: 247 °C.

Crystals were grown using a concentrated solution of **Y2** in DCM overlayered with *n*-pentane.

NMR SPECTRA

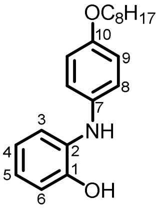

3a

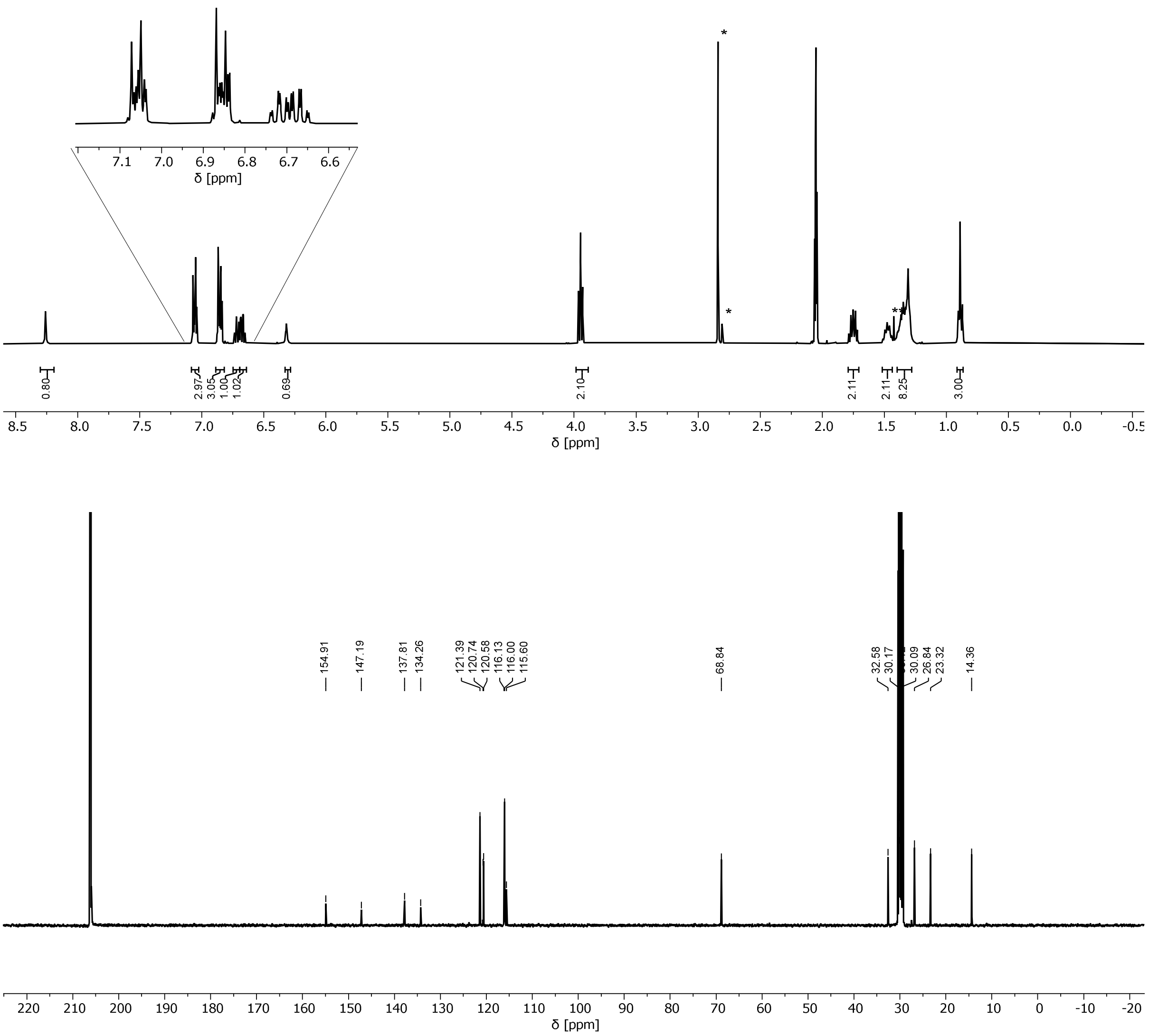

Figure S2: ^{1}H- (top, 400 MHz, acetone-d_6, 298 K) and ^{13}C-NMR spectrum (bottom, 101 MHz, acetone-d_6, 298 K) of compound **3a** (* = water, ** = cyclohexane).

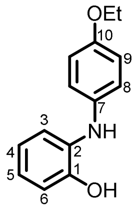

3b

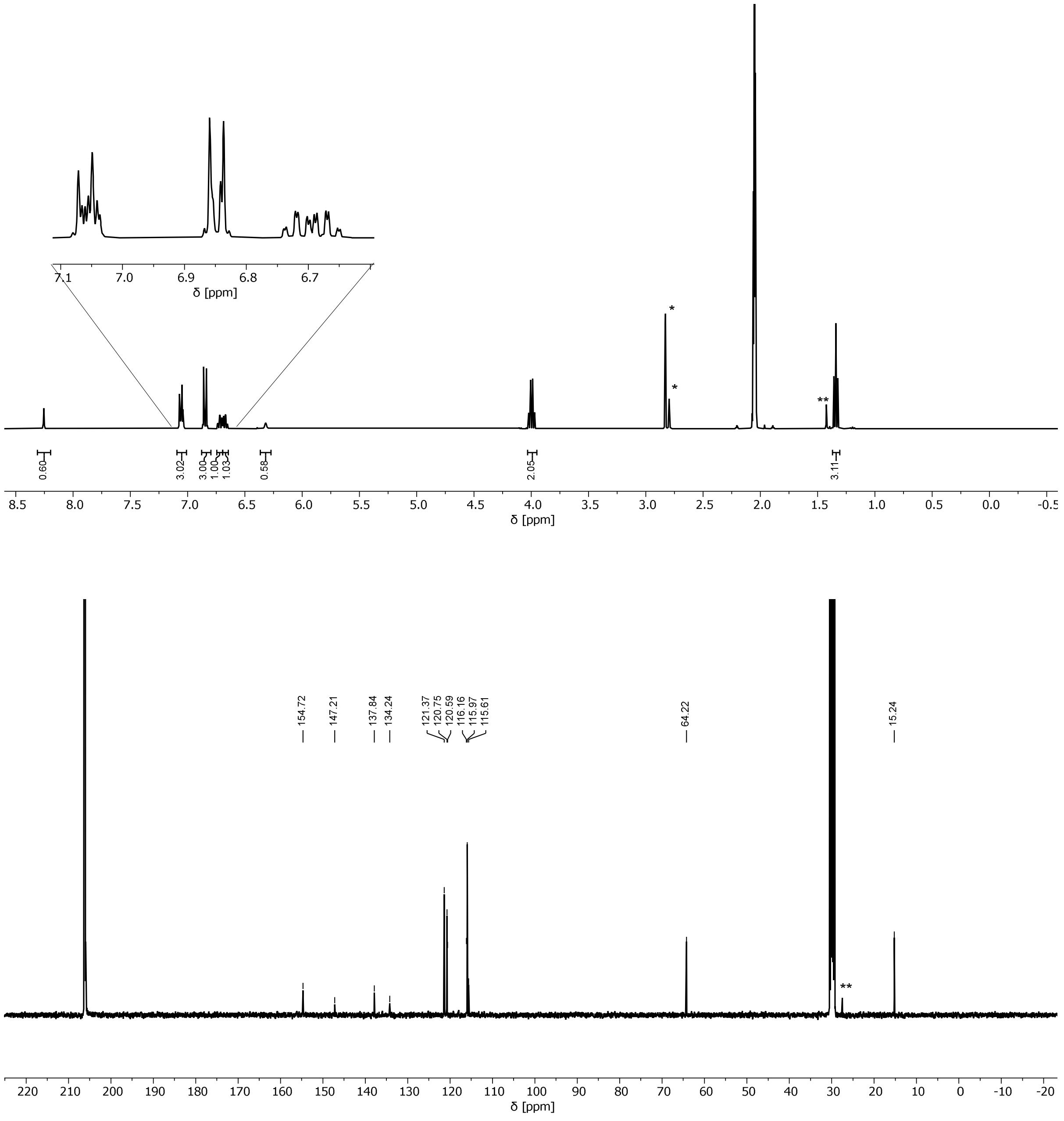

Figure S3: ^{1}H- (top, 400 MHz, acetone-d_6, 298 K) and ^{13}C-NMR spectrum (bottom, 101 MHz, acetone-d_6, 298 K) of compound **3b** (* = water, ** = cyclohexane).

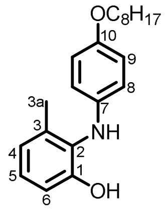

3c

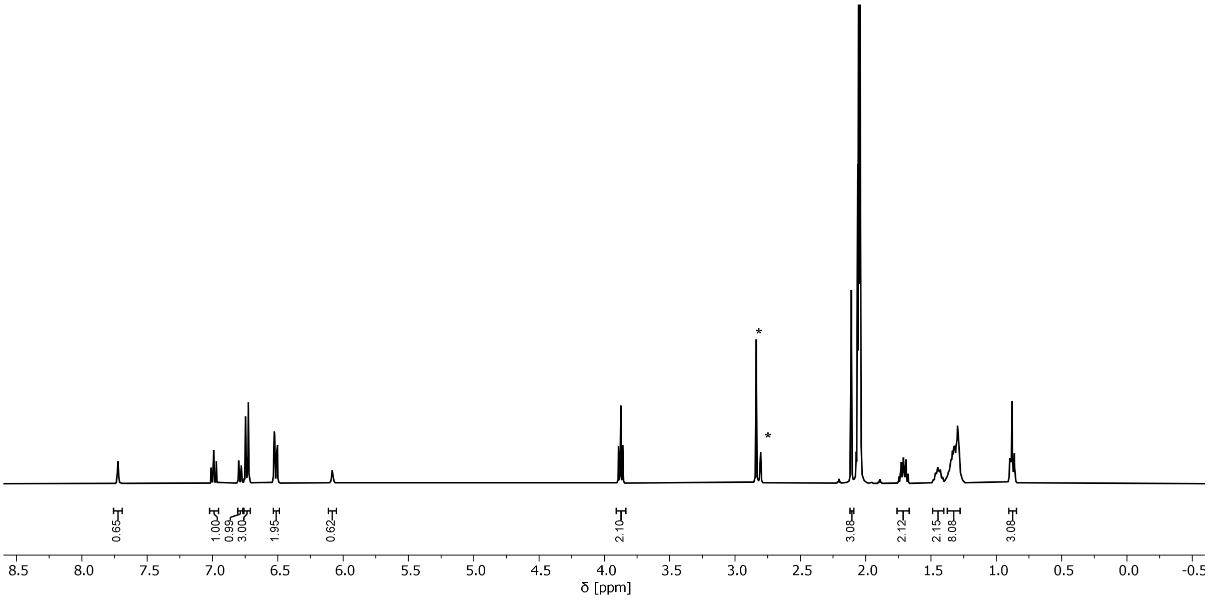

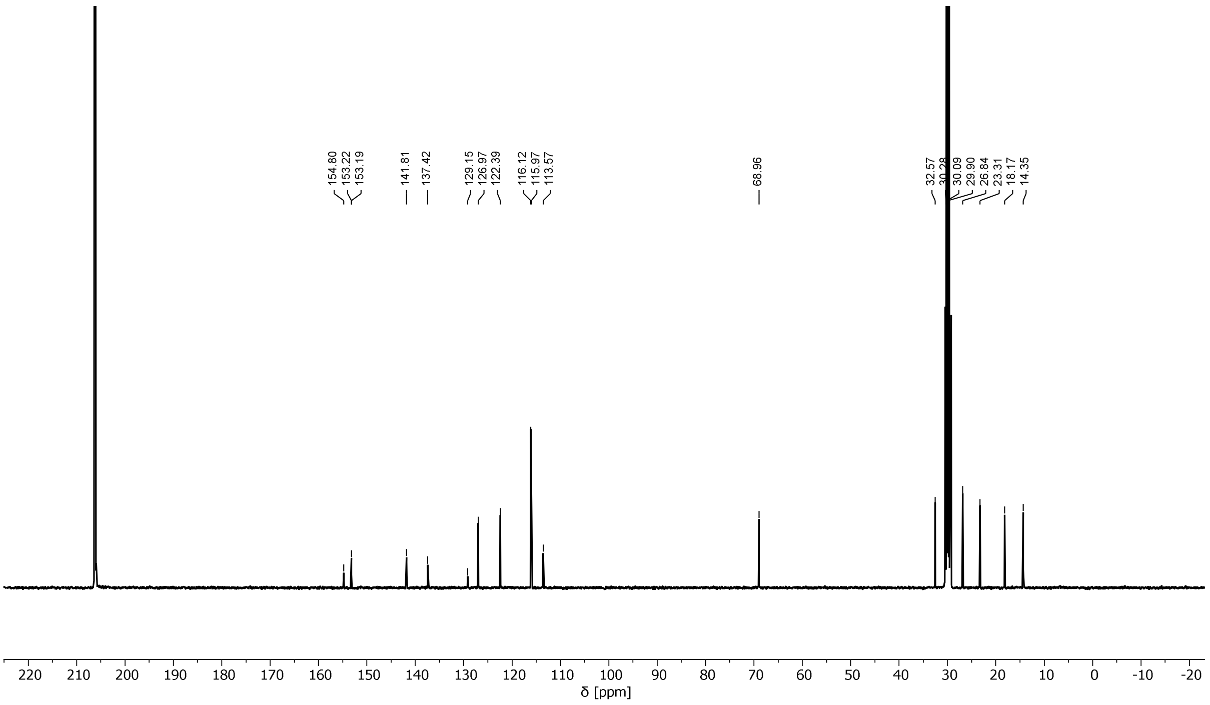

Figure S4: ^{1}H- (top, 400 MHz, acetone-d_6, 298 K) and ^{13}C-NMR spectrum (bottom, 101 MHz, acetone-d_6, 298 K) of compound **3c** (* = water).

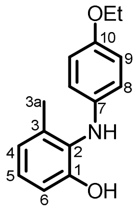

3d

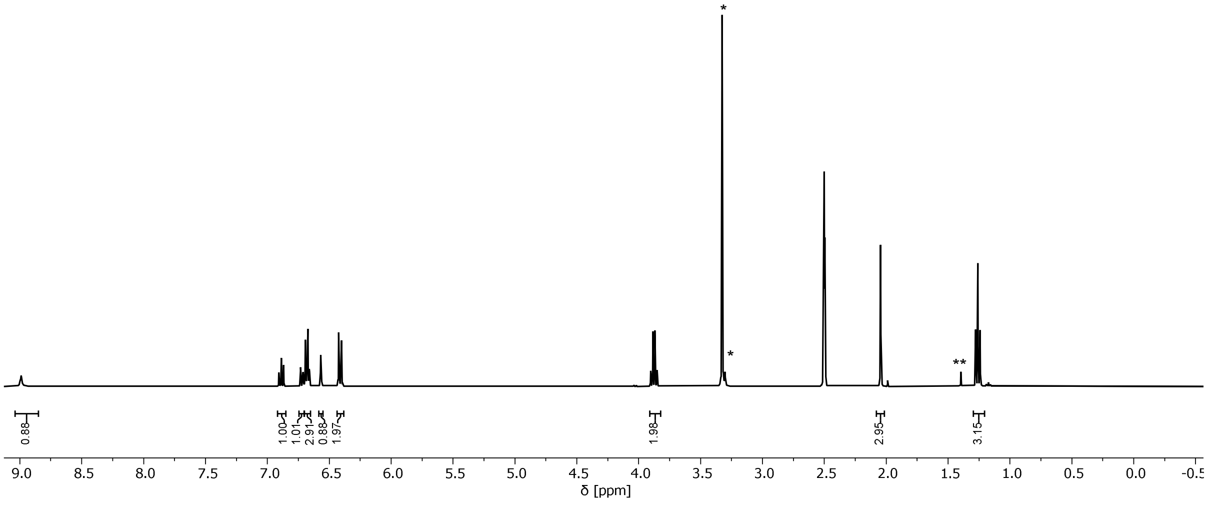

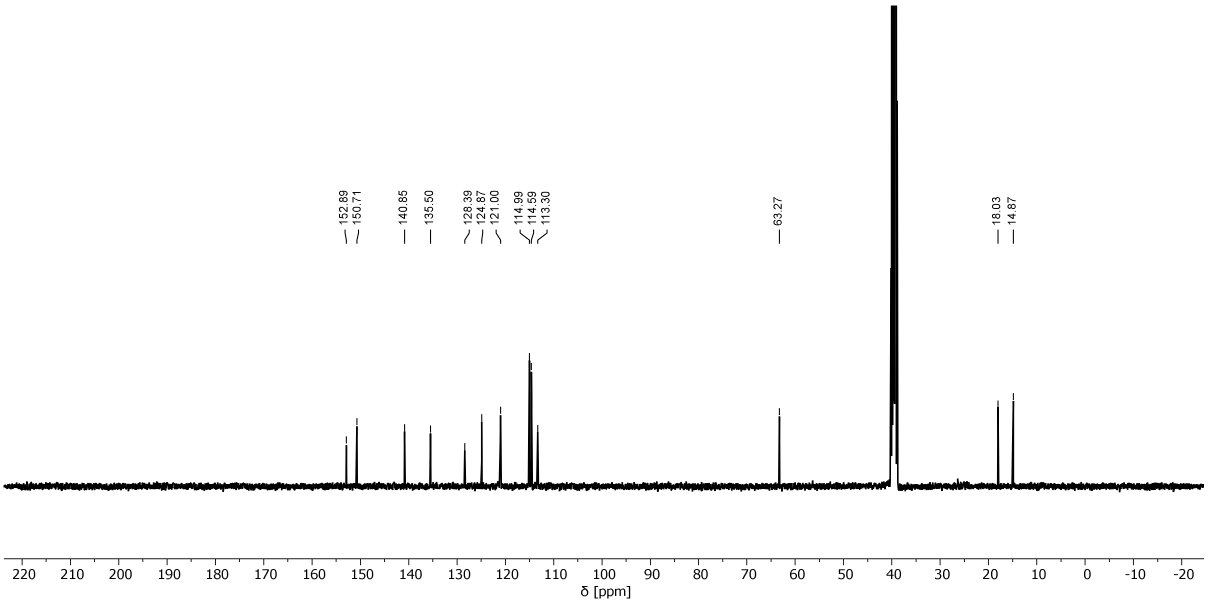

Figure S5: ^{1}H- (top, 400 MHz, DMSO-*d*$_6$, 298 K) and ^{13}C-NMR spectrum (bottom, 101 MHz, DMSO-*d*$_6$, 298 K) of compound **3d** (* = water, ** = cyclohexane).

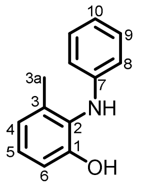

3e

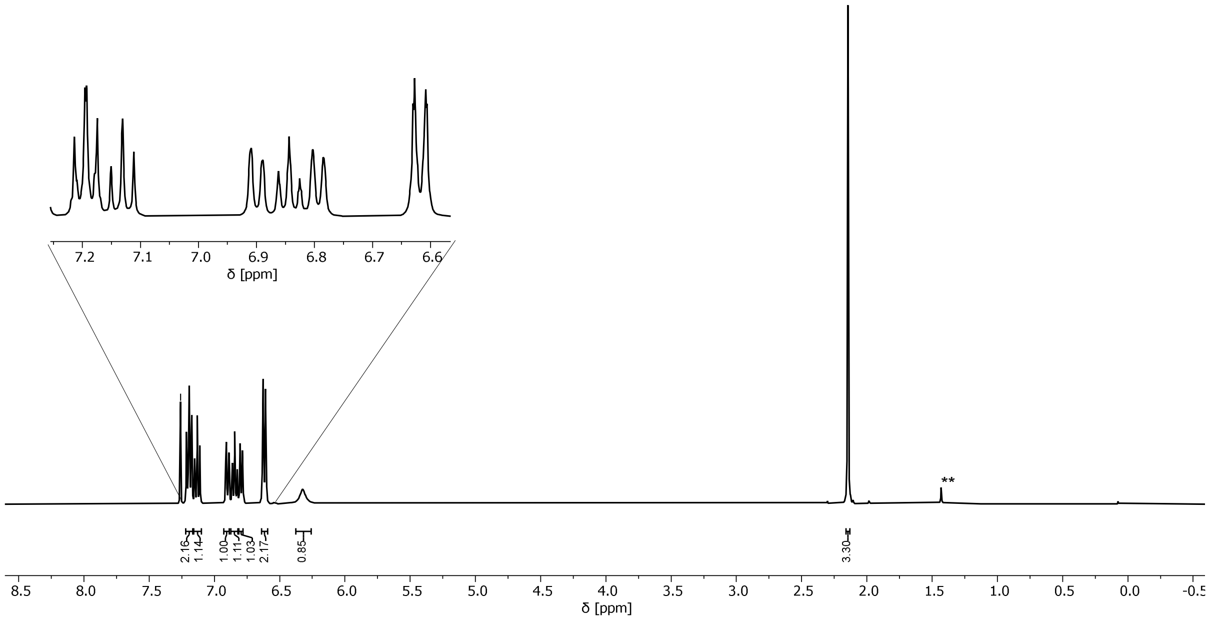

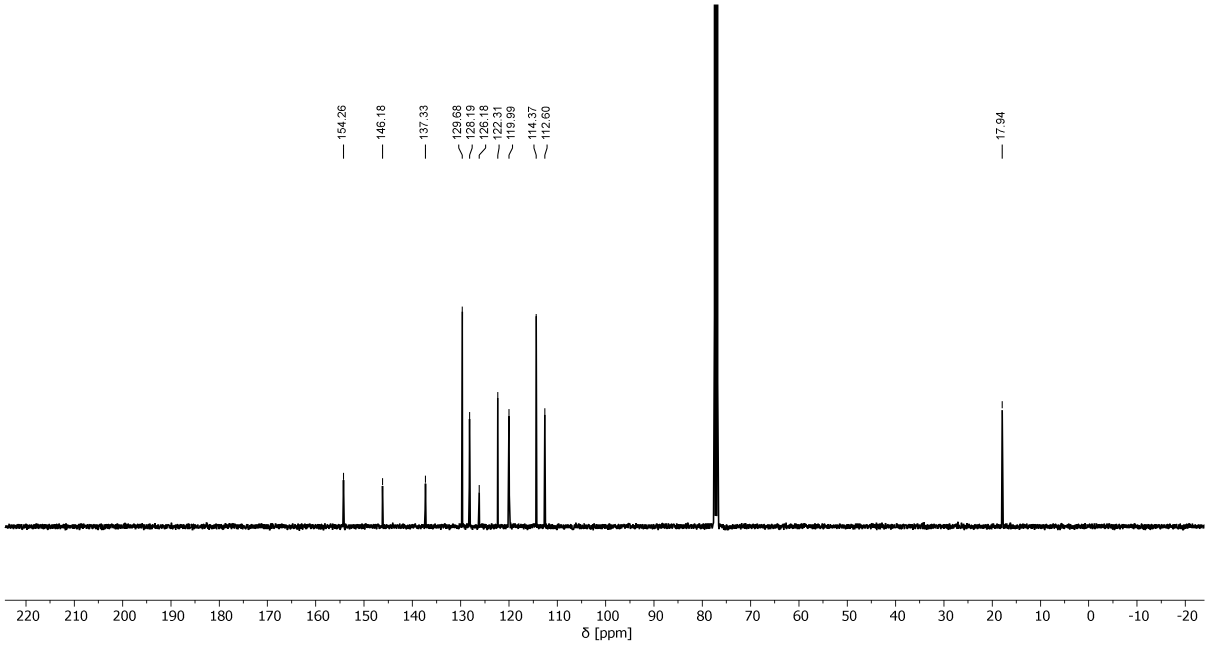

Figure S6: ^{1}H- (top, 400 MHz, $CDCl_3$, 298 K) and ^{13}C-NMR spectrum (bottom, 101 MHz, $CDCl_3$, 298 K) of compound **3e** (** = cyclohexane).

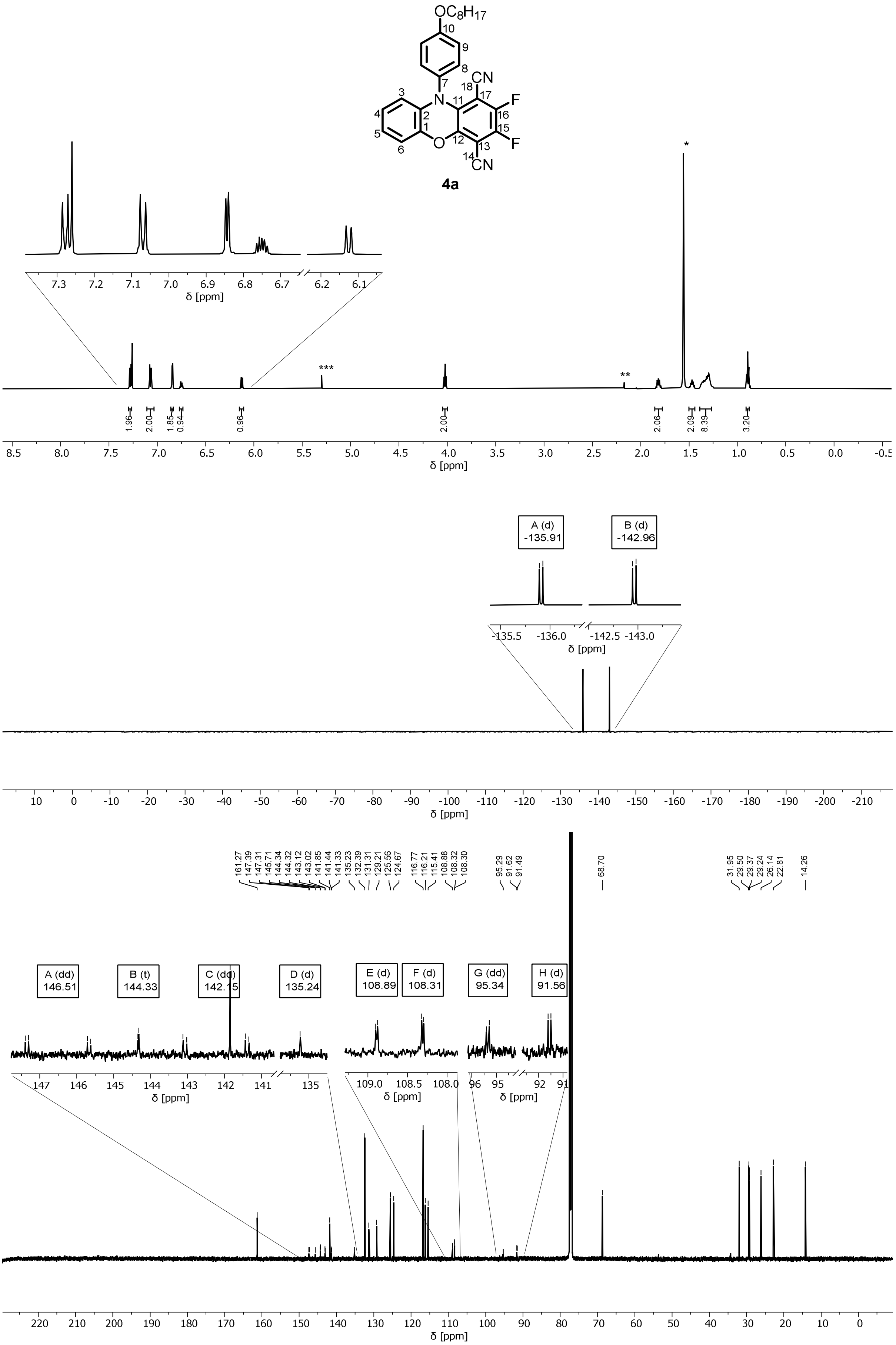

Figure S7: ^{1}H- (top, 600 MHz, $CDCl_3$, 298 K), ^{19}F- (center, 565 MHz, $CDCl_3$, 298 K) and ^{13}C-NMR spectrum (bottom, 151 MHz, $CDCl_3$, 298 K) of compound **4a** (* = water, ** = acetone, *** = dichloromethane).

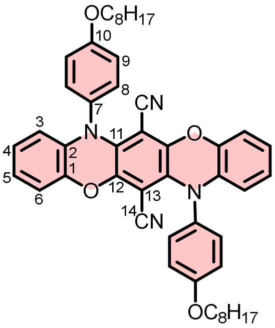

R8

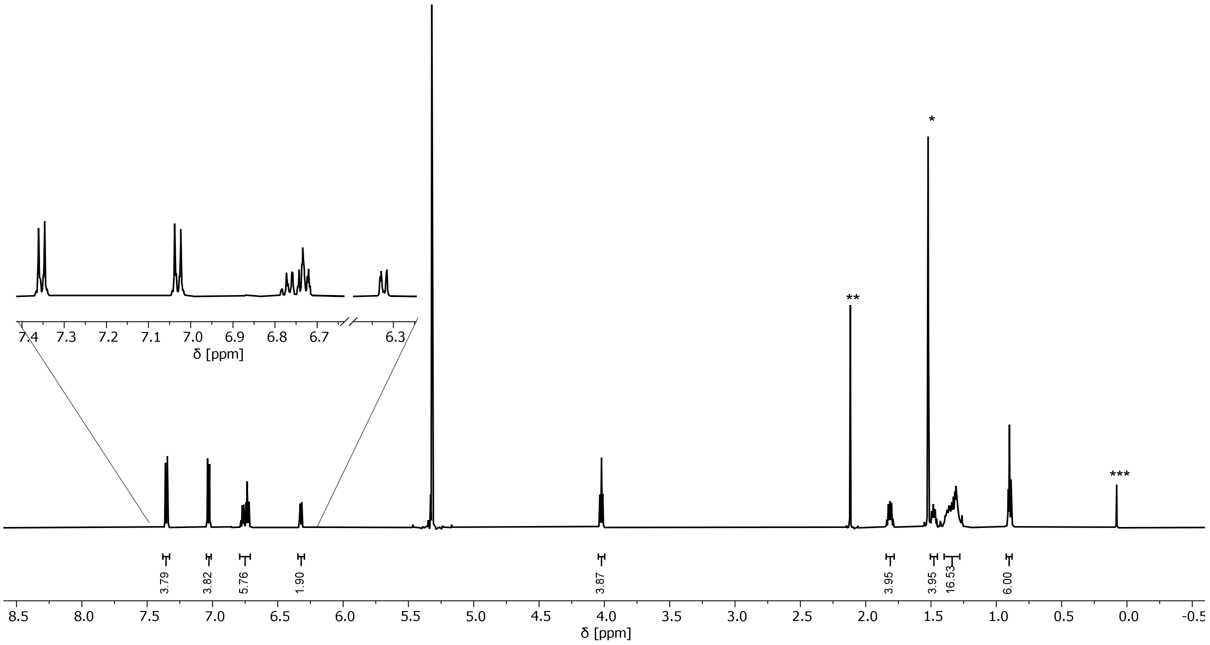

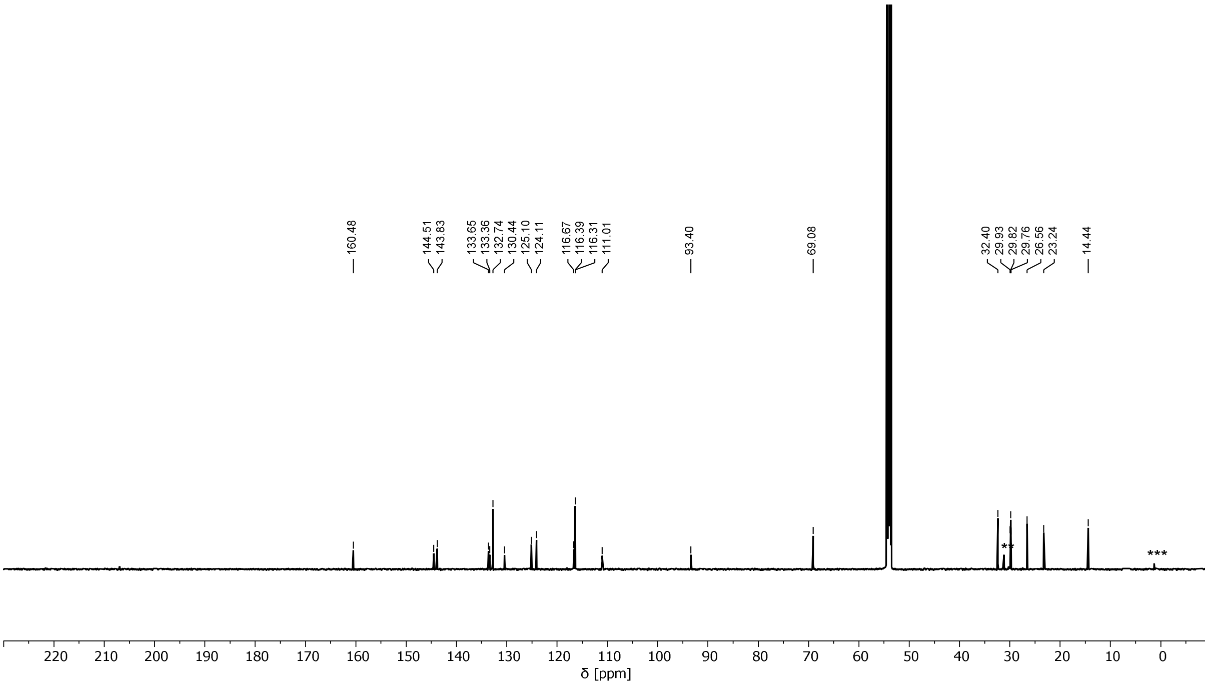

Figure S8: ^{1}H- (top, 600 MHz, CD_2Cl_2, 298 K) and ^{13}C-NMR spectrum (bottom, 151 MHz, CD_2Cl_2, 298 K) of compound **R8** (* = water, ** = acetone, *** = silicon grease).

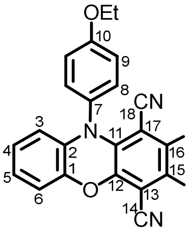

4b

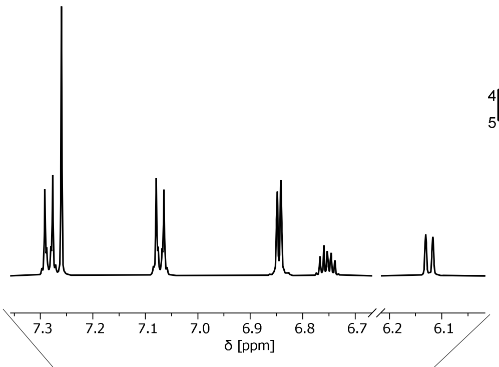

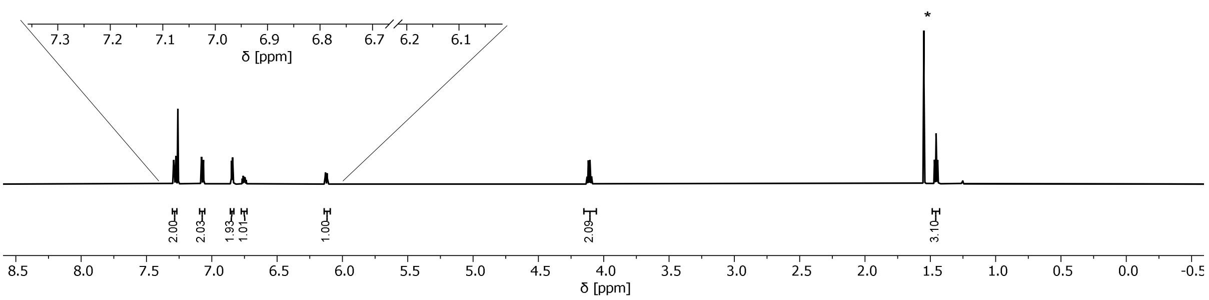

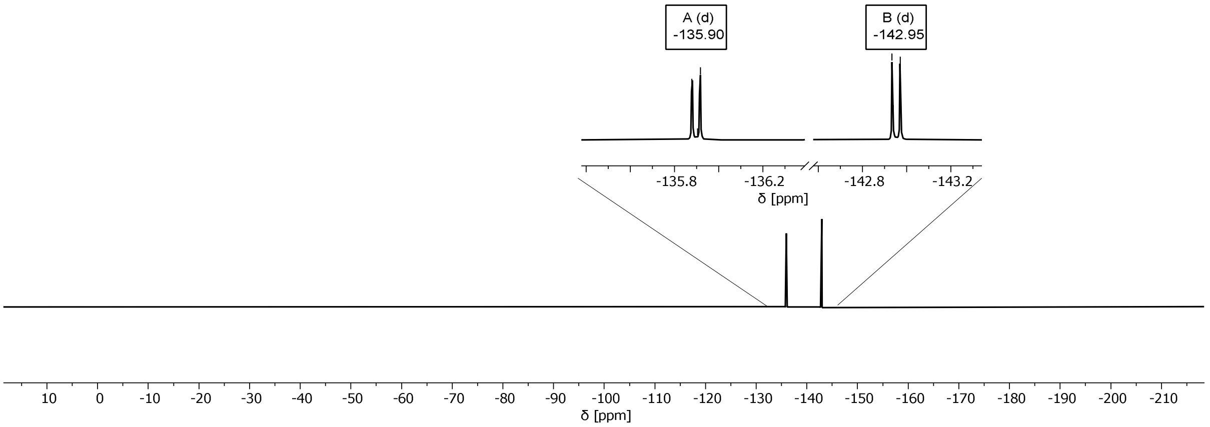

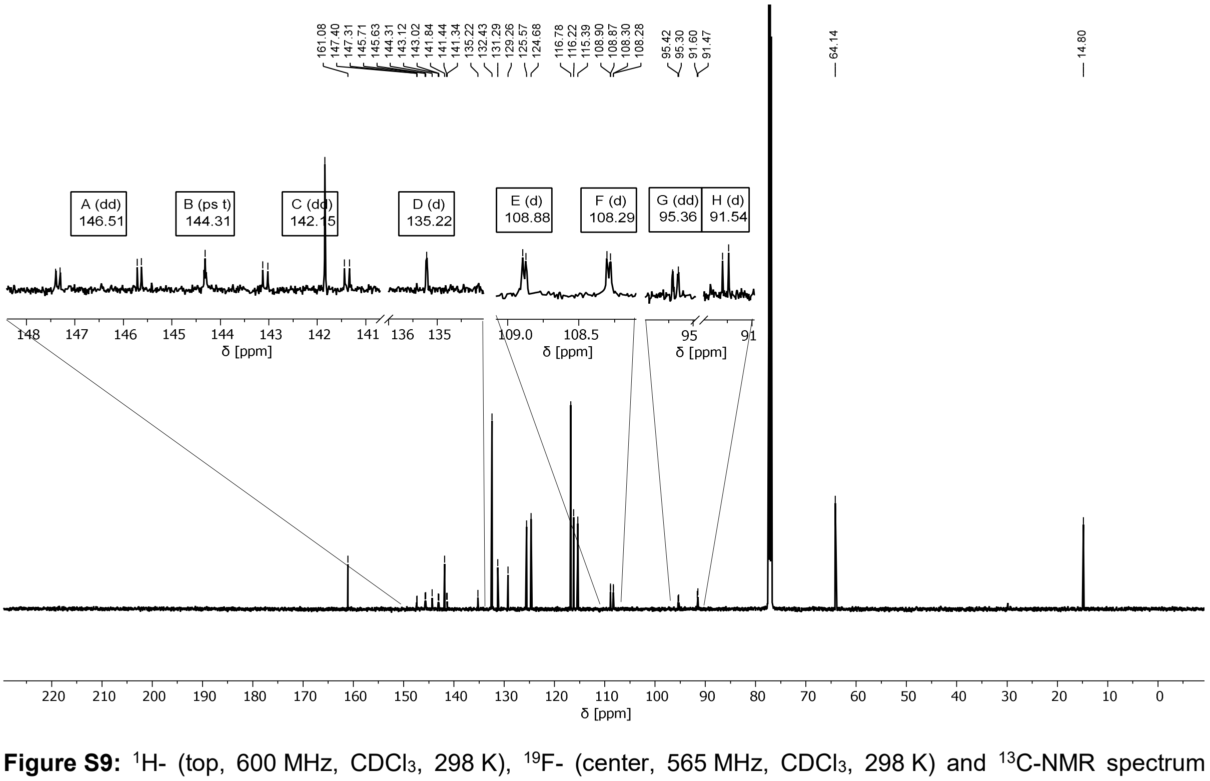

Figure S9: ^{1}H- (top, 600 MHz, $CDCl_3$, 298 K), ^{19}F- (center, 565 MHz, $CDCl_3$, 298 K) and ^{13}C-NMR spectrum (bottom, 151 MHz, $CDCl_3$, 298 K) of compound **4b** (* = water).

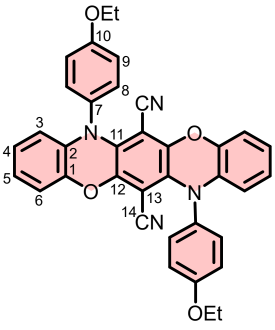

R2

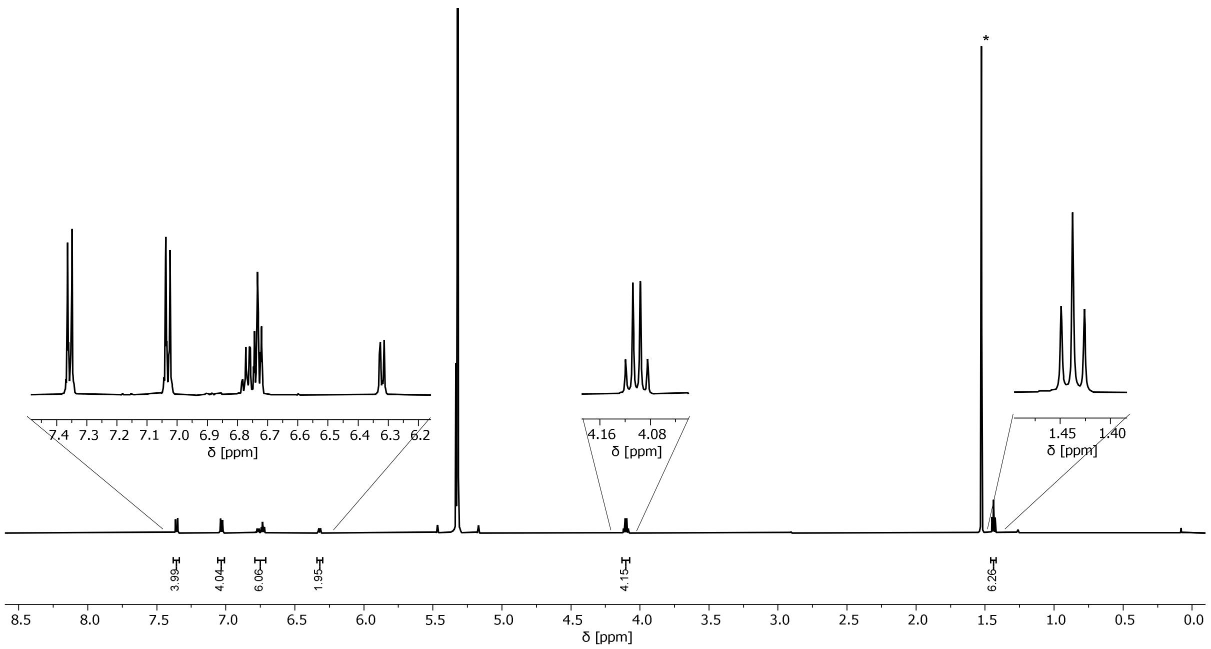

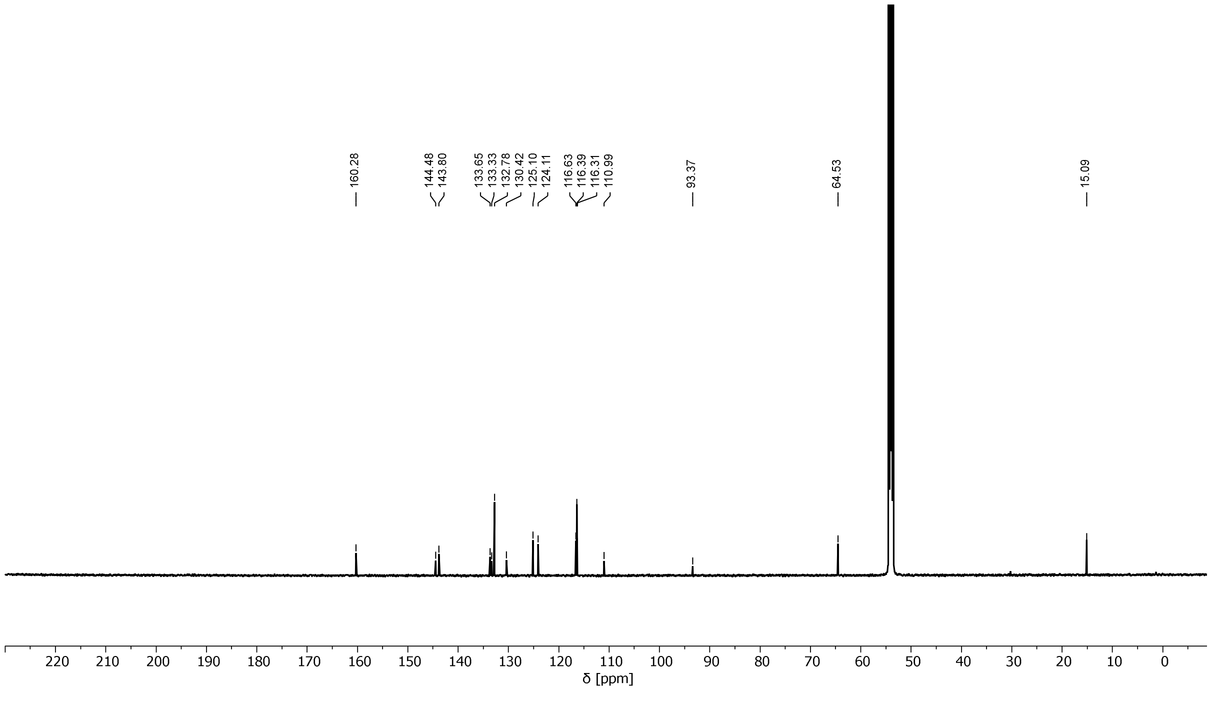

Figure S10: ^{1}H- (top, 600 MHz, CD_2Cl_2, 298 K) and ^{13}C-NMR spectrum (bottom, 151 MHz, CD_2Cl_2, 298 K) of compound **R2** (* = water).

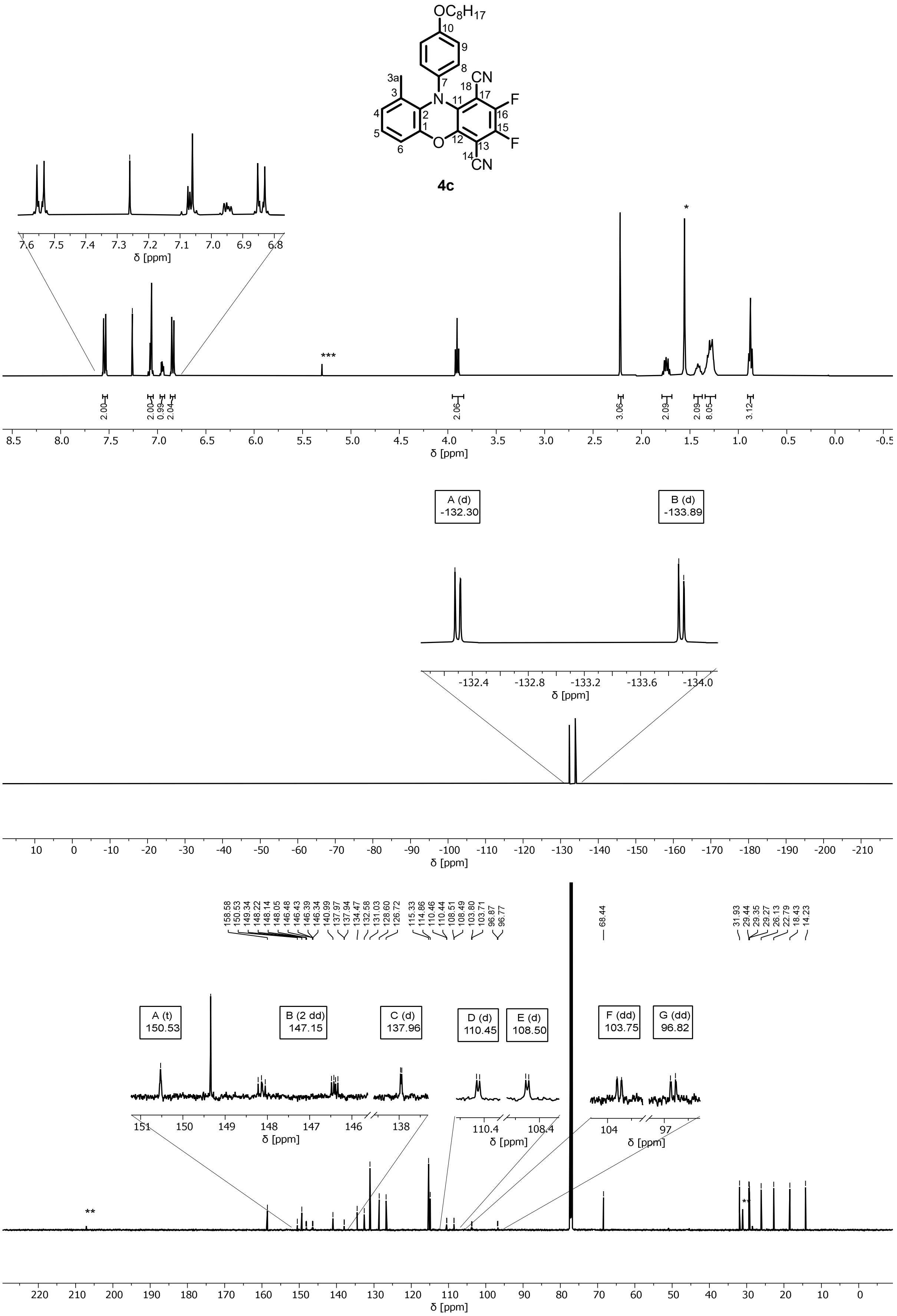

Figure S11: ^{1}H- (top, 600 MHz, $CDCl_3$, 298 K), ^{19}F- (center, 565 MHz, $CDCl_3$, 298 K) and ^{13}C-NMR spectrum (bottom, 151 MHz, $CDCl_3$, 298 K) of compound **4c** (* = water, ** = acetone, *** = dichloromethane).

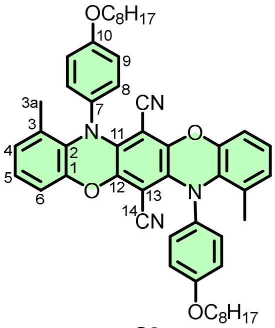

G8

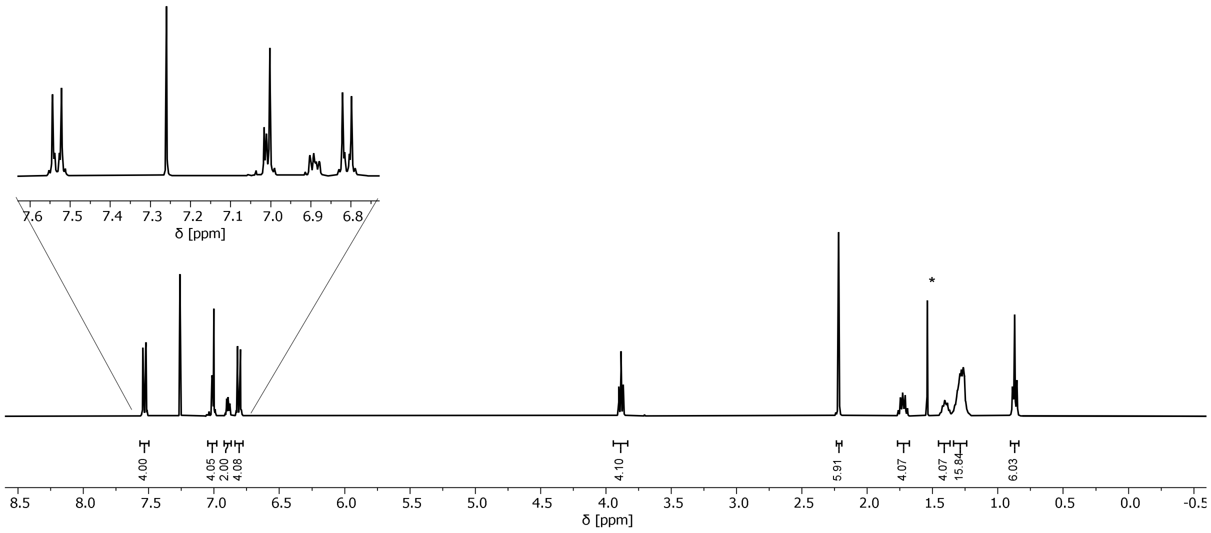

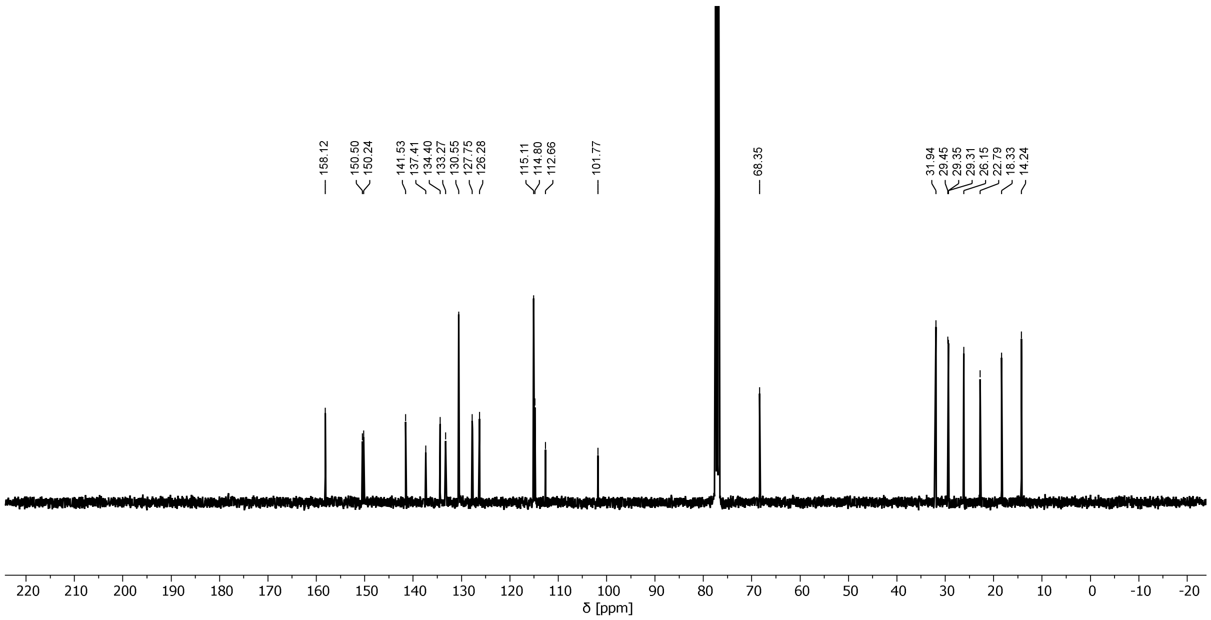

Figure S12: ^{1}H- (top, 400 MHz, $CDCl_3$, 298 K) and ^{13}C-NMR spectrum (bottom, 101 MHz, $CDCl_3$, 298 K) of compound **G8** (* = water).

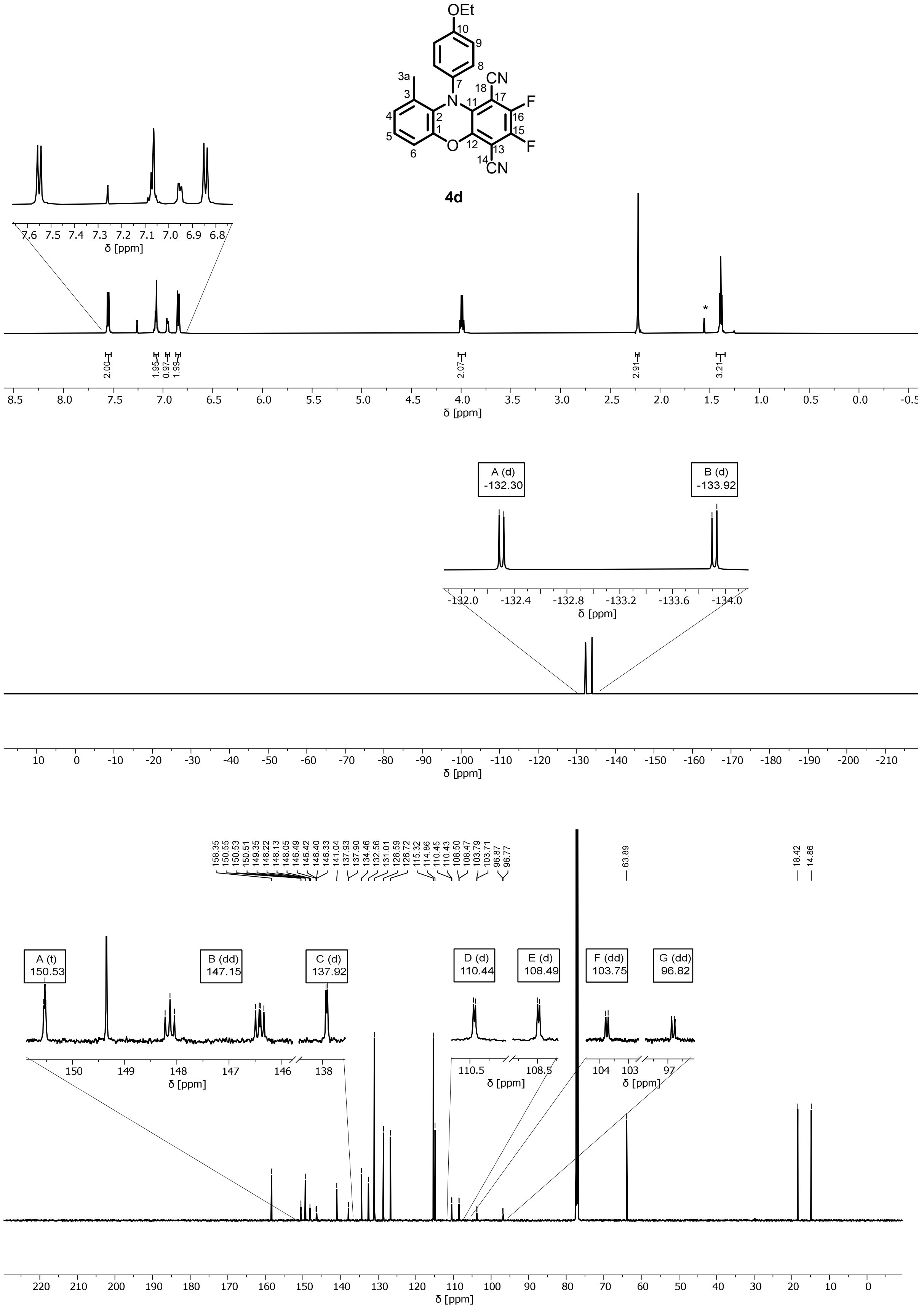

Figure S13: ^{1}H- (top, 600 MHz, $CDCl_3$, 298 K), ^{19}F- (center, 565 MHz, $CDCl_3$, 298 K) and ^{13}C-NMR spectrum (bottom, 151 MHz, $CDCl_3$, 298 K) of compound **4d** (* = water).

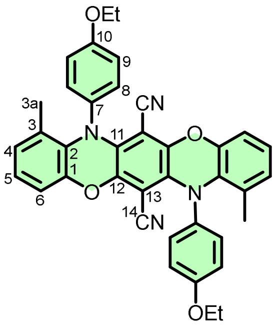

G2

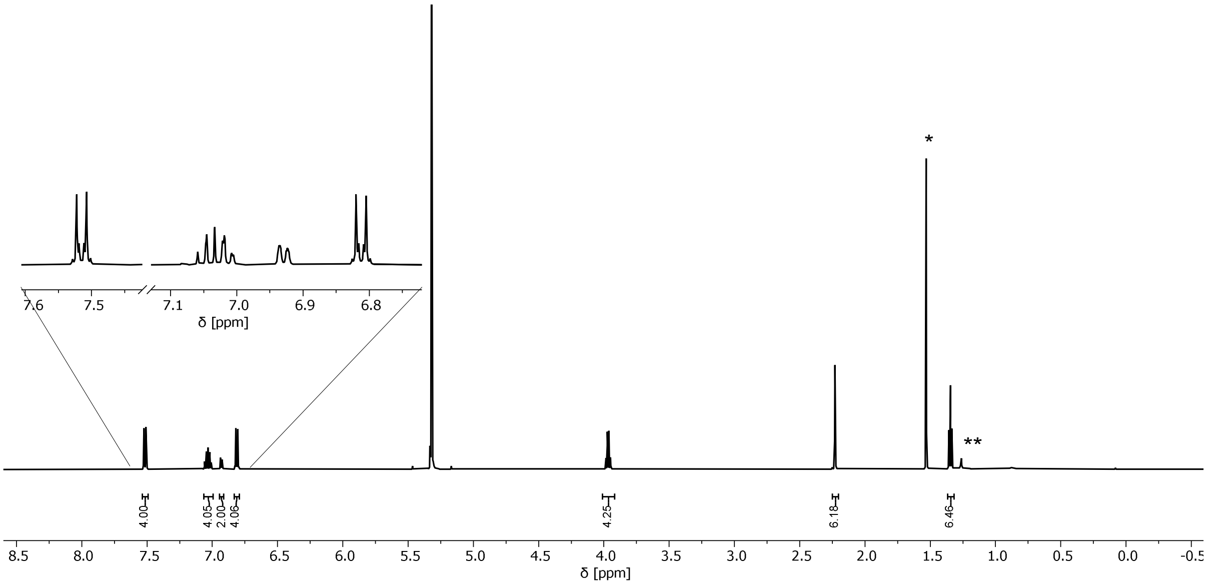

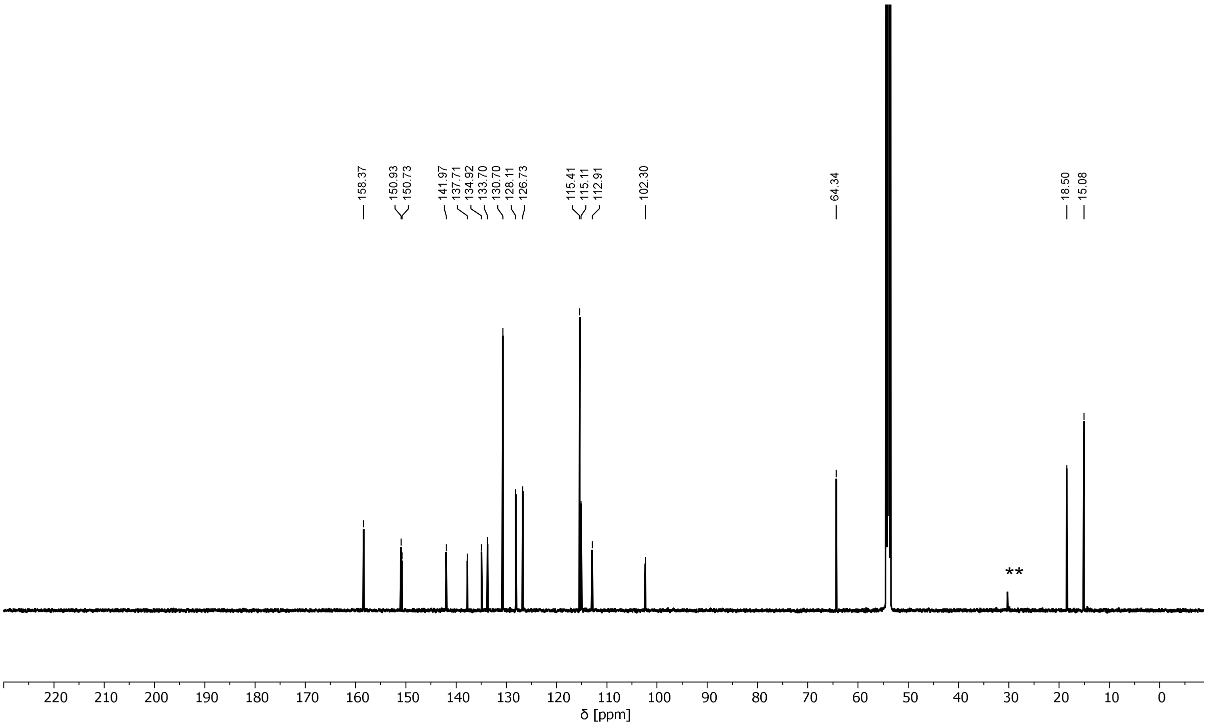

Figure S14: ^{1}H- (top, 600 MHz, CD_2Cl_2, 298 K) and ^{13}C-NMR spectrum (bottom, 151 MHz, CD_2Cl_2, 298 K) of compound **R8** (* = water, ** = H grease.

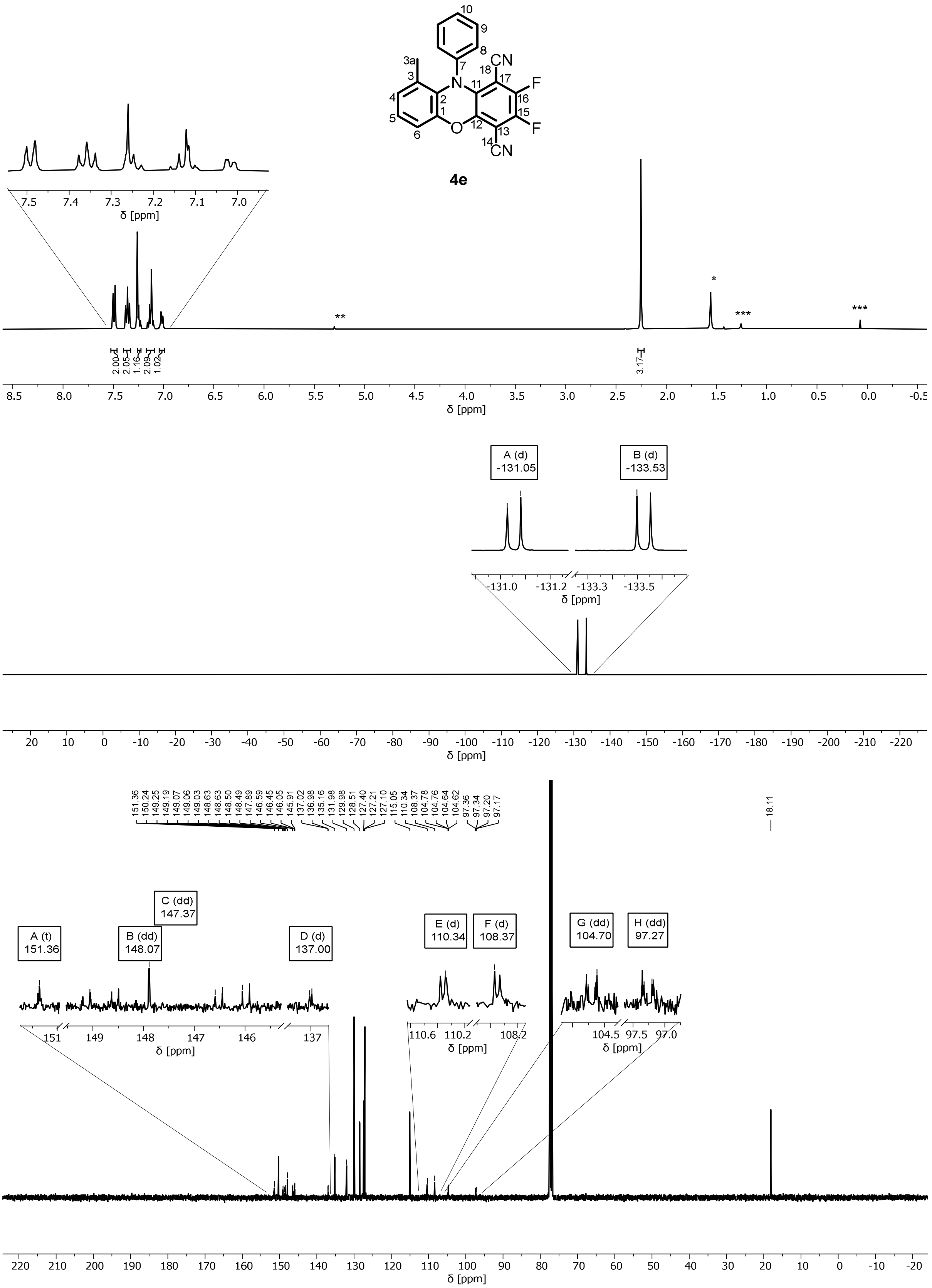

Figure S15: ^{1}H- (top, 400 MHz, $CDCl_3$, 298 K), ^{19}F- (center, 376 MHz, $CDCl_3$, 298 K) and ^{13}C-NMR spectrum (bottom, 101 MHz, $CDCl_3$, 298 K) of compound **4e** (* = water, ** = dichloromethane, *** = grease).

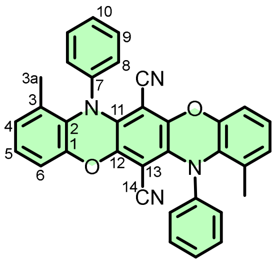

G0

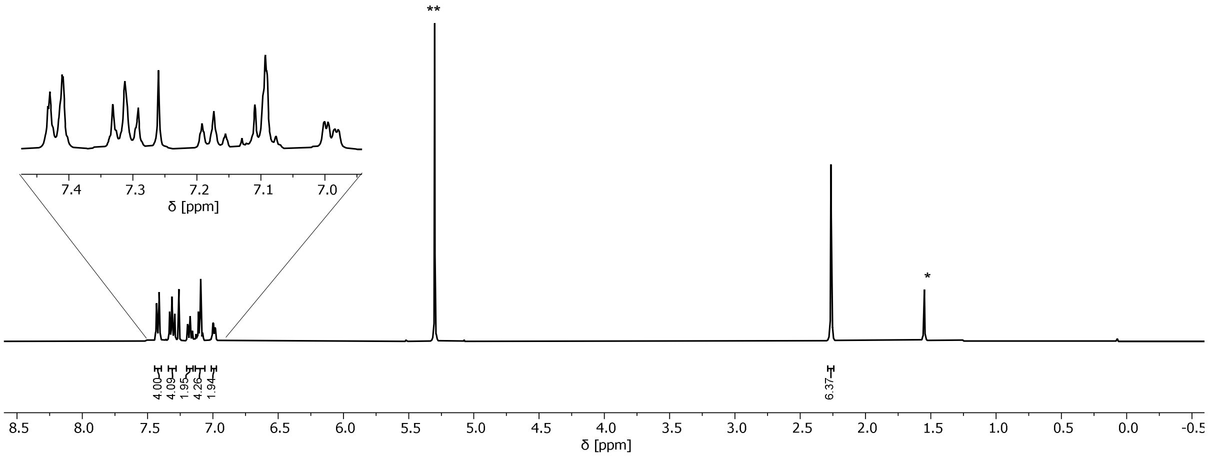

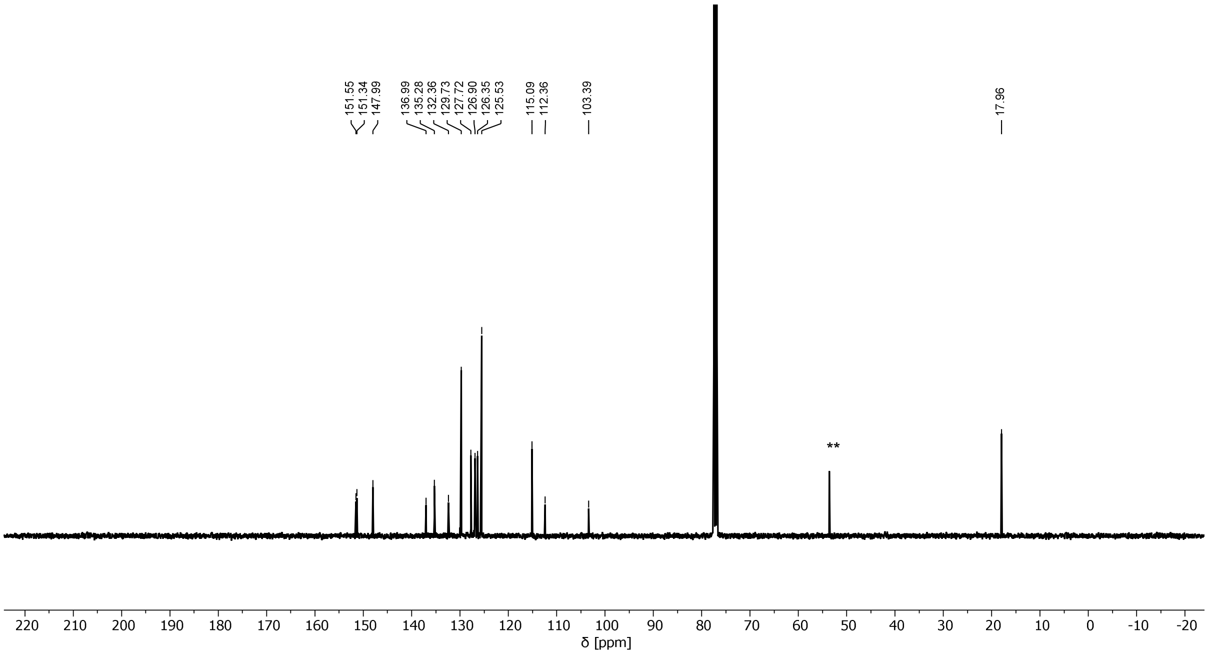

Figure S16: ^{1}H- (top, 400 MHz, $CDCl_3$, 298 K) and ^{13}C-NMR spectrum (bottom, 101 MHz, $CDCl_3$, 298 K) of compound **G0** (* = water, ** = dichloromethane).

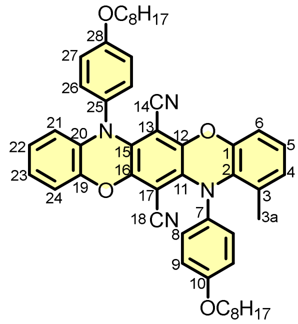

Y8

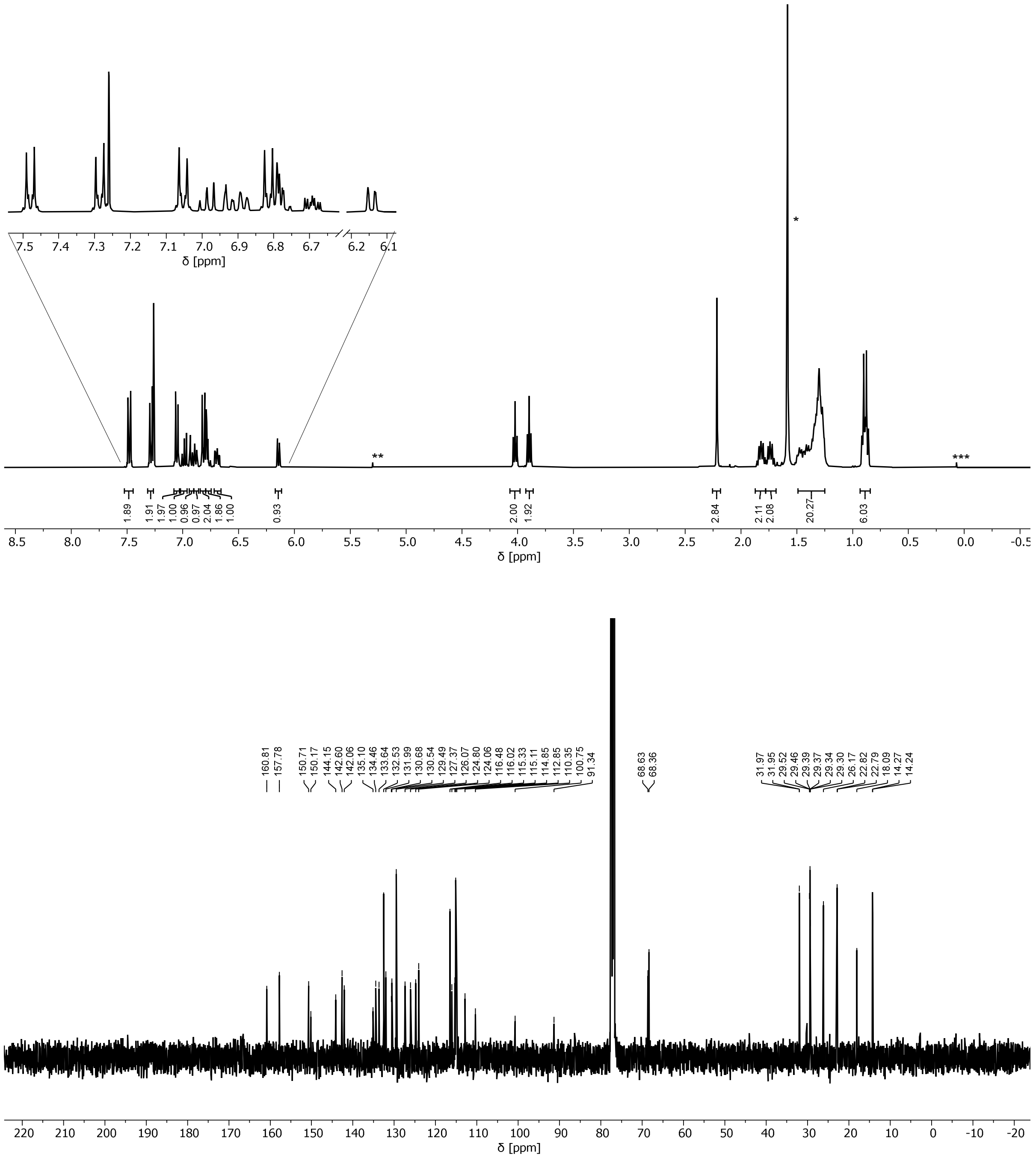

Figure S17: ^{1}H- (top, 400 MHz, $CDCl_3$, 298 K) and ^{13}C-NMR spectrum (bottom, 101 MHz, $CDCl_3$, 298 K) of compound **Y8** (* = water, ** = dichloromethane, *** = silicon grease).

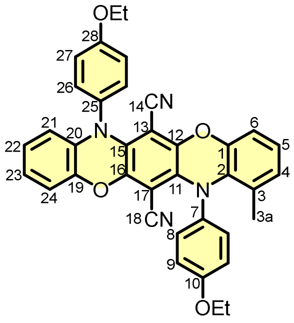

Y2

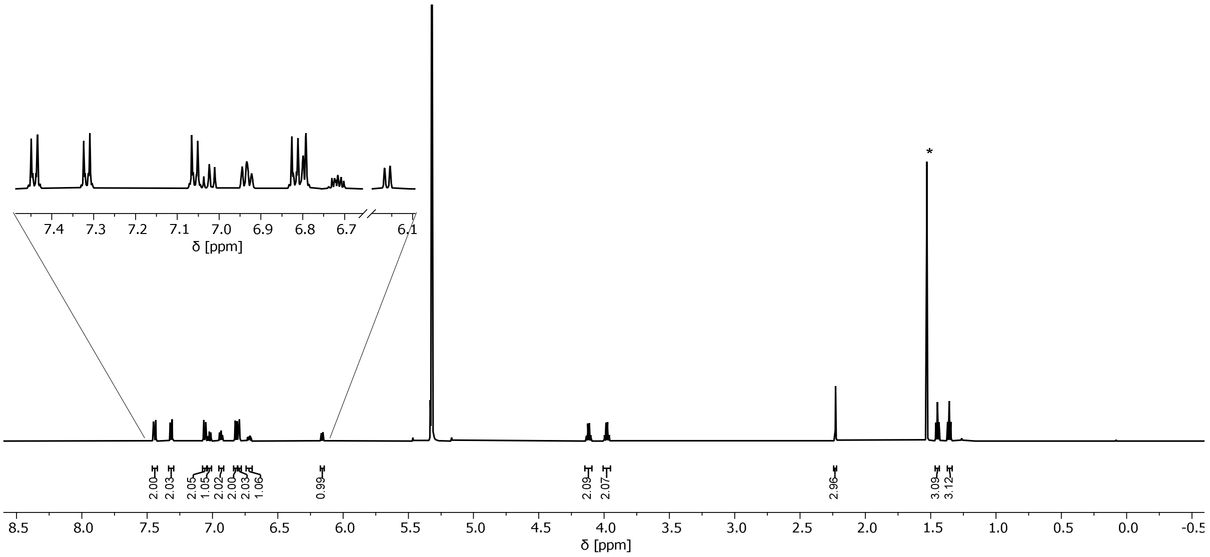

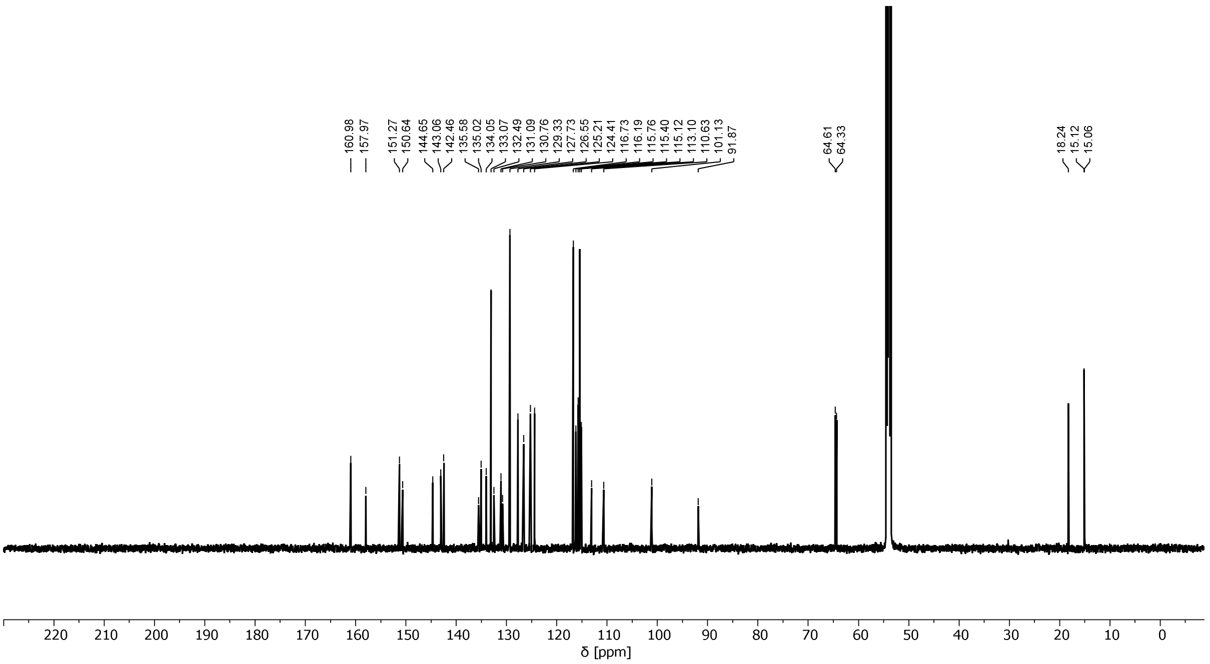

Figure S18: ^{1}H- (top, 600 MHz, CD_2Cl_2, 298 K) and ^{13}C-NMR spectrum (bottom, 151 MHz, CD_2Cl_2, 298 K) of compound **Y2** (* = water).

HPLC CHROMATOGRAMS

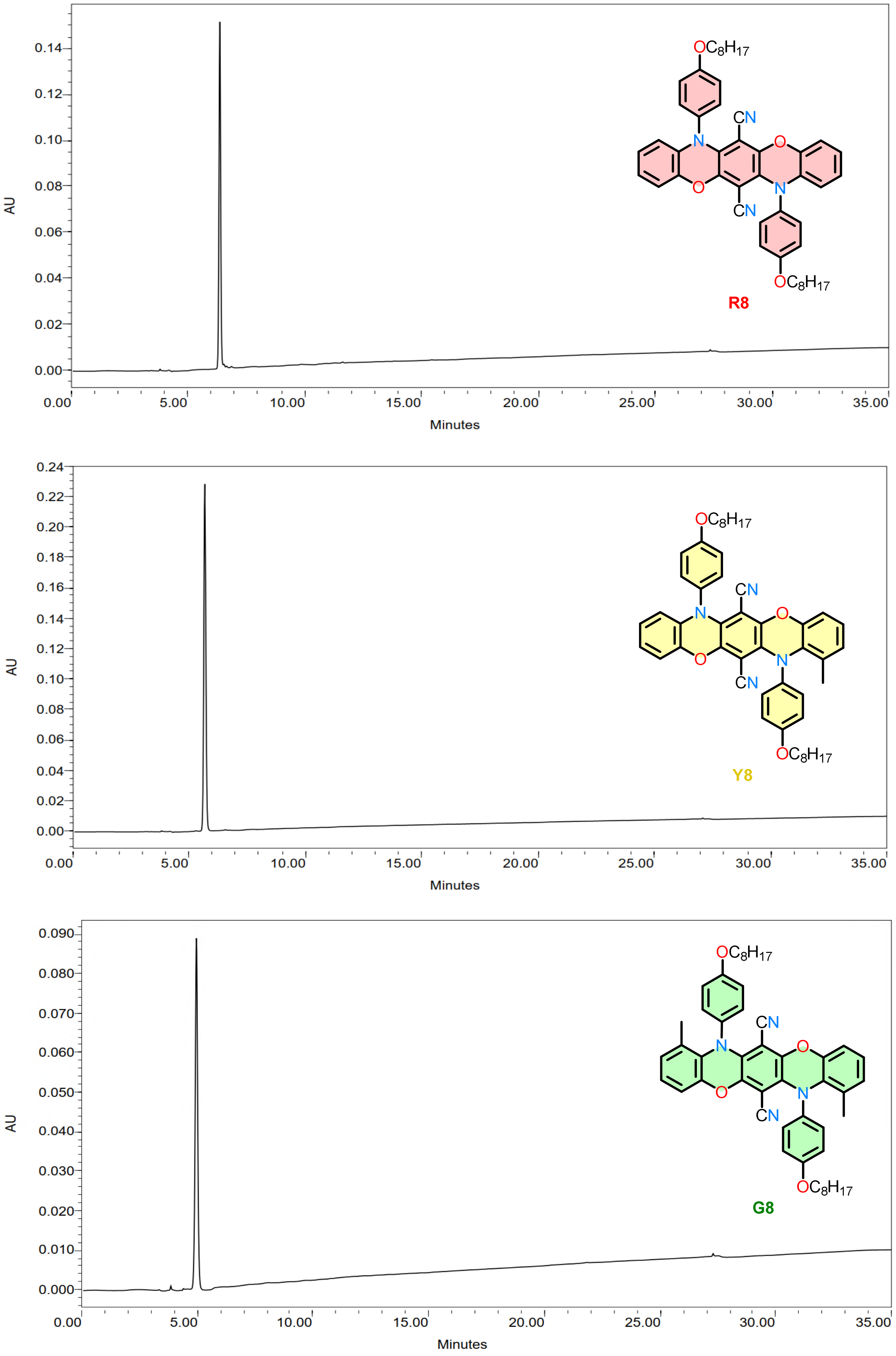

Figure S19: HPLC chromatograms of **R8**, **Y8**, and **G8** (*n*-hexane/ethyl acetate 90/10 → 0/100 over 35 min).

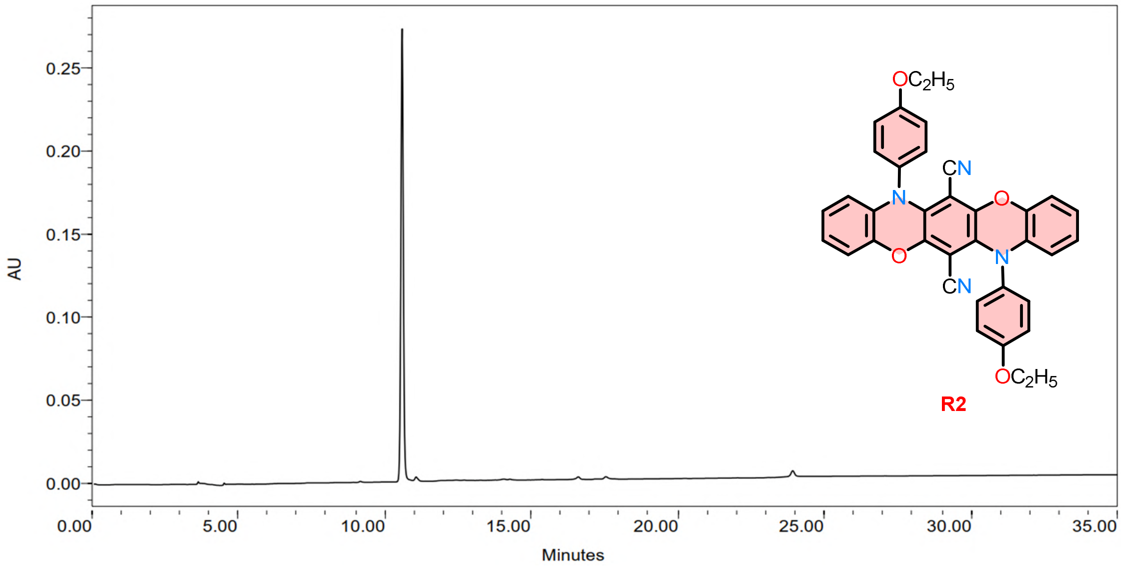

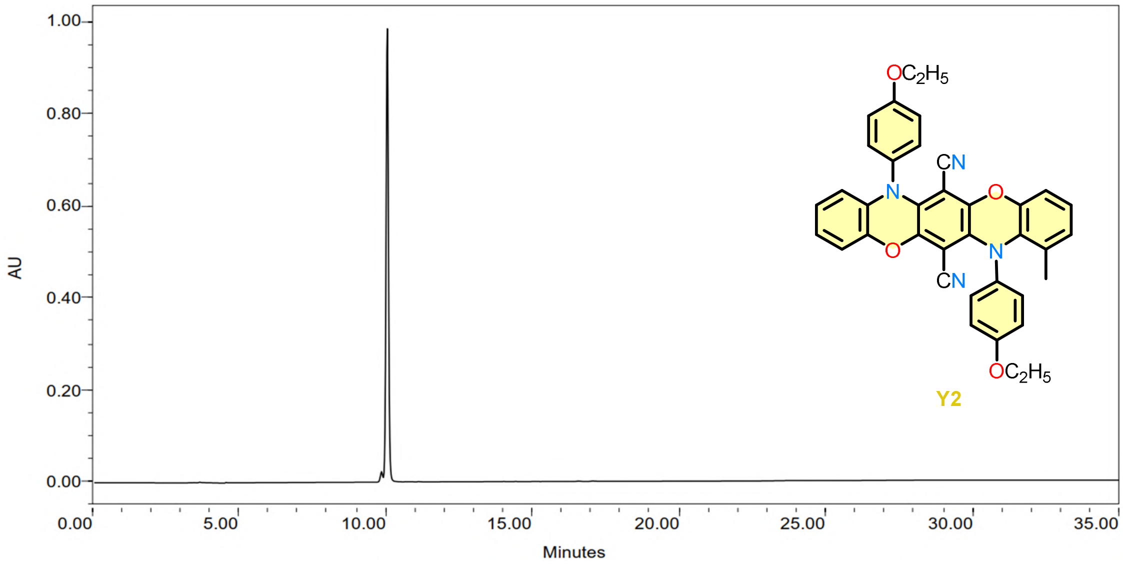

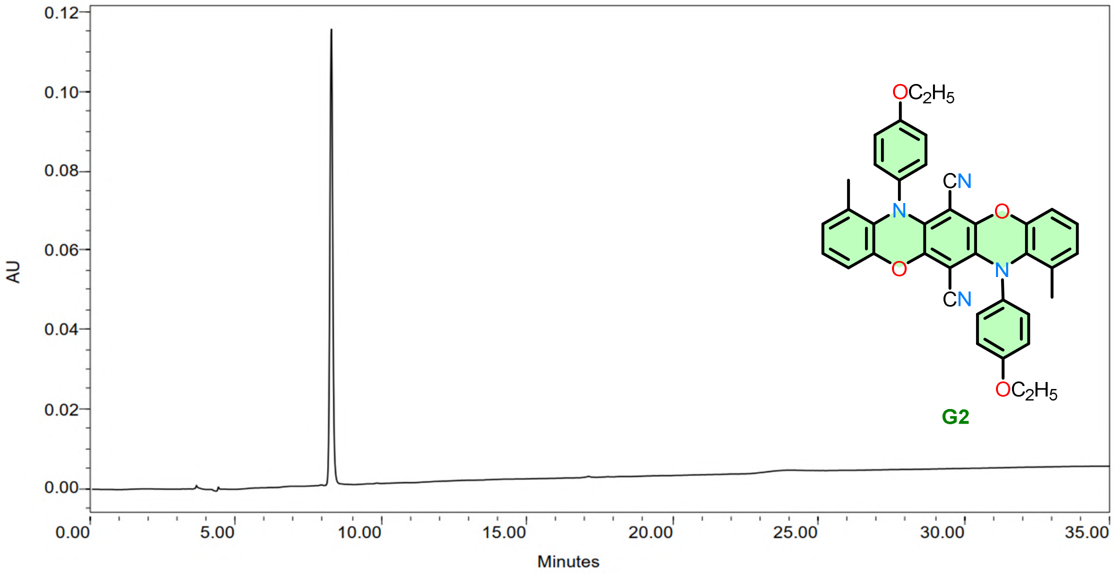

Figure S20: HPLC chromatograms of **R2**, **Y2**, and **G2** (*n*-hexane/ethyl acetate 90/10 → 0/100 over 35 min).

3 Photophysical Properties

UV/Vis absorption spectra

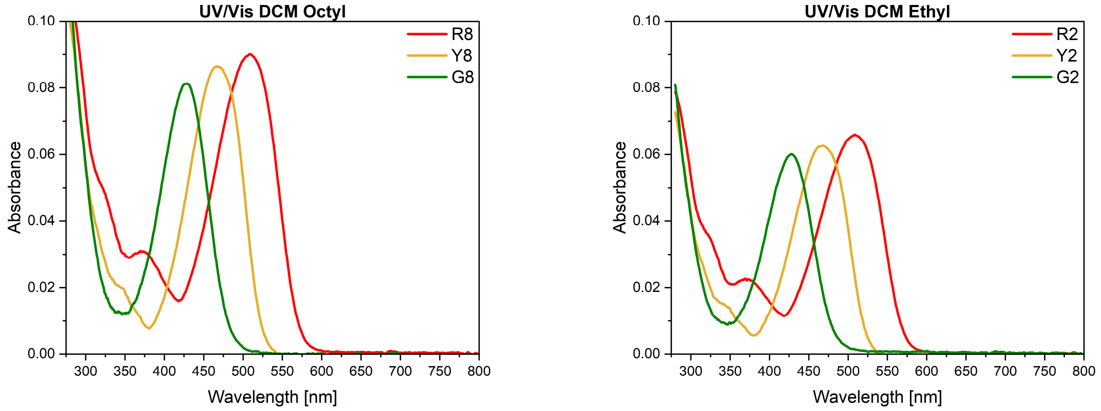

Figure S21: UV/Vis absorption spectra of the target compounds in DCM (10 µM).

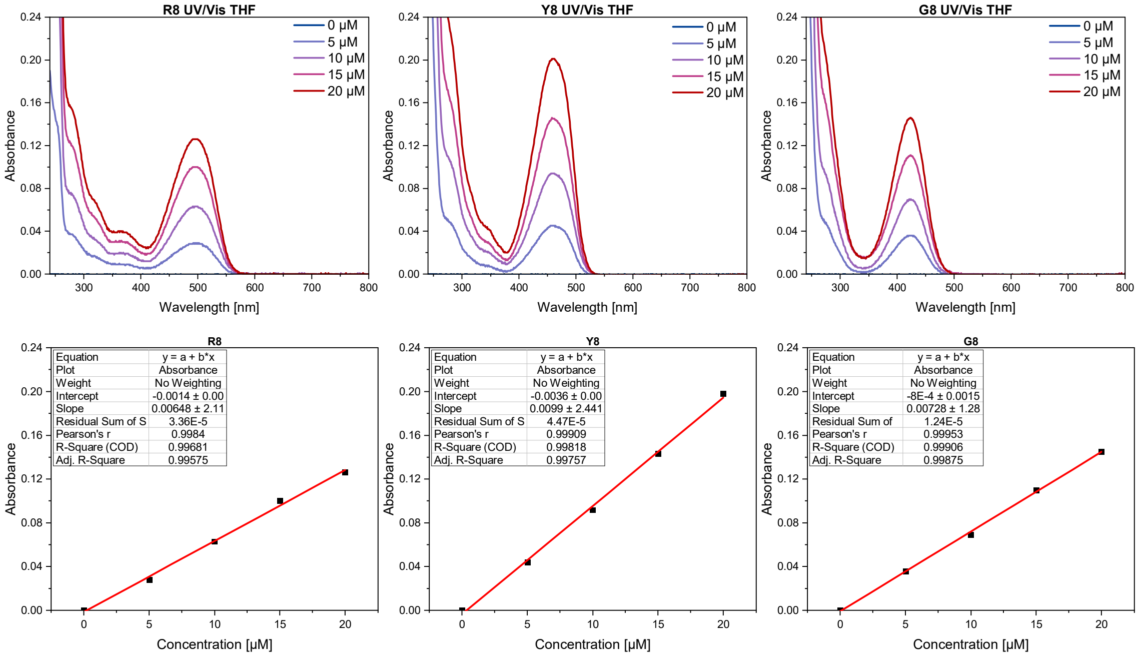

ε = 6480 L·mol^{-1}·cm^{-1} ε = 9900 L·mol^{-1}·cm^{-1} ε = 7280 L·mol^{-1}·cm^{-1}

Figure S22: Determination of molar absorption coefficients of the octyl-substituted compounds in THF.

PHOTOLUMINESCENCE IN DCM

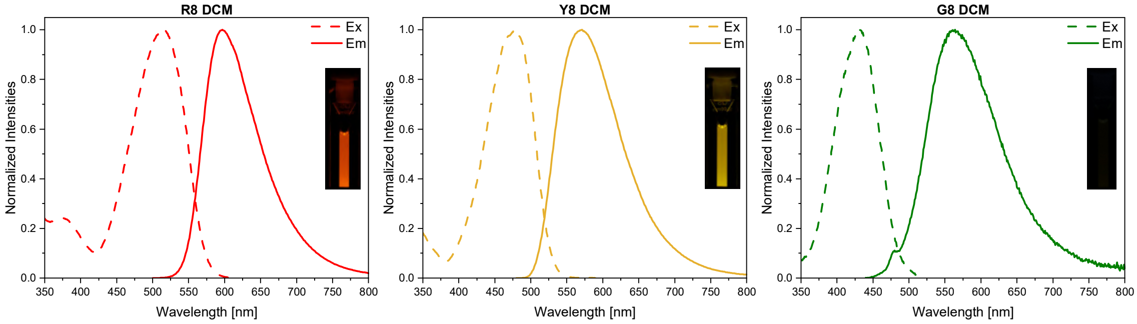

Figure S23: Normalized excitation (colored dotted line) and normalized emission (colored solid line) spectra (each 10 µM) for **R8** (left, emission at λ_{ex} = 480 nm, excitation at λ_{em} = 630 nm), **Y8** (center, emission at λ_{ex} = 460 nm, excitation at λ_{em} = 610 nm), and **G8** (right, emission at λ_{ex} = 420 nm, excitation at λ_{em} = 540 nm) in dichloromethane with corresponding photographs taken under UV-light (365 nm).

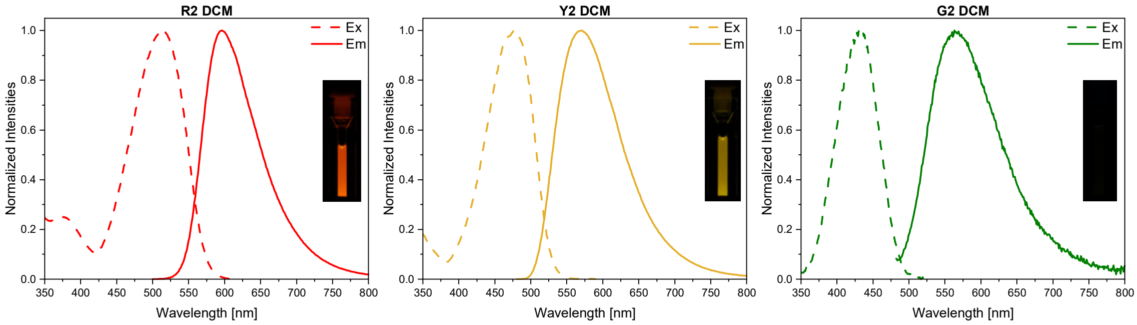

Figure S24: Normalized excitation (colored dotted line) and normalized emission (colored solid line) spectra (each 10 µM) for **R2** (left, emission at λ_{ex} = 480 nm, excitation at λ_{em} = 630 nm), **Y2** (center, emission at λ_{ex} = 460 nm, excitation at λ_{em} = 610 nm), and **G2** (right, emission at λ_{ex} = 420 nm, excitation at λ_{em} = 540 nm) in dichloromethane with corresponding photographs taken under UV-light (365 nm).

Table S1: Summary of the photophysical properties in DCM (measured wavelengths λ for absorption λ_{ab}, excitation λ_{ex} and emission λ_{em}, Stokes shifts in nm ($\Delta\lambda$) and cm^{-1} ($\Delta\nu$), absolute photoluminescence quantum yields Φ_{PL}; amplitude-weighted average fluorescence lifetimes τ_{AvAmp} [ns] as well as average radiative and non-radiative deactivation rate constants (k_r and k_{nr}); n.d. = not detectable).

Compound	λ_{ab} [nm]	λ_{ex} [nm]	λ_{em} [nm]	$\Delta\lambda_{em-ab}$ [nm]	$\Delta\nu_{ab-em}$ [cm^{-1}]	Φ_{PL}	τ_{AvAmp} [ns]	k_r [10^8 s^{-1}]	k_{nr} [10^8 s^{-1}]
**R8**	508	515	597	89	2935	0.52 ± 0.03	10.66 ± 0.02	0.49 ± 0.03	0.45 ± 0.03
**R2**	508	515	596	88	2907	0.53 ± 0.03	10.65 ± 0.02	0.50 ± 0.03	0.44 ± 0.03
**Y8**	467	477	571	104	3900	0.14 ± 0.02	3.111 ± 0.002	0.45 ± 0.1	2.76 ± 0.1
**Y2**	468	477	571	103	3854	0.17 ± 0.02	3.418 ± 0.009	0.50 ± 0.09	2.43 ± 0.09
**G8**	428	432	564	136	5634	<0.01 ± 0.02	n.d.	-	-
**G2**	428	432	563	135	5602	<0.01 ± 0.02	n.d.	-	-

PHOTOLUMINESCENCE IN POWDERS

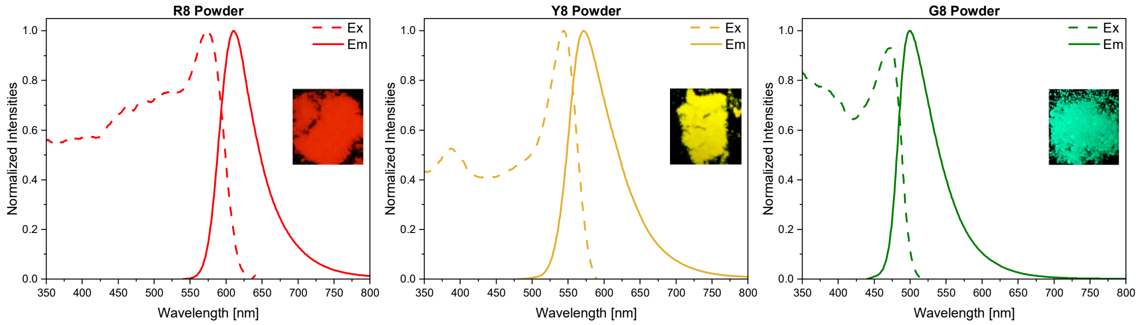

Figure S25: Normalized excitation (colored dotted line) and normalized emission (colored solid line) spectra for **R8** (left, emission at λ_{ex} = 520 nm, excitation at λ_{em} = 660 nm), **Y8** (center, emission at λ_{ex} = 440 nm, excitation at λ_{em} = 620 nm), and **G8** (right, emission at λ_{ex} = 420 nm, excitation at λ_{em} = 540 nm) of the powders with corresponding photographs taken under UV-light (395 nm).

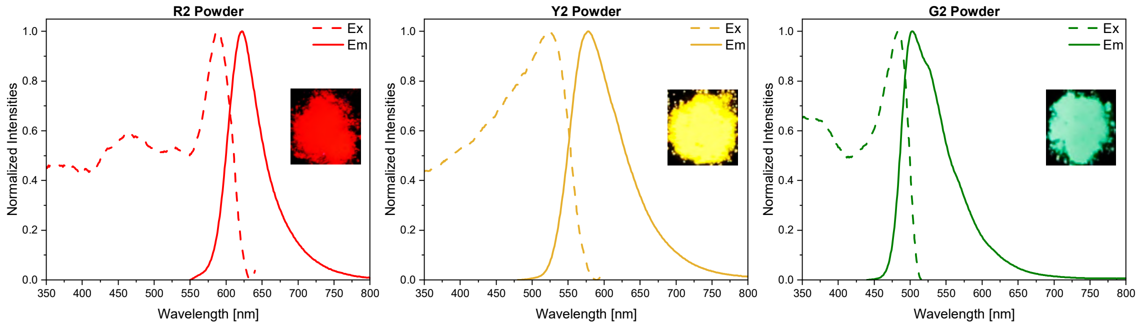

Figure S26: Normalized excitation (colored dotted line) and normalized emission (colored solid line) spectra for **R2** (left, emission at λ_{ex} = 530 nm, excitation at λ_{em} = 660 nm), **Y2** (center, emission at λ_{ex} = 440 nm, excitation at λ_{em} = 620 nm), and **G2** (right, emission at λ_{ex} = 420 nm, excitation at λ_{em} = 540 nm) of the powders with corresponding photographs taken under UV-light (395 nm).

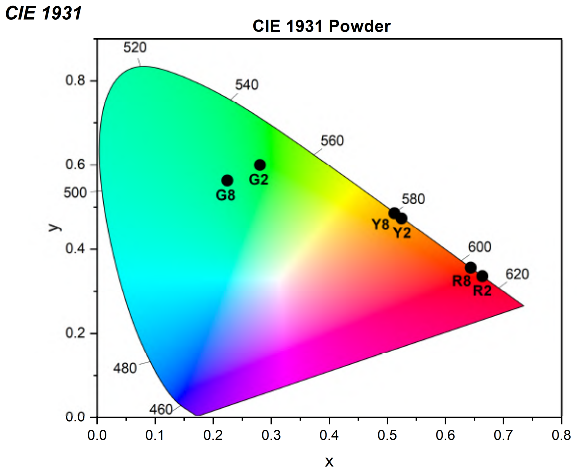

Figure S27: CIE 1931 plot of octyl and ethyl compounds derived from the solid-state emission spectra.

Table S2: Summary of the photophysical properties of the powders (measured wavelengths λ for excitation λ_{ex} and emission λ_{em}, Stokes shifts in nm ($\Delta\lambda$) and cm^{-1} ($\Delta\nu$), absolute photoluminescence quantum yields Φ_{PL}; amplitude-weighted average fluorescence lifetimes τ_{AvAmp} [ns] as well as average radiative and non-radiative

deactivation rate constants (k_r and k_{nr}); n.d. = not detectable; * = calculated for excitation wavelengths since no absorption wavelengths were determined).

Compound	λ_{ex} [nm]	λ_{em} [nm]	$\Delta\lambda_{em-ex}$ [nm]*	$\Delta\nu_{ex-em}$ [cm^{-1}]*	Φ_{PL}	τ_{AvAmp} [ns]	k_r [10^8 s^{-1}]	k_{nr} [10^8 s^{-1}]
R8	574	610	36	1028	0.22 ± 0.02	6.55 ± 0.08	0.34 ± 0.03	1.19 ± 0.03
R2	587	622	35	959	0.04 ± 0.02	1.31 ± 0.04	0.30 ± 0.16	7.33 ± 0.26
Y8	544	571	27	869	0.26 ± 0.02	8.72 ± 0.05	0.30 ± 0.02	0.85 ± 0.02
Y2	523	578	66	1819	0.23 ± 0.02	7.18 ± 0.07	0.32 ± 0.03	1.07 ± 0.03
G8	473	499	26	1102	0.40 ± 0.02	6.96 ± 0.04	0.57 ± 0.03	0.86 ± 0.03
G2	483	507	24	980	0.47 ± 0.02	6.84 ± 0.08	0.68 ± 0.04	0.07 ± 0.03

AGGREGATION SERIES

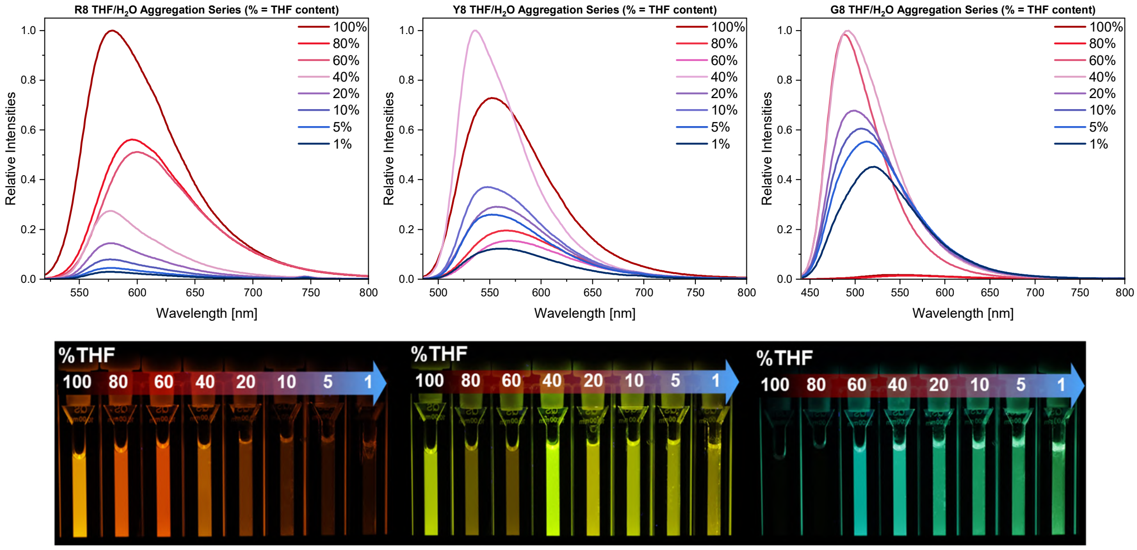

Figure S28: Relative intensities of the emission spectra for **R8** (left, emission at λ_{ex} = 500 nm), **Y8** (center, emission at λ_{ex} = 466 nm), and **G8** (right, emission at λ_{ex} = 422 nm) with corresponding photographs taken under UV-light (365 nm).

Table S3: Summary of the photophysical properties of the aggregates (measured wavelengths λ for excitation λ_{ex} and emission λ_{em}, absolute photoluminescence quantum yields Φ_{PL}, amplitude-weighted average fluorescence lifetimes τ_{AvAmp} [ns] as well as average radiative and non-radiative deactivation rate constants (k_r and k_{nr}); n.d. = not detectable).

		λ_{ex} [nm]	λ_{em} [nm]	Φ_{PL}	τ_{AvAmp} [ns]	k_r [10^8 s^{-1}]	k_{nr} [10^8 s^{-1}]
**R8**	**THF/H_2O 100/0**	503	578	0.56 ± 0.03	10.36 ± 0.02	0.54 ± 0.03	0.42 ± 0.03
	**THF/H_2O 40/60**	541	577	0.36 ± 0.02	7.47 ± 0.02	0.48 ± 0.04	0.86 ± 0.04
	**THF/H_2O 1/99**	541	577	0.11 ± 0.02	1.42 ± 0.02	0.8 ± 0.2	6.3 ± 0.3
**Y8**	**THF/H_2O 100/0**	466	553	0.27 ± 0.02	4.901 ± 0.009	0.55 ± 0.06	1.49 ± 0.06
	**THF/H_2O 40/60**	465	557	0.36 ± 0.02	5.92 ± 0.09	0.61 ± 0.06	1.08 ± 0.6
	**THF/H_2O 1/99**	478	551	0.08 ± 0.02	1.34 ± 0.01	0.6 ± 0.2	6.9 ± 0.3
**G8**	**THF/H_2O 100/0**	422	544	<0.01 ± 0.02	n.d.	-	-
	**THF/H_2O 40/60**	428	493	0.34 ± 0.02	5.45 ± 0.03	0.62 ± 0.06	1.21 ± 0.06
	**THF/H_2O 1/99**	426	520	0.23 ± 0.02	5.09 ± 0.05	0.45 ± 0.06	1.51 ± 0.06

PHOTOLUMINESCENCE IN MESOPOROUS SILICA NANOPARTICLES (MSN)

Mesoporous silica nanoparticles (MSNs) were synthesized *via* a modified *Stöber* synthesis as described before.[3] 2 mg of luminophore was dissolved in 2 mL of distilled THF and added to a solution containing cetyltrimethylammonium bromide (CTAB, 80 mg, 0.220 mmol) and tris(hydroxymethyl)aminomethane (TRISMA, 100 mg, 0.825 mmol) in 15 mL of ultrapure water. After 15 minutes of stirring, tetraethyl orthosilicate (TEOS, 0.700 mL, 658 mg, 3.27 mmol) was added to the mixture. The mixture was stirred at 750 rpm overnight at room temperature, then purified using centrifugation (4000 rpm, 5 min, washing twice with water and twice with methanol) and finally dried *in vacuo*.

Photometry was used for determining the concentration of luminophores in the MSNs, 1 mg of particles was dispersed in 1 mL of THF, filtered using a syringe filter (PTFE, 0.22 µm) and the absorbance was measured using UV/Vis spectroscopy. The determined concentrations were 7.4 µM (**R8**), 4.1 µM (**Y8**), and 3.8 µM (**G8**), corresponding to 5.6 µg/mg (**R8**), 3.1 µg/mg (**Y8**), and 3.8 µg/mg (**G8**) mass fractions of compound per mg MSN.

For the 3D-printed materials, resins were prepared consisting of 1 wt% diphenyl(2,4,6-trimethylbenzoyl)phosphine oxide as the initiator, 30 wt% poly(ethylene glycol) dimethyl acrylate as a linker, and 69 wt% of 2-[[(butylamino)carbonyl]oxy]ethyl acrylate as the monomer. To these resins, 3 wt% of MSN powders containing luminophore were added and stirred overnight. The resins were photopolymerized with 405 nm violet light using a *Photon Mono 4K* DLP printer by *Anycubic*. The applied parameters were 11 s normal exposure time, 0.5 s off time, 11 s bottom exposure time, 0.1 mm layer thickness, and 4 bottom layers. Finally, the printed objects were rinsed with isopropanol.

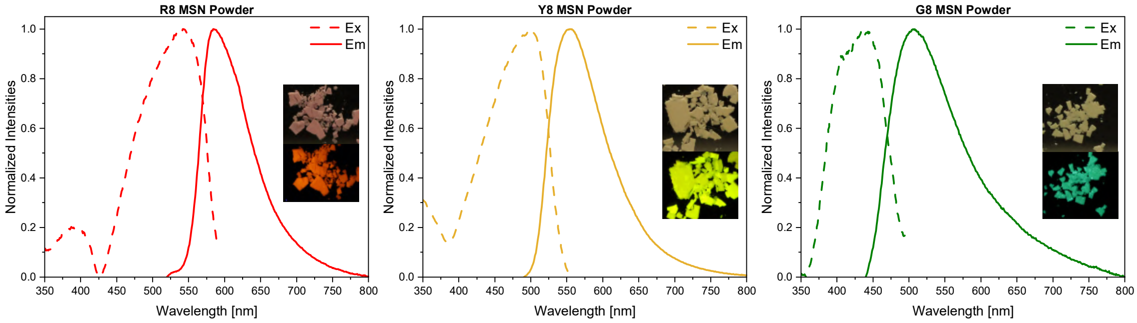

Figure S29: Normalized excitation (colored dotted line) and normalized emission (colored solid line) spectra for **R8** (left, emission at λ_{ex} = 510 nm, excitation at λ_{em} = 620 nm), **Y8** (center, emission at λ_{ex} = 460 nm, excitation at λ_{em} = 580 nm), and **G8** (right, emission at λ_{ex} = 420 nm, excitation at λ_{em} = 520 nm) of the mesoporous silica nanoparticles with corresponding photographs taken under UV-light (395 nm).

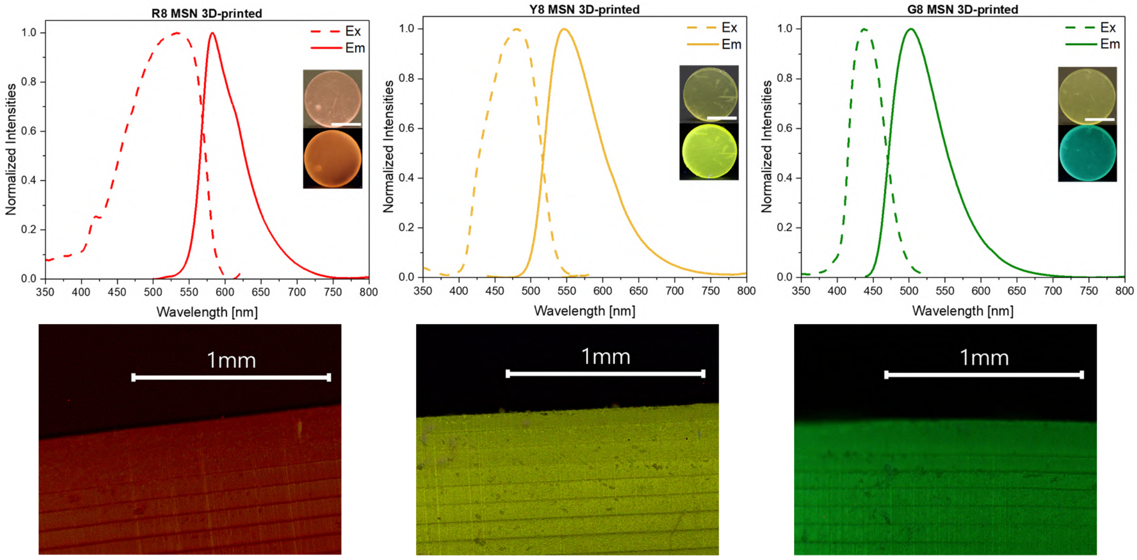

Figure S30: Top: Normalized excitation (colored dotted line) and normalized emission (colored solid line) spectra for **R8** (left, emission at λ_{ex} = 480 nm, excitation at λ_{em} = 650 nm), **Y8** (center, emission at λ_{ex} = 420 nm, excitation at λ_{em} = 600 nm), and **G8** (right, emission at λ_{ex} = 420 nm, excitation at λ_{em} = 540 nm) of the mesoporous silica nanoparticles embedded in 3D-printed objects with corresponding pictures taken under UV-light (395 nm). Scale bar: 0.5 cm. Bottom: Microscopic images of the 3D-printed objects under UV-light (365 nm), showing full homogeneity.

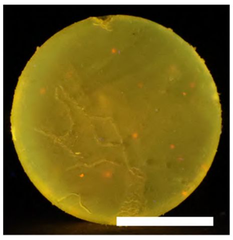

Figure S31: Microscopic images of a 3D-printed object of **R8** without using MSNs under UV-light (365 nm), showing the inhomogeneous distribution of the compound.

Table S4: Summary of the photophysical properties of the compounds in mesoporous silica nanoparticles as powders and in 3D-printed objects (measured wavelengths λ for excitation λ_{ex} and emission λ_{em}, Stokes shifts in nm ($\Delta\lambda$) and cm^{-1} ($\Delta\nu$), absolute photoluminescence quantum yields Φ_{PL}; amplitude-weighted average fluorescence lifetimes τ_{AvAmp} [ns] as well as average radiative and non-radiative deactivation rate constants (k_r and k_{nr})).

Compound	λ_{ex} [nm]	λ_{em} [nm]	$\Delta\lambda_{em-ex}$ [nm]*	$\Delta\nu_{ex-em}$ [cm^{-1}]*	Φ_{PL}	τ_{AvAmp} [ns]	k_r [10^8 s^{-1}]	k_{nr} [10^8 s^{-1}]
R8 MSN Powder	542	585	43	1356	0.08 ± 0.02	2.69 ± 0.03	0.29 ± 0.08	3.42 ± 0.09
R8 MSN 3D	533	582	49	1580	0.12 ± 0.02	2.66 ± 0.02	0.45 ± 0.08	3.31 ± 0.09
Y8 MSN Powder	500	555	55	1982	0.16 ± 0.02	5.33 ± 0.04	0.30 ± 0.04	1.58 ± 0.04
Y8 MSN 3D	482	545	63	2398	0.51 ± 0.03	8.53 ± 0.03	0.60 ± 0.03	0.57 ± 0.02
G8 MSN Powder	436	508	72	3251	0.04 ± 0.02	3.55 ± 0.03	0.11 ± 0.06	2.70 ± 0.07
G8 MSN 3D	437	504	67	3042	0.27 ± 0.02	4.39 ± 0.03	0.62 ± 0.05	1.66 ± 0.05

LIFETIME REPORTS

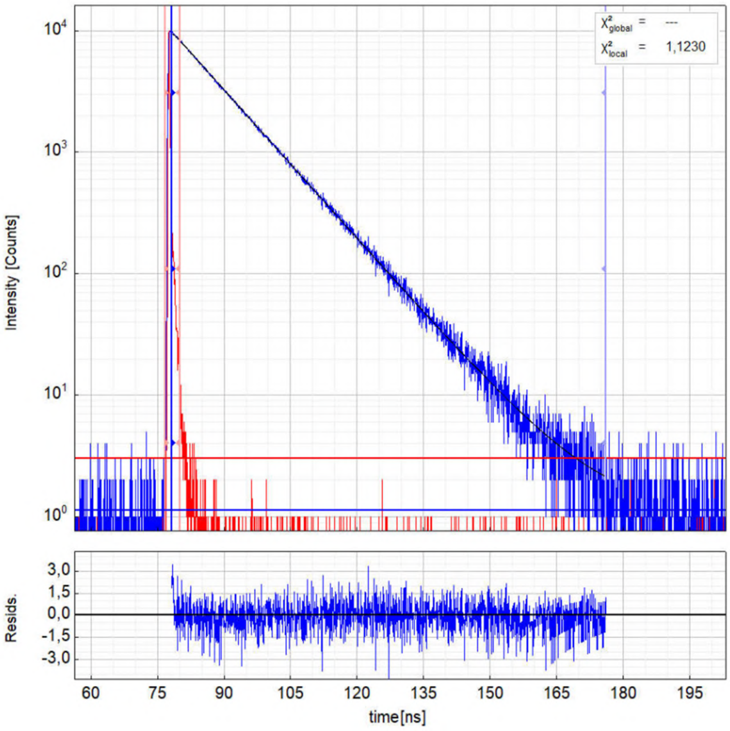

Parameter	Value	Δ	δ
A_1[kCnts/Chnl]	10,477	±0,026	0,2%
τ_1[ns]	10,661	±0,016	0,1%
I_1[kCnts]	1 745,2	±3,0	0,2%
A_{Rel1}[%]	100,0	---	---
I_{Rel1}[%]	100,0	---	---
$Bkgr_{Dec}$[kCnts]	0,0012	±0,0002	13%
$Bkgr_{IRF}$[Cnts/Chnl]	3,0000	---	---
$Shift_{IRF}$[ps]	200,0000	---	---
τ_{AvInt}[ns]	10,661	±0,016	0,1%
τ_{AvAmp}[ns]	10,661	±0,016	0,1%

Figure S32: Raw time-resolved photoluminescence decay of **R8** in liquid DCM at r.t. (blue) with instrumental response function in red (left), including the residuals (λ_{ex} = 505.7 nm, λ_{em} = 597 nm); fitting parameters including pre-exponential factors and confidence limits (right).

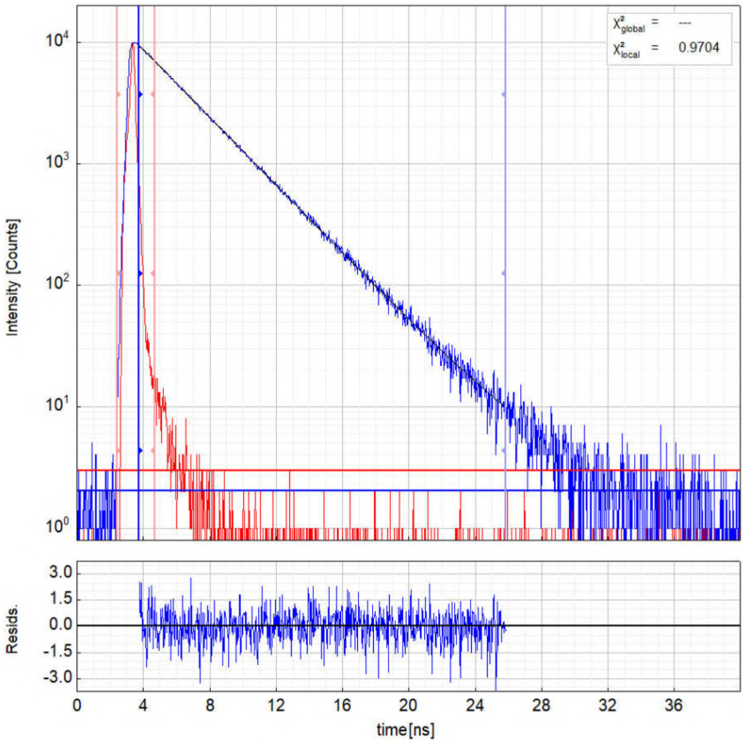

Parameter	Value	Δ	δ
A_1[kCnts/Chnl]	10.893	±0.025	0.2%
τ_1[ns]	3.1109	±0.0020	0.1%
I_1[kCnts]	1 058.9	±1.4	0.1%
A_{Rel1}[%]	100.0	---	---
I_{Rel1}[%]	100.0	---	---
$Bkgr_{Dec}$[kCnts]	0.0021	±0.0002	9.7%
$Bkgr_{IRF}$[Cnts/Chnl]	3.0000	---	---
$Shift_{IRF}$[ps]	250.0000	---	---
τ_{AvInt}[ns]	3.1109	±0.0020	0.1%
τ_{AvAmp}[ns]	3.1109	±0.0020	0.1%

Figure S33: Raw time-resolved photoluminescence decay of **Y8** in liquid DCM at r.t. (blue) with instrumental response function in red (left), including the residuals (λ_{ex} = 440.0 nm, λ_{em} = 597 nm); fitting parameters including pre-exponential factors and confidence limits (right).

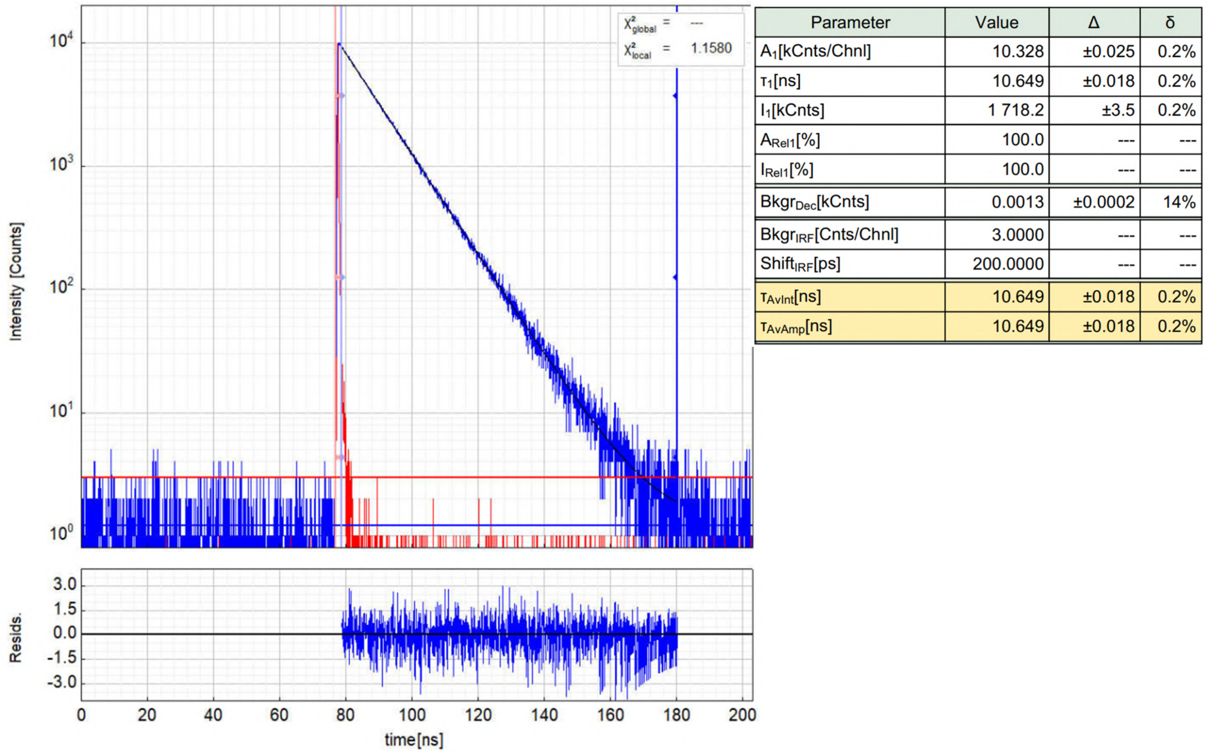

Parameter	Value	Δ	δ
A_1[kCnts/Chnl]	10.328	±0.025	0.2%
τ_1[ns]	10.649	±0.018	0.2%
I_1[kCnts]	1 718.2	±3.5	0.2%
A_{Rel1}[%]	100.0	---	---
I_{Rel1}[%]	100.0	---	---
$Bkgr_{Dec}$[kCnts]	0.0013	±0.0002	14%
$Bkgr_{IRF}$[Cnts/Chnl]	3.0000	---	---
$Shift_{IRF}$[ps]	200.0000	---	---
τ_{AvInt}[ns]	10.649	±0.018	0.2%
τ_{AvAmp}[ns]	10.649	±0.018	0.2%

Figure S34: Raw time-resolved photoluminescence decay of **R2** in liquid DCM at r.t. (blue) with instrumental response function in red (left), including the residuals (λ_{ex} = 505.7 nm, λ_{em} = 596 nm); fitting parameters including pre-exponential factors and confidence limits (right)

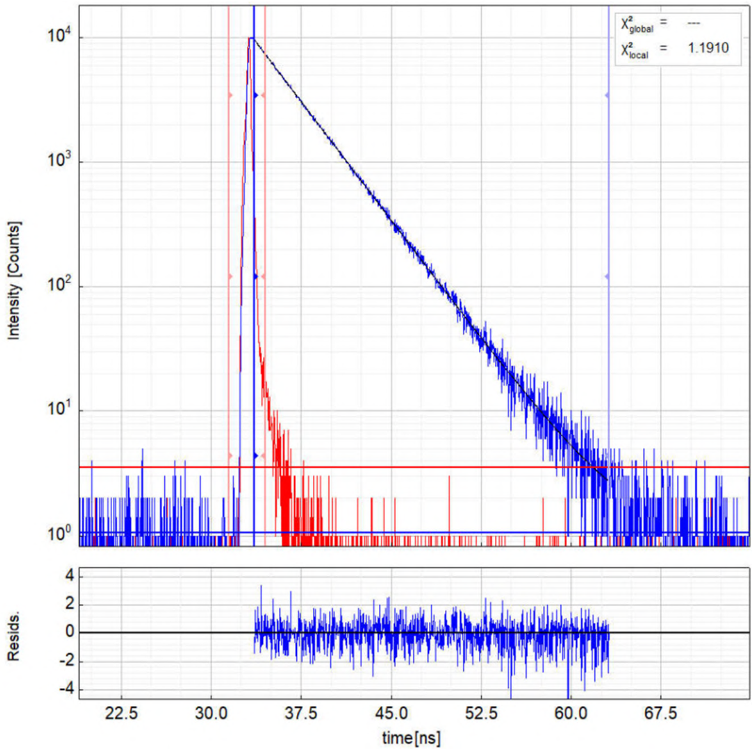

Parameter	Value	Δ	δ
A_1[kCnts/Chnl]	11.139	±0.030	0.3%
τ_1[ns]	3.4175	±0.0086	0.2%
I_1[kCnts]	1 189.6	±1.8	0.1%
A_{Rel1}[%]	100.0	---	---
I_{Rel1}[%]	100.0	---	---
$Bkgr_{Dec}$[kCnts]	0.0011	±0.0003	25%
$Bkgr_{IRF}$[Cnts/Chnl]	3.5471	---	---
$Shift_{IRF}$[ps]	77.3057	---	---
A_{Scatt}[kCnts]	82	±22	26%
τ_{AvInt}[ns]	3.4175	±0.0086	0.2%
τ_{AvAmp}[ns]	3.4175	±0.0086	0.2%

Figure S35: Raw time-resolved photoluminescence decay of **Y2** in liquid DCM at r.t. (blue) with instrumental response function in red (left), including the residuals (λ_{ex} = 440.0 nm, λ_{em} = 597 nm); fitting parameters including pre-exponential factors and confidence limits (right).

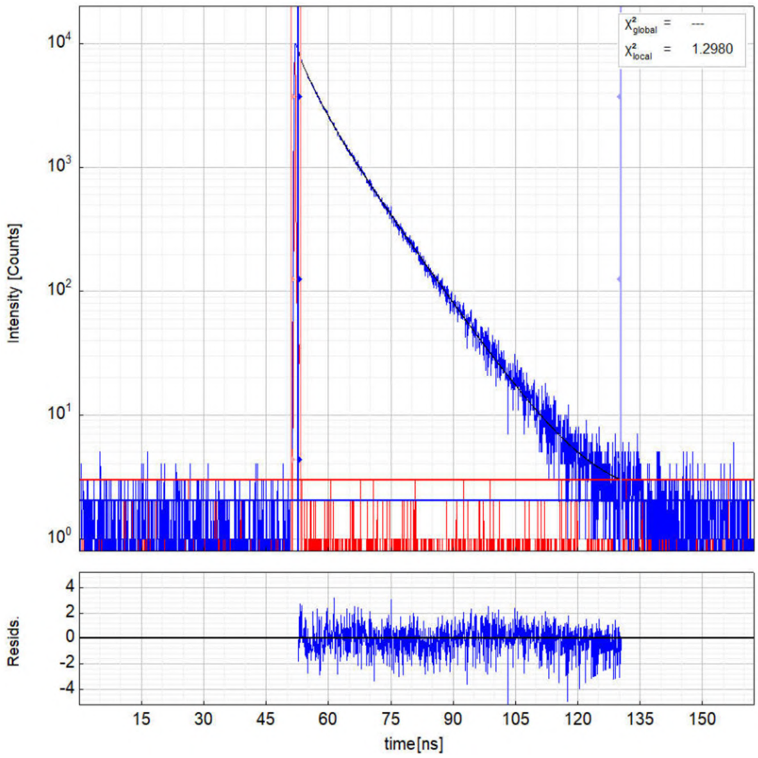

Parameter	Value	Δ	δ
A_1[kCnts/Chnl]	5.10	±0.21	4.1%
τ_1[ns]	9.16	±0.11	1.2%
I_1[kCnts]	730	±22	3.0%
A_{Rel1}[%]	52.6	±1.8	3.4%
I_{Rel1}[%]	73.6	±2.2	2.9%
A_2[kCnts/Chnl]	4.60	±0.14	3.0%
τ_2[ns]	3.65	±0.18	4.8%
I_2[kCnts]	263	±22	8.0%
A_{Rel2}[%]	47.5	±1.8	3.8%
I_{Rel2}[%]	26.5	±2.2	8.1%
$Bkgr_{Dec}$[kCnts]	0.0021	±0.0003	13%
$Bkgr_{IRF}$[Cnts/Chnl]	3.0000	---	---
$Shift_{IRF}$[ps]	200.0000	---	---
A_{Scatt}[kCnts]	500	±150	29%
τ_{AvInt}[ns]	7.700	±0.030	0.4%
τ_{AvAmp}[ns]	6.546	±0.081	1.2%

Figure S36: Raw time-resolved photoluminescence decay of **R8** in the amorphous powder at r.t. (blue) with instrumental response function in red (left), including the residuals (λ_{ex} = 505.7 nm, λ_{em} = 610 nm); fitting parameters including pre-exponential factors and confidence limits (right).

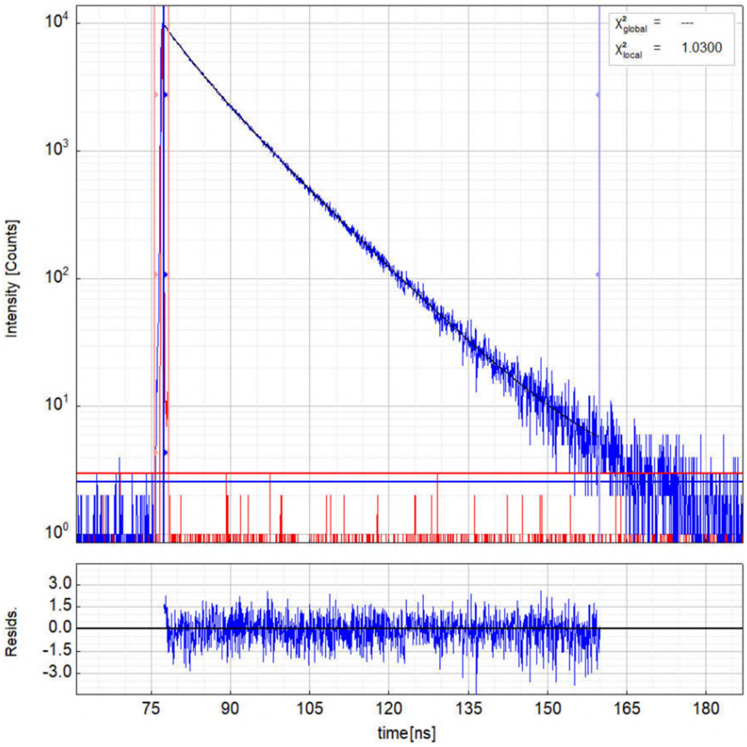

Parameter	Value	Δ	δ
A_1[kCnts/Chnl]	4.05	±0.34	8.2%
τ_1[ns]	5.34	±0.30	5.5%
I_1[kCnts]	338	±40	12%
A_{Rel1}[%]	39.4	±3.2	8.1%
I_{Rel1}[%]	24.2	±2.9	12%
A_2[kCnts/Chnl]	6.23	±0.32	5.1%
τ_2[ns]	10.92	±0.17	1.5%
I_2[kCnts]	1 062	±40	3.7%
A_{Rel2}[%]	60.7	±3.2	5.2%
I_{Rel2}[%]	75.9	±2.9	3.7%
$Bkgr_{Dec}$[kCnts]	0.0026	±0.0005	18%
$Bkgr_{IRF}$[Cnts/Chnl]	3.0000	---	---
$Shift_{IRF}$[ps]	250.0000	---	---
τ_{AvInt}[ns]	9.574	±0.012	0.1%
τ_{AvAmp}[ns]	8.720	±0.046	0.5%

Figure S37: Raw time-resolved photoluminescence decay of **Y8** in the amorphous powder at r.t. (blue) with instrumental response function in red (left), including the residuals (λ_{ex} = 505.7 nm, λ_{em} = 571 nm); fitting parameters including pre-exponential factors and confidence limits (right).

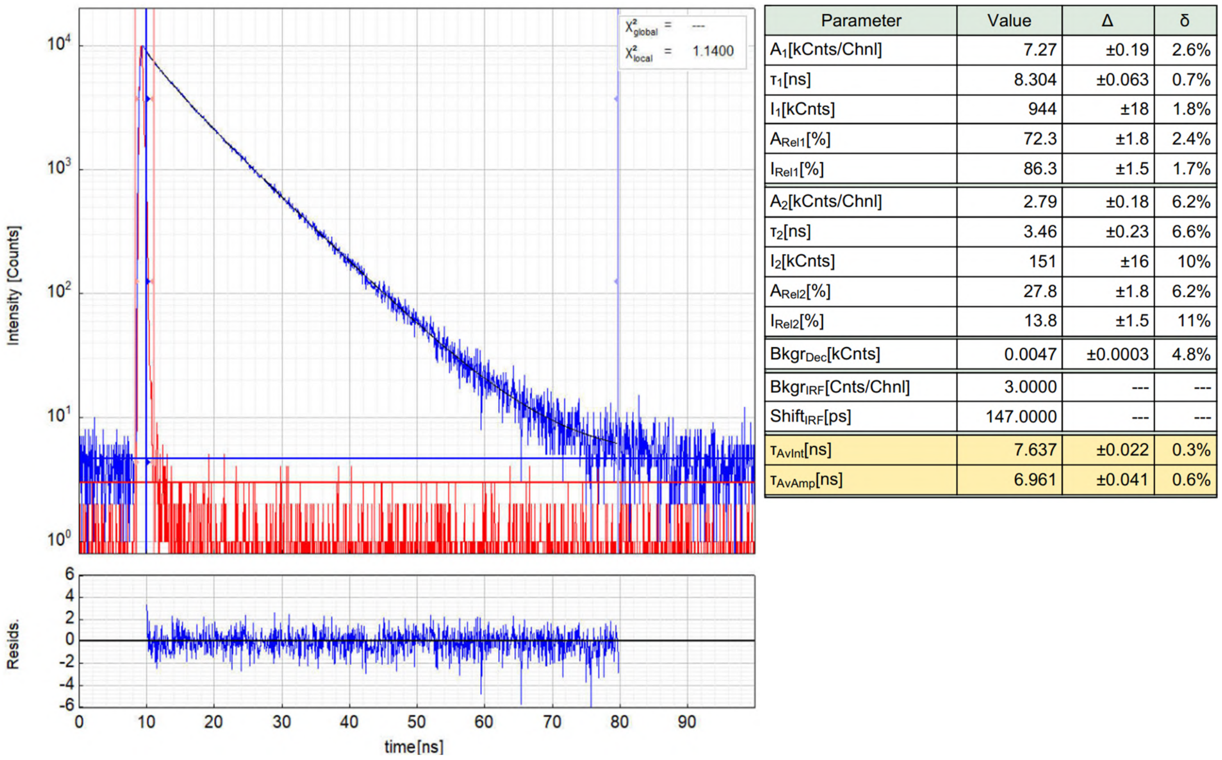

Parameter	Value	Δ	δ
A_1[kCnts/Chnl]	7.27	±0.19	2.6%
τ_1[ns]	8.304	±0.063	0.7%
I_1[kCnts]	944	±18	1.8%
A_{Rel1}[%]	72.3	±1.8	2.4%
I_{Rel1}[%]	86.3	±1.5	1.7%
A_2[kCnts/Chnl]	2.79	±0.18	6.2%
τ_2[ns]	3.46	±0.23	6.6%
I_2[kCnts]	151	±16	10%
A_{Rel2}[%]	27.8	±1.8	6.2%
I_{Rel2}[%]	13.8	±1.5	11%
$Bkgr_{Dec}$[kCnts]	0.0047	±0.0003	4.8%
$Bkgr_{IRF}$[Cnts/Chnl]	3.0000	---	---
$Shift_{IRF}$[ps]	147.0000	---	---
τ_{AvInt}[ns]	7.637	±0.022	0.3%
τ_{AvAmp}[ns]	6.961	±0.041	0.6%

Figure S38: Raw time-resolved photoluminescence decay of **G8** in the amorphous powder at r.t. (blue) with instrumental response function in red (left), including the residuals (λ_{ex} = 440.0 nm, λ_{em} = 499 nm); fitting parameters including pre-exponential factors and confidence limits (right).

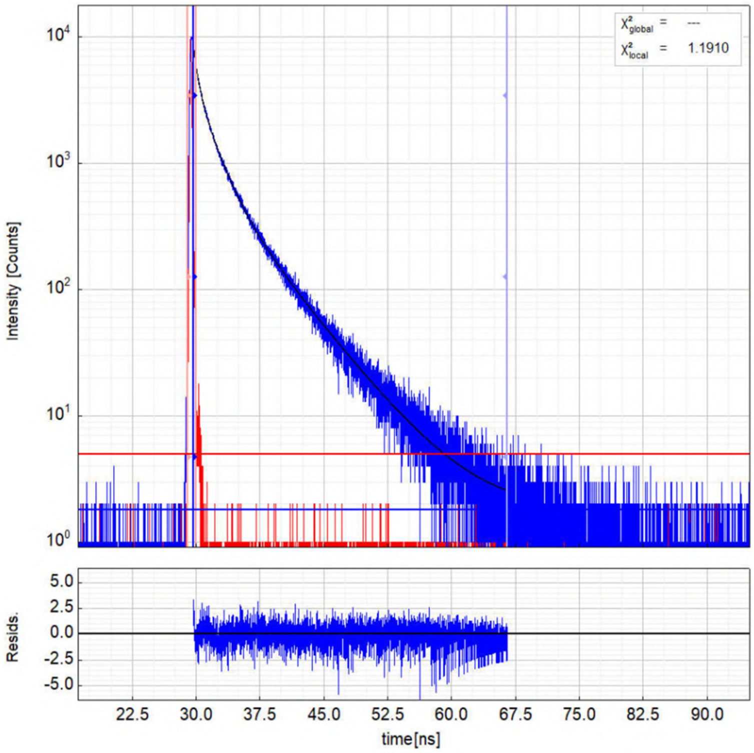

Parameter	Value	Δ	δ
A_1[kCnts/Chnl]	1.019	±0.091	8.9%
τ_1[ns]	5.18	±0.16	2.9%
I_1[kCnts]	659	±38	5.8%
A_{Rel1}[%]	8.2	±0.6	6.6%
I_{Rel1}[%]	32.2	±1.8	5.5%
A_2[kCnts/Chnl]	7.25	±0.29	4.0%
τ_2[ns]	0.473	±0.035	7.4%
I_2[kCnts]	429	±28	6.5%
A_{Rel2}[%]	57.9	±1.4	2.4%
I_{Rel2}[%]	21.0	±1.5	6.7%
A_3[kCnts/Chnl]	4.26	±0.22	5.0%
τ_3[ns]	1.806	±0.094	5.2%
I_3[kCnts]	961.0	±8.8	0.9%
A_{Rel3}[%]	34.0	±1.0	2.8%
I_{Rel3}[%]	47.0	±0.4	0.8%
$Bkgr_{Dec}$[kCnts]	0.0019	±0.0003	16%
$Bkgr_{IRF}$[Cnts/Chnl]	5.0000	---	---
$Shift_{IRF}$[ps]	100.0000	---	---
τ_{AvInt}[ns]	2.610	±0.021	0.8%
τ_{AvAmp}[ns]	1.308	±0.036	2.7%

Figure S39: Raw time-resolved photoluminescence decay of **R2** in the amorphous powder at r.t. (blue) with instrumental response function in red (left), including the residuals (λ_{ex} = 505.7 nm, λ_{em} = 622 nm); fitting parameters including pre-exponential factors and confidence limits (right).

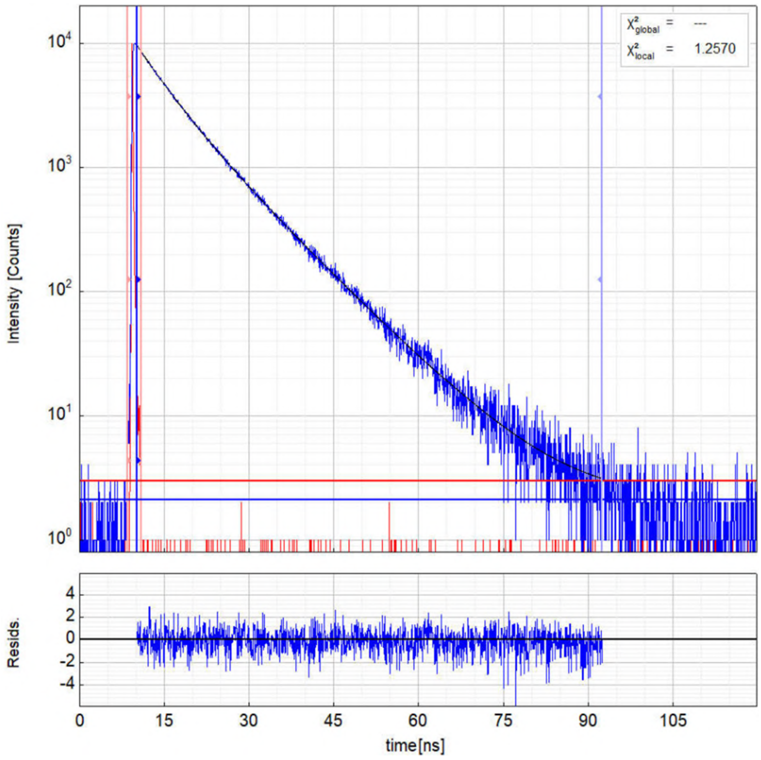

Parameter	Value	Δ	δ
A_1[kCnts/Chnl]	5.86	±0.48	8.2%
τ_1[ns]	4.91	±0.34	6.8%
I_1[kCnts]	449	±70	16%
A_{Rel1}[%]	53.2	±5.1	9.4%
I_{Rel1}[%]	36.4	±5.8	16%
A_2[kCnts/Chnl]	5.16	±0.61	12%
τ_2[ns]	9.76	±0.29	2.9%
I_2[kCnts]	786	±73	9.2%
A_{Rel2}[%]	46.9	±5.1	11%
I_{Rel2}[%]	63.7	±5.8	9.0%
$Bkgr_{Dec}$[kCnts]	0.0022	±0.0004	19%
$Bkgr_{IRF}$[Cnts/Chnl]	3.0000	---	---
$Shift_{IRF}$[ps]	78.3483	---	---
τ_{AvInt}[ns]	7.991	±0.029	0.4%
τ_{AvAmp}[ns]	7.175	±0.071	1.0%

Figure S40: Raw time-resolved photoluminescence decay of **Y2** in the amorphous powder at r.t. (blue) with instrumental response function in red (left), including the residuals (λ_{ex} = 505.7 nm, λ_{em} = 610 nm); fitting parameters including pre-exponential factors and confidence limits (right).

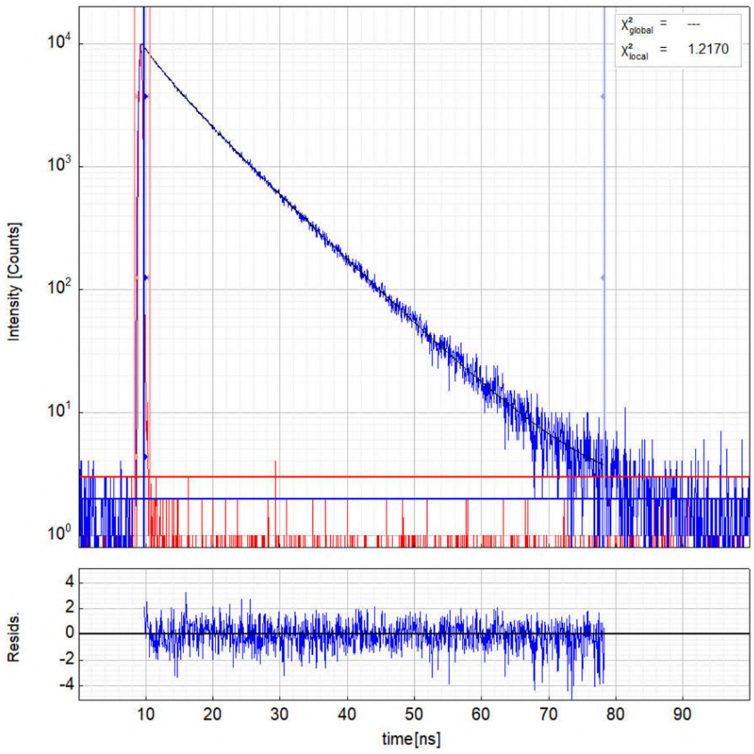

Parameter	Value	Δ	δ
A_1[kCnts/Chnl]	7.11	±0.36	5.0%
τ_1[ns]	8.31	±0.11	1.3%
I_1[kCnts]	923	±35	3.8%
A_{Rel1}[%]	69.1	±2.7	3.9%
I_{Rel1}[%]	84.0	±3.2	3.8%
A_2[kCnts/Chnl]	3.19	±0.24	7.4%
τ_2[ns]	3.55	±0.41	11%
I_2[kCnts]	177	±35	20%
A_{Rel2}[%]	31.0	±2.7	8.6%
I_{Rel2}[%]	16.1	±3.2	20%
$Bkgr_{Dec}$[kCnts]	0.0020	±0.0005	25%
$Bkgr_{IRF}$[Cnts/Chnl]	3.0000	---	---
$Shift_{IRF}$[ps]	300.0000	---	---
τ_{AvInt}[ns]	7.545	±0.023	0.3%
τ_{AvAmp}[ns]	6.835	±0.079	1.2%

Figure S41: Raw time-resolved photoluminescence decay of **G2** in the amorphous powder at r.t. (blue) with instrumental response function in red (left), including the residuals (λ_{ex} = 440.0 nm, λ_{em} = 507 nm); fitting parameters including pre-exponential factors and confidence limits (right).

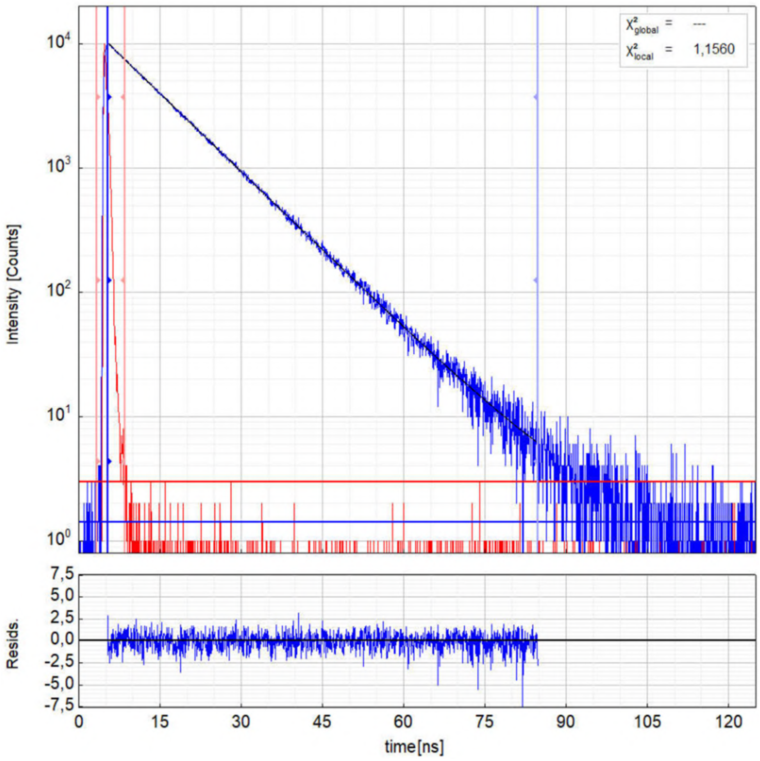

Parameter	Value	Δ	δ
A_1[kCnts/Chnl]	10,506	±0,024	0,2%
τ_1[ns]	10,359	±0,015	0,1%
I_1[kCnts]	1 700,4	±4,0	0,2%
A_{Rel1}[%]	100,0	---	---
I_{Rel1}[%]	100,0	---	---
$Bkgr_{Dec}$[kCnts]	0,0015	±0,0004	25%
$Bkgr_{IRF}$[Cnts/Chnl]	3,0000	---	---
$Shift_{IRF}$[ps]	200,0000	---	---
A_{Scatt}[kCnts]	51,2	±8,0	15%
τ_{AvInt}[ns]	10,359	±0,015	0,1%
τ_{AvAmp}[ns]	10,359	±0,015	0,1%

Figure S42: Raw time-resolved photoluminescence decay of **R8** in a liquid THF/H_2O 100/0 mixture at r.t. (blue) with instrumental response function in red (left), including the residuals (λ_{ex} = 505.7 nm, λ_{em} = 577 nm); fitting parameters including pre-exponential factors and confidence limits (right).

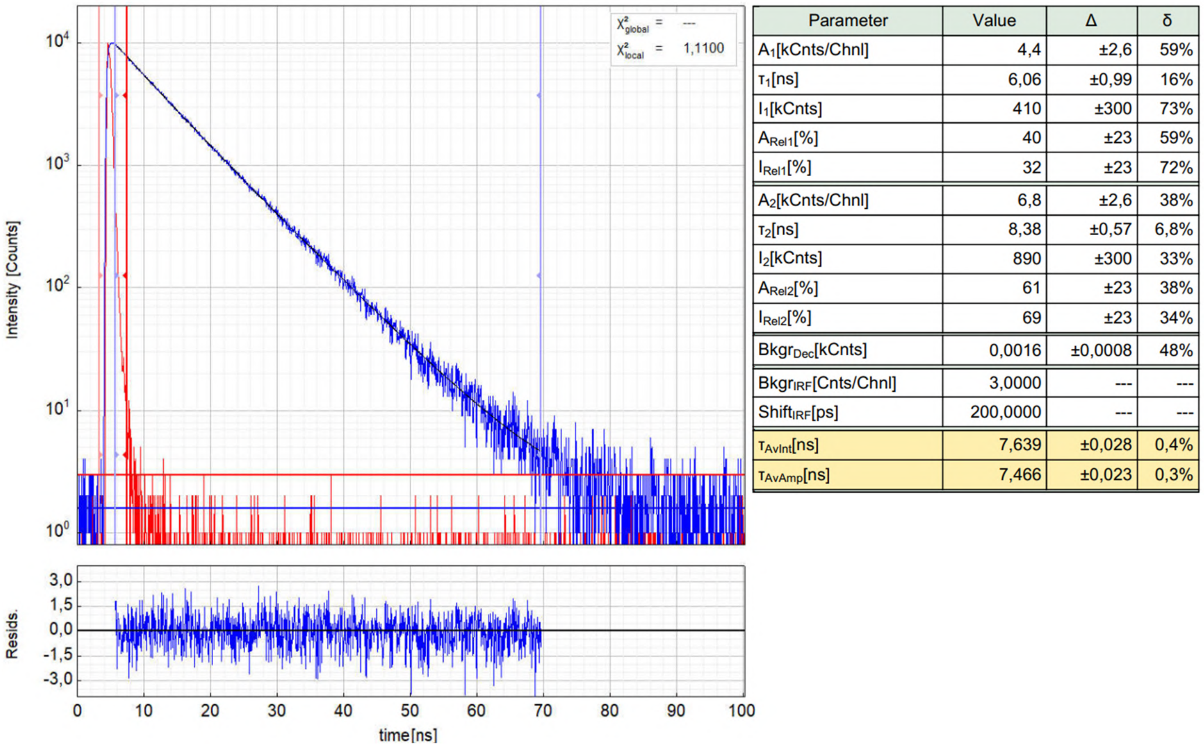

Parameter	Value	Δ	δ
A_1[kCnts/Chnl]	4,4	±2,6	59%
τ_1[ns]	6,06	±0,99	16%
I_1[kCnts]	410	±300	73%
A_{Rel1}[%]	40	±23	59%
I_{Rel1}[%]	32	±23	72%
A_2[kCnts/Chnl]	6,8	±2,6	38%
τ_2[ns]	8,38	±0,57	6,8%
I_2[kCnts]	890	±300	33%
A_{Rel2}[%]	61	±23	38%
I_{Rel2}[%]	69	±23	34%
$Bkgr_{Dec}$[kCnts]	0,0016	±0,0008	48%
$Bkgr_{IRF}$[Cnts/Chnl]	3,0000	---	---
$Shift_{IRF}$[ps]	200,0000	---	---
τ_{AvInt}[ns]	7,639	±0,028	0,4%
τ_{AvAmp}[ns]	7,466	±0,023	0,3%

Figure S43: Raw time-resolved photoluminescence decay of **R8** in a liquid THF/H_2O 4/60 mixture at r.t. (blue) with instrumental response function in red (left), including the residuals (λ_{ex} = 505.7 nm, λ_{em} = 577 nm); fitting parameters including pre-exponential factors and confidence limits (right).

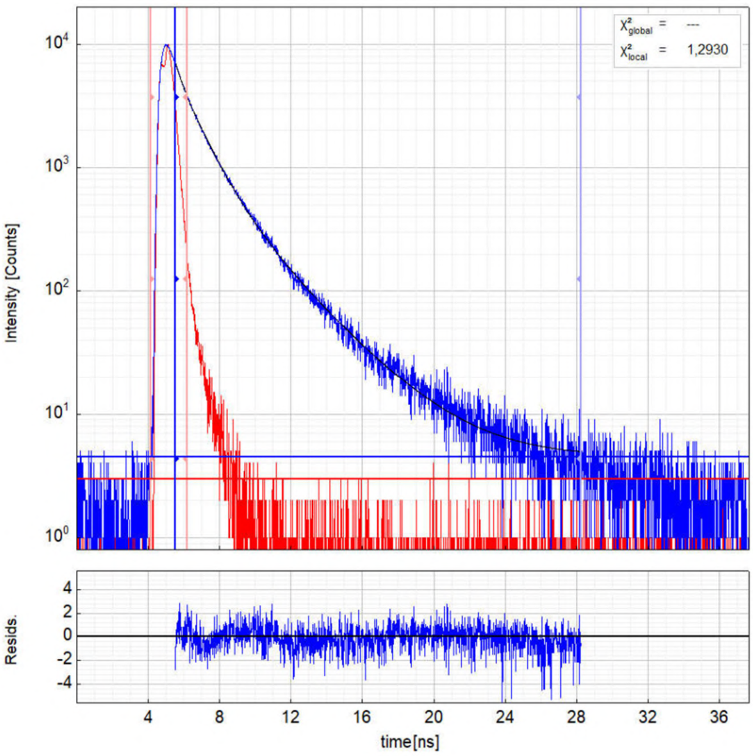

Parameter	Value	Δ	δ
A_1[kCnts/Chnl]	7,025	±0,091	1,3%
τ_1[ns]	1,129	±0,028	2,4%
I_1[kCnts]	495,6	±8,6	1,7%
A_{Rel1}[%]	83,2	±1,0	1,1%
I_{Rel1}[%]	66,1	±1,5	2,3%
A_2[kCnts/Chnl]	1,43	±0,12	8,4%
τ_2[ns]	2,866	±0,077	2,7%
I_2[kCnts]	255	±14	5,4%
A_{Rel2}[%]	16,9	±1,0	5,6%
I_{Rel2}[%]	34,0	±1,5	4,4%
$Bkgr_{Dec}$[kCnts]	0,0046	±0,0003	4,5%
$Bkgr_{IRF}$[Cnts/Chnl]	3,0000	---	---
$Shift_{IRF}$[ps]	0,0000	---	---
A_{Scatt}[kCnts]	263	±13	4,7%
τ_{AvInt}[ns]	1,7185	±0,0095	0,6%
τ_{AvAmp}[ns]	1,422	±0,015	1,0%

Figure S44: Raw time-resolved photoluminescence decay of **R8** in a liquid THF/H_2O 1/99 mixture at r.t. (blue) with instrumental response function in red (left), including the residuals (λ_{ex} = 505.7 nm, λ_{em} = 577 nm); fitting parameters including pre-exponential factors and confidence limits (right).

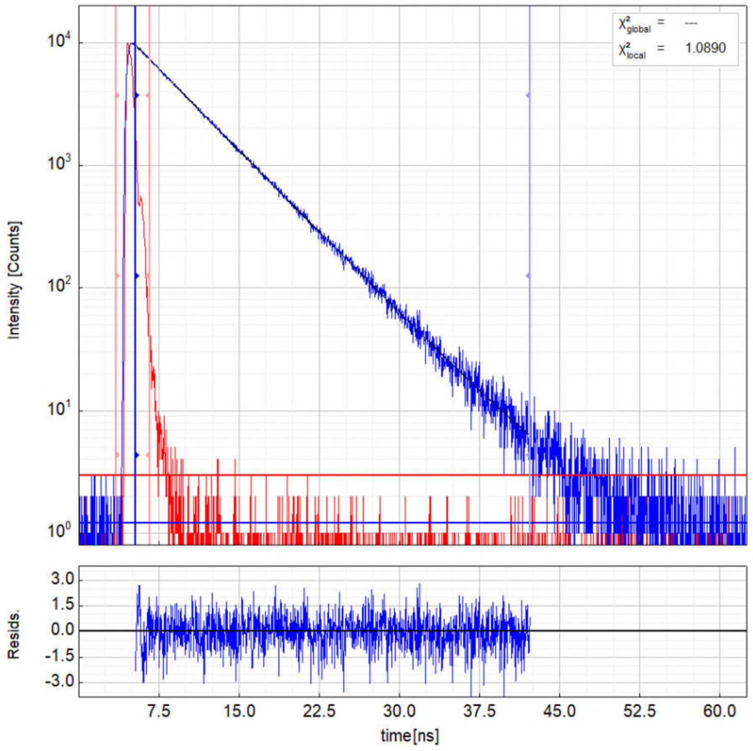

Parameter	Value	Δ	δ
A_1[kCnts/Chnl]	10.525	±0.028	0.3%
τ_1[ns]	4.9010	±0.0086	0.2%
I_1[kCnts]	1 612.0	±3.2	0.2%
A_{Rel1}[%]	100.0	---	---
I_{Rel1}[%]	100.0	---	---
$Bkgr_{Dec}$[kCnts]	0.0013	±0.0004	33%
$Bkgr_{IRF}$[Cnts/Chnl]	3.0000	---	---
$Shift_{IRF}$[ps]	150.0000	---	---
A_{Scatt}[kCnts]	137	±13	9.4%
τ_{AvInt}[ns]	4.9010	±0.0086	0.2%
τ_{AvAmp}[ns]	4.9010	±0.0086	0.2%

Figure S45: Raw time-resolved photoluminescence decay of **Y8** in a liquid THF/H_2O 100/0 mixture at r.t. (blue) with instrumental response function in red (left), including the residuals (λ_{ex} = 440.0 nm, λ_{em} = 557 nm); fitting parameters including pre-exponential factors and confidence limits (right).

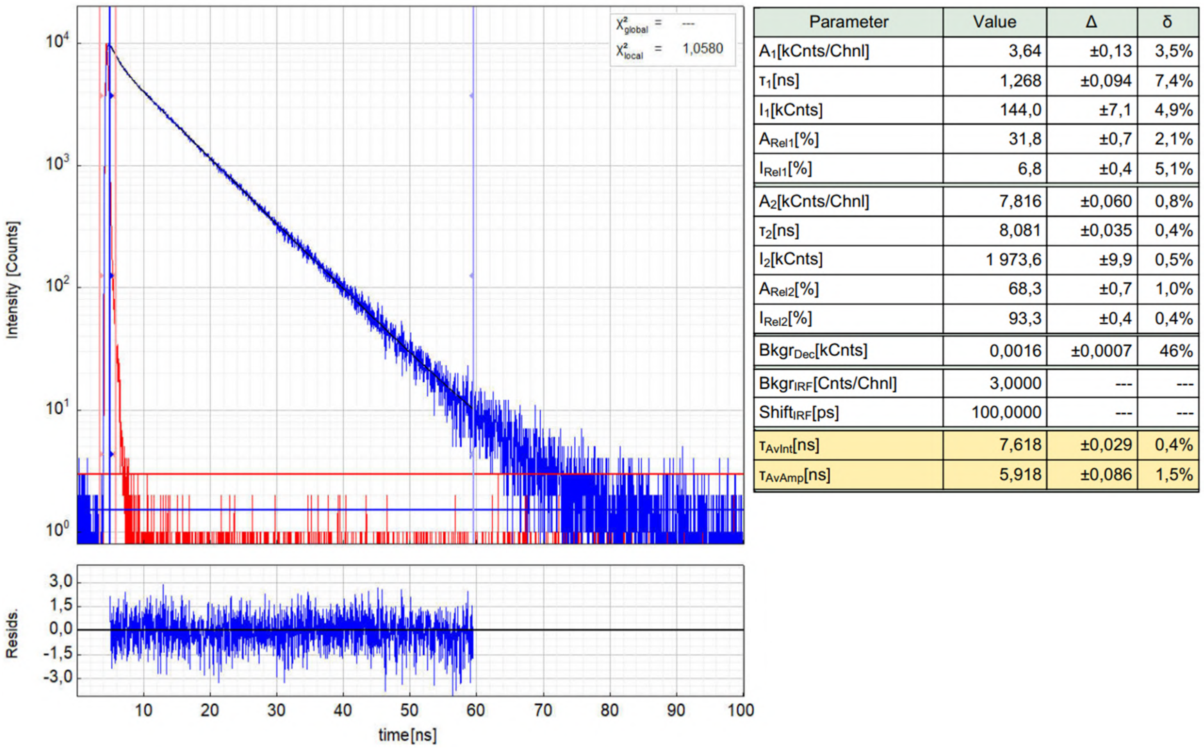

Parameter	Value	Δ	δ
A_1[kCnts/Chnl]	3,64	±0,13	3,5%
τ_1[ns]	1,268	±0,094	7,4%
I_1[kCnts]	144,0	±7,1	4,9%
A_{Rel1}[%]	31,8	±0,7	2,1%
I_{Rel1}[%]	6,8	±0,4	5,1%
A_2[kCnts/Chnl]	7,816	±0,060	0,8%
τ_2[ns]	8,081	±0,035	0,4%
I_2[kCnts]	1 973,6	±9,9	0,5%
A_{Rel2}[%]	68,3	±0,7	1,0%
I_{Rel2}[%]	93,3	±0,4	0,4%
$Bkgr_{Dec}$[kCnts]	0,0016	±0,0007	46%
$Bkgr_{IRF}$[Cnts/Chnl]	3,0000	---	---
$Shift_{IRF}$[ps]	100,0000	---	---
τ_{AvInt}[ns]	7,618	±0,029	0,4%
τ_{AvAmp}[ns]	5,918	±0,086	1,5%

Figure S46: Raw time-resolved photoluminescence decay of **Y8** in a liquid THF/H_2O 40/60 mixture at r.t. (blue) with instrumental response function in red (left), including the residuals (λ_{ex} = 440.0 nm, λ_{em} = 557 nm); fitting parameters including pre-exponential factors and confidence limits (right).

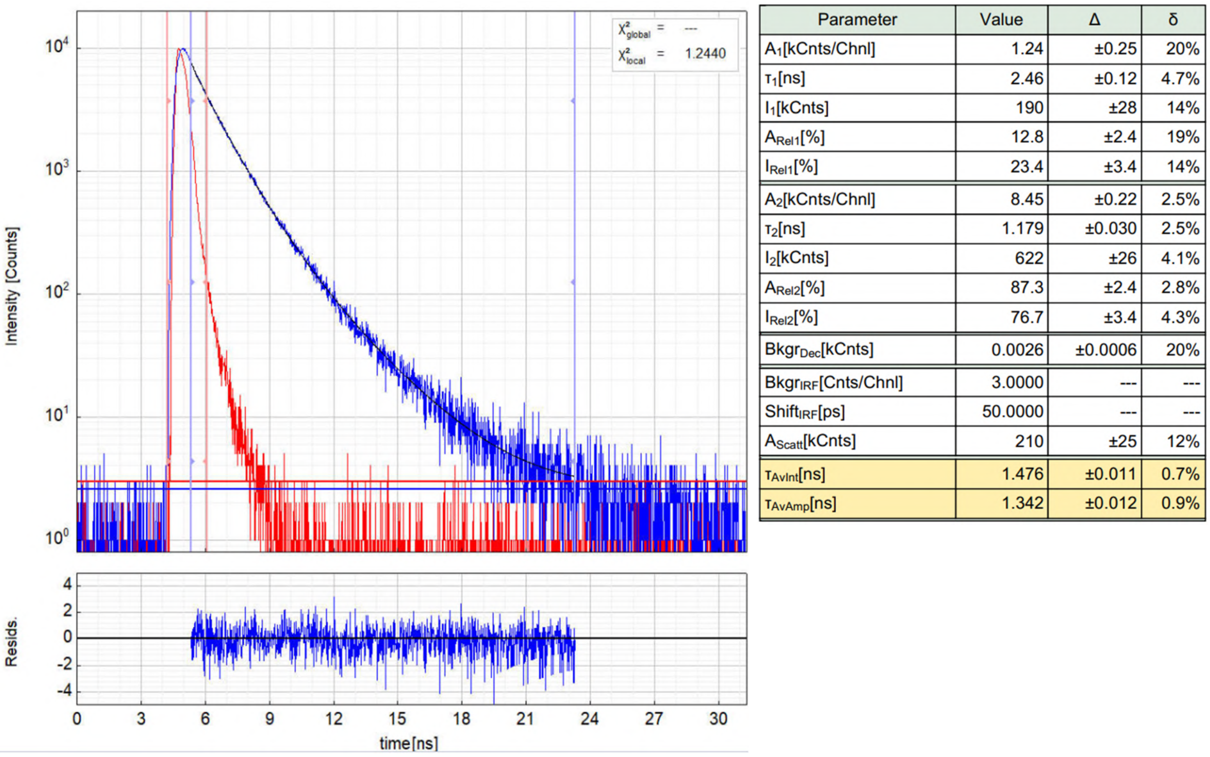

Parameter	Value	Δ	δ
A_1[kCnts/Chnl]	1.24	±0.25	20%
τ_1[ns]	2.46	±0.12	4.7%
I_1[kCnts]	190	±28	14%
A_{Rel1}[%]	12.8	±2.4	19%
I_{Rel1}[%]	23.4	±3.4	14%
A_2[kCnts/Chnl]	8.45	±0.22	2.5%
τ_2[ns]	1.179	±0.030	2.5%
I_2[kCnts]	622	±26	4.1%
A_{Rel2}[%]	87.3	±2.4	2.8%
I_{Rel2}[%]	76.7	±3.4	4.3%
$Bkgr_{Dec}$[kCnts]	0.0026	±0.0006	20%
$Bkgr_{IRF}$[Cnts/Chnl]	3.0000	---	---
$Shift_{IRF}$[ps]	50.0000	---	---
A_{Scatt}[kCnts]	210	±25	12%
τ_{AvInt}[ns]	1.476	±0.011	0.7%
τ_{AvAmp}[ns]	1.342	±0.012	0.9%

Figure S47: Raw time-resolved photoluminescence decay of **Y8** in a liquid THF/H_2O 1/99 mixture at r.t. (blue) with instrumental response function in red (left), including the residuals (λ_{ex} = 440.0 nm, λ_{em} = 557 nm); fitting parameters including pre-exponential factors and confidence limits (right).

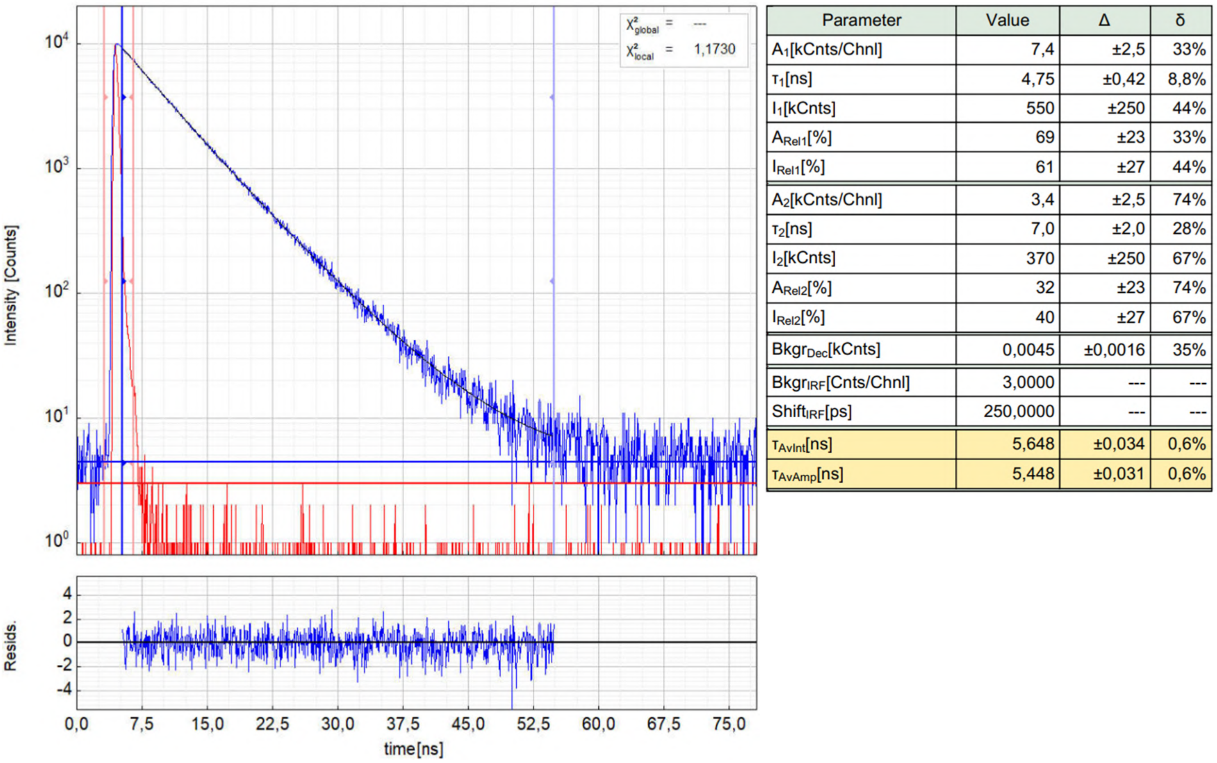

Parameter	Value	Δ	δ
A_1[kCnts/Chnl]	7,4	±2,5	33%
τ_1[ns]	4,75	±0,42	8,8%
I_1[kCnts]	550	±250	44%
A_{Rel1}[%]	69	±23	33%
I_{Rel1}[%]	61	±27	44%
A_2[kCnts/Chnl]	3,4	±2,5	74%
τ_2[ns]	7,0	±2,0	28%
I_2[kCnts]	370	±250	67%
A_{Rel2}[%]	32	±23	74%
I_{Rel2}[%]	40	±27	67%
$Bkgr_{Dec}$[kCnts]	0,0045	±0,0016	35%
$Bkgr_{IRF}$[Cnts/Chnl]	3,0000	---	---
$Shift_{IRF}$[ps]	250,0000	---	---
τ_{AvInt}[ns]	5,648	±0,034	0,6%
τ_{AvAmp}[ns]	5,448	±0,031	0,6%

Figure S48: Raw time-resolved photoluminescence decay of **G8** in a liquid THF/H_2O 40/60 mixture at r.t. (blue) with instrumental response function in red (left), including the residuals (λ_{ex} = 402.6 nm, λ_{em} = 505 nm); fitting parameters including pre-exponential factors and confidence limits (right).

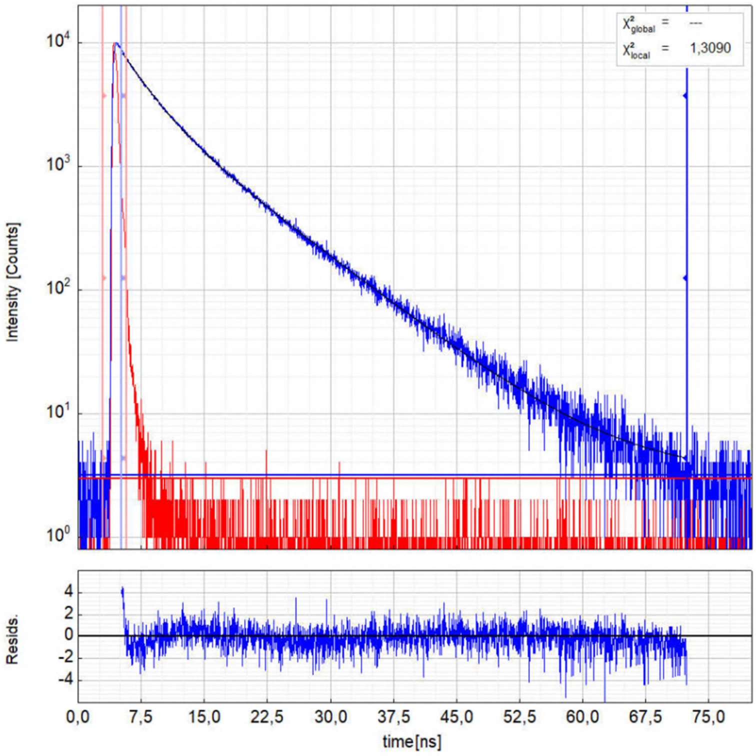

Parameter	Value	Δ	δ
A_1[kCnts/Chnl]	3,897	±0,089	2,3%
τ_1[ns]	8,392	±0,067	0,8%
I_1[kCnts]	1 022	±16	1,5%
A_{Rel1}[%]	38,1	±0,6	1,5%
I_{Rel1}[%]	62,8	±0,9	1,3%
A_2[kCnts/Chnl]	6,346	±0,052	0,8%
τ_2[ns]	3,062	±0,068	2,2%
I_2[kCnts]	608	±12	1,9%
A_{Rel2}[%]	62,0	±0,6	0,9%
I_{Rel2}[%]	37,3	±0,9	2,2%
$Bkgr_{Dec}$[kCnts]	0,0032	±0,0002	4,7%
$Bkgr_{IRF}$[Cnts/Chnl]	3,0000	---	---
$Shift_{IRF}$[ps]	100,0000	---	---
τ_{AvInt}[ns]	6,406	±0,020	0,3%
τ_{AvAmp}[ns]	5,090	±0,045	0,9%

Figure S49: Raw time-resolved photoluminescence decay of **G8** in a liquid THF/H_2O 1/99 mixture at r.t. (blue) with instrumental response function in red (left), including the residuals (λ_{ex} = 402.6 nm, λ_{em} = 526 nm); fitting parameters including pre-exponential factors and confidence limits (right).

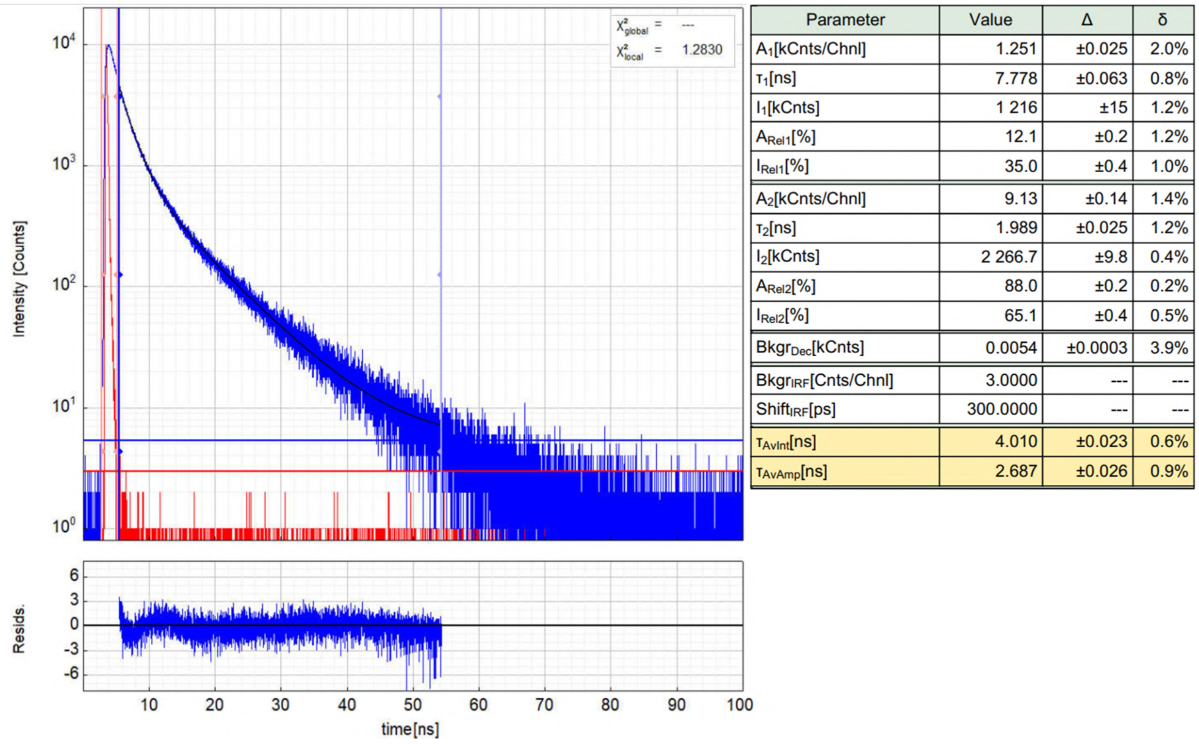

Parameter	Value	Δ	δ
A_1[kCnts/Chnl]	1.251	±0.025	2.0%
τ_1[ns]	7.778	±0.063	0.8%
I_1[kCnts]	1 216	±15	1.2%
A_{Rel1}[%]	12.1	±0.2	1.2%
I_{Rel1}[%]	35.0	±0.4	1.0%
A_2[kCnts/Chnl]	9.13	±0.14	1.4%
τ_2[ns]	1.989	±0.025	1.2%
I_2[kCnts]	2 266.7	±9.8	0.4%
A_{Rel2}[%]	88.0	±0.2	0.2%
I_{Rel2}[%]	65.1	±0.4	0.5%
$Bkgr_{Dec}$[kCnts]	0.0054	±0.0003	3.9%
$Bkgr_{IRF}$[Cnts/Chnl]	3.0000	---	---
$Shift_{IRF}$[ps]	300.0000	---	---
τ_{AvInt}[ns]	4.010	±0.023	0.6%
τ_{AvAmp}[ns]	2.687	±0.026	0.9%

Figure S50: Raw time-resolved photoluminescence decay of **R8** in MSNs at r.t. (powder) (blue) with instrumental response function in red (left), including the residuals (λ_{ex} = 505.7 nm, λ_{em} = 585 nm); fitting parameters including pre-exponential factors and confidence limits (right).

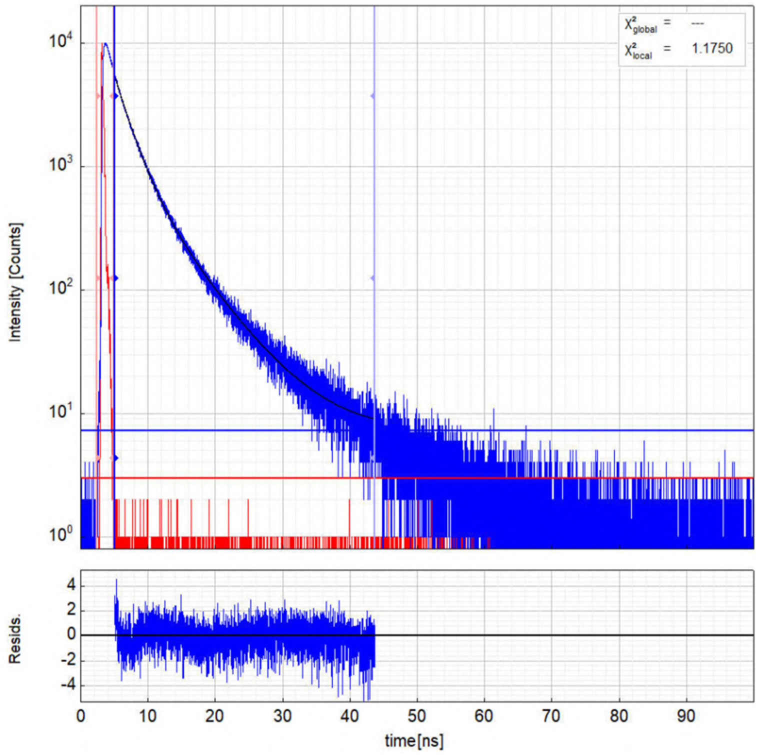

Parameter	Value	Δ	δ
A_1[kCnts/Chnl]	9.847	±0.080	0.8%
τ_1[ns]	2.144	±0.032	1.5%
I_1[kCnts]	2 638	±29	1.1%
A_{Rel1}[%]	86.7	±0.6	0.6%
I_{Rel1}[%]	70.0	±1.0	1.4%
A_2[kCnts/Chnl]	1.517	±0.080	5.2%
τ_2[ns]	5.98	±0.11	1.8%
I_2[kCnts]	1 133	±40	3.5%
A_{Rel2}[%]	13.4	±0.6	4.2%
I_{Rel2}[%]	30.1	±1.0	3.2%
$Bkgr_{Dec}$[kCnts]	0.0073	±0.0004	4.9%
$Bkgr_{IRF}$[Cnts/Chnl]	3.0000	---	---
$Shift_{IRF}$[ps]	0.0000	---	---
τ_{AvInt}[ns]	3.293	±0.018	0.5%
τ_{AvAmp}[ns]	2.655	±0.021	0.8%

Figure S51: Raw time-resolved photoluminescence decay of **R8** in MSNs at r.t. (3D-printed) (blue) with instrumental response function in red (left), including the residuals (λ_{ex} = 505.7 nm, λ_{em} = 582 nm); fitting parameters including pre-exponential factors and confidence limits (right).

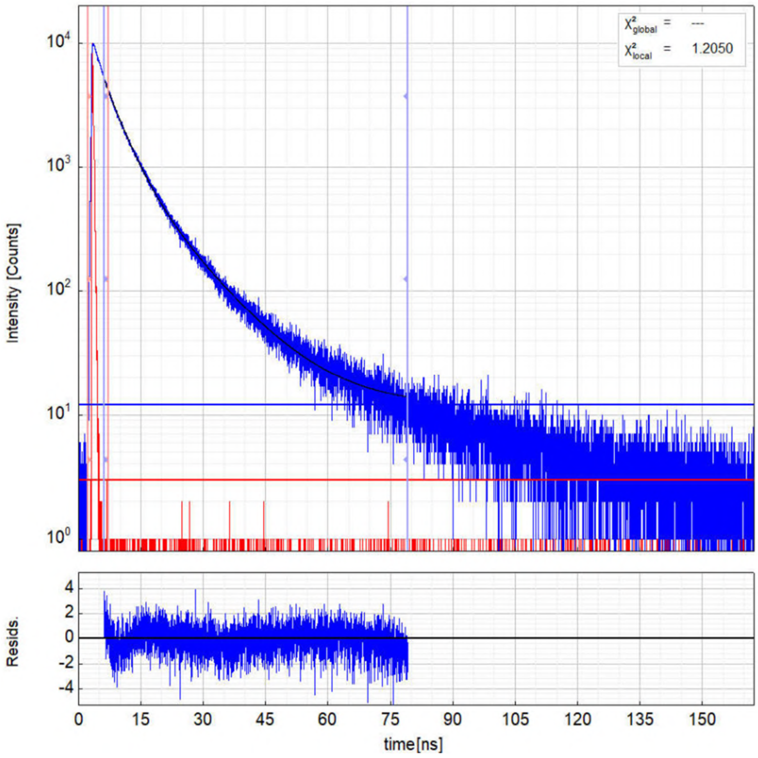

Parameter	Value	Δ	δ
A_1[kCnts/Chnl]	1.548	±0.092	5.9%
τ_1[ns]	11.36	±0.25	2.2%
I_1[kCnts]	1 099	±40	3.6%
A_{Rel1}[%]	17.0	±0.9	5.0%
I_{Rel1}[%]	36.3	±1.3	3.5%
A_2[kCnts/Chnl]	7.562	±0.057	0.7%
τ_2[ns]	4.095	±0.070	1.7%
I_2[kCnts]	1 935	±36	1.8%
A_{Rel2}[%]	83.1	±0.9	1.0%
I_{Rel2}[%]	63.8	±1.3	2.0%
$Bkgr_{Dec}$[kCnts]	0.0121	±0.0007	5.2%
$Bkgr_{IRF}$[Cnts/Chnl]	3.0000	---	---
$Shift_{IRF}$[ps]	250.0000	---	---
τ_{AvInt}[ns]	6.724	±0.046	0.7%
τ_{AvAmp}[ns]	5.328	±0.040	0.7%

Figure S52: Raw time-resolved photoluminescence decay of **Y8** in MSNs at r.t. (powder) (blue) with instrumental response function in red (left), including the residuals (λ_{ex} = 505.7 nm, λ_{em} = 555 nm); fitting parameters including pre-exponential factors and confidence limits (right).

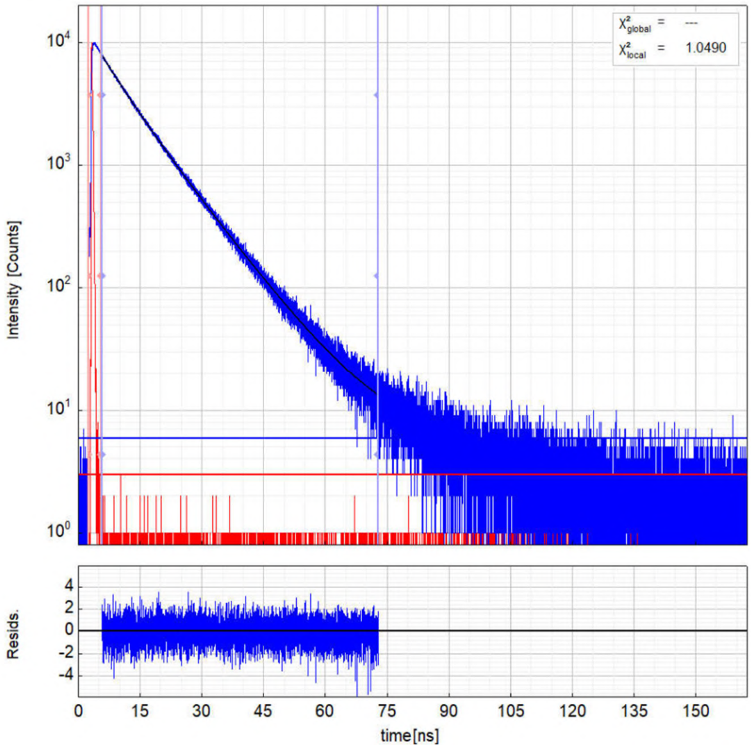

Parameter	Value	Δ	δ
A_1[kCnts/Chnl]	4.26	±0.46	11%
τ_1[ns]	5.89	±0.31	5.2%
I_1[kCnts]	3 130	±480	15%
A_{Rel1}[%]	40.2	±4.5	11%
I_{Rel1}[%]	27.8	±4.3	15%
A_2[kCnts/Chnl]	6.33	±0.50	7.8%
τ_2[ns]	10.32	±0.19	1.7%
I_2[kCnts]	8 160	±490	5.9%
A_{Rel2}[%]	59.9	±4.5	7.4%
I_{Rel2}[%]	72.3	±4.3	5.9%
$Bkgr_{Dec}$[kCnts]	0.0059	±0.0006	9.9%
$Bkgr_{IRF}$[Cnts/Chnl]	3.0000	---	---
$Shift_{IRF}$[ps]	120.0000	---	---
τ_{AvInt}[ns]	9.085	±0.021	0.2%
τ_{AvAmp}[ns]	8.534	±0.032	0.4%

Figure S53: Raw time-resolved photoluminescence decay of **Y8** in MSNs at r.t. (3D-printed) (blue) with instrumental response function in red (left), including the residuals (λ_{ex} = 505.7 nm, λ_{em} = 545 nm); fitting parameters including pre-exponential factors and confidence limits (right).

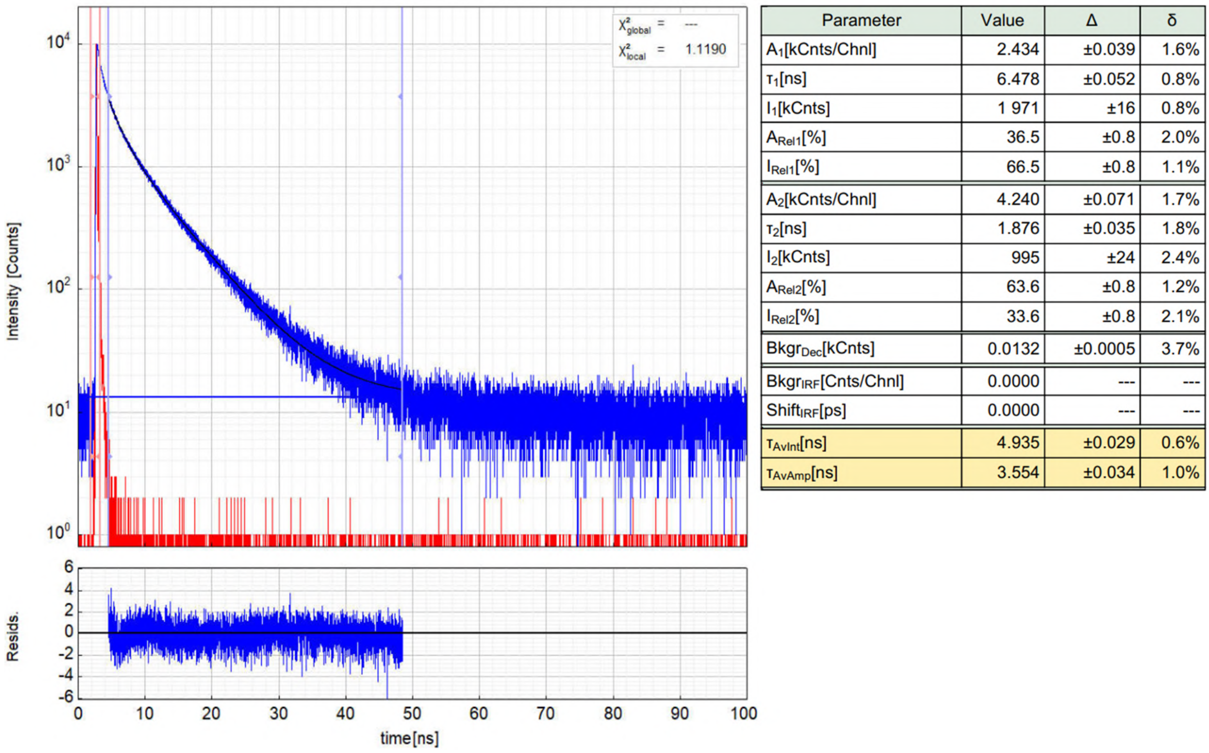

Parameter	Value	Δ	δ
A_1[kCnts/Chnl]	2.434	±0.039	1.6%
τ_1[ns]	6.478	±0.052	0.8%
I_1[kCnts]	1 971	±16	0.8%
A_{Rel1}[%]	36.5	±0.8	2.0%
I_{Rel1}[%]	66.5	±0.8	1.1%
A_2[kCnts/Chnl]	4.240	±0.071	1.7%
τ_2[ns]	1.876	±0.035	1.8%
I_2[kCnts]	995	±24	2.4%
A_{Rel2}[%]	63.6	±0.8	1.2%
I_{Rel2}[%]	33.6	±0.8	2.1%
$Bkgr_{Dec}$[kCnts]	0.0132	±0.0005	3.7%
$Bkgr_{IRF}$[Cnts/Chnl]	0.0000	---	---
$Shift_{IRF}$[ps]	0.0000	---	---
τ_{AvInt}[ns]	4.935	±0.029	0.6%
τ_{AvAmp}[ns]	3.554	±0.034	1.0%

Figure S54: Raw time-resolved photoluminescence decay of **G8** in MSNs at r.t. (powder) (blue) with instrumental response function in red (left), including the residuals (λ_{ex} = 402.6 nm, λ_{em} = 508 nm); fitting parameters including pre-exponential factors and confidence limits (right).

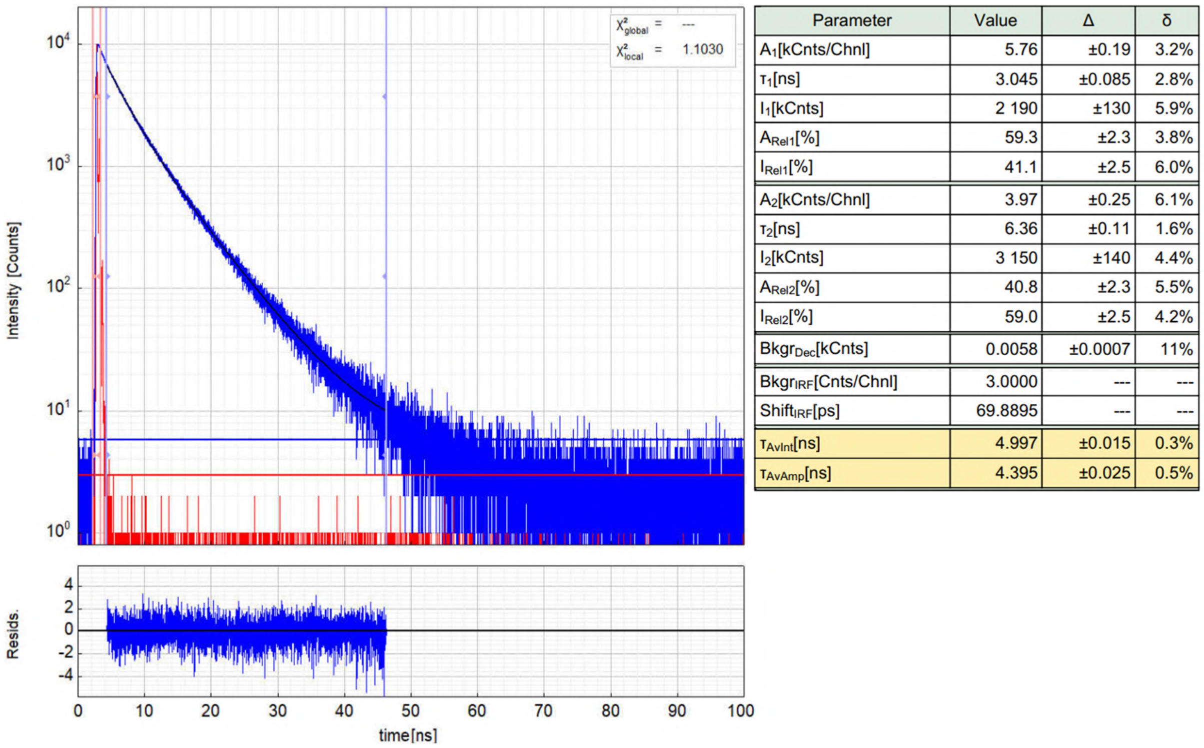

Parameter	Value	Δ	δ
A_1[kCnts/Chnl]	5.76	±0.19	3.2%
τ_1[ns]	3.045	±0.085	2.8%
I_1[kCnts]	2 190	±130	5.9%
A_{Rel1}[%]	59.3	±2.3	3.8%
I_{Rel1}[%]	41.1	±2.5	6.0%
A_2[kCnts/Chnl]	3.97	±0.25	6.1%
τ_2[ns]	6.36	±0.11	1.6%
I_2[kCnts]	3 150	±140	4.4%
A_{Rel2}[%]	40.8	±2.3	5.5%
I_{Rel2}[%]	59.0	±2.5	4.2%
$Bkgr_{Dec}$[kCnts]	0.0058	±0.0007	11%
$Bkgr_{IRF}$[Cnts/Chnl]	3.0000	---	---
$Shift_{IRF}$[ps]	69.8895	---	---
τ_{AvInt}[ns]	4.997	±0.015	0.3%
τ_{AvAmp}[ns]	4.395	±0.025	0.5%

Figure S55: Raw time-resolved photoluminescence decay of **G8** in MSNs at r.t. (3D-printed) (blue) with instrumental response function in red (left), including the residuals (λ_{ex} = 402.6 nm, λ_{em} = 504 nm); fitting parameters including pre-exponential factors and confidence limits (right).

4 SCANNING ELECTRON MICROSCOPY (SEM)

Scanning electron microscopy was performed using a *ZEISS Crossbeam 540*. The samples were sputtered with gold and palladium using a *Leica EM ACE 600 Sputter Coater*. SEM images of MSN powders without luminophores were recently examined.[3]

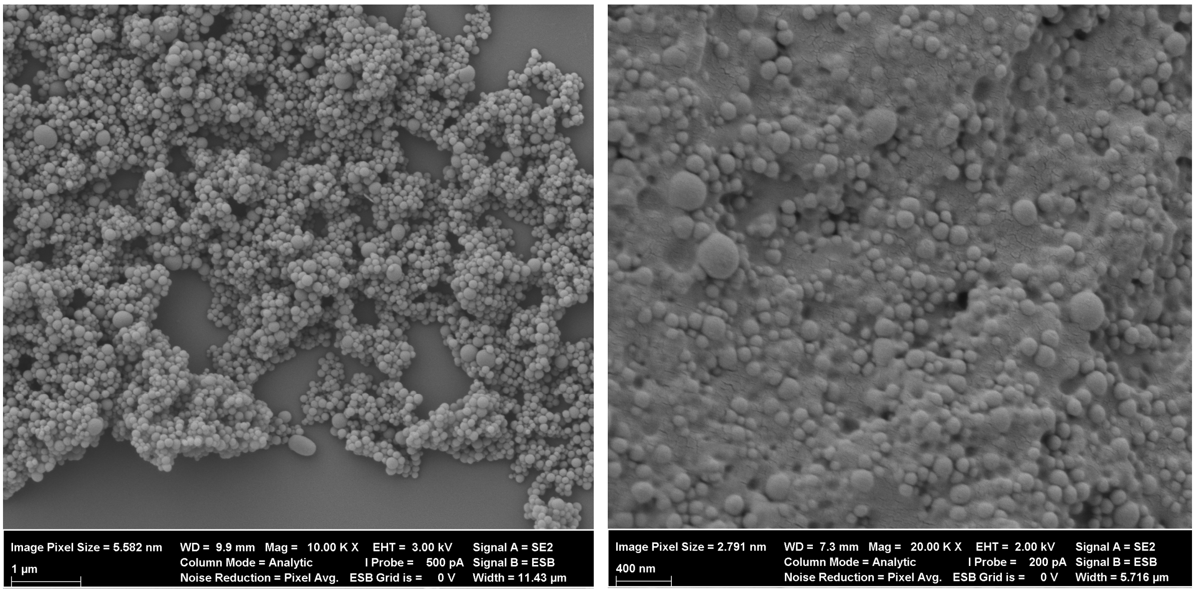

Figure S56: SEM images of MSNs of **R8** as powders (left) and 3D-printed material (right).

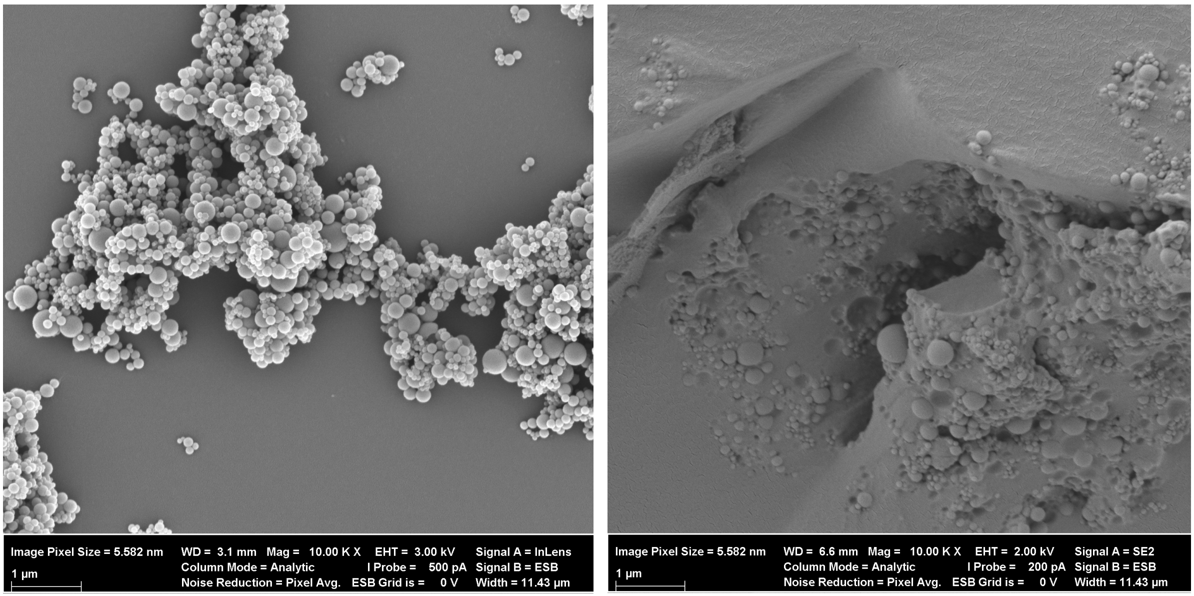

Figure S57: SEM images of MSNs of **Y8** as powders (left) and 3D-printed material (right).

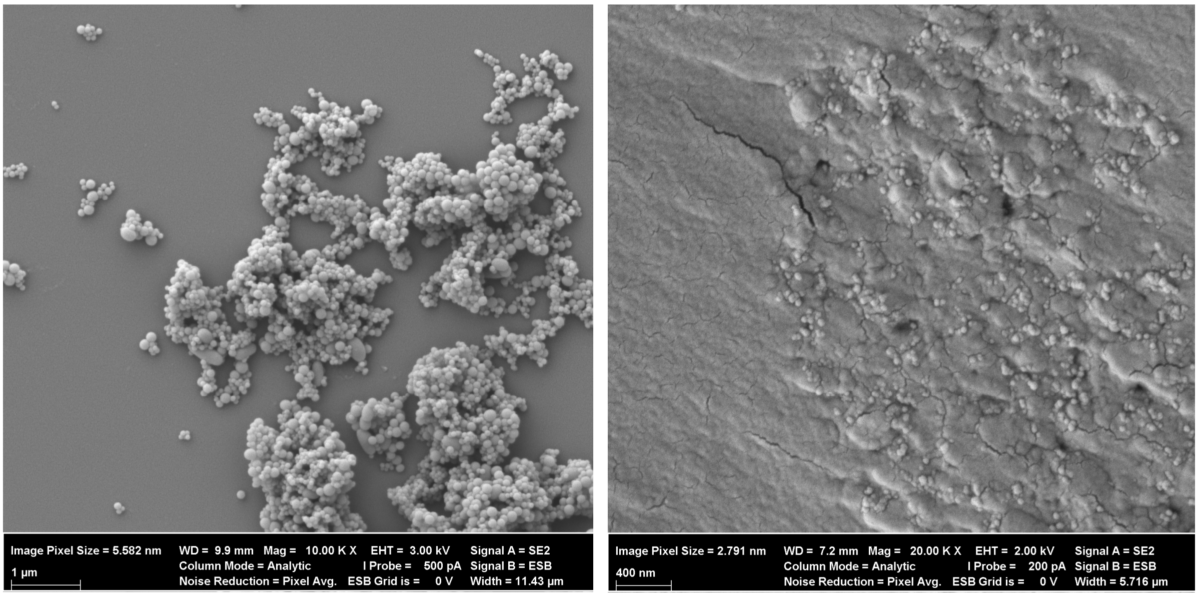

Figure S58: SEM images of MSNs of **G8** as powders (left) and 3D-printed material (right).

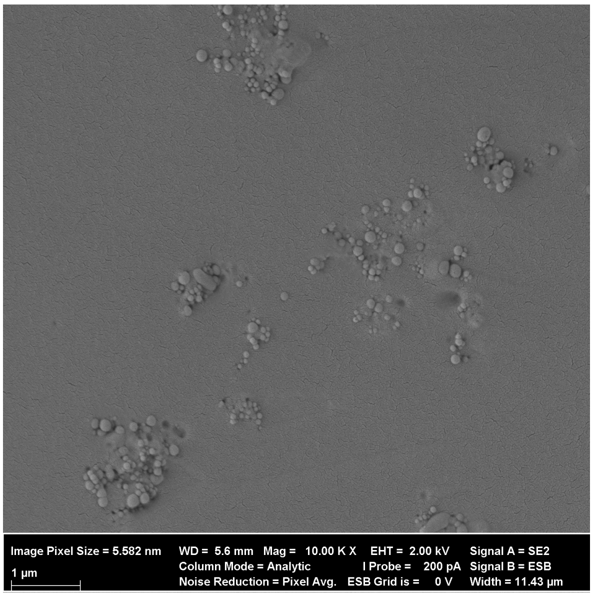

Figure S59: SEM images of 3D-printed MSNs without additive. See ref. 3 for SEM images of the MSN powders.[3]

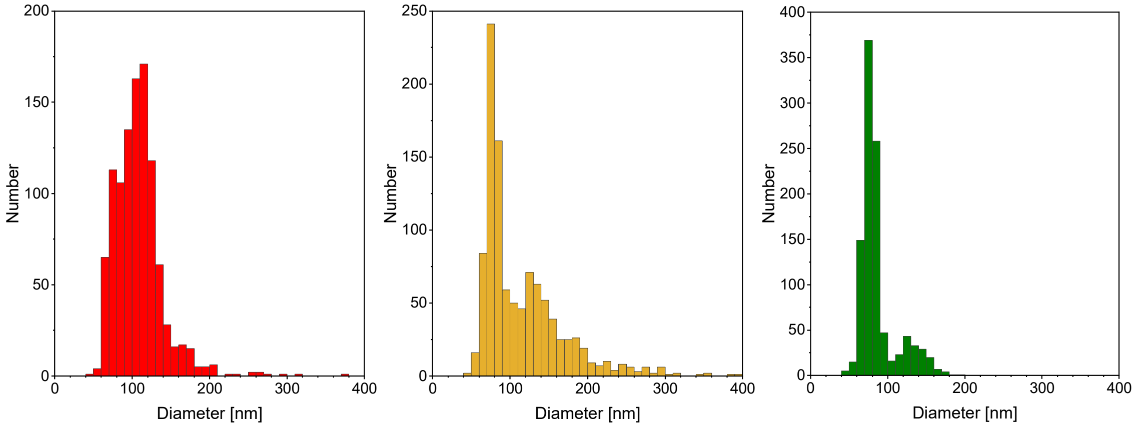

Figure S60: Histograms of the size distribution of the luminescent MSNs containing **R8** (left), **Y8** (center), and **G8** (right).

5 LIQUID CRYSTAL (LC) SECTION

As the hosts, E7 (*Synthon Chemicals*) or 8CB (*TCI*) were chosen. 8CB is the abbreviation for 4'-octyl-4-cyanobiphenyl. E7 consists of 51% 4'-pentyl-4-cyanobiphenyl (5CB), 25% 4'-heptyl-4-cyanobiphenyl (7CB), 16% 4'-octylocy-4-cyanobiphenyl (8OCB), and 8% 4-cyano-4'-pentylterphenyl (5CT).

LC EXPERIMENTS

For the LC experiments, samples were prepared by dissolving the host (E7 or 8CB) as well as an emitter (**R8**, **Y8** or **G8**) in DCM and pipetting the required amounts using an *Eppendorf* Multipette E3x. After the solvent was removed and the samples dried *in vacuo* for at least 15 minutes, the liquid crystalline materials were obtained. The sample were filled into polyimide coated sandwich cells for LC evaluation (KSRO-05/B511P7NSS; E.H.C Co. Ltd.) at 100 °C by using capillary forces. Afterwards it was cooled down to the first temperature e.g. 20 °C. The cell was placed on a Linkam Heating stage (LTS420) within a self-built benchtop fluorescence spectrometer setup, as seen schematically in Fig. S54. A thin black polypropylene foil was set underneath the sample to reduce the light scattering. Cells were used to ensure better allignment and prevent the film from thinning during heating, which would also result in a lower fluorescence intensity.

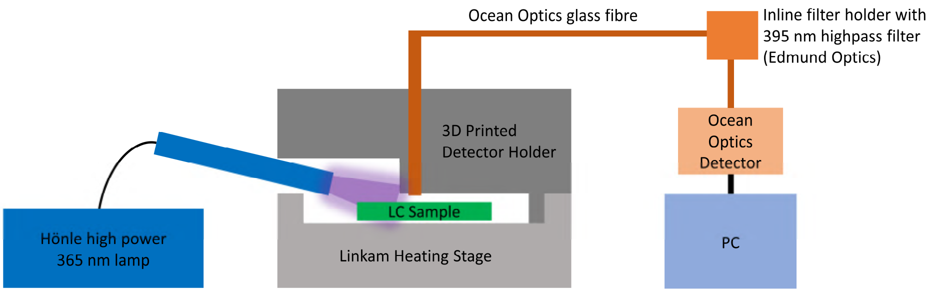

Figure S61: Schematic overview of the experimental setup for LC fluorescence measurements.

Irradiation occurred at 35% of maximum intensity with a *Hönle* 365 nm LED lamp (*Bluepoint-LED Eco*) at a roughly 45° angle. The setup was fixed with a tripod and stayed identical for each measurement and for the same sample. Fluorescence spectra were recorded using an *Ocean Optics* spectrometer (*FLAME-S-XR1-ES*) connected to a PC. The light was transferred by an Ocean Optics glass fibre, including an inline filter holder equipped with a 395 nm highpass filter from *Edmund Optics* to filter out the excitation wavelength. In this way, an optimal temperature control over the LC sample was realized with the only downside being that excitation had to occur at 365 nm and that the intensity of the light had to be relatively high due to the sensitivity of this detector setup.

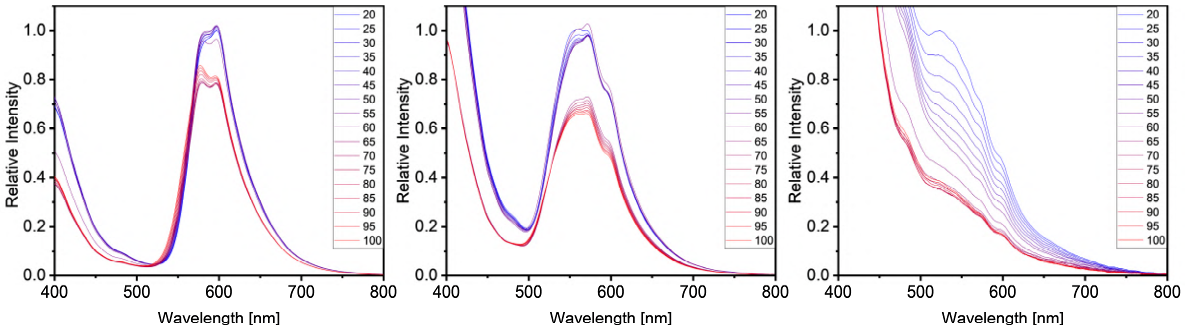

Figure S62: Plots of relative fluorescence intensity at different temperatures during heating of **R8** (left), **Y8** (center) and **G8** (right) in E7.

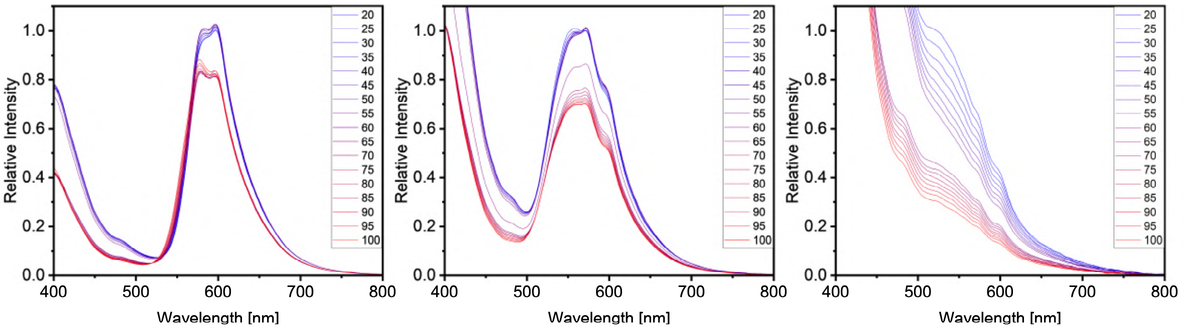

Figure S63: Plots of relative fluorescence intensity at different temperatures during cooling of **R8** (left), **Y8** (center) and **G8** (right) in E7.

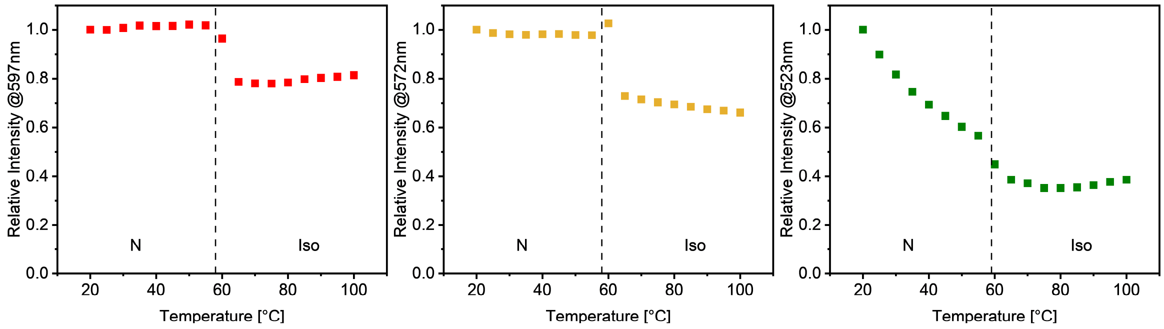

Figure S64: Plots of relative fluorescence intensity at the maximum versus the temperature during heating of **R8** (left), **Y8** (center) and **G8** (right) in E7.

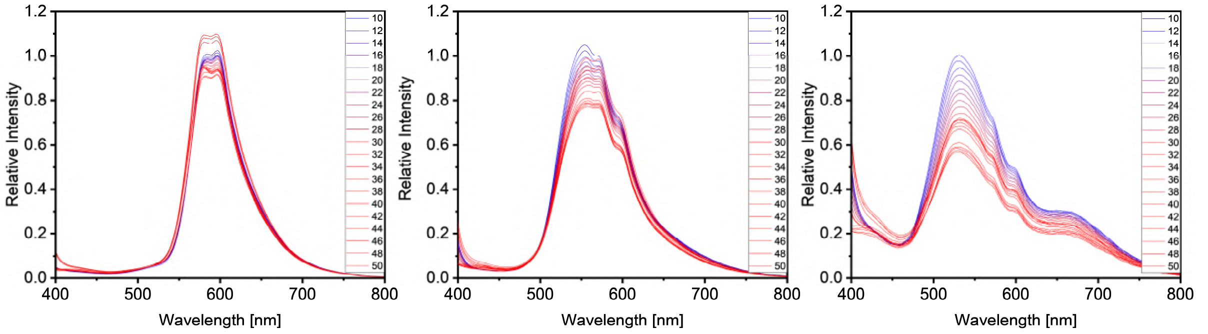

Figure S65: Plots of relative fluorescence intensity at different temperatures during heating of **R8** (left), **Y8** (center) and **G8** (right) in 8CB.

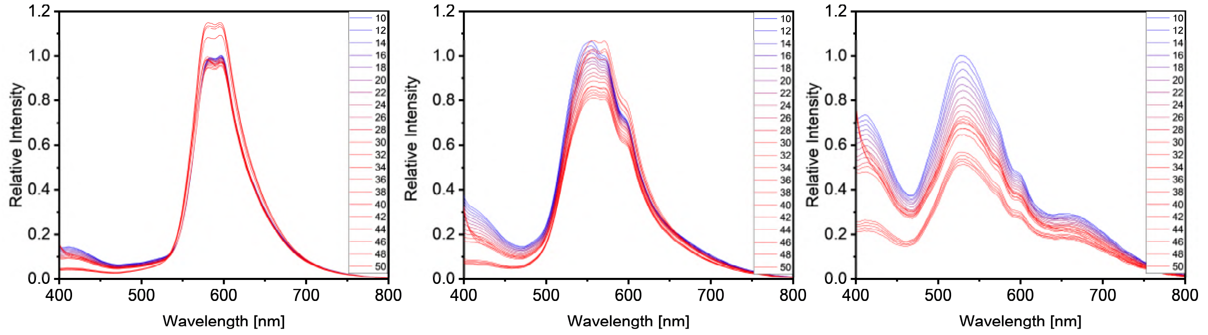

Figure S66: Plots of relative fluorescence intensity at different temperatures during cooling of **R8** (left), **Y8** (center) and **G8** (right) in 8CB.

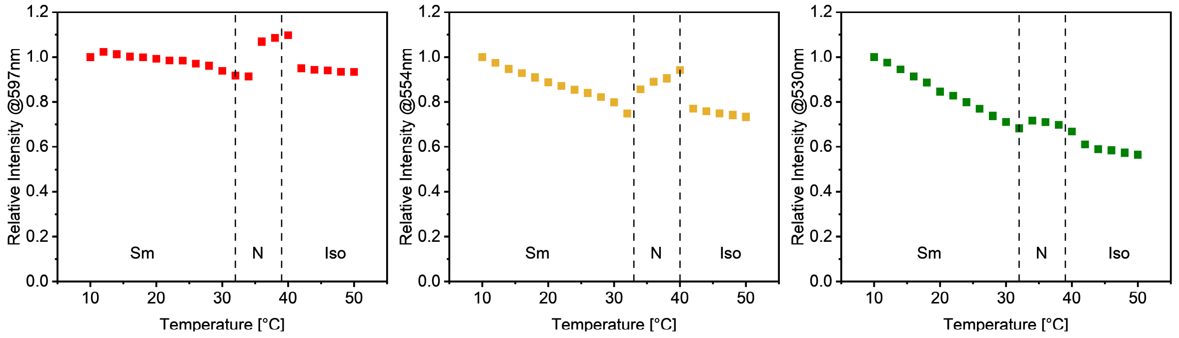

Figure S67: Plots of relative fluorescence intensity at the maximum versus the temperature during heating of **R8** (left), **Y8** (center) and **G8** (right) in 8CB.

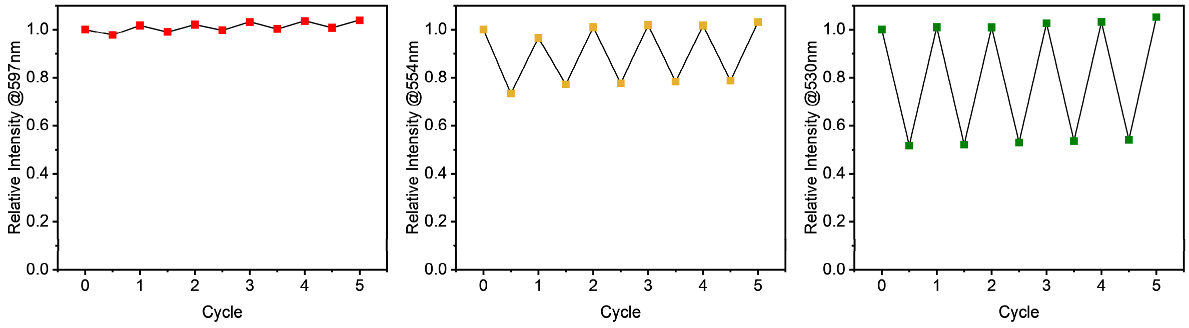

Figure S68: Plots of relative fluorescence intensity at the maximum versus the number of cycles of **R8** (left), **Y8** (center) and **G8** (right) in 8CB.

Differential Scanning Calorimetry (DSC)

Differential Scanning Calorimetry (DSC) curves were obtained using a *DSC 3+ Star^{e} System* from *Mettler Toledo* under a nitrogen atmosphere. For each mixture, 2-6 mg of compound was weighed into a 40 µL standard aluminium crucible. To secure reproducibility, each mixture was measured in triplicates. All samples were treated the same way and heated and cooled at 10 K/min between 0 and 100 °C in three consecutive cycles. The peak positions of the second heating and cooling cycles were used to determine phase transitions, and the average of all triplicates was used.

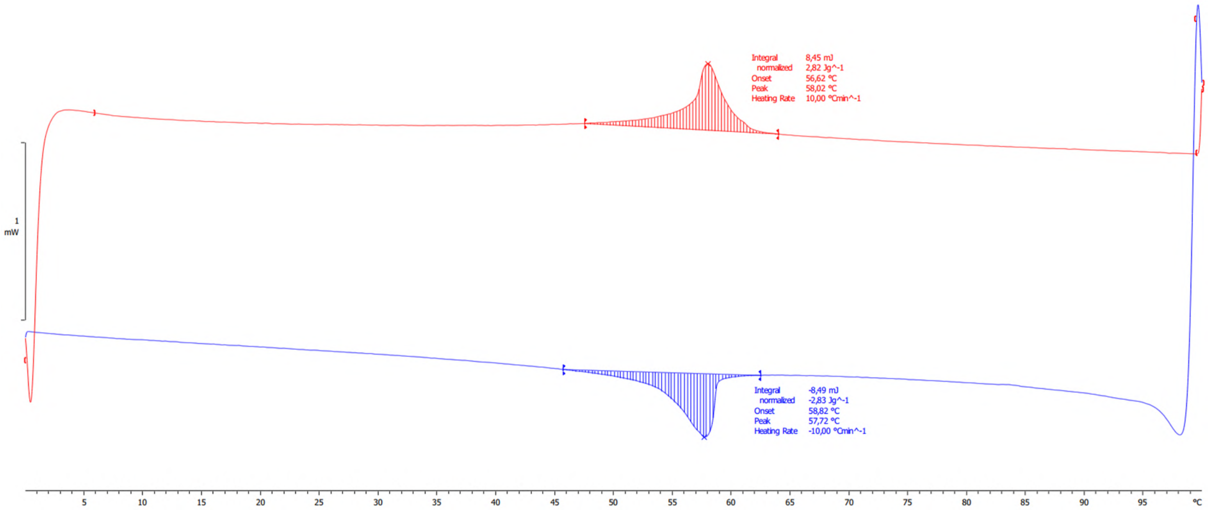

Figure S69: Representative heating and cooling cycle of **R8** in E7 (endo up). Mixtures were measured in triplicates.

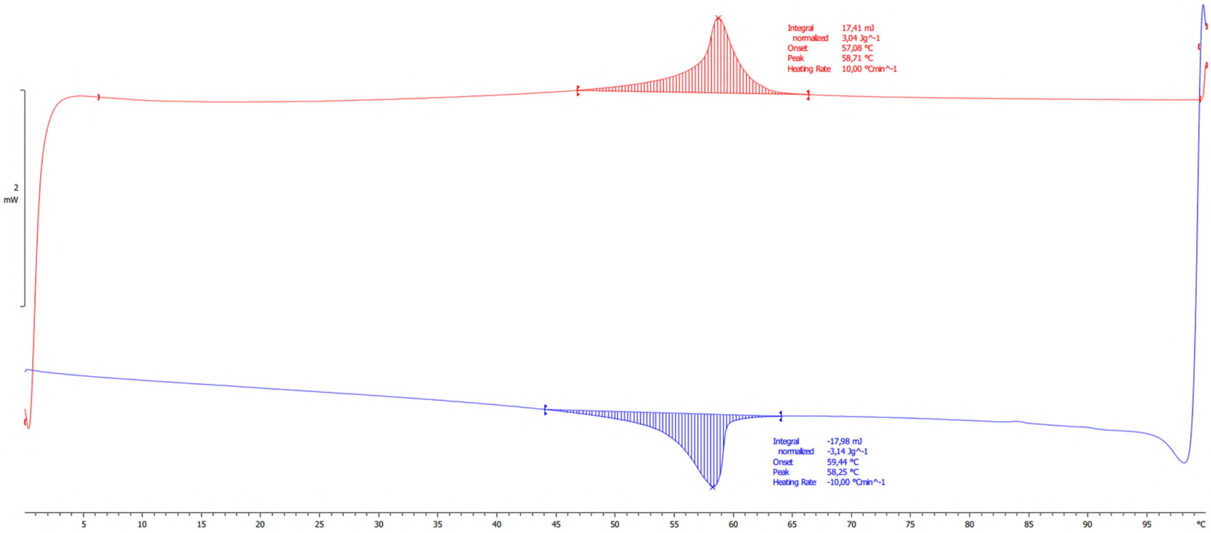

Figure S70: Representative heating and cooling cycle of **Y8** in E7 (endo up). Mixtures were measured in triplicates.

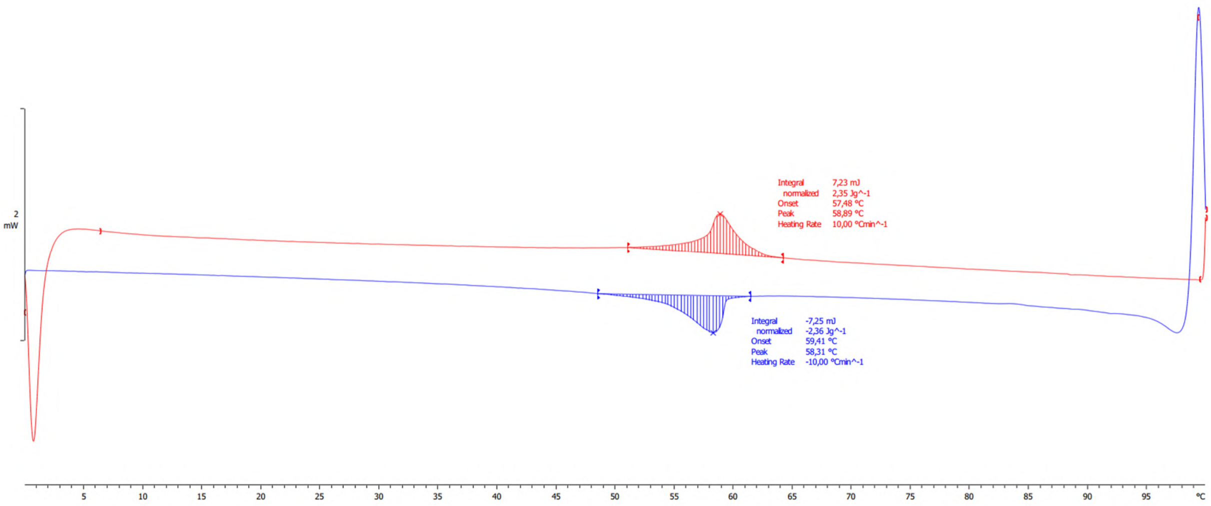

Figure S71: Representative heating and cooling cycle of **G8** in E7 (endo up). Mixtures were measured in triplicates.

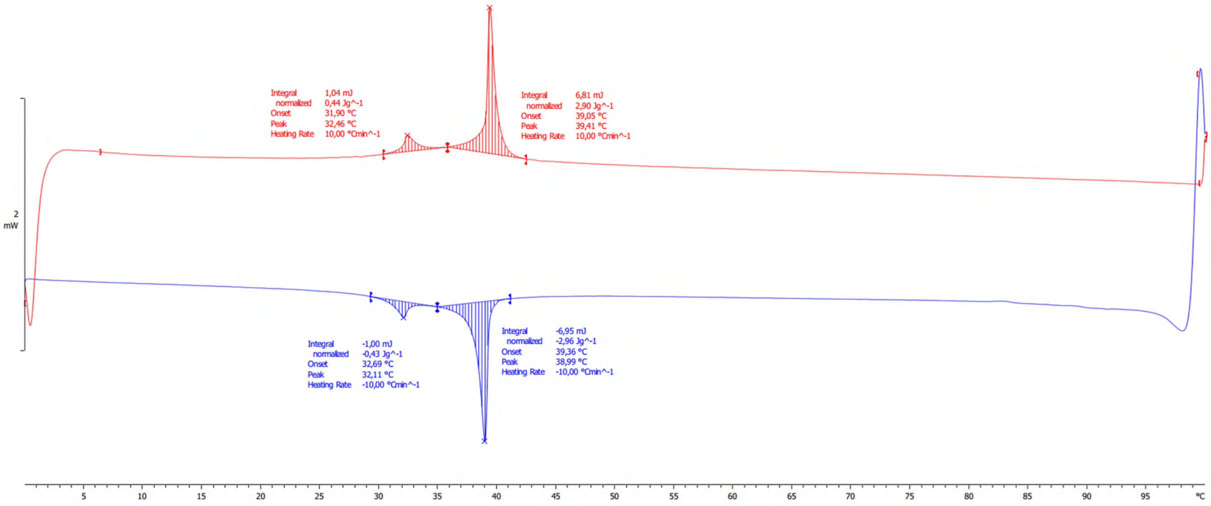

Figure S72: Representative heating and cooling cycle of **R8** in 8CB (endo up). Mixtures were measured in triplicates.

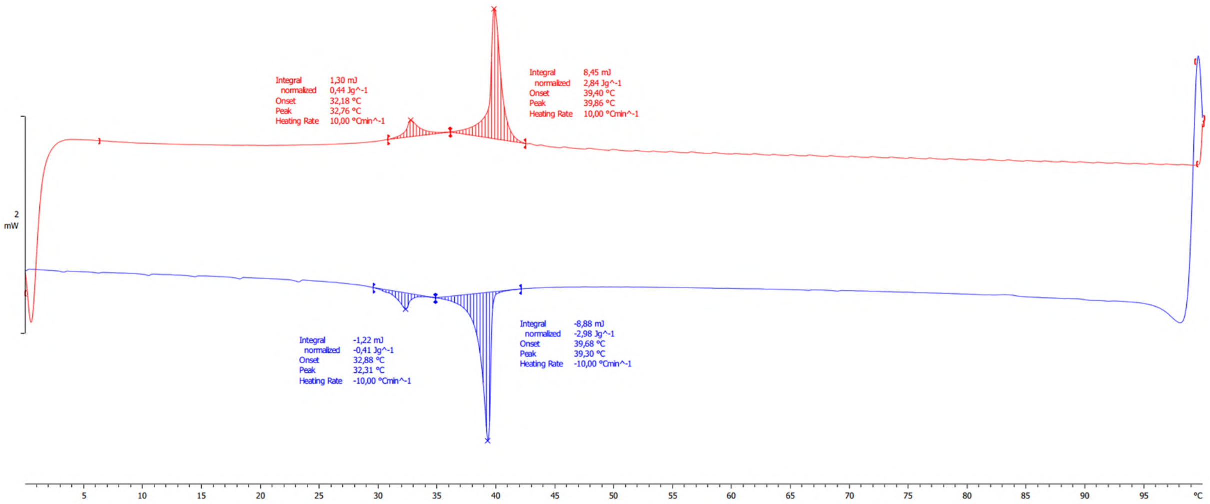

Figure S73: Representative heating and cooling cycle of **Y8** in 8CB (endo up). Mixtures were measured in triplicates.

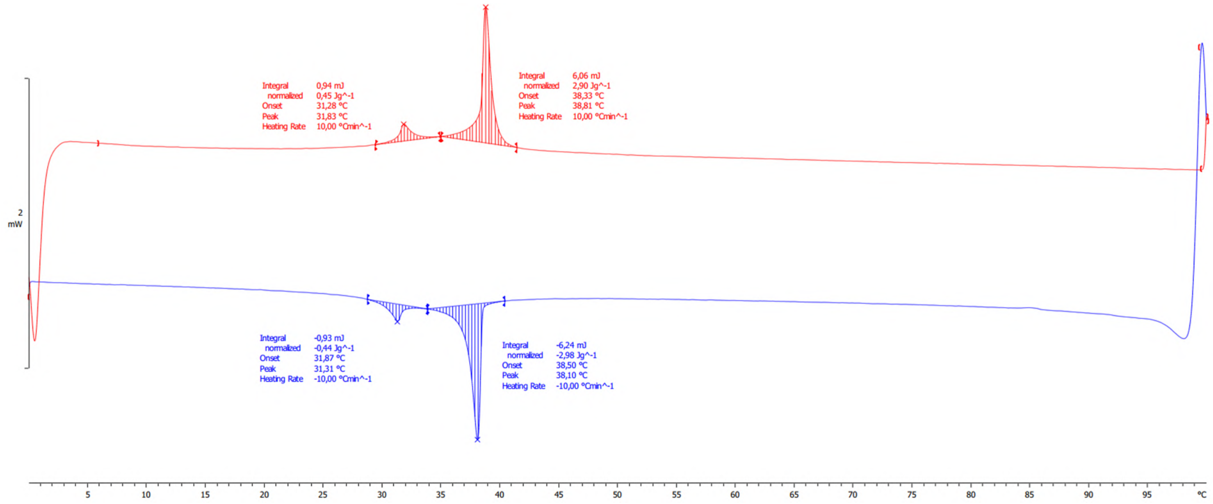

Figure S74: Representative heating and cooling cycle of **G8** in 8CB (endo up). Mixtures were measured in triplicates.

Polarized Optical Microscopy (POM)

Polarized optical microscopy was performed with polyimide-coated sandwich cells for LC evaluation (KSRO-05/B511P7NSS; *E.H.C Co. Ltd.*) on a *Nikon* Eclipse Ni microscope, equipped with a *Linkham* LTS420 heating stage under crossed polarizers. Micrographs were recorded using an OptixCam Summit K2 OCS-D3K4-14 camera and were obtained after the fluorescence measurements while cooling from the isotropic melt with a cooling rate of 10 K/min.

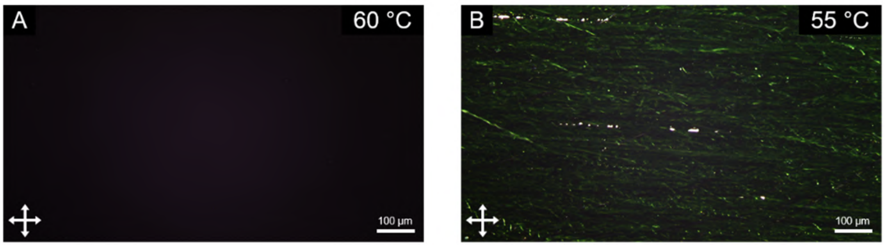

Figure S75: POM images of **R8** in E7 (0.1 mol%) upon cooling from the isotropic phase (A) to the nematic phase (B).

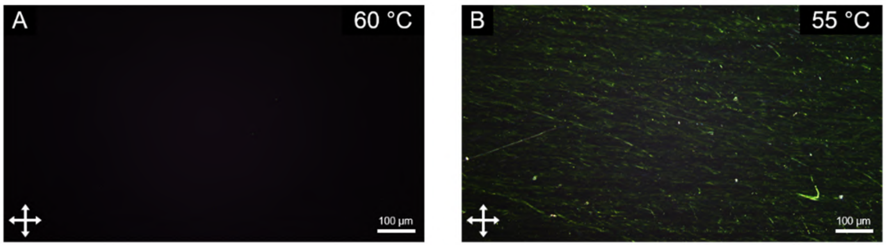

Figure S76: POM images of **Y8** in E7 (0.1 mol%) upon cooling from the isotropic phase (A) to the nematic phase (B).

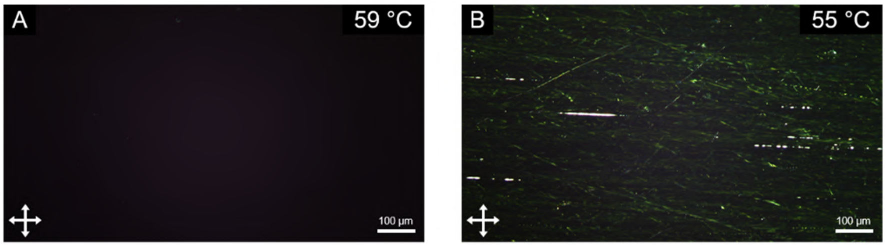

Figure S77: POM images of **G8** in E7 (0.1 mol%) upon cooling from the isotropic phase (A) to the nematic phase (B).

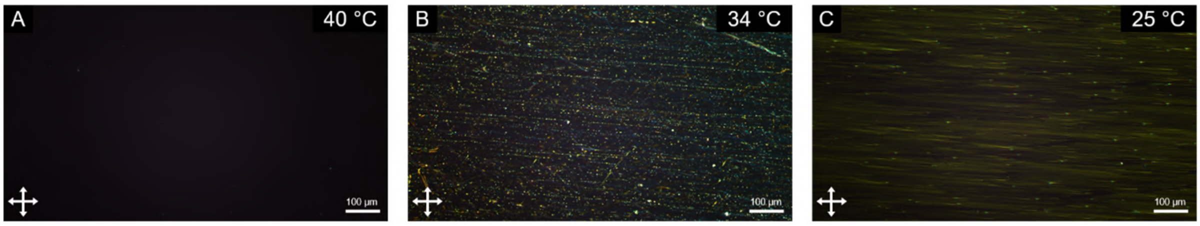

Figure S78: POM images of **R8** in 8CB (0.05 mol%) upon cooling from the isotropic phase (A) to the nematic phase (B) and smectic phase (C).

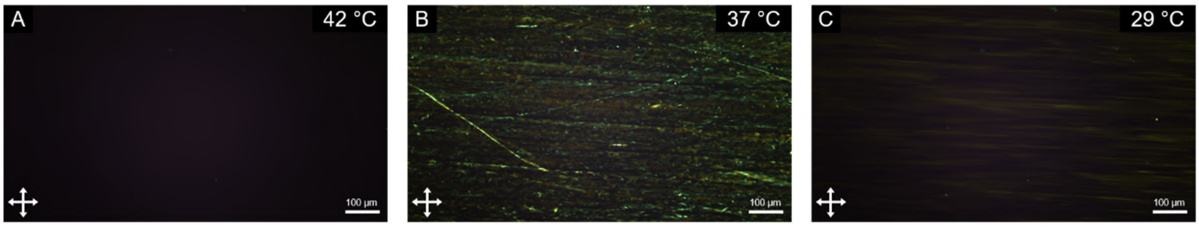

Figure S79: POM images of **Y8** in 8CB (0.05 mol%) upon cooling from the isotropic phase (A) to the nematic phase (B) and smectic phase (C).

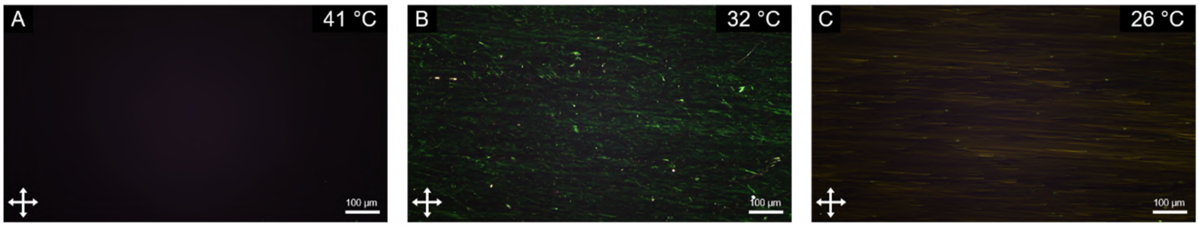

Figure S80: POM images of **G8** in 8CB (0.05 mol%) upon cooling from the isotropic phase (A) to the nematic phase (B) and smectic phase (C).

6 X-RAY DIFFRACTOMETRIC ANALYSIS ON SINGLE CRYSTALS

Crystals of **R2**, **Y2**, **G0,** and phenoxazine precursors **4c** and **4d** were measured using X-ray diffractometric analysis. Graphics were created using *Mercury 3.0*.[4]

For compounds **Y2**, **4c,** and **4d**, the following conditions were used. The data sets were collected with *a Bruker D8 Venture Photon III* Diffractometer (*APEX4* Version 2021.4-0,[1] *Bruker AXS Inc*., 2021) with a MX mirror monochromator with either a micro focus tube Mo ImS ($Mo_{K\alpha}$, λ = 0.71073 Å) (for **Y2**) or with a micro focus tube Cu ImS ($Cu_{K\alpha}$, λ = 1.54178 Å) (for **4c**, **4d**). Cell refinement and data reduction were performed using *SAINT* Version 8.40B (*Bruker AXS Inc*., 2021). The frames were integrated with the Bruker SAINT software package using a wide-frame algorithm for **4c, 4d** and a narrow-frame algorithm for **Y2**. The absorption was corrected using *SADABS* Version 2016/2 (*Bruker AXS Inc*., 2021); the structures were solved using *SHELXT*-Version 2018-3 and refined using *SHELXL*- Version 2018-3.[5,6]

For compounds **R2** and **G0**, the following conditions were used. The crystals were mounted on nylon loops in inert oil. The data set for compound **G0** was collected with a *Bruker AXS D8 Kappa* diffractometer with APEX2 detector (monochromated $Mo_{K\alpha}$ radiation, λ = 0.71073 Å) at 100(2) K. The data set for compound **R2** was collected on a *Bruker AXS D8 Venture* diffractometer with Photon II detector (monochromated $Cu_{K\alpha}$ radiation, λ = 1.54178 Å, microfocus source) at 100(2) K (*APEX3* Version 2019.1-0, *Bruker AXS Inc*., 2019). and for compounds **R2** and **G0** with *SAINT* Version 8.40A (*Bruker AXS Inc*., 2019). Absorption corrections were performed semi-empirically from equivalent reflections based on multi-scans (*SADABS* Version 2016/2, *Bruker AXS Inc*., 2019). The structures **R2** and **G0** were solved using Direct Methods (*SHELXS-2013*)[7] and refined anisotropically by full-matrix least-squares on F^2 (*SHELXL-2017*).[6,8] Hydrogen atoms were refined using a riding model or rigid methyl groups. The structure of **G0** (alh_fr18am) shows pseudo translational symmetry along *b*. Reducing the unit cell size leads to a model with significantly larger *R* values and enlarged displacement ellipsoids of the phenyl group. Simulated precession images support the choice of a larger cell.

Deposition numbers 2429052 (for **R2**), 2427615 (for **Y2**), 2429053 (for **G0**), 2427616 (for **4c**), and 2427617 (for **4d**) contain the supplementary crystallographic data for this paper. These data are provided free of charge by the joint Cambridge Crystallographic Data Centre and Fachinformationszentrum Karlsruhe Access Structures service *via* http://www.ccdc.cam.ac.uk/data_request/cif.

Table S5: Crystallographic data.

**Identification code**	**R2**	**Y2**	**G0**	**4c**	**4d**
CCDC number	2429052	2427615	2429053	2427616	2427617
Name in cif	alh_206_5dm	dan10575	alh_fr18m	dan10717	dan10606
Empirical formula	$C_{36}H_{26}N_4O_4$	$C_{37}H_{28}N_4O_4$	$C_{36}H_{26}N_4O_4$	$C_{22}H_{13}F_2N_3O_2$	$C_{23}H_{15}F_2N_3O_2$
M [g/mol]	578.61	592.63	578.61	389.35	403.38
Crystal habit	Red plate	Yellow-orange plate	Yellow plate	Yellow needle	Yellow plate
Crystal size [mm]	0.306×0.300×0.048	0.063×0.154×0.159	0.302×0.188×0.074	0.042x0.046x0.140	0.039x0.074x0.166
T [K]	100(2)	102(2)	100(2)	100(2)	100(2)
Crystal system	triclinic	triclinic	triclinic	monoclinic	triclinic
Space group	$P\bar{1}$	$P\bar{1}$	$P\bar{1}$	$C2/c$	$P\bar{1}$
a [Å]	7.6840(7)	9.3355(8)	6.8945(7)	30.5559(13)	11.6575(2)
b [Å]	7.9687(8)	11.5289(12)	7.9423(8)	6.7574(3)	12.9957(3)
c [Å]	12.3504(12)	14.8376(15)	12.3462(12)	21.5694(9)	14.0878(3)
α [°]	71.581(3)	100.397(3)	74.095(3)	90	66.3220(10)
β [°]	72.351(3)	93.544(3)	83.457(3)	124.734(2)	89.8080(10)
γ [°]	85.723(3)	110.076(3)	82.201(3)	90	75.4290(10)
V [Å^3]	683.53(12)	1462.0(2)	642.07(11)	3660.0(3)	1880.21(7)
Z	1	2	1	8	4
D_{calc} [g·cm^{-3}]	1.406	1.346	1.341	1.413	1.425
μ(Ka [mm^{-1}])	0.755(Cu)	0.089(Mo)	0.085(Mo)	0.898(Cu)	0.894(Cu)
Transmissions	0.75/0.59	0.9940/0.9860	0.75/0.70	0.8850/0.9630	0.8660/0.9660
F(000)	302	620	270	1600	832
Index ranges	$-9 \leq h \leq 9$	$-1 \leq h \leq 11$	$-10 \leq h \leq 10$	$-35 \leq h \leq 36$	$-13 \leq h \leq 13$
	$-10 \leq k \leq 10$	$-13 \leq k \leq 13$	$-12 \leq k \leq 12$	$-8 \leq k \leq 8$	$-15 \leq k \leq 15$
	$-15 \leq l \leq 15$	$-17 \leq l \leq 17$	$-19 \leq l \leq 19$	$-25 \leq l \leq 25$	$-16 \leq l \leq 16$
θ_{max} [°]	80.549	25.03	33.265	66.75	66.73
Reflections collected	32614	33236	52443	27987	30628
Independent reflections	2975	5166	4924	3217	6609
R_{int}	0.0408	0.1211	0.0374	0.0818	0.0392
Refined parameters	200	410	183	263	545
R_1 [$I > 2\sigma(I)$]	0.0345	0.0630	0.0478	0.0480	0.0337
wR_2 [all data]	0.0964	0.1877	0.1438	0.1368	0.0883
GooF	1.067	1.014	1.041	1.020	1.031
$\Delta\rho_{final}$ (max/min) [e·Å^{-3}]	0.254/-0.247	0.305/-0.335	0.910/-0.248	0.216/-0.377	0.209/-0.196

Crystal structure of R2

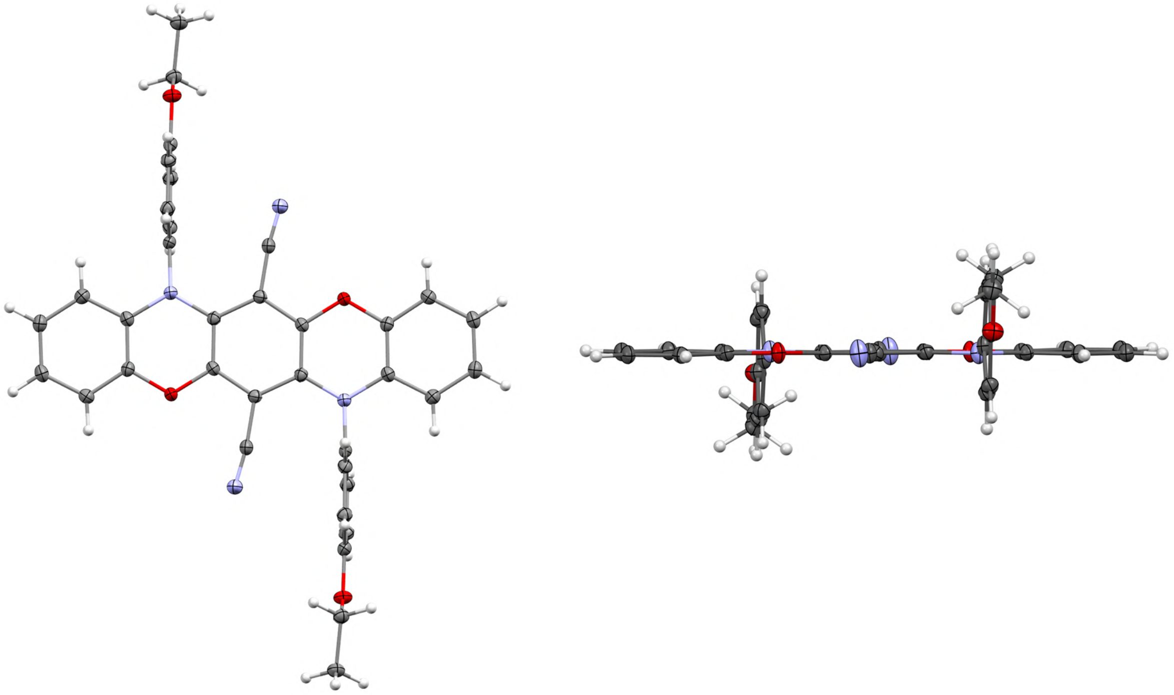

Figure S81: Molecular structure in the crystal of compound **R2.** Displacement ellipsoids are set at 50% probability. View perpendicular (left) and in plane (right) of the luminophore unit.

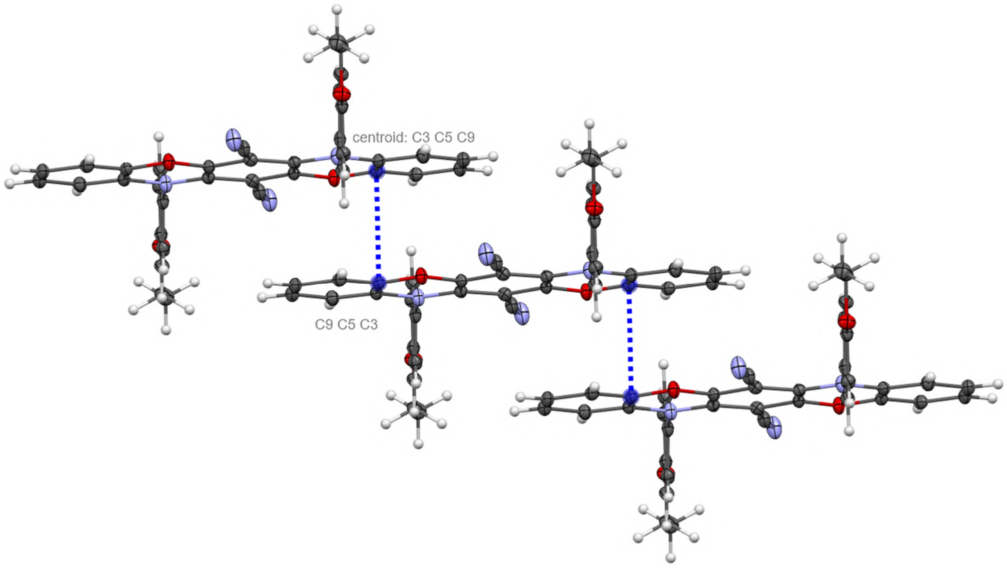

Figure S82: Stairs-like chain formation *via* $\pi\cdots\pi$ interactions (blue color) between the luminophore units of compound **R2**.

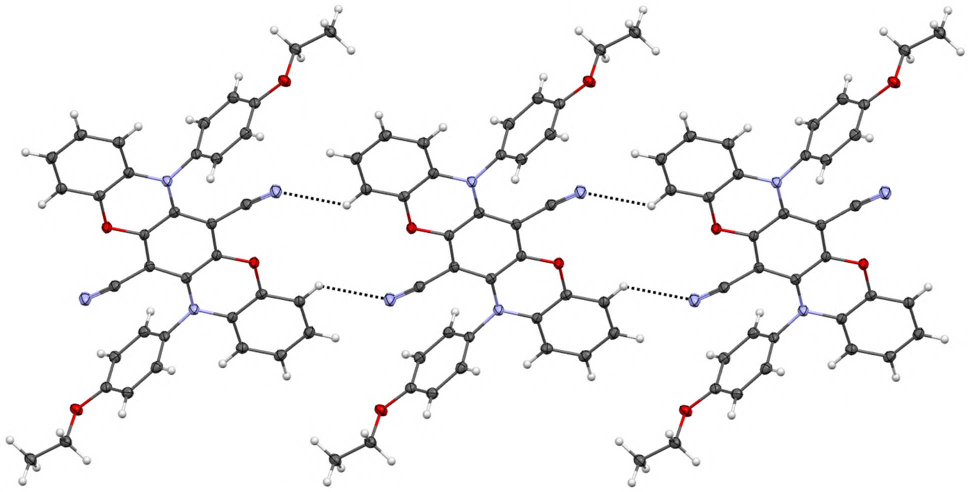

Figure S83: Linear chain formation *via* C-H···N hydrogen bond interactions (black color) involving the luminophore unit and the nitrile groups.

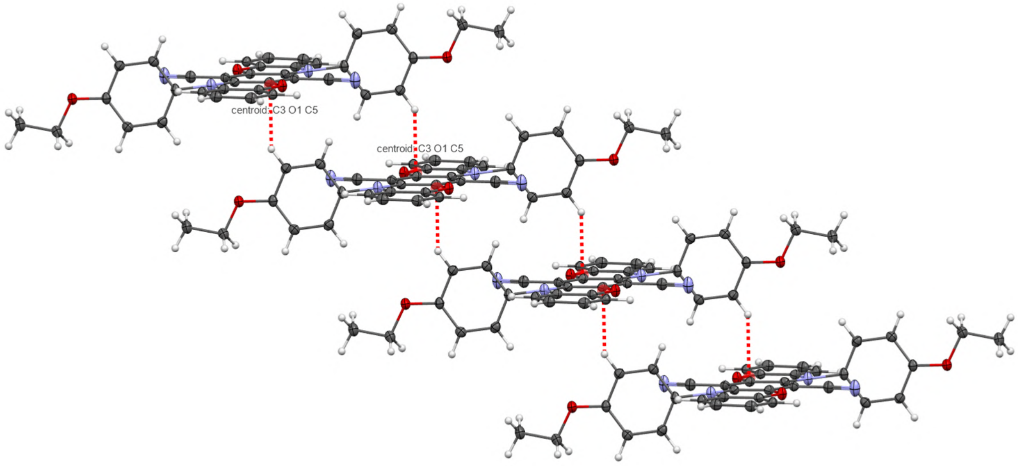

Figure S84: Additional C-H···π interactions (red color) between the *N*-aryl substituent and the adjacent oxazine ring.

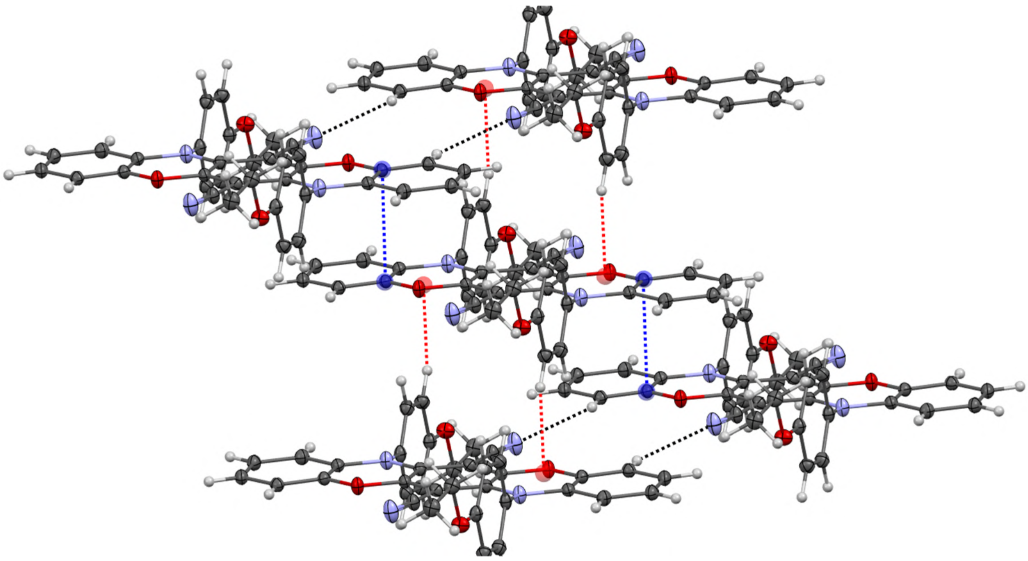

Figure S85: Excerpt of the packing diagram representing all three types of interactions between the **R2** molecules.

Table S6: Non-covalent intermolecular CH···π, π···π and CH···N interactions in compound **R2** (Å and deg).

D-H···*A*	*d*(*D*-H)	*d*(H···*A*)	(*D*···*A*)	∠(*DHA*)
*C*g1···*C*g1[#1, a]			3.287	
C13-H13···*C*g2[#2, b]	0.95	2.488	3.296	142.9
C10-H10···N1[#3]	0.95	2.540	3.222	128.8

Symmetry transformations used to generate equivalent atoms: [#1] x, 1+y, z; [#2] -1+x, y, z; [#3] 1+x, -1+y, z.
[a] *C*g1 is the centroid involving C3/C5/C9 atoms. [b] *C*g2 is the centroid involving C3/O1/C5 atoms.

CRYSTAL STRUCTURE OF Y2

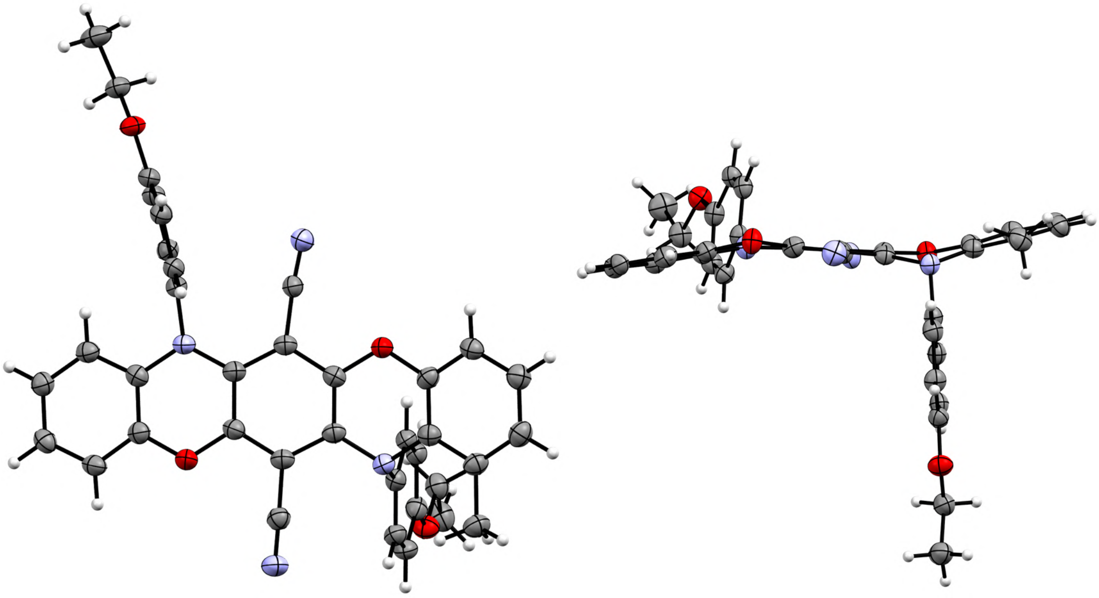

Figure S86: Molecular structure in the crystal of compound **Y2.** Displacement ellipsoids are set at 50% probability. View perpendicular (left) and in plane (right) of the luminophore unit.

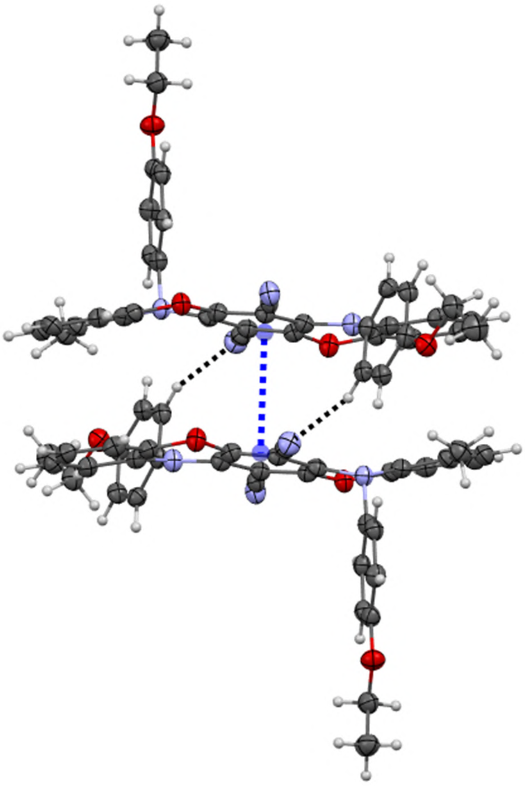

Figure S87: Dimer type formation *via* $\pi\cdots\pi$ interactions (blue color) between the parts with sp^2-hybridized nitrogen atoms, supported by C-H···N hydrogen bond interactions (black color).

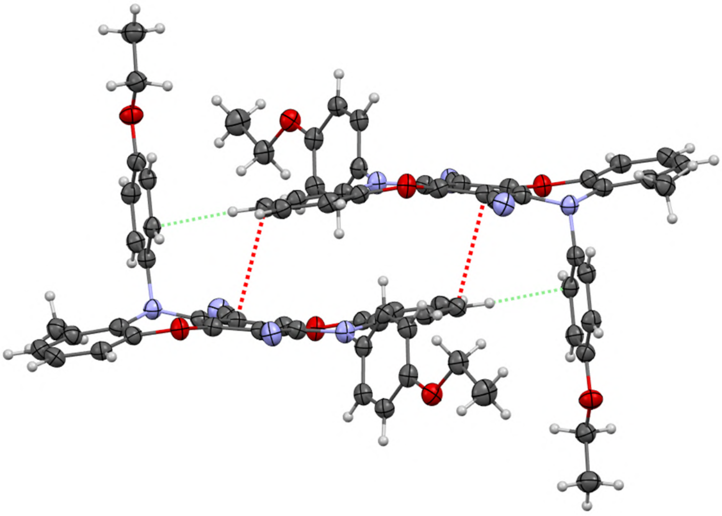

Figure S88: Dimer type formation via $\pi\cdots\pi$ interactions (red color) between the parts with sp^3-hybridized nitrogen atoms, supported by CH···π interactions (light green color).

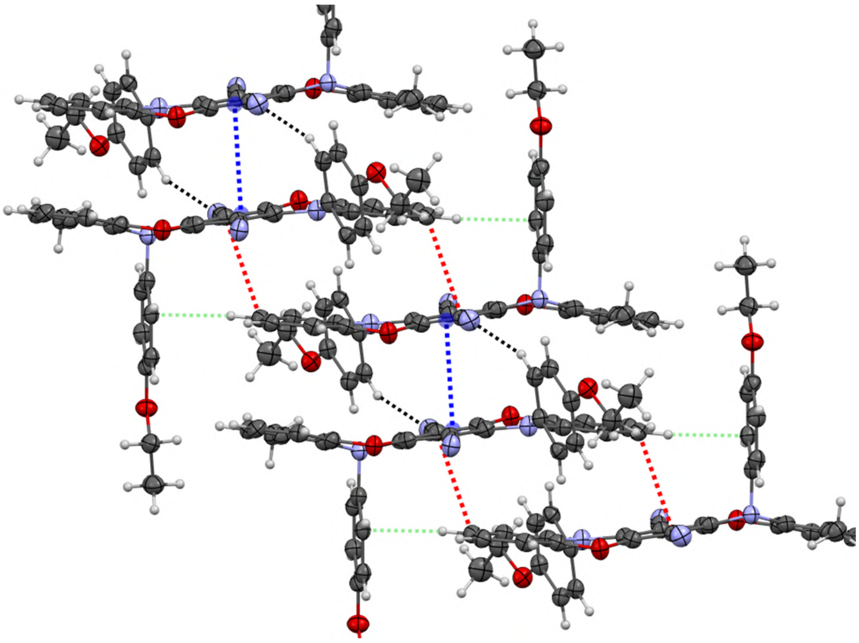

Figure S89: Excerpt of the packing diagram representing all interactions between the **Y2** molecules.

Table S7: Non-covalent intermolecular CH···π, π···π and CH···N interactions in compound **Y2** (Å and deg).

D-H···*A*	*d*(*D*-H)	*d*(H···*A*)	(*D*···*A*)	∠(*D*H*A*)
*C*g1···*C*g2[#1, a, b]			3.335	
*C*g3···*C*g3[#1, c]			3.383	
C14-H14···*C*g4[#1, d]	0.95	2.594	3.472	153.8
C46-H46···N1[#2]	0.95	2.558	3.466	157.8

Symmetry transformations used to generate equivalent atoms: [#1] 1-x, 2+y, 1-z; [#2] 2-x, 2-y, 1-z. [a] *C*g1 is the centroid involving C1/C6/C5 atoms. [b] *C*g2 is the centroid involving C14/C15 atoms. [c] *C*g3 is the centroid involving C5/C4/C1/C7/N1 atoms. [d] *C*g4 is the centroid involving C33/C32/C31/C36 atoms.

Crystal structure of G0

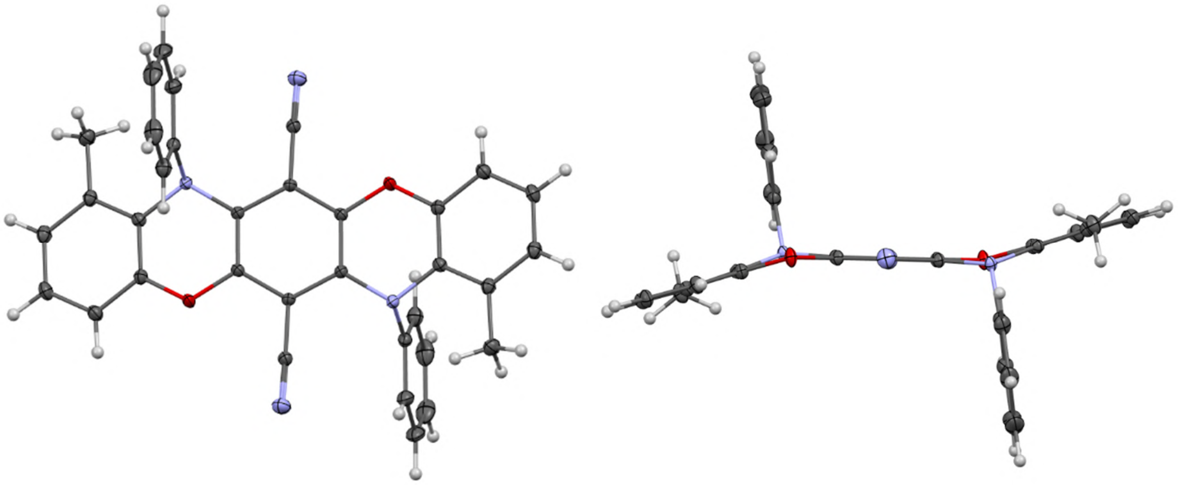

Figure S90: Molecular structure in the crystal of compound **G0**. Displacement ellipsoids are set at 50% probability. View perpendicular (left) and in plane (right) of the luminophore unit.

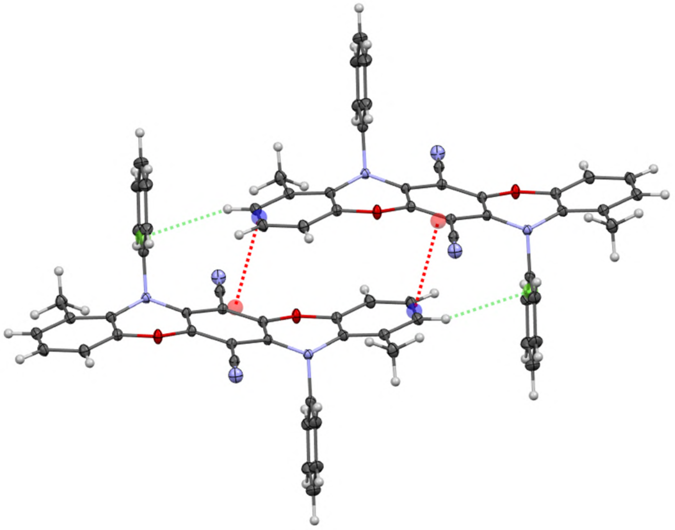

Figure S91: Dimer type formation *via* $\pi\cdots\pi$ interactions (red color) between the luminophore units with sp^3-hybridized nitrogen atoms, supported by C-H$\cdots\pi$ interactions (light green color).

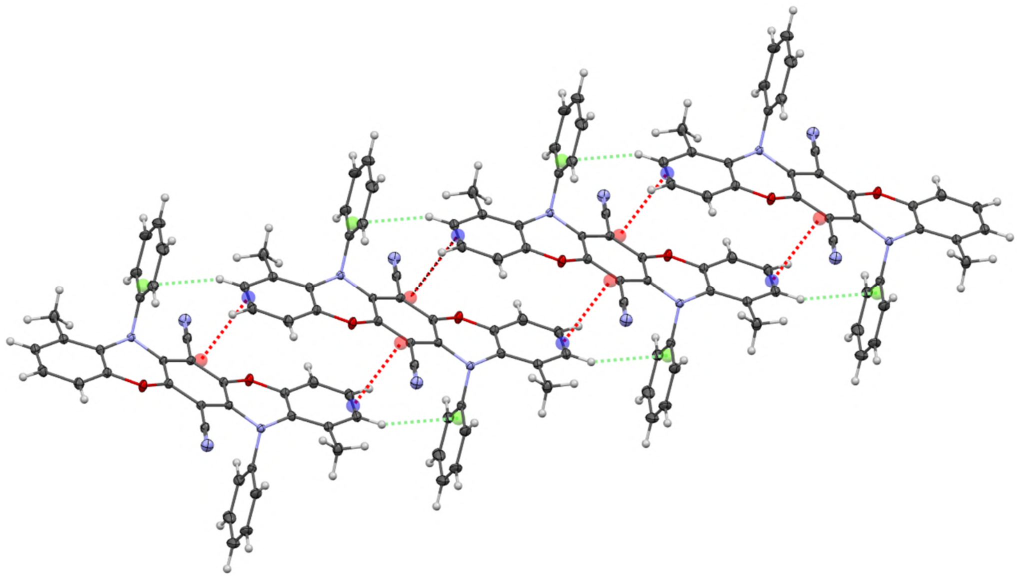

Figure S92: Stairs-like chain formation *via* $\pi\cdots\pi$ interactions (red color) supported by C-H$\cdots\pi$ interactions (light green color).

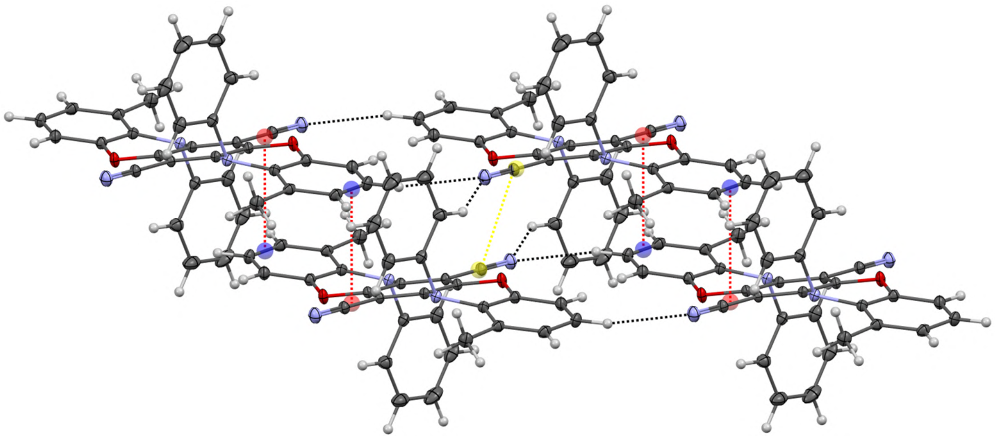

Figure S93: Excerpt of the packing diagram representing all interactions between the **G0** molecules ($\pi\cdots\pi$: red color; C-H$\cdots$N: black color; $\pi\cdots\pi$ between the CN groups: yellow color).

Table S8: Non-covalent intermolecular CH$\cdots\pi$, $\pi\cdots\pi$ and CH$\cdots$N interactions in compound **G0** (Å and deg).

D-H$\cdots$*A*	*d*(*D*-H)	*d*(H$\cdots$*A*)	(*D*$\cdots$*A*)	∠(*DHA*)
*C*g1$\cdots$*Cg*2[#1, a, b]			3.285	
*C*g3$\cdots$*C*g3[#2, c]			3.399	
C8-H8$\cdots$*Cg*4[#3, d]	0.95	2.798	3.645	153.8
C9-H9$\cdots$N1[#3]	0.95	2.588	3.488	157.7
C17-H17$\cdots$N1[#3]	0.95	2.642	3.472	146.2

Symmetry transformations used to generate equivalent atoms: [#1] 2-x, 3+y, 1-z; [#2] 1-x, 2+y, 1-z; [#3] x, 1+y, z. [a] *C*g1 is the centroid involving C3/C1/C4 atoms. [b] *C*g2 is the centroid involving C8/C9 atoms. [c] *C*g3 is the centroid involving N1/C4/C1 atoms. [d] *C*g4 is the centroid involving C13/C12/C17 atoms.

CRYSTAL STRUCTURE OF 4C

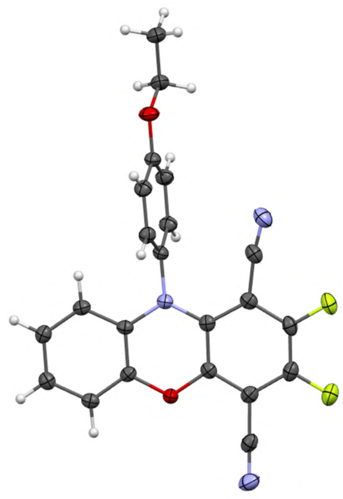

Figure S94: Molecular structure in the crystal of compound **4c**. Displacement ellipsoids are set at 50% probability.

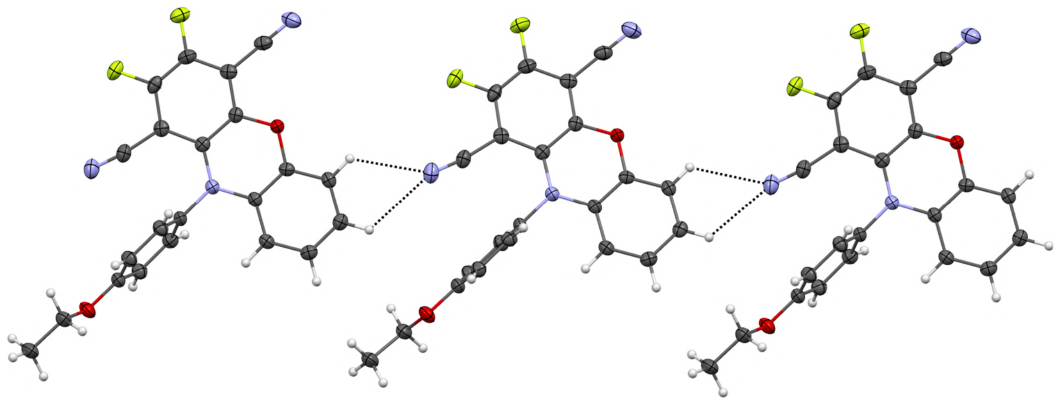

Figure S95: Linear chain formation via alternating $\pi\cdots\pi$ interactions (red and blue color).

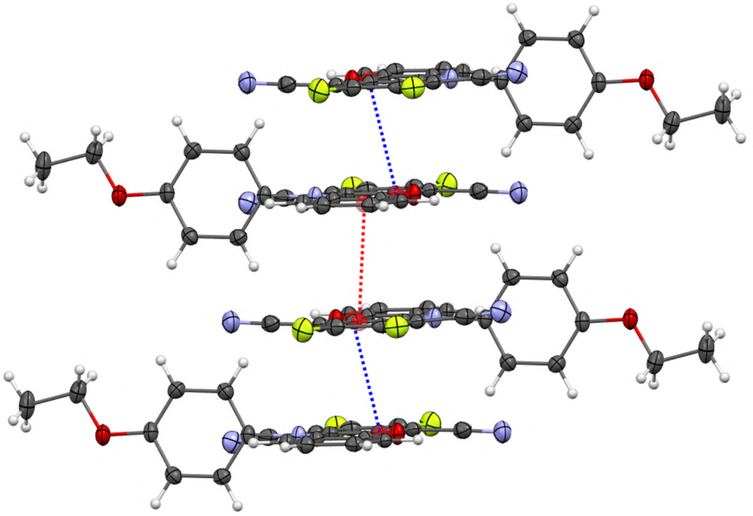

Figure S96: Linear chain formation via C-H···N interactions (black color) between the CN groups and the phenoxazine unit.

Table S9: Non-covalent intermolecular CH···π, π···π and CH···N interactions in compound **4c** (Å and deg).

D-H···*A*	*d*(*D*-H)	*d*(H···*A*)	(*D*···*A*)	∠(*DHA*)
*C*g1···*C*g1[#1, a]			3.410	
*C*g2···*C*g3[#1, b, c]			3.494	
C14-H14···N2[#2]	0.95	2.672	3.488	124.0
C15-H15···N2[#2]	0.95	2.712	3.315	121.9

Symmetry transformations used to generate equivalent atoms: [#1] 0.5-x, 1.5-y, 1-z; [#2] x, 1-y, -0.5+z. [a] *C*g1 is the centroid involving C3/C4/C5/C6/C13/C14/C15/C16/C11 atoms. [b] *C*g2 is the centroid involving C4/C5/C6 atoms. [c] *C*g3 is the centroid involving C14/C15/C16 atoms.

CRYSTAL STRUCTURE OF 4D

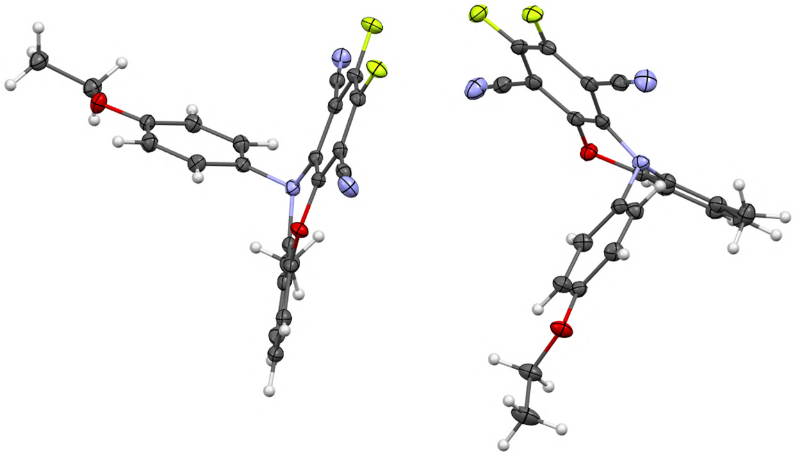

Figure S97: Molecular structure in the crystal of compound **4d**. Two independent molecules named with suffix A (right side) and B (left side) were found in the asymmetric unit. Displacement ellipsoids are set at 50% probability.

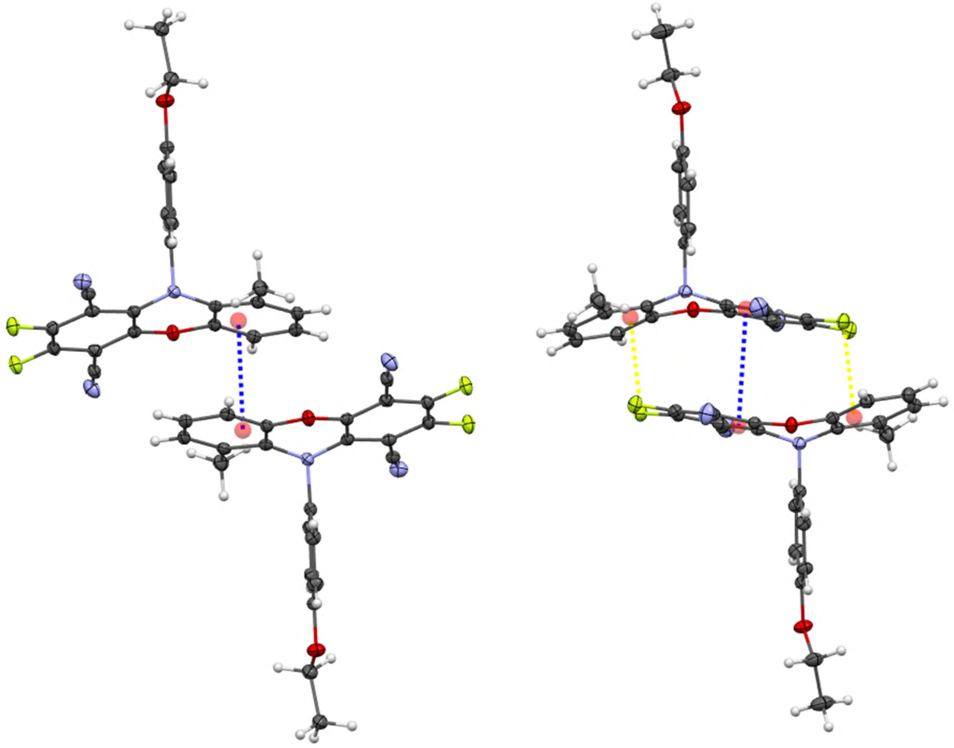

Figure S98: Dimer type formation via $\pi\cdots\pi$ interactions (left: blue color for molecule with suffix B) and $\pi\cdots\pi$ interactions combined with F$\cdots\pi$ interactions (right: blue and yellow color for molecule with suffix A).

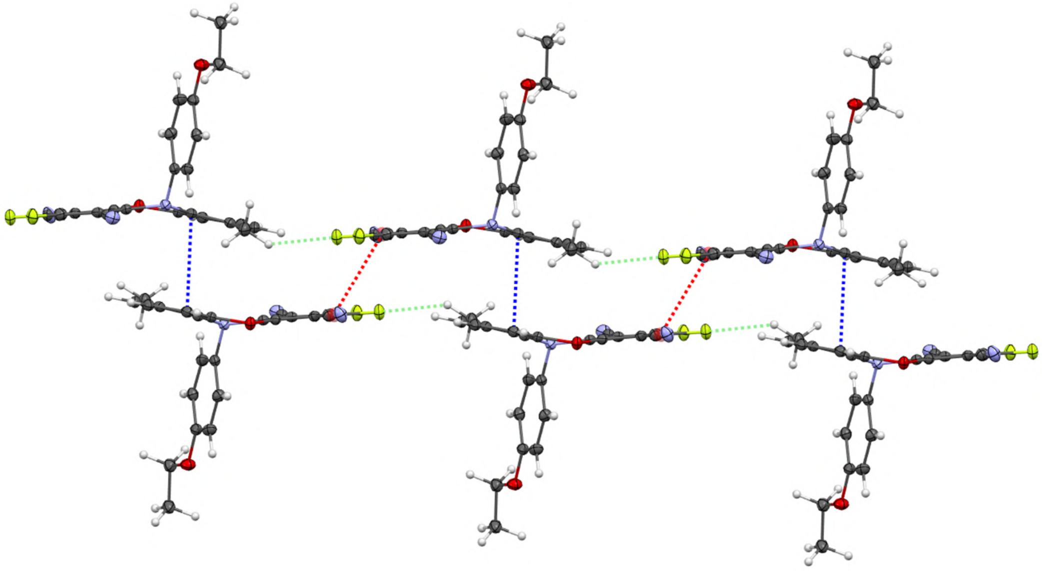

Figure S99: Linear chain formation *via* $\pi\cdots\pi$ interactions (red color) between the CN groups supported by C-H$\cdots$F interactions (light green color).

Table S10: Non-covalent intermolecular CH···π, π···π and CH···N interactions in compound **4d** (Å and deg).

D-H···*A*	*d*(*D*-H)	*d*(H···*A*)	(*D*···*A*)	∠(*DHA*)
*C*g1···*C*g1[#1, a]			3.503	
F···*C*g2[#1, b]			2.967	
*C*g3···*C*g3[#2, c]			3.309	
*C*g4···*C*g4[#3, d]			3.296	
C17B-H17E···F2B[#3]	0.98	2.529	3.311	136.6

Symmetry transformations used to generate equivalent atoms: [#1] 1-x, 2-y, 1-z; [#2] -x, 1-y, 1-z; [#3] -1+x, y, z. [a] *C*g1 is the centroid involving C8/C1/C6/C5 atoms from molecule with suffix A. [b] *C*g2 is the centroid involving C11/C12/C13/C14 atoms from molecule with suffix A. [c] *C*g3 is the centroid involving C12/C11/C16/C15/C14/C13/C5 atoms from molecule with suffix B. [d] *C*g4 is the centroid involving C7/N1 atoms from molecule with suffix B.

7 CrystalExplorer

Further analysis of occurring interactions in the crystal packing were carried out using Hirshfeld surface (HS) analysis and the corresponding Fingerprint plots (FPP). HS calculations using the parameter d_{norm} were performed with *CrystalExplorer17* program.[9] Reciprocal contacts are included in the FP plots.

Table S11: Display of all interactions in the crystal lattices determined by HS analysis. Reciprocal contacts are summarized.

	**R2**	**Y2**	**G0**
O-O	0.2%	0.0%	0.0%
O-N	0.0%	1.0%	1.8%
O-C	3.2%	3.2%	3.3%
O-H	8.3%	6.6%	0.9%
N-N	0.0%	0.0%	0.0%
N-C	0.9%	2.2%	2.1%
N-H	12.9%	11.9%	12.4%
C-C	4.4%	4.4%	7.6%
C-H	24.4%	24.0%	24.7%
H-H	45.7%	46.6%	47.1%
Sum	100.0%	99.9%	99.9%

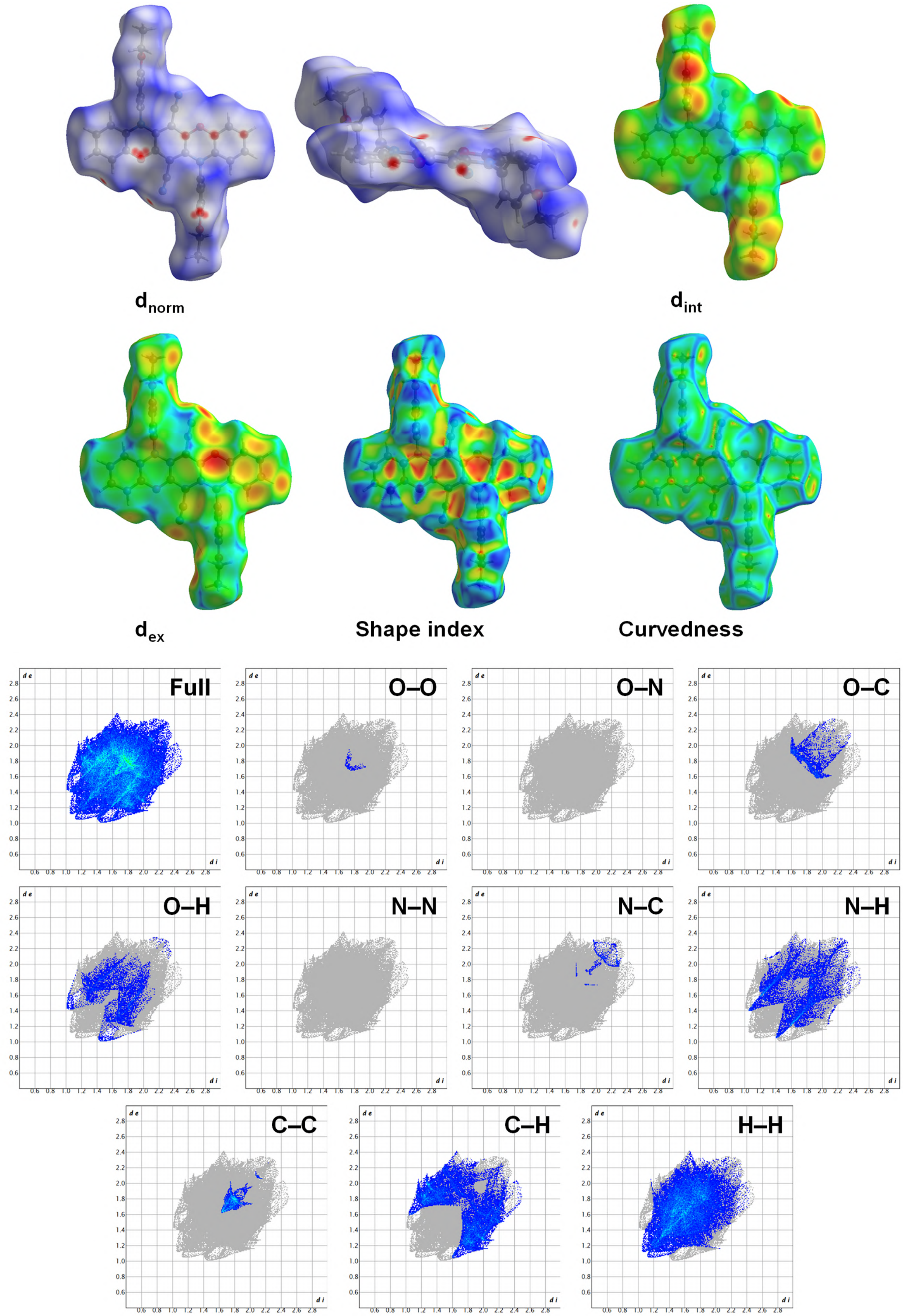

Figure S100: Hirshfeld surface of **R2** mapped with d_{norm} (normalised contact distance), d_{int}, d_{ex}, shape index and curvedness; Fingerprint plot for **R2** resolved into the contacts of all elements contained. Reciprocal contacts are included.

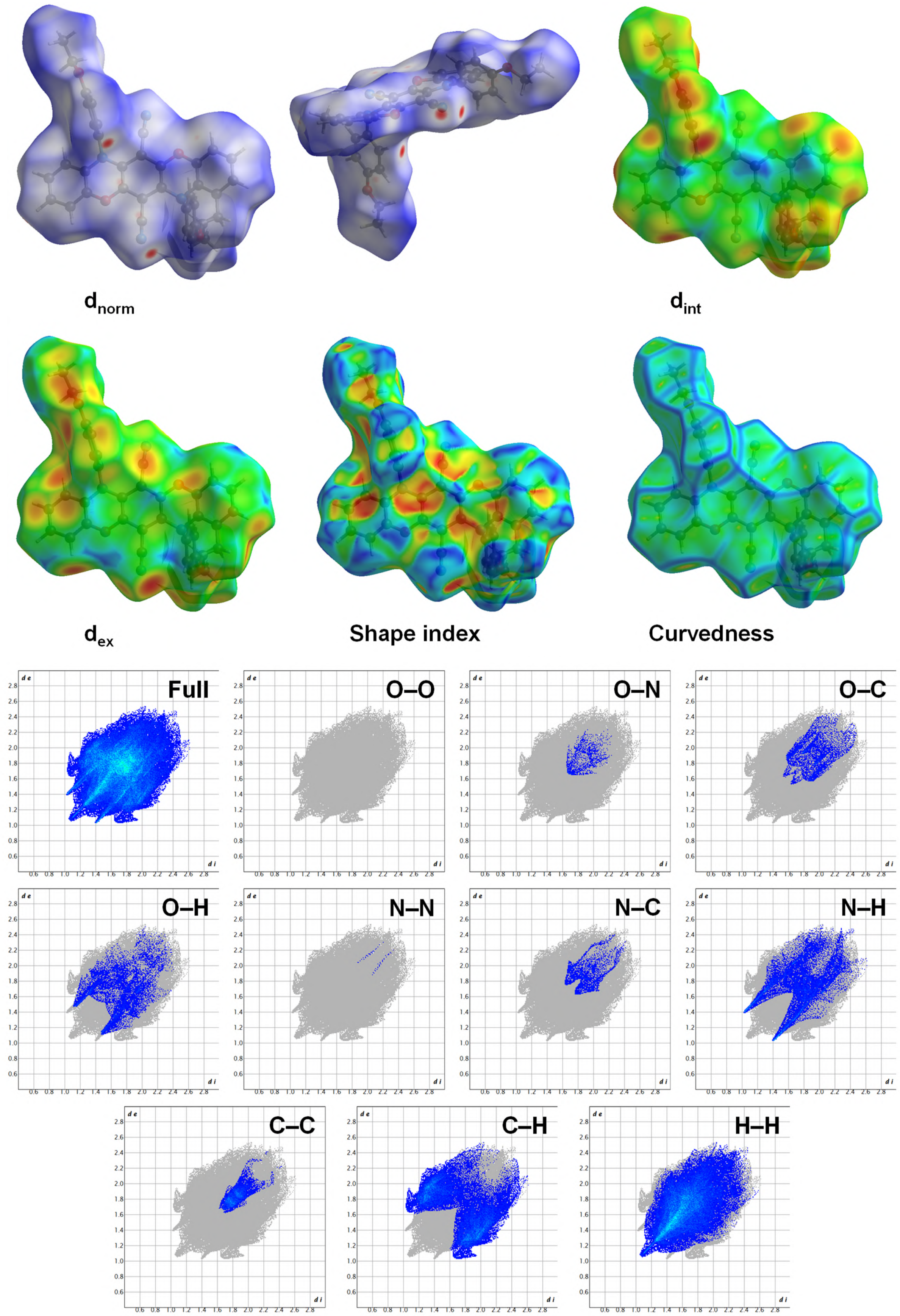

Figure S101: Hirshfeld surface of **Y2** mapped with d_{norm} (normalised contact distance), d_{int}, d_{ex}, shape index and curvedness; Fingerprint plot for **Y2** resolved into the contacts of all elements contained. Reciprocal contacts are included.

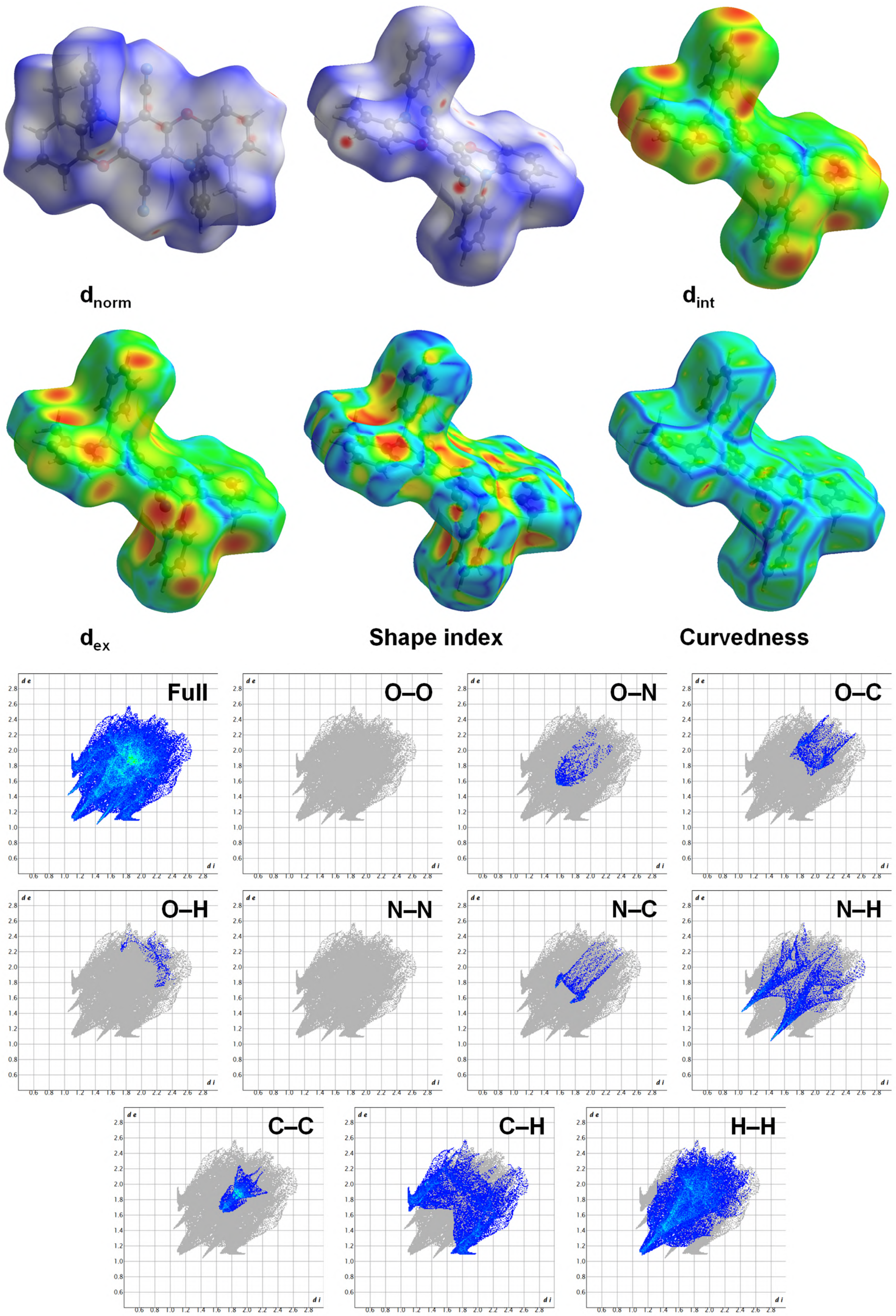

Figure S102: Hirshfeld surface of **G0** mapped with d_{norm} (normalised contact distance), d_{int}, d_{ex}, shape index and curvedness; Fingerprint plot for **G0** resolved into the contacts of all elements contained. Reciprocal contacts are included.

8 QUANTUM CHEMICAL CALCULATIONS

COMPUTATIONAL DETAILS

Theoretical calculations were performed using *Gaussian 16*.[10] The compound geometries in the energetic ground states were calculated using PBE0/def2-TZVP with the Grimme dispersion correction (D3BJ).[11–13] It was ensured that the four convergence criteria of *Gaussian* are fulfilled. Frequency analysis revealed no imaginary frequencies, indicating that the obtained structures are energetic minima. To simulate UV/Vis-spectra, the first 40 transitions were calculated using the *TD* keyword.[14] The optimized first excited state geometries, natural transition orbital (NTO) pairs, and electron-density differences were obtained using TD-PBE0/def2-TZVP. NICS(1) values were obtained using GIAO-PBE0/def2-TZVP. The centroids of the oxazine rings were placed 1 Å above the ring planes in z-direction. Using the same level of theory, the NICS(1) value of benzene was calculated as -10.0 ppm, denoting aromaticity.[15]

Table S12: Overview of the calculated absorption and emission wavelengths using TD-PBE0/def2-TZVP and energies (eV) of the respective HOMOs and LUMOs.

		R2	Y2	G2
λ_{ab} [nm]	Calculated	500	455	426
λ_{em} [nm]	Calculated	592	569	521
Energy (eV)	LUMO (S_0)	-1.833	-1.994	-2.192
	HOMO (S_0)	-4.982	-5.428	-5.896
	HOMO-LUMO (S_0) gap	-3.149	-3.434	-3.704
NICS(1) [ppm]	Oxazine rings	6.4	6.7 / -0.12	0.25

CARTESIAN COORDINATES OF THE OPTIMIZED GEOMETRIES

Cartesian coordinates of the optimized geometry of **R2** (S_0) using PBE0-GD3BJ/def2-TZVP.

O	1.08546800	-2.46046500	-0.45162800
O	-5.73438800	-0.40819100	-4.75364300
N	-3.03795400	1.67234200	-1.89043300
N	-1.12808300	-1.69499700	-1.96046800
C	-1.09038400	0.45493600	-0.72928300
C	-0.59979600	-0.83715700	-1.00411600
C	0.49786300	-1.25335500	-0.25820600
C	-2.18302700	1.06735600	-1.40920800
C	0.48070000	-3.35958400	-1.28990800
C	-0.62149900	-2.99592900	-2.05972900
C	-1.17207700	-3.94952600	-2.90676200
H	-2.02792700	-3.68513900	-3.51330800
C	-0.63009200	-5.22666700	-2.97845000
H	-1.07556500	-5.95147100	-3.64891400
C	0.46283300	-5.57034000	-2.20456400
H	0.88722500	-6.56503200	-2.25713700
C	1.01928000	-4.62521700	-1.35139900
H	1.87604400	-4.84958300	-0.72780500
C	-2.29646500	-1.36646600	-2.70081400
C	-3.55494900	-1.65373000	-2.18228900
H	-3.63571200	-2.11970600	-1.20697600
C	-4.68648400	-1.32671800	-2.89425800

H	-5.67584900	-1.52007200	-2.49905600
C	-4.57510900	-0.71127300	-4.14222600
C	-3.31524400	-0.45619500	-4.67806300
H	-3.20597000	0.01468100	-5.64474400
C	-2.18355200	-0.79229300	-3.95326800
H	-1.19840700	-0.58603800	-4.35497100
C	-5.68398100	0.28561300	-5.98736700
H	-5.15724300	-0.32373800	-6.73244800
H	-5.12663600	1.22102800	-5.85787700
C	-7.10000300	0.55680800	-6.42050800
H	-7.10243800	1.09129100	-7.37247700
H	-7.61834300	1.16724900	-5.67937700
H	-7.65102300	-0.37674700	-6.54649400
O	-1.08546800	2.46046500	0.45162800
O	5.73438800	0.40819100	4.75364300
N	3.03795400	-1.67234200	1.89043300
N	1.12808300	1.69499700	1.96046800
C	1.09038400	-0.45493600	0.72928300
C	0.59979600	0.83715700	1.00411600
C	-0.49786300	1.25335500	0.25820600
C	2.18302700	-1.06735600	1.40920800
C	-0.48070000	3.35958400	1.28990800
C	0.62149900	2.99592900	2.05972900
C	1.17207700	3.94952600	2.90676200
H	2.02792700	3.68513900	3.51330800
C	0.63009200	5.22666700	2.97845000
H	1.07556500	5.95147100	3.64891400
C	-0.46283300	5.57034000	2.20456400
H	-0.88722500	6.56503200	2.25713700
C	-1.01928000	4.62521700	1.35139900
H	-1.87604400	4.84958300	0.72780500
C	2.29646500	1.36646600	2.70081400
C	3.55494900	1.65373000	2.18228900
H	3.63571200	2.11970600	1.20697600
C	4.68648400	1.32671800	2.89425800
H	5.67584900	1.52007200	2.49905600
C	4.57510900	0.71127300	4.14222600
C	3.31524400	0.45619500	4.67806300
H	3.20597000	-0.01468100	5.64474400
C	2.18355200	0.79229300	3.95326800
H	1.19840700	0.58603800	4.35497100
C	5.68398100	-0.28561300	5.98736700
H	5.15724300	0.32373800	6.73244800
H	5.12663600	-1.22102800	5.85787700
C	7.10000300	-0.55680800	6.42050800
H	7.10243800	-1.09129100	7.37247700
H	7.61834300	-1.16724900	5.67937700
H	7.65102300	0.37674700	6.54649400

Cartesian coordinates of the optimized geometry of **R2** (S_1) using TD-PBE0/def2-TZVP.

O	-0.01625400	2.75406100	-0.38066000
O	-7.25916100	-1.14536900	0.64696700
N	-3.19914700	-2.27976600	-1.11504100
N	-2.46093700	1.43528600	-0.38152700
C	-1.24848000	-0.70752000	-0.57442800
C	-1.26935800	0.71319300	-0.43825800
C	-0.06509500	1.39488700	-0.41236500
C	-2.34882500	-1.52689900	-0.86905400
C	-1.16661500	3.46009700	-0.45088800
C	-2.41521700	2.82396800	-0.42232100

C	-3.55691200	3.62760200	-0.39983600
H	-4.53077700	3.16310700	-0.33510800
C	-3.44817700	5.00538900	-0.45129700
H	-4.34922300	5.60653300	-0.43690000
C	-2.20200400	5.61632100	-0.51435600
H	-2.12011200	6.69533800	-0.55744300
C	-1.05836000	4.83774500	-0.50632200
H	-0.06598400	5.27109300	-0.52944300
C	-3.69677400	0.78900400	-0.09207800
C	-4.71311500	0.74946300	-1.04214600
H	-4.55465000	1.19801800	-2.01584500
C	-5.89510900	0.10448000	-0.76072100
H	-6.68615900	0.03979900	-1.49741100
C	-6.07922600	-0.52204000	0.47455800
C	-5.06865700	-0.47051600	1.43030100
H	-5.18713900	-0.94652400	2.39377700
C	-3.88524200	0.19139200	1.14294300
H	-3.09298700	0.22868300	1.88165800
C	-7.47470700	-1.86887400	1.84592000
H	-7.42338400	-1.18638300	2.70370500
H	-6.68769700	-2.62330800	1.96416700
C	-8.83200100	-2.51573500	1.75706300
H	-9.03407500	-3.08296200	2.66791700
H	-8.87839100	-3.19859100	0.90718500
H	-9.61308900	-1.76262900	1.63886900
O	-0.02322900	-2.68361900	-0.55288900
O	7.03443900	1.14368300	1.43733600
N	3.19192700	2.37682300	-0.75923200
N	2.41243600	-1.37105400	-0.31368400
C	1.21109800	0.78063800	-0.44654600
C	1.22635900	-0.64492900	-0.40143200
C	0.02356200	-1.32506800	-0.49713000
C	2.32690700	1.61438900	-0.61616600
C	1.13178400	-3.38561200	-0.59837200
C	2.37377700	-2.75432500	-0.45010300
C	3.51505300	-3.55725800	-0.41540200
H	4.48122400	-3.09688800	-0.26313700
C	3.41474300	-4.92878400	-0.56587500
H	4.31549900	-5.52996300	-0.53882300
C	2.17697300	-5.53320500	-0.74424600
H	2.10143700	-6.60679600	-0.86491600
C	1.03192100	-4.75601000	-0.75344800
H	0.04448500	-5.18638400	-0.86717900
C	3.62275300	-0.74946700	0.11177100
C	3.73310300	-0.28314900	1.41835400
H	2.89995000	-0.40128400	2.10147600
C	4.88800700	0.34333800	1.83212700
H	4.98548100	0.72918900	2.83939900
C	5.95475000	0.50650400	0.94797500
C	5.85310200	0.01641500	-0.35292600
H	6.66056900	0.14217600	-1.06059900
C	4.68615700	-0.60585900	-0.76232500
H	4.58849800	-0.95178500	-1.78475800
C	8.11615600	1.41499100	0.56312300
H	8.53050900	0.47224200	0.18396500
H	7.75696600	1.99761700	-0.29354900
C	9.15477900	2.18209900	1.33859900
H	10.00753700	2.40812400	0.69522200
H	8.74264800	3.12220300	1.70888300
H	9.50987600	1.60031900	2.19106700

Cartesian coordinates of the optimized geometry of **Y2** (S_0) using PBE0-GD3BJ/def2-TZVP.

N	2.58607900	3.04514600	-2.13389800
N	-2.43436700	-2.61552200	0.11824500
N	-2.63320400	1.23063800	-0.36221800
N	2.68109900	-0.46314000	-1.22208600
O	-0.63369900	2.99077400	-1.18761900
O	0.66999900	-2.27706900	-0.57472400
O	6.11899300	1.15552200	2.86376800
O	-6.74038100	-2.17149700	1.12668800
C	0.93914800	1.28039000	-1.24428500
C	1.33984000	-0.05318900	-1.06137200
C	0.36931100	-0.96418200	-0.71389100
C	-0.97012200	-0.58911900	-0.48544200
C	-1.35490300	0.75974100	-0.59722400
C	-0.36867300	1.67559000	-0.99664300
C	1.85773900	2.25545200	-1.71967400
C	-1.84129100	-1.66201200	-0.14286400
C	-1.89370300	3.45750800	-0.92436200
C	-2.90696900	2.59729500	-0.51055600
C	-4.16245300	3.13529800	-0.25879600
H	-4.96137400	2.48182300	0.06496800
C	-4.39220100	4.49530300	-0.42197900
H	-5.38015400	4.89115600	-0.22116600
C	-3.37397600	5.33435300	-0.83583500
H	-3.54939800	6.39491900	-0.96485800
C	-2.11377400	4.80709700	-1.08728500
H	-1.28755600	5.42762600	-1.41203100
C	2.80108700	-1.83282000	-1.58715700
C	1.79092300	-2.72088400	-1.23842100
C	1.86573100	-4.06856300	-1.52899000
H	1.05085700	-4.71799300	-1.23461900
C	2.98975600	-4.53933100	-2.18655700
H	3.07247500	-5.59359600	-2.42163900
C	3.99654500	-3.66330400	-2.56011800
H	4.86517800	-4.03624700	-3.09121200
C	3.91909300	-2.30245500	-2.28005600
C	5.00345800	-1.36580700	-2.70195600
H	5.63816900	-1.08311800	-1.85748300
H	4.58217000	-0.44204900	-3.10400500
H	5.63447700	-1.82864700	-3.46110500
C	3.56385000	-0.05460300	-0.15093900
C	3.50755700	-0.69477900	1.07737200
H	2.80057600	-1.50345000	1.22845700
C	4.34725600	-0.32184100	2.11556500
H	4.28242700	-0.84040300	3.06219800
C	5.25906800	0.71353900	1.92245500
C	5.31694300	1.35657000	0.68763000
H	6.03090300	2.15981300	0.55326700
C	4.47944600	0.97257700	-0.34031800
H	4.52516500	1.47466100	-1.29705800
C	6.09848100	0.53702000	4.13551100
H	6.31149500	-0.53420600	4.02964000
H	5.10072400	0.63925200	4.58057800
C	7.13912700	1.20892100	4.99156500
H	8.13047300	1.10200400	4.54805400
H	7.15380300	0.75715400	5.98539800
H	6.92190000	2.27304000	5.09799900
C	-3.68890900	0.36951400	0.04681100
C	-3.89149200	0.11002700	1.38987000
H	-3.23335300	0.56015300	2.12405300
C	-4.90804400	-0.73871500	1.79574600

H	-5.03543900	-0.94909200	2.84816300
C	-5.73805500	-1.32209300	0.84134800
C	-5.55535000	-1.02645800	-0.51075400
H	-6.21460500	-1.48811300	-1.23499200
C	-4.53937800	-0.18502400	-0.90298500
H	-4.38225400	0.03553500	-1.95233100
C	-6.92591600	-2.56781300	2.47412300
H	-7.15744500	-1.68886000	3.08865900
H	-5.99919400	-3.01409900	2.85407600
C	-8.05712200	-3.56033800	2.50970500
H	-8.97641100	-3.11225400	2.12879200
H	-8.23184000	-3.88908700	3.53599500
H	-7.82047800	-4.43480800	1.90156700

Cartesian coordinates of the optimized geometry of **Y2** (S_1) using TD-PBE0/def2-TZVP.

N	2.53026300	3.45879100	-1.45266000
N	-2.76560400	-2.41560300	-1.42786600
N	-2.76228900	1.26486000	-0.40343900
N	2.59439300	-0.26128800	-1.21324400
O	-0.71859900	3.14255600	-0.60639900
O	0.54251600	-2.10638500	-1.32543000
O	5.91190900	0.76447900	3.16008000
O	-6.35366900	-2.49139200	1.51783600
C	0.91628700	1.52441900	-0.98945300
C	1.25840300	0.15265000	-1.10178600
C	0.24895200	-0.79694300	-1.13131100
C	-1.12253400	-0.50298000	-0.96745400
C	-1.44080900	0.86426600	-0.67472700
C	-0.44892400	1.81294000	-0.73712500
C	1.80634400	2.57461700	-1.24514900
C	-2.05331600	-1.52381600	-1.21168000
C	-2.00563000	3.53976600	-0.57025700
C	-3.05923700	2.61611200	-0.42751200
C	-4.35020900	3.12821700	-0.25363900
H	-5.17210300	2.44910200	-0.08027200
C	-4.57844500	4.48846500	-0.28728100
H	-5.58851200	4.85703500	-0.15581500
C	-3.52845500	5.38349800	-0.47843400
H	-3.71476800	6.44980300	-0.50835400
C	-2.24034300	4.90445600	-0.60872800
H	-1.38876500	5.56349700	-0.72449500
C	2.79672100	-1.50082300	-1.85641000
C	1.74402500	-2.41527800	-1.88850200
C	1.87328700	-3.66777100	-2.46070100
H	1.02290300	-4.33811800	-2.45169800
C	3.08610000	-4.01265700	-3.02586100
H	3.21132000	-4.98741900	-3.48189000
C	4.13344000	-3.10164700	-3.03324600
H	5.07139900	-3.36757900	-3.50810200
C	4.01348600	-1.83839000	-2.46616200
C	5.14944900	-0.86894700	-2.54048600
H	5.66391100	-0.76870200	-1.58135400
H	4.79741300	0.12678100	-2.81858300
H	5.87692700	-1.20019300	-3.28228600
C	3.45606100	0.01727500	-0.08179500
C	3.43240100	-0.85323700	0.99742800
H	2.77305700	-1.71441000	0.97537000
C	4.24195200	-0.64336600	2.10396600
H	4.20186100	-1.34168800	2.92897700
C	5.08728400	0.46313800	2.13522200

C	5.11053900	1.33758100	1.04985500
H	5.77110500	2.19527800	1.08695000
C	4.30775000	1.11457200	-0.05062000
H	4.33364800	1.80198900	-0.88448600
C	5.92410400	-0.08636400	4.29058400
H	6.20705700	-1.10228600	3.98690400
H	4.91928000	-0.13370700	4.72914200
C	6.91402400	0.47219700	5.27927000
H	7.91366900	0.51406400	4.84336600
H	6.95080500	-0.16057800	6.16840000
H	6.62835300	1.48044200	5.58396900
C	-3.69279700	0.32777200	0.11626700
C	-3.37788700	-0.37274100	1.27228900
H	-2.43976200	-0.17439100	1.77728900
C	-4.24646200	-1.32576400	1.77685500
H	-3.97506100	-1.86504400	2.67377200
C	-5.44752900	-1.58200100	1.12053900
C	-5.76715400	-0.86775000	-0.03808300
H	-6.69182200	-1.10079400	-0.55117100
C	-4.89710200	0.07162500	-0.53768800
H	-5.12100000	0.57578600	-1.47001800
C	-6.05141000	-3.30906100	2.63600200
H	-5.91684500	-2.68131100	3.52576200
H	-5.11289200	-3.84545200	2.45234000
C	-7.19420800	-4.27120900	2.82652400
H	-8.12681400	-3.73417200	3.00811700
H	-6.99595800	-4.91840600	3.68324500
H	-7.32197000	-4.89784700	1.94241300

Cartesian coordinates of the optimized geometry of **G2** (S_0) using PBE0-GD3BJ/def2-TZVP.

O	-0.77553100	-2.25528200	-1.33546300
N	-2.05549600	1.46913800	3.04715800
N	-2.44217800	-1.25300300	0.65726500
C	-0.77844300	0.51661200	1.02792600
C	-1.21456700	-0.63253300	0.35626400
C	-0.41285600	-1.14441200	-0.65393900
C	-1.50229500	1.04392200	2.13120200
C	-1.65350800	-3.10451100	-0.70122200
C	-2.48062800	-2.63294900	0.31058400
C	-3.33944100	-3.52074000	0.96274200
C	-3.36594600	-4.84498500	0.53554100
H	-4.03631600	-5.53742600	1.03216700
C	-2.55278600	-5.29276600	-0.49286800
H	-2.59042300	-6.32996600	-0.80304000
C	-1.67424000	-4.42138200	-1.11412500
H	-1.01033400	-4.73617100	-1.90939300
C	-4.20835700	-3.06164300	2.08764100
H	-5.07400100	-2.50266100	1.72194300
H	-3.65873000	-2.39497200	2.75523600
H	-4.57219500	-3.91515900	2.66016700
C	-3.61130800	-0.50261000	0.25185100
C	-4.40677100	0.13264700	1.18556200
H	-4.14770200	0.07424300	2.23367600
C	-5.52430300	0.85582900	0.78921800
H	-6.12323700	1.35015500	1.54129500
C	-5.85281600	0.93757500	-0.55971500
C	-5.05408400	0.28902000	-1.50353100
H	-5.32391400	0.36082200	-2.55003300
C	-3.94573900	-0.42141800	-1.09873200
H	-3.32975800	-0.92477900	-1.83610900

O	0.77545500	2.25519400	1.33547500
N	2.05544300	-1.46926500	-3.04712100
N	2.44212300	1.25291300	-0.65723400
C	0.77840000	-0.51673100	-1.02788400
C	1.21452500	0.63241500	-0.35622100
C	0.41280700	1.14429900	0.65397600
C	1.50221100	-1.04401400	-2.13120000
C	1.65340400	3.10443500	0.70120900
C	2.48053800	2.63286200	-0.31057800
C	3.33935800	3.52063500	-0.96274100
C	3.36583900	4.84489500	-0.53558200
H	4.03621700	5.53732600	-1.03221100
C	2.55264900	5.29269700	0.49279500
H	2.59026800	6.32990700	0.80293700
C	1.67410800	4.42131900	1.11407000
H	1.01019000	4.73612200	1.90932300
C	4.20832700	3.06148000	-2.08757700
H	5.07395400	2.50251700	-1.72180800
H	3.65873200	2.39477100	-2.75515900
H	4.57219200	3.91496500	-2.66013300
C	3.61129200	0.50257600	-0.25182700
C	4.40687100	-0.13249900	-1.18556200
H	4.14786400	-0.07399700	-2.23368600
C	5.52445100	-0.85561500	-0.78923200
H	6.12347700	-1.34980000	-1.54132800
C	5.85290200	-0.93746500	0.55971000
C	5.05406500	-0.28907200	1.50354800
H	5.32385300	-0.36094500	2.55005600
C	3.94567300	0.42130100	1.09876400
H	3.32961200	0.92453700	1.83615900
O	-6.91325700	1.61243800	-1.04726600
C	-7.74919600	2.29828300	-0.13396900
H	-7.15847000	3.03380900	0.42586700
H	-8.17095600	1.58638400	0.58652700
C	-8.83922100	2.97254000	-0.92384100
H	-9.50708100	3.51386000	-0.25091400
H	-8.41606100	3.68263100	-1.63626900
H	-9.42640300	2.23729500	-1.47661800
O	6.91338700	-1.61226800	1.04724800
C	7.74944100	-2.29793900	0.13392400
H	7.15881900	-3.03347500	-0.42600800
H	8.17116000	-1.58592900	-0.58648500
C	8.83950000	-2.97215700	0.92378400
H	8.41638100	-3.68235700	1.63612700
H	9.50744700	-3.51334500	0.25083800
H	9.42658100	-2.23690200	1.47665700

Cartesian coordinates of the optimized geometry of **G2** (S_1) using TD-PBE0/def2-TZVP.

O	-0.59626000	-2.42825700	-1.07199200
N	-2.23589200	1.81503900	2.75895200
N	-2.47256000	-1.23811400	0.57611200
C	-0.91119900	0.65235300	0.90291300
C	-1.23373100	-0.61187600	0.33137100
C	-0.32604400	-1.22174600	-0.50970200
C	-1.65149200	1.28061000	1.90846000
C	-1.57479400	-3.18544700	-0.52744900
C	-2.52490600	-2.61889400	0.33305200
C	-3.50454600	-3.45007700	0.90478300
C	-3.51699700	-4.78953600	0.54385500
H	-4.26427900	-5.43644800	0.98964700

C	-2.59321800	-5.32481600	-0.34812600
H	-2.63505400	-6.37631000	-0.60534900
C	-1.60658800	-4.52398400	-0.88295400
H	-0.85406100	-4.90014600	-1.56446400
C	-4.48857900	-2.94099400	1.90888800
H	-5.28941700	-2.36419200	1.44017900
H	-4.00523300	-2.28539700	2.63595700
H	-4.94018400	-3.77763100	2.44300600
C	-3.66587700	-0.49458300	0.22080500
C	-4.41296200	0.20157700	1.15148800
H	-4.10738400	0.21701900	2.18776800
C	-5.54916100	0.89889100	0.76435200
H	-6.10517500	1.44521400	1.51363500
C	-5.94954100	0.89389200	-0.56816700
C	-5.20131100	0.18041200	-1.50641500
H	-5.52138700	0.18421000	-2.54138500
C	-4.07191000	-0.50348600	-1.11313600
H	-3.48975900	-1.04948700	-1.84770400
O	0.59585700	2.42801500	1.07250200
N	2.23504100	-1.81507900	-2.75898400
N	2.47231000	1.23792900	-0.57540300
C	0.91091600	-0.65271000	-0.90233800
C	1.23347500	0.61154600	-0.33070300
C	0.32580300	1.22137000	0.51029400
C	1.65082800	-1.28074100	-1.90830400
C	1.57433300	3.18516400	0.52806000
C	2.52461300	2.61860400	-0.33237900
C	3.50441000	3.44985000	-0.90389000
C	3.51678400	4.78924600	-0.54287000
H	4.26409100	5.43621100	-0.98854000
C	2.59287300	5.32450000	0.34905100
H	2.63471400	6.37597900	0.60633200
C	1.60616800	4.52368700	0.88368900
H	0.85357200	4.89980700	1.56514500
C	4.48854900	2.94093900	-1.90798200
H	5.28936200	2.36408400	-1.43930600
H	4.00530200	2.28546900	-2.63522600
H	4.94016700	3.77769300	-2.44190500
C	3.66588800	0.49455100	-0.22074700
C	4.41234500	-0.20200600	-1.15164900
H	4.10612900	-0.21787200	-2.18772700
C	5.54883900	-0.89908400	-0.76499000
H	6.10435400	-1.44569900	-1.51442900
C	5.95019100	-0.89344400	0.56724200
C	5.20263700	-0.17949900	1.50567800
H	5.52345300	-0.18278700	2.54042100
C	4.07293300	0.50417300	1.11288700
H	3.49129600	1.05050600	1.84761300
O	-7.03327800	1.53954300	-1.04309600
C	-7.81744900	2.29842500	-0.13966000
H	-7.19351400	3.06469600	0.33634000
H	-8.20859700	1.64406800	0.64942200
C	-8.94134600	2.92852900	-0.91976700
H	-9.56950700	3.52278700	-0.25309800
H	-8.55027700	3.58334900	-1.70033600
H	-9.56270600	2.16409800	-1.38958300
O	7.03426800	-1.53883600	1.04168900
C	7.81778900	-2.29818900	0.13806300
H	7.19349200	-3.06467800	-0.33710400
H	8.20838100	-1.64422400	-0.65161600
C	8.94222700	-2.92792400	0.91768400

H	8.55170200	-3.58235100	1.69885400
H	9.56989800	-3.52252500	0.25086000
H	9.56393900	-2.16327400	1.38667500

Natural Transition Orbitals and Electron density differences

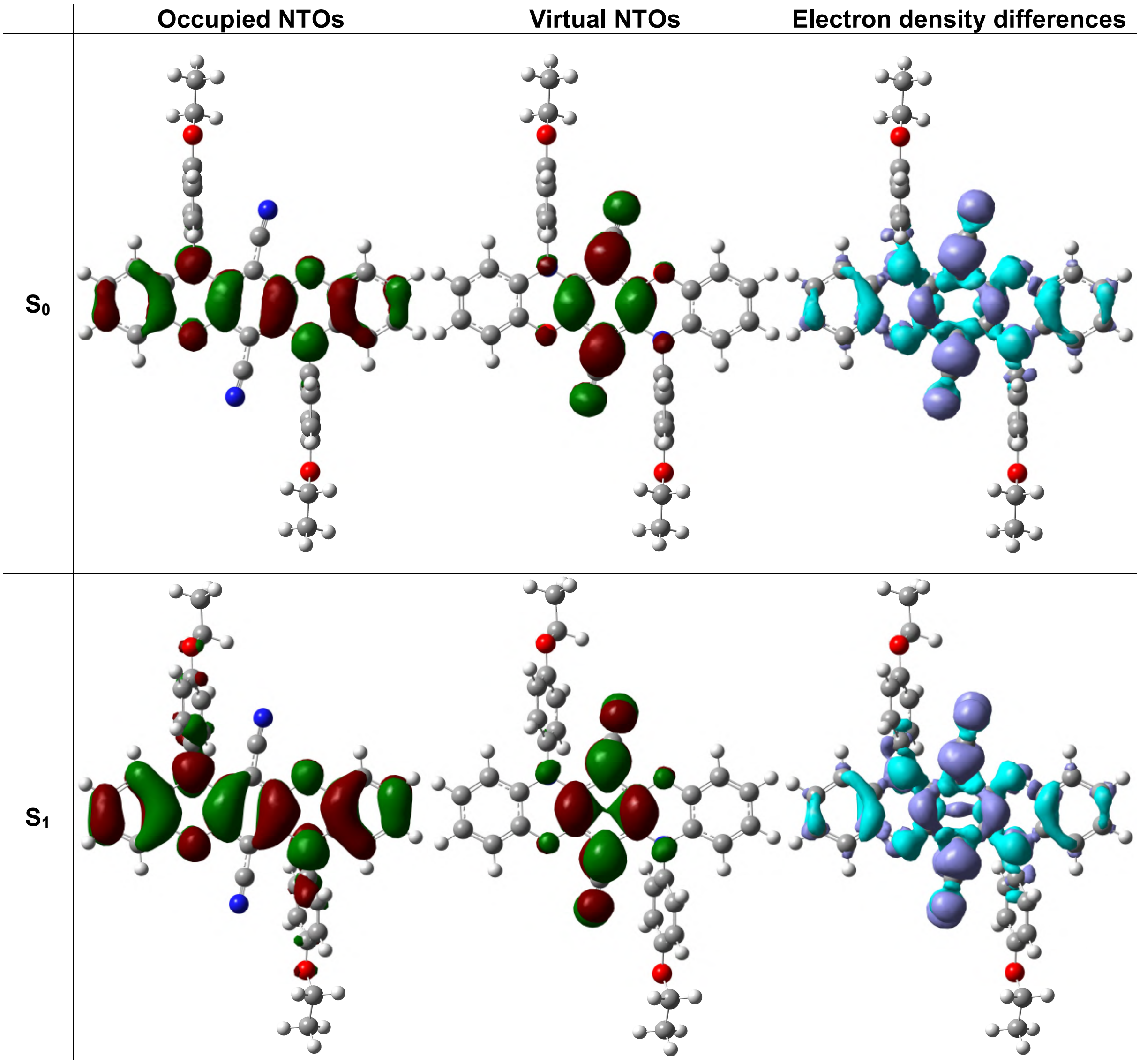

Figure S103: Occupied (left) and virtual (center) natural transition orbital pairs (isovalue = 0.030 au) and electron density differences (isovalue = 0.0008 au) between excited states and ground states of **R2**. The violet area represents an increase in electron density upon absorption, and the cyan area represents a reduction. Calculations were performed for the optimized S_0 (top) and S_1 (bottom) geometries using TD-PBE0/def2-TZVP.

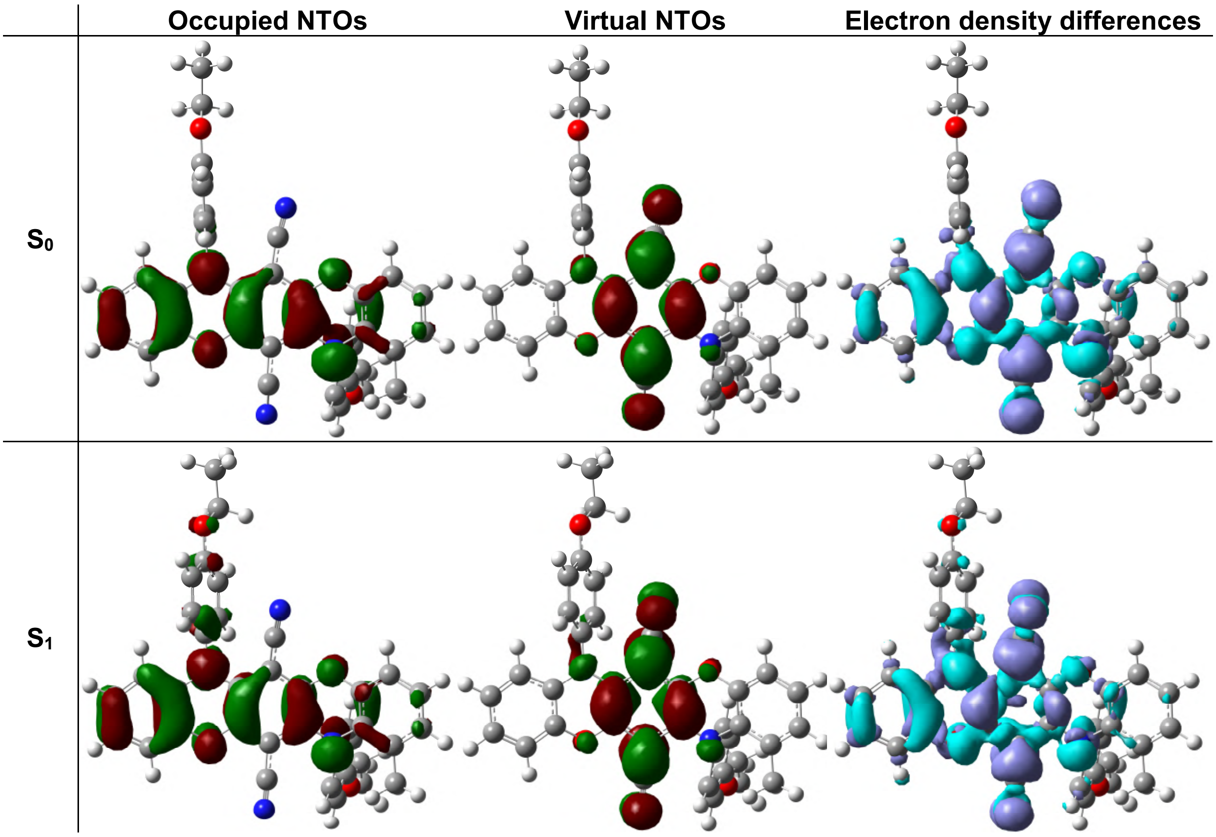

Figure S104: Occupied (left) and virtual (center) natural transition orbital pairs (isovalue = 0.030 au) and electron density differences (isovalue = 0.0008 au) between excited states and ground states of **Y2**. The violet area represents an increase in electron density upon absorption, and the cyan area represents a reduction. Calculations were performed for the optimized S_0 (top) and S_1 (bottom) geometries using TD-PBE0/def2-TZVP.

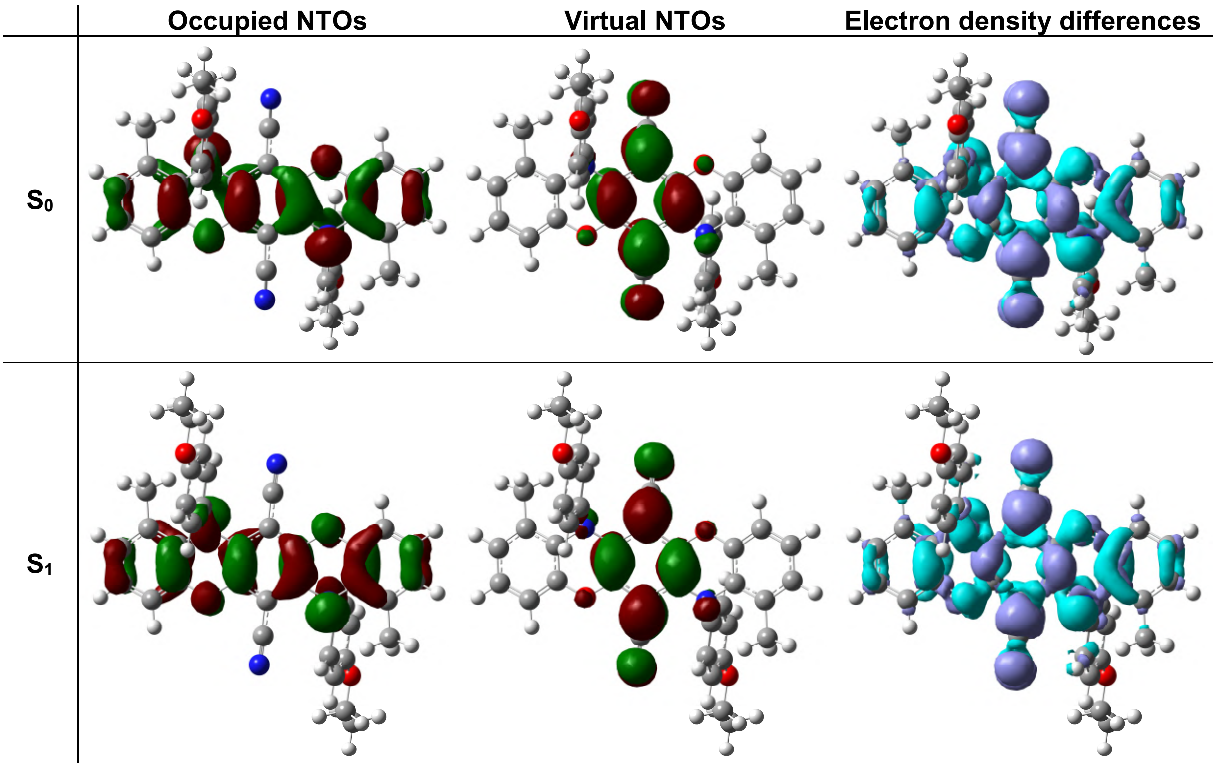

Figure S105: Occupied (left) and virtual (center) natural transition orbital pairs (isovalue = 0.030 au) and electron density differences (isovalue = 0.0008 au) between excited states and ground states of **G2**. The violet area represents an increase in electron density upon absorption, and the cyan area represents a reduction. Calculations were performed for the optimized S_0 (top) and S_1 (bottom) geometries using TD-PBE0/def2-TZVP.

9 COMPARISON WITH LITERATURE-KNOWN LUMINOPHORES

Table S13: Comparison of selected photophysical properties of the compounds **R8**, **Y8**, and **G8** with literature-known luminophores (* =measured in DCM; ** =measured in $CHCl_3$; *** = thin films; **** = DMSO; ***** = cyclohexane; ****** = Zeonex matrix; n.d. = no data reported).

Entry	Structure	Solution λ_{ab} [nm] / λ_{em} [nm] / Φ_{PL}/ τ_{av} [ns]	Powder λ_{ex} [nm] / λ_{em} [nm] / Φ_{PL}/ τ_{av} [ns]	Steric Pressure?	SSSE?	Ref
1	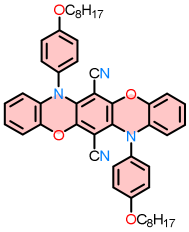 R8	508 / 597 / 0.52 / 10.7 *	574 / 610 / 0.22 / 6.6	No	No	This work
2	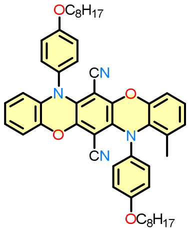 Y8	467 / 571 / 0.14 / 3.1 *	544 / 571 / 0.26 / 8.7	Yes	Yes	This work
3	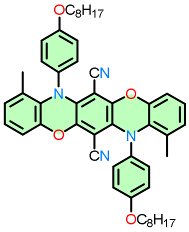 G8	428 / 564 / <0.01 / - *	473 / 499 / 0.40 / 7.0	Yes	No	This work
4	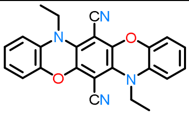	495 / 615 / 0.44 / n.d.**	495 / 630 / 0.07 / n.d. ***	No	No	Ref[16]
5	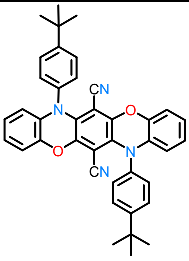	501 / 599 / 0.74 / n.d.*	n.d. / 602 / 0.07 / n.d.	No	No	Ref[17]
6	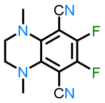	432 / 533 / 0.46 / n.d ****	n.d.	No	n/a	Ref[18]

7	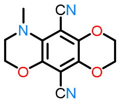	410 / 495 / 0.78 / 14.0 ****	479 / 515 / 0.32 / 12.78	No	Yes	Ref[1]
8	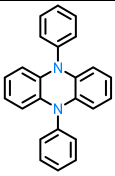	416 / 455 / 0.03 / 7.9 *****	n.d.	No	n/a	Ref[19]
9	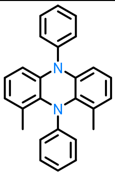	335 / 507 / 0.12 / 12.7 *****	n.d.	Yes	n/a	Ref[19]
10	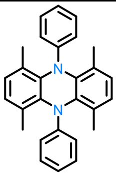	324 / 584 / 0.03 / 12.4 *****	n.d.	Yes	n/a	Ref[19]
11	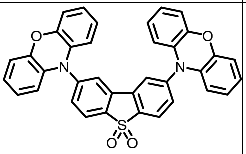	n.d.	398 / 494 / 0.42 / n.d. ******	No	n/a	Ref[20]
12	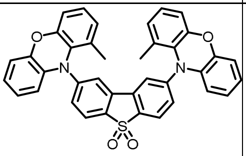	n.d.	328 / 491 / 0.19 / n.d. ******	Yes	n/a	Ref[20]

10 LITERATURE

1 A. Huber, L. Schmidt, T. Gatz, J. Bublitz, T. Rex, S. T. N. Sailaja, E. Verheggen, L. Höfmann, C. Wölper, C. A. Strassert, S. K. Knauer and J. Voskuhl, *Chem. – Eur. J.*, 2025, e202404263.
2 S. Rondeau-Gagné, C. Curutchet, F. Grenier, G. D. Scholes and J.-F. Morin, *Tetrahedron*, 2010, **66**, 4230–4242.
3 R. Y. Lorberg, S. T. Nair Sailaja, F. Terlau, M. Victoria Cappellari, M. Schmiedtchen, A. Galstyan, C. A. Strassert, M. Giese and J. Voskuhl, *Chem. – Asian J.*, 2024, e202401415.
4 C. F. Macrae, I. Sovago, S. J. Cottrell, P. T. A. Galek, P. McCabe, E. Pidcock, M. Platings, G. P. Shields, J. S. Stevens, M. Towler and P. A. Wood, *J. Appl. Crystallogr.*, 2020, **53**, 226–235.
5 G. M. Sheldrick, *Acta Crystallogr. Sect. Found. Adv.*, 2015, **A71**, 3–8.
6 G. M. Sheldrick, *Acta Crystallogr. Sect. C Struct. Chem.*, 2015, **C71**, 3–8.
7 G. M. Sheldrick, *Acta Crystallogr. Sect. A*, 1990, **A46**, 467–473.
8 C. B. Hübschle, G. M. Sheldrick and B. Dittrich, *J. Appl. Crystallogr.*, 2011, **44**, 1281–1284.
9 P. R. Spackman, M. J. Turner, J. J. McKinnon, S. K. Wolff, D. J. Grimwood, D. Jayatilaka and M. A. Spackman, *J. Appl. Crystallogr.*, 2021, **54**, 1006–1011.
10 M. J. Frisch, G. W. Trucks, H. B. Schlegel, G. E. Scuseria, M. A. Robb, J. R. Cheeseman, G. Scalmani, V. Barone, G. A., Petersson, H. Nakatsuji, X. Li, M. Caricato, A. V. Marenich, J. Bloino, B. G. Janesko, R. Gomperts, B. Mennucci, H. P., Hratchian, J. V. Ortiz, A. F. Izmaylov, J. L. Sonnenberg, D. Williams-Young, F. Ding, F. Lipparini, F. Egidi, J. Goings, B. Peng, A., Petrone, T. Henderson, D. Ranasinghe, V. G. Zakrzewski, J. Gao, N. Rega, G. Zheng, W. Liang, M. Hada, M. Ehara, K. Toyota, R. Fukuda, J. Hasegawa, M. Ishida, T. Nakajima, Y. Honda, O. Kitao, H. Nakai, T. Vreven, K. Throssell, J. A. Montgomery, Jr., J. E. Peralta, F. Ogliaro, M. J. Bearpark, J. J. Heyd, E. N. Brothers, K. N. Kudin, V. N. Staroverov, T. A. Keith, R. Kobayashi, J., Normand, K. Raghavachari, A. P. Rendell, J. C. Burant, S. S. Iyengar, J. Tomasi, M. Cossi, J. M. Millam, M. Klene, C. Adamo, and R. Cammi, J. W. Ochterski, R. L. Martin, K. Morokuma, O. Farkas, J. B. Foresman, D. J. Fox, *Gaussian 16, Revision A.03,* 2016, Gaussian, Inc., Wallingford CT.
11 S. Grimme, S. Ehrlich and L. Goerigk, *J. Comput. Chem.*, 2011, **32**, 1456–1465.
12 J. P. Perdew, K. Burke and M. Ernzerhof, *Phys. Rev. Lett.*, 1996, **77**, 3865–3868.
13 F. Weigend and R. Ahlrichs, *Phys. Chem. Chem. Phys.*, 2005, **7**, 3297–3305.
14 F. Furche and R. Ahlrichs, *J. Chem. Phys.*, 2002, **117**, 7433–7447.
15 Z. Chen, C. S. Wannere, C. Corminboeuf, R. Puchta and P. von R. Schleyer, *Chem. Rev.*, 2005, **105**, 3842–3888.
16 L. K. Hiscock, C. Yao, W. G. Skene, L. N. Dawe and K. E. Maly, *J. Org. Chem.*, 2019, **84**, 15530–15537.
17 L. K. Hiscock, V. S. Patel, A. M. Raj, C. Amoah, A. J. Talreja, W. G. Skene and K. E. Maly, *Can. J. Chem.*, 2023, **101**, 186–197.
18 A. Ashok Phadte, A. Chattopadhyay, S. Banerjee, D. Singh Sisodiya and T. Raghava, *ChemistrySelect*, 2020, **5**, 10177–10186.
19 X. Jin, S. Li, L. Guo, J. Hua, D.-H. Qu, J. Su, Z. Zhang and H. Tian, *J. Am. Chem. Soc.*, 2022, **144**, 4883–4896.
20 J. S. Ward, R. S. Nobuyasu, M. A. Fox, A. S. Batsanov, J. Santos, F. B. Dias and M. R. Bryce, *J. Org. Chem.*, 2018, **83**, 14431–14442.
